# Structure modification, antialgal, antiplasmodial, and toxic evaluations of a series of new marine-derived 14-membered resorcylic acid lactone derivatives

**DOI:** 10.1007/s42995-021-00103-0

**Published:** 2021-06-28

**Authors:** Wei-Feng Xu, Na-Na Wu, Yan-Wei Wu, Yue-Xuan Qi, Mei-Yan Wei, Laura M. Pineda, Michelle G. Ng, Carmenza Spadafora, Ji-Yong Zheng, Ling Lu, Chang-Yun Wang, Yu-Cheng Gu, Chang-Lun Shao

**Affiliations:** 1 Key Laboratory of Marine Drugs, the Ministry of Education of China, School of Medicine and Pharmacy, Ocean University of China, Qingdao, 266003 China; 2 Laboratory for Marine Drugs and Bioproducts, Qingdao National Laboratory for Marine Science and Technology, Qingdao, 266237 China; 3 State Key Laboratory for Marine Corrosion and Protection, Luoyang Ship Material Research Institute (LSMRI), Qingdao, 266237 China; 4 Center of Cellular and Molecular Biology of Diseases, Instituto de Investigaciones Científcas y Servicios de Alta Tecnología, City of Knowledge, Clayton, Apartado 0816-02852 Panama; 5 Syngenta Jealott’s Hill International Research Centre, Bracknell, Berkshire, RG42 6EY UK

**Keywords:** Marine natural product, 14-Membered resorcylic acid lactone, Zeaenol, Semisynthesis, Antialgal activity, Antiplasmodial activity

## Abstract

Marine natural products play critical roles in the chemical defense of many marine organisms and are essential, reputable sources of successful drug leads. Sixty-seven 14-membered resorcylic acid lactone derivatives **3**–**27** and **30**–**71** of the natural product zeaenol (**1**) isolated from the marine-derived fungus *Cochliobolus lunatus* were semisynthesized by chlorination, acylation, esterification, and acetalization in one to three steps. The structures of these new derivatives were established by HRESIMS and NMR techniques. All the compounds (**1**–**71**) were evaluated for their antialgal and antiplasmodial activities. Among them, 14 compounds displayed antifouling activities against adhesion of the fouling diatoms. In particular, **9** and **34** exhibited strong and selective inhibitory effects against the diatoms *Navicula laevissima* and *Navicula exigua* (EC_50_ = 6.67 and 8.55 μmol/L), respectively, which were similar in efficacy to those of the positive control SeaNine 211 (EC_50_ = 2.90 and 9.74 μmol/L). More importantly, **38**, **39**, and **69**–**71** showed potent antiplasmodial activities against *Plasmodium falciparum* with IC_50_ values ranging from 3.54 to 9.72 μmol/L. Very interestingly, the five antiplasmodial derivatives displayed non-toxicity in the cytotoxicity assays and the zebrafish embryos model, thus, representing potential promising antiplasmodial drug agents. The preliminary structure–activity relationships indicated that biphenyl substituent at C-2, acetonide at positions C-5′ and C-6′, and tri- or tetra-substituted of acyl groups increased the antiplasmodial activity. Therefore, combining evaluation of chemical ecology with pharmacological models will be implemented as a systematic strategy, not only for environmentally friendly antifoulants but also for structurally novel drugs.

**Supplementary Information:**

The online version contains supplementary material available at 10.1007/s42995-021-00103-0.

## Introduction

Marine natural products play critical roles in the chemical defense of many marine organisms and in some cases can influence the community structure of entire ecosystems (Bhadury et al. 2006; Paul et al. 2007). Marine-derived organisms have the capability to produce structurally novel and pronounced biologically active secondary metabolites that have become interesting and significant resources for drug discovery (Hou et al. 2019; Molinski et al. 2009; Shinde et al. 2019; Simmons et al. 2005). Given their core scaffolds with specific stereochemistry, related modified structures may possess potent bioactivities. Therefore, the modification of molecules derived from natural products to obtain novel bioactive derivatives has attracted significant attention.

14-Membered resorcylic acid lactones (RALs) are polyketide natural products with a 14-membered macrocyclic ring fused to a resorcylic acid residue (Jana and Nanda 2018). More than 130 naturally occurring 14-membered RALs have been described from many fungal genera since the first-discovered RAL radicicol was isolated in 1953 (Jana and Nanda 2018; Lai et al. 2016; Shen et al. 2015; Xu et al. 2014). In our previous investigation for bioactive natural 14-membered RALs, cochliomycin A with the acetonide moiety, isolated from the marine-derived fungus *Cochliobolus lunatus*, showed significant antifouling activity against the larval settlement of the barnacle *Balanus Amphitrite* at the concentration of 1.2 μg/mL (Shao et al. 2011). Molecular mechanism studies revealed that cochliomycin A affected the cytochrome P450, glutathione *S*-transferase (GST), and NO/cGMP pathways, among which the NO/cGMP pathway was considered to play a key role in barnacle larval settlement (Wang et al. 2016). Cochliomycin F and analogue LL-Z1640-2 exhibited potent antifouling activities at nontoxic concentrations (Liu et al. 2014). In addition, cochliomycin G showed strong antifouling activity against diatoms, which was similar to that of the positive control SeaNine 211 (Xu et al. 2021). Further, some of the 14-membered RALs exhibited antiplasmodial activities against *Plasmodium falciparum*. Hypothemycin, paecilomycin E, and aigialomycins D and F exhibited strong in vitro antiplasmodial activities against *P. falciparum* (Isaka et al. 2002; Xu et al. 2010). Paecilomycins A, B, and F, and aigialomycins B and D displayed moderate activities against the *P. falciparum* line 3D7 (Xu et al. 2010). Recently, a series of 14-membered RAL derivatives were semisynthesized and their biological activities evaluated. Three of them showed strong antiplasmodial activities against *P. falciparum* with IC_50_ values ranging from 1.84 to 6.95 μmol/L. Importantly, two of them were non-toxic with very safe and high therapeutic indices (CC_50_/IC_50_ > 180). To further investigate antifouling activity and antiplasmodial activity of the OH substituent groups of RALs, synthesis of the etherification products of the zeaenol were encouraging. The structure–activity relationships analysis indicated that the acetoxy and acetonide groups at C5′–C6′ had positive effects on the antiplasmodial activity (Zhang et al. 2017). Recently, total syntheses of paecilomycins E (Reddy et al. 2019) and F, cochliomycin C, 6-epi-cochliomycin C (Kadari et al. 2018), monocillin VII (Mallampudi et al. 2019), 5′-hydroxyzearalenone, 5′β-hydroxyzearalenone (Thiraporn et al. 2017), L-783290, L-783277 (Chakraborty et al. 2020), paecilomycin B, 6′-epi-paecilomycin B (Ohba and Nakata 2018), zeaenol, and 7-epi-zeaenol (Doda et al. 2019; Mohapatra et al. 2014) were accomplished and reported. Given their intriguing antifouling and antiplasmodial properties and attractive chemical structures, this family of macrolides has attracted a growing interest in the fields of medicinal chemistry and organic chemistry.

As part of our ongoing investigation of new antialgal and antiplasmodial agents, a series of new 14-membered RAL derivatives **3**–**27** and **30**–**71** of the marine-derived zeaenol (**1**) were semisynthesized. The antifouling activity against adhesion of the fouling diatoms *Phaeodactylum tricornutum* Bohlin, *Chlorella vulgaris*, *Chaetoceros socialis*, *Navicula laevissima*, and *Navicula exigua*, and the antiplasmodial activities against *P. falciparum* of all the compounds (**1**–**71**) were evaluated. The preliminary structure–activity relationships of antiplasmodial activity are also discussed.

## Results and discussion

### Chemistry

The fungal strain *Cochliobolus lunatus* (TA26-46) was cultured in a 500-mL flask containing 200 mL of liquid medium (soluble starch 10 g/L, NaNO_3_ 5 g/L, NaOAc 1 g/L, 1% salinity). The fungus was fermented at 28 °C for 9 days on a rotary shaker at 120 r/min, and the fermented liquid medium was extracted with an equal volume of EtOAc. The organic extract (23.2 g) was subjected to silica gel column chromatography (CC), Sephadex LH-20 CC, and then recrystallization to obtain **1** (7.0 g). The chemical structure of **1** was elucidated by analysis of NMR data and compared with literature (Sugawara et al. 1992).

**Fig. 1:**
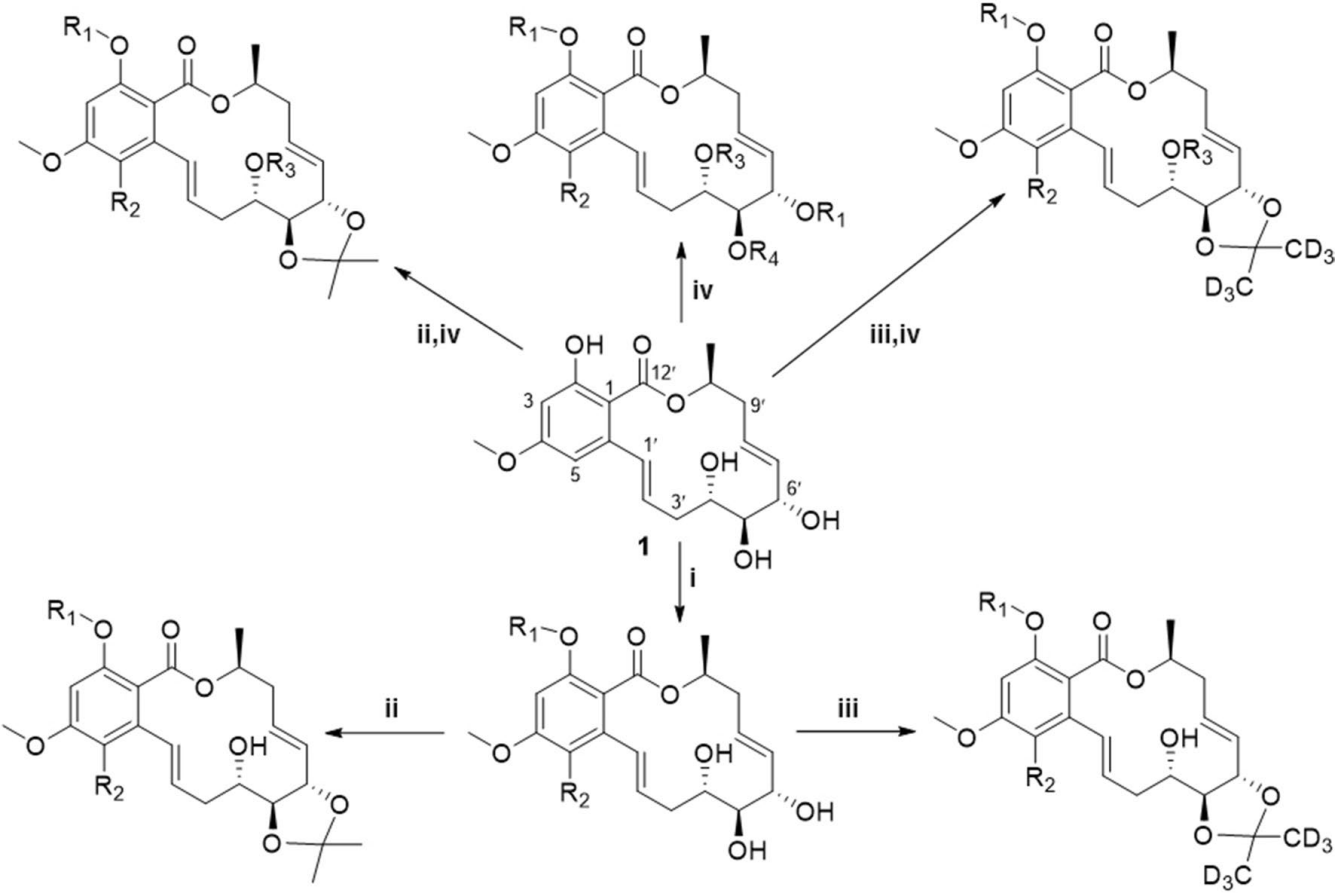
Reagents and conditions: i benzyl bromide, K_2_CO_3_, acetone, 50 °C, 24 h; ii *p*-TsOH, acetone, room temperature, 3–5 h; iii *p*-TsOH, deuterated acetone, room temperature, 3–5 h; iv anhydride or acyl chloride, K_2_CO_3_, acetone, 50 ℃, 24–36 h

The 14-membered RAL derivatives **2**–**71** (Table 1) of **1** were semisynthesized by one to three steps, and their structures were identified by extensive spectroscopic methods including HRESIMS, ^1^H NMR, and ^13^C NMR. The chlorinated derivative **2** was prepared from the chlorination of **1** with sulfuryl chloride (Yang et al. 2004). The next synthetic route was shown in Fig. 1. The derivatives **4**–**27** were formed via etherification reaction of **1** (or **2**) and benzyl bromide reagents, which were prepared as described in the literature (Bodor et al. 1983). Compound **1** (or **2**) underwent etherification reaction, and then acetal formation using a modified method from the literature with acetone/deuterated acetone to yield derivatives **28**, **29**, **37**–**55**, and **58**–**68** (Stoessl and Stothers 1983). *O*-Acylation of the hydroxyl group of **1** (or **2**) with acid anhydride or acyl chloride afforded acyl derivatives **3** and **69**–**71**. Acetal formation of **1** (or **2**) with acetone or deuterated acetone were subjected to acylation to afford derivatives **30**–**36**, **56**, and **57**.

**Table 1:**
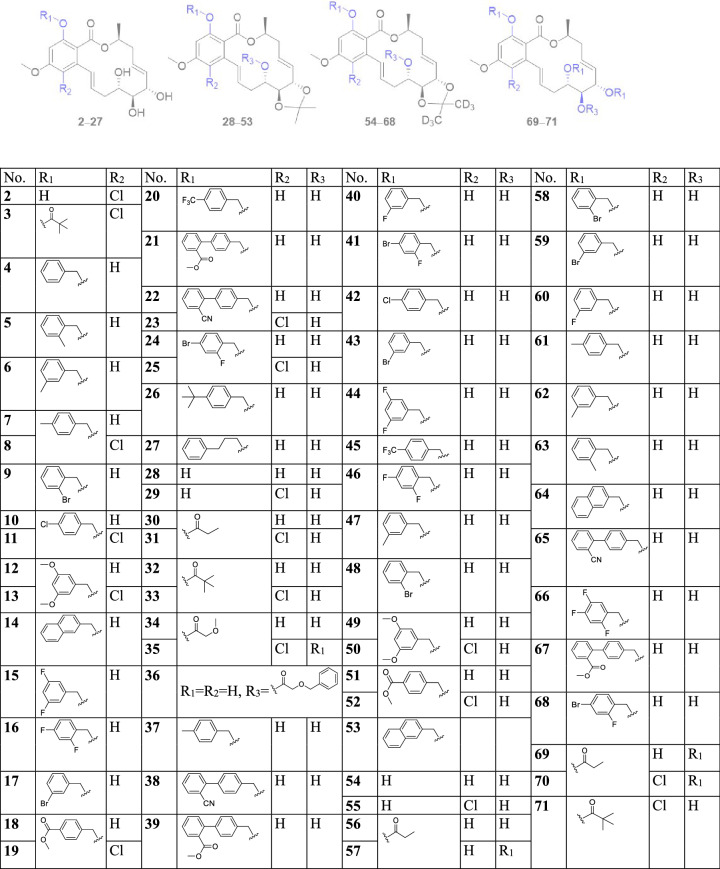
The derivatives **2**–**71** of zeaenol (**1**)

### Evaluation of biological activity

**Fig. 2:**
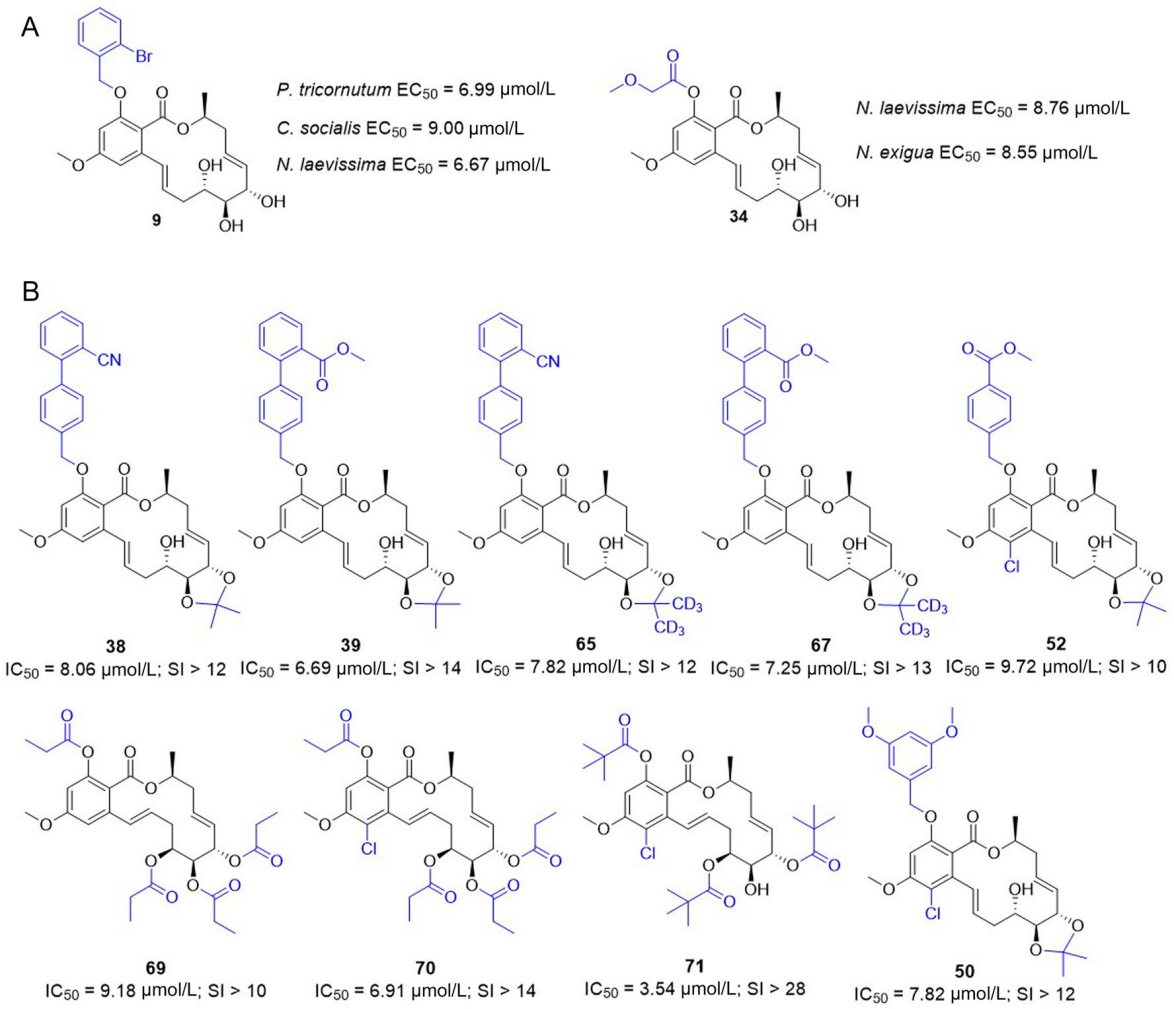
The selective derivatives with strong **A** antialgal activities or **B** antiplasmodial activities

Diatoms have been identified as biofoulers of artificial surfaces placed in marine environments, the attachment of which was thought to be the initial stage of the biofouling process (Molino and Wetherbee 2008). In addition, harmful algal blooms in eutrophic water bodies have caused serious problems with regard to effective utilization of water resources such as fisheries, water supply, and recruitment (Hachani et al. 2018). Therefore, inhibition of the adhesion of diatoms can prevent the fouling communities found on artificial surfaces, and inhibition of the entire phytoplankton community can reduce harmful algal blooms. The antialgal effects of all the compounds (**1**–**71**) against the stained diatoms *N. laevissima* and *N. exigua* and planktonic microalgae *P. tricornutum* Bohlin, *C. vulgaris*, and *C. socialis* were evaluated. As shown in Table 2, compounds **3**, **4**, **12**–**15**, **24**, **26**, **45**, **58**, and **62** displayed selective and moderate activities with EC_50_ values ranging from 11.04 to 32.55 μmol/L. The natural product zeaenol (**1**) showed moderate activity against the microalgae *P. tricornutum* Bohlin, *C. socialis*, and *N. exigua* and showed strong activity against the diatom *N. laevissima,* with an EC_50_ value of 8.79 μmol/L. Significantly, **9** exhibited selective and strong activity against the microalgae *P. tricornutum* Bohlin, *C. socialis*, and *N. laevissima* (Fig. 2), with EC_50_ values below 10.0 μmol/L, and **34** exhibited selective and strong activity against the diatoms *N. laevissima* and *N. exigua*, with EC_50_ values of 8.55 and 8.76 μmol/L, respectively, with effects similar to that of the positive control SeaNine 211 (EC_50_ = 2.90 and 9.47 μmol/L). These represent an effective inhibitory adhesion structural class of potential antifouling leads.

**Table 2:** Antialgal activities of **1** and its derivatives

Compound	EC_50_ (μmol/L)				
	*P. tricornutum*	*C. socialis*	*N. laevissima*	*N. exigua*	*C. vulgaris*
1	12.55	25.41	8.79	23.93	–
3	22.34	–	–	–	30.89
4	21.04	–	13.59	11.04	–
9	6.99	9.00	6.67	19.19	–
12	32.55	–	20.02	–	–
13	14.01	24.01	16.90	–	–
14	–	17.20	20.36	18.47	–
15	33.08	15.02	–	18.82	–
24	11.22	–	22.36	10.24	–
26	25.71	18.41	–	–	–
34	16.16	–	8.76	8.55	12.08
45	–	–	27.44	18.27	12.97
58	28.15	–	–	20.12	15.93
62	–	25.82	–	14.18	16.28
SeaNine 211	0.74	1.87	2.90	9.47	6.52

Compounds (**1**–**71**) were tested for antialgal activities; only tabulated compounds showed activities

– Inactive at 10 μmol/L

Malaria, a life-threatening mosquito-borne infection caused by protozoa of the genus *Plasmodium*, remains a major threat to public health especially in the tropical regions of the world (Ferguson et al. 2018; Miller et al. 2013). According to the World Health Organization (WHO) (2020), there are approximately 228 million new cases of malaria and 405,000 attributed deaths in 2018 (World malaria report 2019). Resistance to artemisinin-based combination therapies, the standard treatments for malaria in Africa, has emerged in southeast Asia (Conrad and Rosenthal 2019). There exists a real and urgent need for new generation of antiplasmodial drugs that can meet the threat of acquired resistance to artemisinin derivatives. The natural product zeaenol (**1**) and its derivatives **2**–**71** were evaluated for their in vitro antiplasmodial activities against *P. falciparum*, and the results are displayed in Table 3. Derivatives **38**, **39**, **50**, **52**, **65**, **67**, and **69**–**71** (Fig. 2) showed strong activity with the IC_50_ values ranging from 3.54 to 9.72 μmol/L. Structure–activity relationships showed that **69**–**71** with tri- or tetra-substituted acyl groups displayed strong antiplasmodial activities, while the mono- and di-acyl derivatives **3**, **31**, **32**, **33**, **35**, **36**, **56**, and **57** were inactive, except for **30** and **34** which showed moderate activity. The results clearly indicated that three or four acyl substituents at C-2, C-4′, C-5′, C-6′ have positive effects on activity. The introduction of the biphenyl substituents at C-2 and acetonide or deuterium acetonide groups at C-5′ and C-6′ in **38**, **39**, **65**, and **67** increases the activity remarkably, while the individual introduction of the biphenyl groups at C-2 in **21**–**23** or the acetonide and deuterium acetonide groups at C-5′ and C-6′ in **28**, **29**, **54**, and **55** were inactive. These results indicated that adding the biphenyl group at C-2 and adding acetonide group at C-5′ and C-6′ simultaneously increases activity notably. Comparing the active **60** and the inactive **57**–**59**, we found that, for the 4-methyl benzoic acid methyl ester group at C-2, the introduction of a chlorine atom at C-5 and the substitutions at C-5′ and C-6′ with an acetonide group, simultaneously, were essential for the activity. Similarly, a comparison of the active **13** and **50** and the inactive **12** and **49** indicated that, for the 3,5-dimethoxybenzyl group at C-2, the introduction of a chlorine atom at C-5 is beneficial for antiplasmodial activity.

**Table 3:** Antiplasmodial activity and cytotoxicity

No	*P. falciparum*		HUVEC (CC_50_)
	IC_50_ (μmol/L)	SI	
13	12.59	–	nt
30	12.39	–	nt
35	11.55	–	nt
38	8.06	> 12	> 100
39	6.69	> 14	> 100
50	7.82	> 12	> 100
52	9.72	> 10	> 100
65	7.82	> 12	> 100
67	7.25	> 13	> 100
69	9.18	–	nt
70	6.91	> 14	> 100
71	3.54	> 28	> 100

Chloroquine control: IC_50_ = 37.9 ± 17.8 μmol/L

*SI* Selectivity Index (CC_50_/IC_50_), *nt* not tested, – not calculated

Toxicity is a major concern of drug discovery and development, and the selectivity index (SI) is used as the evaluation parameter of drug potential of the test samples by comparing the toxicity on a human cell line (CC_50_) and the selective inhibitory effect on *P*. *falciparum* (IC_50_) calculated here as CC_50_/IC_50_. We decided to evaluate in vitro cytotoxicity of the active derivatives **38**, **39**, **50**, **52**, **65**, **67**, **70**, and **71** by determining their 50% cytotoxic concentrations. The results are presented in Table 3, presenting only those CC_50_ values > 100 μmol/L, reaching selectivity indexes > 10. The cytotoxic concentration at 150 μmol/L was not be determined because of a lack of solubility of the tested molecule in the culture medium. Moreover, the toxicity of these derivatives was also evaluated against two other cell lines: hepatocellular liver carcinoma cell line (Hep G2) and cervical carcinoma (HeLa), finding CC_50_ values > 100 μmol/L.

**Fig. 3:**
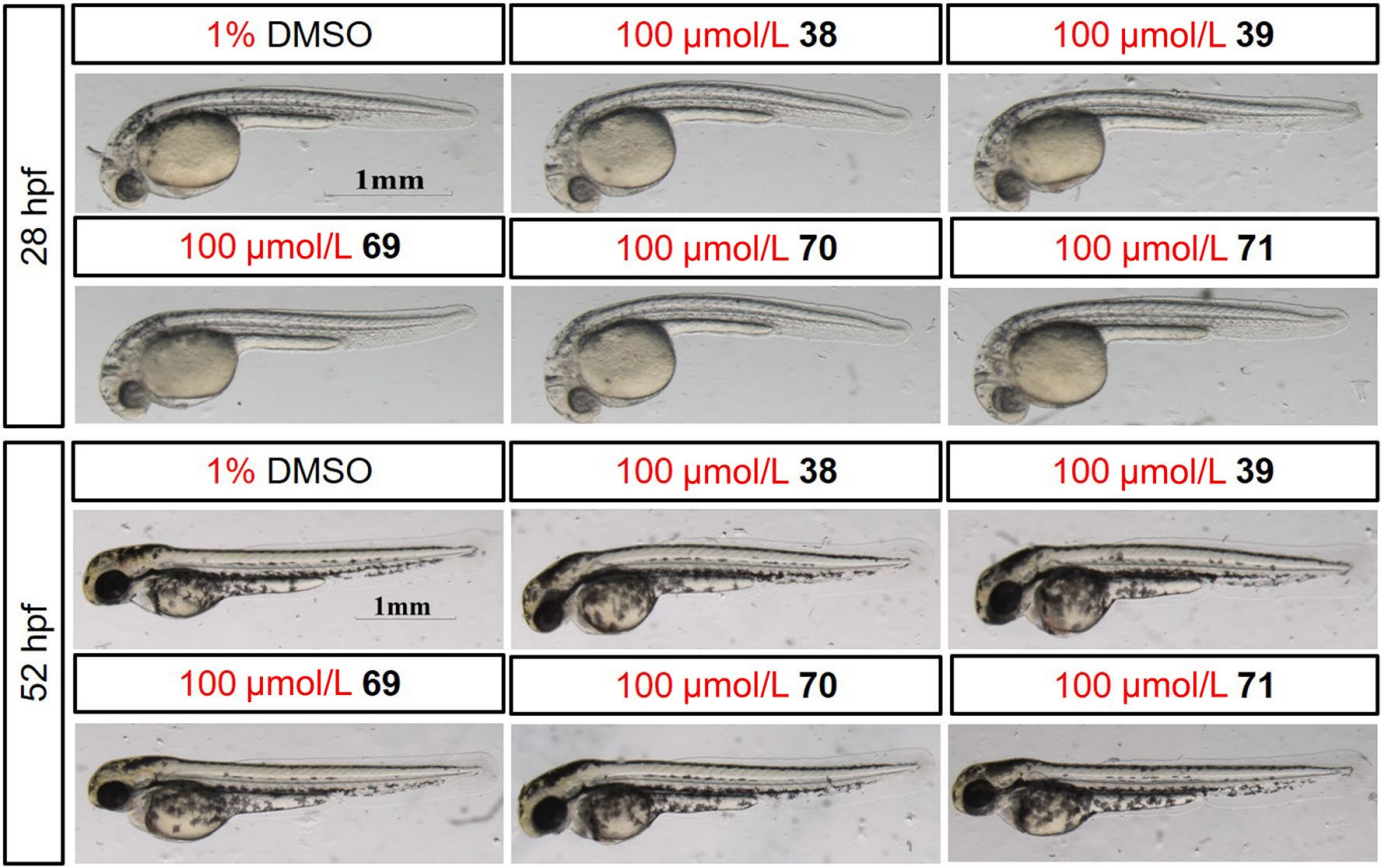
Non-toxicity effects of **38**, **39**, and **69**–**71** on the development of zebrafish embryos at 28 and 52 hpf

Zebrafish embryos serve as animal models for drug screening as well as toxicity testing due to a unique set of attributes such as small size, development outside of the mother, and cheap maintenance of the adult (Richards et al. 2008; Wang et al. 2011). The toxicity testing of the antiplasmodial derivatives **38**, **39**, and **69**–**71** was investigated using the zebrafish embryos model. All embryos survived for 52 h at the concentration of 100 μmol/L of derivatives **38**, **39**, and **69**–**71** (Fig. 3). On the contrary, all embryos died after 24 h at the concentration of 50 μmol/L of **14** and **26**. Therefore, the non-toxicity of these derivatives agreed with their in vitro toxicity against HUVEC, HepG2, and HeLa cell lines. Thus, these derivatives represent potential leads for antiplasmodial drug discovery.

Furthermore, compounds **1**–**71** were also evaluated for their antibacterial activities against *Staphylococcus aureus*, *Pseudomonas aeruginosa*, *Photobacterium halotolerans*, *Enterobacter cloacae*, and *Escherichia coli*, and antifungal activities against *Candida albicans*. However, only **26** exhibited selective antibacterial activity against *S. aureus* with a minimum inhibitory concentration (MIC) value of 25 μmol/L.

## Conclusion

In summary, a series of new14-membered RAL derivatives **3**–**27** and **30**–**71** of the natural product zeaenol (**1**) isolated from the marine-derived fungus *C. lunatus* were semisynthesized. Two derivatives (**9** and **34**) displayed strong and selective antialgal activities against the diatoms *N. laevissima* and *N. exigua*, with effects similar to those of the positive control SeaNine 211. Importantly, **38**, **39**, and **69**–**71** showed potent non-toxic antiplasmodial activities against *P. falciparum*, and their structure–activity relationships were also discussed. Our results offer important insights for the development of a new and safer class of antialgal and antiplasmodial agents.

## Materials and methods

Reagents and solvents were purchased from commercial suppliers and used without further purification. The progress of the reactions was monitored by TLC on SiO_2_. Silica gel (100–200 and 200–300 mesh particle size) was used for column chromatography. Thin layer chromatography (TLC) was precoated with silica gel GF 254 plates (Yantai Zi Fu Chemical Co., Ltd., Yantai, China). NMR spectra were obtained at 500 MHz for ^1^H and 125 MHz for ^13^C on a JEOL JEM-ECP NMR spectrometer. Chemical shifts *δ* are reported in parts per million (ppm) values, using TMS as the internal standard, and coupling constants *J* are in Hz. HRESIMS spectra were obtained from a Micromass Q-TOF spectrometer.

### General procedure for the synthesis of 2

To a solution of compound **1** (100 mg, 274.43 µmol) in dry CH_2_Cl_2_ (10 ml) was added a solution of SO_2_Cl_2_ (22.61 µl, 329.31 µmol, 1.2 equiv) in CH_2_Cl_2_ (4.0 ml) at 0 °C. After 1 h, the reaction was quenched by the addition of 15.0 ml of 5% aqueous NH_4_Cl solution. The organic phases were separated and evaporated. The residue was purified by silica gel column chromatography (EtOAc/petroleum, 9:1, v/v) to give derivative **2**.

### General procedure for the synthesis of 4–27

A mixture comprising compound **1** or** 2** (411.64 µmol, 1 equiv), benzyl bromide reagent (1.23 mmol, 3 equiv) and K_2_CO_3_ (500 mg, 3.62 mmol, 8.79 equiv) in acetone (15 ml) was stirred at 60 ℃ for 12–24 h. The reaction mixture was washed with aqueous saturated NaHCO_3_ solution. The organic layer was evaporated to dryness to leave the crude product. This was purified by silica gel column chromatography (EtOAc/petroleum, 8:2, v/v) to give the etherification derivatives **4**–**27**.

### General procedure for the synthesis of 28, 29, 37–55, and 58–68

A mixture of compound **1** or above etherification derivatives (150 µmol, 1 equiv), and *p*-toluene sulfonic acid (2.24 mg, 13 µmol, 0.1 equiv) in acetone (8 ml) was stirred at 30 ℃ for 1–3 h. Progress of the reaction was monitored by TLC. Upon completion, the reaction mixture was purified by silica gel column chromatography (EtOAc/petroleum, 3:7, v/v) to give the acetonide derivatives **28**, **29**, **37**–**52**. Similarly, the deuterated acetonide derivatives **54**, **55**, and **58**–**68** were obtained with deuterated acetone instead of acetone as a solvent.

### General procedure for the synthesis of 3, 30–36, 56, 57, 69–71

A mixture comprising compound **1** (or **2**, **28**, **29**, and **55**) (411.64 µmol, 1 equiv), acid anhydride or acyl chloride reagent (2.06 mmol, 5 equiv), and K_2_CO_3_ (500 mg, 3.62 mmol, 8.79 equiv) in acetone (15 ml) was stirred at 50 ℃ for 12–48 h. Progress of the reaction was monitored by TLC. The reaction mixture was washed with aqueous saturated NaHCO_3_ solution. The organic layer was evaporated to dryness to leave the crude product. This was purified by silica gel column chromatography to give the *O*-acylation derivatives **3**, **30**–**36**, **56**, **57**, and **69**–**71**.

### Diatom growth inhibition assay

The antialgal activity against the microalgae *P. tricornutum* Bohlin, *C. vulgaris*, *C. socialis*, *N. laevissima*, and *N. exigua* was evaluated by the method of Ortlepp et al. (2008). Briefly, the microalgae were grown in sterilized BG11 medium under light intensity of 2500 lx and 12 h: 12 h light: dark cycle at temperature of 20 ℃. The microalgae were grown to reach the logarithmic phase (approximately 5 × 10^5^ cells/ml), which were used for microalgae growth inhibition assay. The compounds **1**–**71** were first dissolved in a small amount of DMSO and then diluted with 0.22 μm of filtered seawater (FSW) to achieve a range of concentrations from 0.625 to 50 μg/ml. Ten microliters of the compound solution was added to multiwell Petri dishes containing a 1-mL suspension of the microalgae (approximately 12 × 10^4^ cells/ml). FSW with DMSO was used as a negative control, and solutions of SeaNine 211 in seawater were used as positive controls. After the plates were incubated for 48 h (16 ± 1 ℃) under continuous light, the FSW with non-attached microalgae was removed. The amount of chlorophyll *a* in attached microalgae was determined with a spectrophotometer (Beckman, model DU650, USA). Then the EC_50_ (inhibits 50% of settlement of cyprids in comparison with the control) was calculated using the Probit software program.

### Antiplasmodial assay

In vitro antiplasmodial activity was performed by culturing human erythrocytes and infecting them with *P. falciparum*, as reported by Trager and Jensen (1976). Briefly, the chloroquine sensitive HB3 strain of *P. falciparum*-infected O + erythrocytes was cultured in RPMI 1640 medium supplemented with 10% human serum (from O + blood) at a hematocrit of 2% erythrocytes at 37 °C with a gas mixture containing 90% N_2_, 5% CO_2_, and 5% O_2_. Parasites were synchronized with 5% d-sorbitol (Lambros and Vanderberg 1979). *P. falciparum* growth inhibition bioassays were performed with the procedure as described by Corbett et al. (2004), which used Pico-Green, a double stranded-DNA fluorescent dye to test the effect of compounds. Chloroquine was used as positive control, with an IC_50_ value of 32.9 nmol/L.

### Chemical treatment and phenotype observation in toxicity test

Toxicity assays were carried out using the method of Wang et al. (2011). Briefly, the normal embryos were selected and transferred into a 24-well plate. The embryos at 1 h postfertilization (hpf) were exposed by immersion in E3 embryo medium containing the derivative in a 1% (v/v) DMSO final solvent concentration in a 24-well plate. The developmental phenotypes of the experimental status of the embryos were observed every 24 h for 72 h and were photographed with a charge-coupled device (CCD) camera.

### Antimicrobial assay

The antimicrobial activities were carried out by a serial dilution technique using 96-well microtiter plates (Pierce et al. 2008). The derivatives were dissolved in DMSO to obtain a stock solution. Microbial species were cultured overnight at 37 °C in LB broth and diluted to 10^6^ cfu/ml when used. LB broth was used as a blank control, and DMSO was used as a negative control, while ciprofloxacin was used as a positive control. The plates were incubated at 37 °C for 24 h.

## Supplementary Information

Below is the link to the electronic supplementary material.

Supplementary file1 (DOCX 18434 kb)
