# Supplementary material for: Structure modification, antialgal, antiplasmodial, and toxic evaluations of a series of new marine-derived 14-membered resorcylic acid lactone derivatives
Source: Mar Life Sci Technol. 2021 Jun 28;4(1):88–97. doi: 10.1007/s42995-021-00103-0 (PMC10077203; doi:10.1007/s42995-021-00103-0)
Supplement: Supplementary file 1 — Supplementary file1 (DOCX 18434 kb) [file 42995_2021_103_MOESM1_ESM.docx]

**Structure modification,** **antialgal, antiplasmodial and toxic evaluations of a series of new marine-derived 14-membered resorcylic acid lactone derivatives**

Wei-Feng Xu^1,2,3^ **·** Na-Na Wu^1,2,3^ **·** Yan-Wei Wu^1,2^ **·** Yue-Xuan Qi^1,2,3^ **·** Mei-Yan Wei^1,2^ **·** Laura M. Pineda^4^ **·** Michelle G. Ng^4^ **·** Carmenza Spadafora*^,4^ **·** Ji-Yong Zheng*^,3^ **·** Ling Lu^1,2^ **·** Chang-Yun Wang^1,2^ **·** Yu-Cheng Gu^5^ **·** Chang-Lun Shao*^,1,2,3^

1 Key Laboratory of Marine Drugs, the Ministry of Education of China, School of Medicine and Pharmacy, Ocean University of China, Qingdao 266003, China

2 Laboratory for Marine Drugs and Bioproducts, Qingdao National Laboratory for Marine Science and Technology, Qingdao 266237, China

3 State Key Laboratory for Marine Corrosion and Protection, Luoyang Ship Material Research Institute (LSMRI), Qingdao 266061, China

4 Center of Cellular and Molecular Biology of Diseases, Instituto de Investigaciones Científcas y Servicios de Alta Tecnología, City of Knowledge, Clayton, Apartado, 0816-02852, Panama

5 Syngenta Jealott’s Hill International Research Centre, Bracknell, Berkshire, RG42 6EY, United Kingdom

This Paper is Dedicated to Professor Youyou Tu, the 2015 Nobel Prize Laureate of

Physiology or Medicine on the Occasion of Her 90th Birthday

* Correspondence: shaochanglun@163.com (Chang-Lun Shao); zhengjy@sunrui.net (Ji-Yong Zheng); cspadafora@indicasat.org.pa (Carmenza Spadafora)

**Compound 1**: White, amorphous powder; ^1^H NMR (500 MHz, DMSO-*d*_6_): *δ* ppm 11.23 (s, 1H), 6.83 (d, *J* = 15.6 Hz, 1H), 6.42 (d, *J* = 2.2 Hz, 1H), 6.34 (d, *J* = 2.2 Hz, 1H), 6.05 (m, 1H), 5.76 (m, 1H), 5.53 (dd, *J* = 15.4, 7.3 Hz, 1H), 5.12 (m, 1H), 4.66 (d, *J* = 4.0 Hz, 1H), 4.56 (d, *J* = 3.8 Hz, 1H), 4.52 (d, *J* = 5.8 Hz, 1H), 3.85 (m, 1H), 3.75 (s, 3H), 3.49 (s, 1H), 3.35 (s, 1H), 2.45 (m, 1H), 2.39 (m, 1H), 2.32 (m, 1H), 2.09 (m, 1H), 1.35 (d, *J* = 6.2 Hz, 3H); ^13^C NMR (125 MHz, DMSO-*d*_6_): *δ* 170.2, 163.1, 162.3, 141.8, 133.2, 132.8, 130.9, 126.9, 106.9, 105.3, 100.4, 78.5, 73.9, 72.7, 72.6, 55.8, 37.2, 35.9, 19.7.

**Compound 2**: White, amorphous powder; ^1^H NMR (500 MHz, CDCl_3_): *δ* ppm 11.36 (s, 1H), 6.54 (d, *J* = 16.2 Hz, 1H), 6.43 (s, 1H), 5.90 (dt, *J* = 15.4, 5.8 Hz, 1H), 5.62–5.49 (overlapped, 2H), 5.27 (m, 1H), 4.20 (t, *J* = 8.3 Hz, 1H), 3.92 (t, *J* = 7.2 Hz, 1H), 3.88 (s, 3H), 3.56 (d, *J* = 8.3 Hz, 1H), 2.59 (m, 1H), 2.50–2.24 (overlapped, 3H), 1.36 (d, *J* = 6.2 Hz, 3H); ^13^C NMR (125 MHz, CDCl_3_): *δ* 170.7, 162.1, 159.6, 139.9, 131.9, 131.4, 129.5, 128.0, 113.8, 106.4, 99.3, 76.2, 72.2, 72.1, 72.0, 56.3, 37.1, 35.2, 19.7; HRESIMS *m*/*z* 397.1064 [M – H]^–^ (calcd for C_19_H_22_O_7_Cl, 397.1054); 86% yield.

**Compound 3**: Colorless oil; ^1^H NMR (500 MHz, CDCl_3_): *δ* ppm 6.57 (s, 1H), 6.54 (d, *J* = 15.8 Hz, 1H), 5.86 (m, 1H), 5.79 (m, 1H), 5.49 (dd, *J* = 15.8, 8.5 Hz, 1H), 4.91 (m, 1H), 4.09 (t, *J* = 8.8 Hz, 1H), 3.89 (s, 3H), 3.68–3.60 (overlapped, 2H), 2.58 (m, 1H), 2.41–2.31 (overlapped, 2H), 2.20 (m, 1H), 1.33 (s, 9H), 1.31 (d, *J* = 6.4 Hz, 3H); ^13^C NMR (125 MHz, CDCl_3_): *δ* 176.2, 165.6, 156.0, 147.3, 136.8, 134.9, 132.1, 131.2, 126.8, 120.4, 119.0, 105.2, 78.1, 74.6, 74.1, 71.8, 56.5, 39.1, 38.1, 36.2, 27.0, 21.0; HRESIMS *m*/*z* 483.1791 [M + H]^+^ (calcd for C_24_H_32_O_8_Cl, 483.1780); 43% yield.

**Compound 4**: White, amorphous powder; ^1^H NMR (500 MHz, CDCl_3_): *δ* ppm 7.36 (d, *J* = 7.4 Hz, 2H), 7.32 (t, *J* = 7.4 Hz, 2H), 7.26 (t, *J* = 7.4 Hz, 1H), 6.52 (d, *J* = 15.8 Hz, 1H), 6.41 (d, *J* = 1.8 Hz, 1H), 6.37 (d, *J* = 1.8 Hz, 1H), 6.04 (ddd, *J* = 13.8, 8.2, 5.4 Hz, 1H), 5.77 (m, 1H), 5.55 (dd, *J* = 15.8, 7.8 Hz, 1H), 5.08 (m, 1H), 5.04 (d, *J* = 12.6 Hz, 1H), 5.01 (d, *J* = 12.6 Hz, 1H), 4.02 (d, *J* = 8.0 Hz, 1H), 3.86 (br s, 1H), 3.71 (s, 3H), 3.66 (d, *J* = 8.0 Hz, 1H), 2.53 (m, 1H), 2.37–2.25 (overlapped, 3H), 1.24 (d, *J* = 6.2 Hz, 3H); ^13^C NMR (125 MHz, CDCl_3_): *δ* 167.8, 161.1, 156.8, 137.8, 136.5, 131.6, 131.5, 131.0, 129.3, 128.4, 127.8, 127.0, 116.1, 103.3, 98.9, 77.2, 72.9, 72.9, 72.0, 70.4, 55.3, 38.0, 35.2, 20.5; HRESIMS *m*/*z* 455.2067 [M + H]^+^ (calcd for C_26_H_31_O_7_, 455.2064); 89% yield.

**Compound 5**: White, amorphous powder; ^1^H NMR (500 MHz, CDCl_3_): *δ* ppm 7.38 (d, *J* = 7.4 Hz, 1H), 7.23–7.16 (overlapped, 3H), 6.57 (d, *J* = 15.8 Hz, 1H), 6.44–6.43 (overlapped, 2H), 6.03 (m, 1H), 5.82 (m, 1H), 5.58 (dd, *J* = 15.8, 7.6 Hz, 1H), 5.15 (m, 1H), 5.03 (d, *J* = 12.0 Hz, 1H), 5.00 (d, *J* = 12.0Hz, 1H), 4.11 (8, *J* = 8.0 Hz, 1H), 3.93 (br s, 1H), 3.78 (s, 3H), 3.66 (d, *J* = 8.0 Hz, 1H), 2.56 (dt, *J* = 15.2, 7.7 Hz, 1H), 2.42–2.29 (overlapped, 6H), 1.21 (d, *J* = 6.2 Hz, 3H); ^13^C NMR (125 MHz, CDCl_3_): *δ* 167.6, 161.2, 157.0, 137.7, 136.2, 134.3, 131.4, 131.3, 130.6, 130.1, 129.8, 128.1, 128.0, 125.8, 116.0, 103.0, 98.8, 76.6, 72.3, 72.3, 72.0, 69.0, 55.4, 38.1, 35.4, 20.5, 18.8; HRESIMS *m*/*z* 469.2229 [M + H]^+^ (calcd for C_27_H_33_O_7_, 469.2221); 77% yield.

**Compound 6**: White, amorphous powder; ^1^H NMR (500 MHz, CDCl_3_): *δ* ppm 7.23–7.15 (overlapped, 3H), 7.08 (d, *J* = 7.4 Hz, 1H), 6.53 (d, *J* = 15.8 Hz, 1H), 6.41 (d, *J* = 1.9 Hz, 1H), 6.38 (d, *J* = 1.9 Hz, 1H), 6.04 (ddd, *J* = 13.8, 8.0, 5.4 Hz, 1H), 5.78 (m, 1H), 5.56 (dd, *J* = 15.8, 7.8 Hz, 1H), 5.11 (m, 1H), 5.00 (s, 2H), 4.04 (t, *J* = 8.0 Hz, 1H), 3.88 (t, *J* = 8.0 Hz, 1H), 3.73 (s, 3H), 3.66 (d, *J* = 8.0 Hz, 1H), 2.54 (m, 1H), 2.42–2.26 (overlapped, 6H), 1.27 (d, *J* = 6.2 Hz, 3H); ^13^C NMR (125 MHz, CDCl_3_): *δ* 167.9, 161.1, 156.9, 138.0, 137.7, 136.4, 131.5, 131.3, 131.2, 129.4, 128.5, 128.3, 127.7, 124.1, 116.1, 103.2, 98.9, 77.1, 72.8, 72.7, 72.0, 70.4, 55.3, 38.0, 35.3, 21.3, 20.6; HRESIMS *m*/*z* 469.2228 [M + H]^+^ (calcd for C_27_H_33_O_7_, 469.2221); 84% yield.

**Compound 7**: White, amorphous powder; ^1^H NMR (500 MHz, CDCl_3_): *δ* ppm 7.27 (d, *J* = 7.8 Hz, 2H), 7.15 (d, *J* = 7.8 Hz, 2H), 6.58 (d, *J* = 16.0 Hz, 1H), 6.42 (d, *J* = 2.0 Hz, 1H), 6.40 (d, *J* = 2.0 Hz, 1H), 6.03 (m, 1H), 5.85 (m, 1H), 5.60 (dd, *J* = 15.8, 7.6 Hz, 1H), 5.20 (m, 1H), 5.02 (s, 2H), 4.12 (br s, 1H), 3.96 (br s, 1H), 3.77 (s, 3H), 3.67 (br s, 1H), 2.57 (dt, *J* = 15.4, 7.8 Hz, 1H), 2.38–2.36 (overlapped, 3H), 2.33 (s, 3H), 1.29 (d, *J* = 6.2 Hz, 3H); ^13^C NMR (125 MHz, CDCl_3_): *δ* ppm 167.6, 161.2, 157.0, 137.6, 137.5, 133.4, 131.5, 131.2, 130.4, 129.9, 129.0, 127.1, 116.1, 103.2, 99.0, 76.4, 72.2, 72.1, 72.0, 70.4, 55.4, 38.1, 35.4, 21.1, 20.6. ^13^C NMR (125 MHz, CDCl_3_): *δ* ppm 167.6, 161.2, 157.0, 137.6, 137.5, 133.4, 131.5, 131.2, 130.4, 129.9, 129.0, 127.1, 116.1, 103.2, 99.0, 76.4, 72.2, 72.1, 72.0, 70.4, 55.4, 38.1, 35.4, 21.1, 20.6; HRESIMS *m*/*z* 469.2229 [M + H]^+^ (calcd for C_27_H_33_O_7_, 469.2221); 89% yield.

**Compound 8**: White, amorphous powder; ^1^H NMR (500 MHz, CDCl_3_): *δ* ppm 7.03 (dd, *J* = 15.6, 1.4 Hz, 1H), 6.76 (d, *J* = 2.4 Hz, 1H), 6.53 (d, *J* = 2.4 Hz, 1H), 5.98 (dt, *J* = 15.6, 5.1 Hz, 1H), 5.80 (m, 1H), 5.48 (dd, *J* = 15.6, 8.9 Hz, 1H), 5.41 (m, 1H), 4.54 (t, *J* = 8.0 Hz, 1H), 4.14 (m, 1H), 3.86 (dd, *J* = 8.0, 2.1 Hz, 1H), 3.80 (s, 3H), 2.65 (m, 1H), 2.58 (q, *J* = 7.6 Hz, 2H), 2.50–2.46 (overlapped, 2H), 2.27 (dd, *J* = 25.2, 12.0 Hz, 1H), 1.42 (s, 3H), 1.36 (d, *J* = 6.4 Hz, 3H), 1.35 (s, 3H), 1.24 (t, *J* = 7.6 Hz, 3H); ^13^C NMR (125 MHz, CDCl_3_): *δ* 172.7, 165.0, 161.5, 151.2, 139.7, 132.7, 131.2, 130.3, 128.0, 116.9, 110.1, 108.4, 107.7, 81.0, 75.7, 69.3, 68.4, 55.5, 37.0, 36.0, 27.5, 26.9, 26.9, 19.9, 8.8; HRESIMS *m*/*z* 485.1733 [M–H_2_O + H]^+^ (calcd for C_27_H_30_O_6_Cl, 485.1725); 82% yield.

**Compound 9**: White, amorphous powder; ^1^H NMR (500 MHz, CDCl_3_): *δ* ppm 7.53 (d, *J* = 7.8 Hz, 2H), 7.28 (m, 1H), 7.14 (t, *J* = 7.8 Hz, 1H), 6.57 (d, *J* = 15.8 Hz, 1H), 6.43 (s, 1H), 6.39 (s, 1H), 6.04 (m, 1H), 5.81 (m, 1H), 5.59 (dd, *J* = 15.8, 7.8 Hz, 1H), 5.16 (m, 1H), 5.10 (s, 2H), 4.07 (t, *J* = 8.0 Hz, 1H), 3.91 (t, *J* = 8.0 Hz, 1H), 3.75 (s, 3H), 3.67 (d, *J* = 8.0 Hz, 1H), 2.54 (m, 1H), 2.38–2.29 (overlapped, 3H), 1.30 (d, *J* = 6.4 Hz, 3H); ^13^C NMR (125 MHz, CDCl_3_): *δ* ppm 167.7, 161.2, 156.5, 137.9, 135.7, 132.3, 131.5, 131.1, 131.1, 129.5, 129.1, 128.5, 127.5, 121.7, 115.9, 103.5, 98.8, 72.7, 72.6, 72.0, 69.6, 60.4, 55.4, 38.0, 35.3, 20.6; HRESIMS *m*/*z* 533.1185 [M + H]^+^ (calcd for C_26_H_30_O_7_Br, 533.1169); 86% yield.

**Compound 10**: White, amorphous powder; ^1^H NMR (500 MHz, CDCl_3_): *δ* ppm 7.38–7.29 (overlapped, 4H), 6.61 (d, *J* = 16.0 Hz, 1H), 6.45 (d, *J* = 2.1 Hz, 1H), 6.38 (d, *J* = 2.1 Hz, 1H), 6.04 (ddd, *J* = 16.0, 8.4, 5.3 Hz, 1H), 5.87 (dt, *J* = 15.8, 6.6 Hz, 1H), 5.61 (dd, *J* = 15.8, 7.7 Hz, 1H), 5.22 (dq, *J* = 12.6, 6.2 Hz, 1H), 5.03 (s, 2H), 4.15 (t, *J* = 8.0 Hz, 1H), 3.98 (br s, 1H), 3.79 (s, 3H), 3.68 (d, *J* = 8.0 Hz, 1H), 2.58 (m, 1H), 2.42–2.38 (overlapped, 3H), 1.30 (d, *J* = 6.2 Hz, 3H); ^13^C NMR (125 MHz, CDCl_3_): *δ* 167.3, 161.2, 156.7, 137.8, 134.9, 133.6, 131.5, 131.2, 130.3, 129.9, 128.6, 128.3, 109.9, 103.3, 99.1, 76.4, 72.1, 71.9, 71.9, 69.7, 55.4, 38.1, 35.5, 20.6; HRESIMS *m*/*z* 489.1685 [M + H]^+^ (calcd for C_26_H_30_O_7_Cl, 489.1675); 88% yield.

**Compound 11**: White, amorphous powder; ^1^H NMR (500 MHz, CDCl_3_, DMSO-*d*_6_): *δ* ppm 6.97 (br s, 4H), 6.15 (s, 1H), 6.07 (d, *J* = 15.6 Hz, 1H), 5.60 (m, 1H), 5.35 (m, 1H), 5.13 (dd, *J* = 15.6, 8.6 Hz, 1H), 4.70 (s, 2H), 4.56 (br s, 1H), 3.55 (m, 1H), 3.51 (s, 3H), 3.28–3.17 (overlapped, 2H), 2.26 (m, 1H), 1.91–1.89 (overlapped, 2H), 1.82 (m, 1H), 0.82 (d, *J* = 6.2 Hz, 3H); ^13^C NMR (125 MHz, CDCl_3_, DMSO-*d*_6_): *δ* 166.2, 155.9, 154.4, 135.9, 135.6, 134.7, 133.6, 132.0, 131.4, 128.7, 128.5, 125.9, 117.9, 113.5, 96.6, 78.7, 74.3, 74.1, 71.4, 70.1, 56.4, 38.0, 36.8, 20.7; HRESIMS *m*/*z* 521.1144 [M – H]^–^ (calcd for C_26_H_27_O_7_Cl_2_, 521.1128); 85% yield.

**Compound 12**: White, amorphous powder; ^1^H NMR (500 MHz, CDCl_3_): *δ* ppm 6.54 (d, *J* = 1.9 Hz, 2H), 6.53 (d, *J* = 16.0 Hz, 1H), 6.40 (d, *J* = 1.6 Hz, 1H), 6.36–6.35 (overlapped, 2H), 6.01 (m, 1H), 5.78 (m, 1H), 5.54 (dd, *J* = 15.8, 7.8 Hz, 1H), 5.12 (m, 1H), 4.99 (d, *J* = 14.0 Hz, 1H), 4.96 (d, *J* = 14.0 Hz, 1H), 4.04 (t, *J* = 8.0 Hz, 1H), 3.86 (br s, 1H), 3.75 (s, 6H), 3.72 (s, 3H), 3.63 (d, *J* = 8.0 Hz, 1H), 2.51 (m, 1H), 2.33–2.27 (overlapped, 3H), 1.28 (d, *J* = 6.4 Hz, 3H); ^13^C NMR (125 MHz, CDCl_3_): *δ* 167.7, 161.1, 160.8, 156.8, 138.9, 137.8, 131.5, 131.2, 131.0, 129.5, 116.0, 104.7, 103.3, 99.7, 99.0, 77.0, 72.6, 72.6, 72.0, 70.3, 55.3, 55.3, 38.0, 35.3, 20.6; HRESIMS *m*/*z* 515.2288 [M + H]^+^ (calcd for C_28_H_35_O_9_, 515.2276); 89% yield.

**Compound 13**: White, amorphous powder; ^1^H NMR (500 MHz, CDCl_3_): *δ* ppm 6.55 (s, 2H), 6.48 (d, *J* = 16.6 Hz, 1H), 6.46 (s, 1H), 6.39 (s, 1H), 5.94 (m, 1H), 5.80 (m, 1H), 5.54 (dd, *J* = 15.6, 8.4 Hz, 1H), 5.10–4.94 (overlapped, 3H), 4.07 (t, *J* = 7.6 Hz, 1H), 3.85 (s, 3H), 3.78 (s, 6H), 3.71–3.68 (overlapped, 2H), 2.66 (m, 1H), 2.44–2.27 (overlapped, 2H), 2.20 (dd, *J* = 21.2, 9.2 Hz, 1H), 1.23 (d, *J* = 6.0 Hz, 3H); ^13^C NMR (125 MHz, CDCl_3_): *δ* ppm 166.7, 160.9, 156.0, 154.6, 138.4, 135.8, 134.4, 132.8, 130.8, 126.7, 117.9, 112.8, 105.0, 99.9, 96.8, 78.2, 73.9, 73.8, 71.7, 70.9, 56.3, 55.3, 38.0, 36.4, 20.8; HRESIMS *m*/*z* 549.1894 [M + H]^+^ (calcd for C_28_H_34_O_9_Cl, 549.1886); 79% yield.

**Compound 14**: White, amorphous powder; ^1^H NMR (500 MHz, CDCl_3_): *δ* ppm 7.87–7.76 (overlapped, 4H), 7.54–7.43 (overlapped, 3H), 6.60 (d, *J* = 16.0 Hz, 1H), 6.46 (d, *J* = 1.8 Hz, 1H), 6.44 (d, *J* = 1.8 Hz, 1H), 6.05 (ddd, *J* = 15.8, 8.2, 5.4 Hz, 1H), 5.86 (m, 1H), 5.61 (dd, *J* = 15.8, 7.7 Hz, 1H), 5.23–5.18 (overlapped, 3H), 4.13 (t, *J* = 7.8 Hz, 1H), 3.97 (br s, 1H), 3.77 (s, 3H), 3.68 (d, *J* = 7.8 Hz, 1H), 3.24 (s, 1H), 2.80 (s, 1H), 2.73 (s, 1H), 2.59 (dt, *J* = 15.6, 7.9 Hz, 1H), 2.44–2.34 (overlapped, 3H), 1.30 (d, *J* = 6.2 Hz, 3H); ^13^C NMR (125 MHz, CDCl_3_): *δ* ppm 167.5, 161.2, 156.9, 137.7, 133.9, 133.2, 133.0, 131.5, 131.2, 130.4, 129.9, 128.2, 127.8, 127.7, 126.1, 126.0, 125.9, 124.9, 116.1, 103.3, 99.1, 76.5, 72.2, 72.1, 71.9, 70.6, 55.4, 38.1, 35.5, 20.7; HRESIMS *m*/*z* 505.2233 [M + H]^+^ (calcd for C_30_H_33_O_7_, 505.2221); 83% yield.

**Compound 15**: White, amorphous powder; ^1^H NMR (500 MHz, CDCl_3_): *δ* ppm 6.94 (d, *J* = 6.2 Hz, 2H), 6.73 (t, *J* = 8.8 Hz, 1H), 6.62 (d, *J* = 15.8 Hz, 1H), 6.47 (s, 1H), 6.35 (s, 1H), 6.05 (m, 1H), 5.88 (m, 1H), 5.62 (dd, *J* = 15.8, 7.8 Hz, 1H), 5.24 (m, 1H), 5.04 (s, 2H), 4.14 (t, *J* = 7.8 Hz, 1H), 3.98 (br s, 1H), 3.79 (s, 3H), 3.68 (d, *J* = 7.8 Hz, 1H), 2.59 (m, 1H), 2.41–2.40 (overlapped, 3H), 1.36 (d, *J* = 6.2 Hz, 3H); ^13^C NMR (125 MHz, CDCl_3_): *δ* 167.2, 161.2, 156.4, 140.5, 138.0, 131.5, 131.2, 130.5, 129.9, 116.0, 109.4, 105.0, 103.5, 103.1, 99.0, 76.4, 72.1, 72.1, 71.9, 69.1, 55.4, 38.1, 35.4, 20.6; HRESIMS *m*/*z* 491.1884 [M + H]^+^ (calcd for C_26_H_29_F_2_O_7_, 491.1876); 88% yield.

**Compound 16**: White, amorphous powder; ^1^H NMR (500 MHz, CDCl_3_): *δ* ppm 7.45 (dd, *J* = 15.0, 8.4 Hz, 1H), 6.86 (t, *J* = 8.4 Hz, 1H), 6.80 (t, *J* = 10.6 Hz, 1H), 6.55 (d, *J* = 16.0 Hz, 1H), 6.44 (d, *J* = 1.9 Hz, 1H), 6.41 (d, *J* = 1.9 Hz, 1H), 6.02 (ddd, *J* = 15.8, 8.4, 5.4 Hz, 1H), 5.80 (m, 1H), 5.58 (dd, *J* = 15.8, 7.8 Hz, 1H), 5.14 (m, 1H), 5.08 (d, *J* = 12.0 Hz, 1H), 5.04 (d, *J* = 12.0 Hz, 1H), 4.07 (t, *J* = 7.8 Hz, 1H), 3.91 (br s, 1H), 3.77 (s, 3H), 3.65 (d, *J* = 7.8 Hz, 1H), 2.54 (m, 1H), 2.37–2.29 (overlapped, 3H), 1.25 (d, *J* = 6.2 Hz, 3H); ^13^C NMR (125 MHz, CDCl_3_): *δ* 167.6, 161.2, 156.5, 137.9, 131.4, 131.2, 131.0, 130.5, 129.6, 116.0, 111.5, 111.3, 103.8, 103.7, 103.6, 103.4, 98.8, 76.8, 72.5, 72.4, 72.0, 63.7, 55.4, 38.0, 35.3, 20.5; HRESIMS *m*/*z* 491.1880 [M + H]^+^ (calcd for C_26_H_29_O_7_F_2_, 491.1876); 85% yield.

**Compound 17**: White, amorphous powder; ^1^H NMR (500 MHz, CDCl_3_): *δ* ppm 7.57 (s, 1H), 7.43 (d, *J* = 7.8 Hz, 1H), 7.31 (d, *J* = 7.8 Hz, 1H), 7.22 (t, *J* = 7.8 Hz, 1H), 6.60 (d, *J* = 16.0 Hz, 1H), 6.46 (d, *J* = 2.0 Hz, 1H), 6.38 (d, *J* = 2.0 Hz, 1H), 6.05 (ddd, *J* = 16.0, 8.2, 5.4 Hz, 1H), 5.87 (dt, *J* = 15.8, 6.6 Hz, 1H), 5.61 (dd, *J* = 15.8, 7.7 Hz, 1H), 5.23 (m, 1H), 5.03 (s, 2H), 4.14 (t, *J* = 8.0 Hz, 1H), 3.98 (br s, 1H), 3.79 (s, 3H), 3.69 (d, *J* = 8.0 Hz, 1H), 2.59 (m, 1H), 2.52–2.39 (overlapped, 3H), 1.34 (d, *J* = 6.3 Hz, 3H); ^13^C NMR (125 MHz, CDCl_3_): *δ* 167.3, 161.2, 156.6, 138.7, 137.8, 131.6, 131.1, 130.9, 130.4, 130.0, 129.9, 129.8, 125.4, 122.5, 116.1, 103.4, 99.0, 76.4, 72.1, 72.1, 71.9, 69.5, 55.4, 38.1, 35.5, 20.7; HRESIMS *m*/*z* 555.1000 [M + Na]^+^ (calcd for C_26_H_29_BrO_7_, 555.0994); 84% yield.

**Compound 18**: White, amorphous powder; ^1^H NMR (500 MHz, CDCl_3_): *δ* ppm 7.97 (d, *J* = 8.2 Hz, 2H), 7.42 (d, *J* = 8.2 Hz, 2H), 6.54 (d, *J* = 15.8 Hz, 1H), 6.40 (br s, 1H), 6.33 (br s, 1H), 6.00 (m, 1H), 5.80 (m, 1H), 5.55 (dd, *J* = 15.8, 7.8 Hz, 1H), 5.15 (m, 1H), 5.07 (s, 2H), 4.06 (t, *J* = 7.8 Hz, 1H), 3.90 (br s, 1H), 3.86 (s, 3H), 3.72 (s, 3H), 3.62 (d, *J* = 7.8 Hz, 1H), 2.52 (dt, *J* = 15.7, 7.8 Hz, 1H), 2.40–2.24 (overlapped, 3H), 1.26 (d, *J* = 6.2 Hz, 3H); ^13^C NMR (125 MHz, CDCl_3_): *δ* 167.4, 166.8, 161.2, 156.6, 141.6, 137.8, 131.3, 130.7, 129.7, 129.5, 126.5, 116.2, 115.9, 109.9, 103.3, 98.9, 76.6, 72.3, 72.3, 71.9, 69.7, 55.4, 52.0, 38.0, 35.4, 20.6; HRESIMS *m*/*z* 513.2131 [M + H]^+^ (calcd for C_28_H_33_O_9_, 513.2119); 86% yield.

**Compound 19**: White, amorphous powder; ^1^H NMR (500 MHz, DMSO-*d*_6_): *δ* ppm 7.95 (d, *J* = 8.2 Hz, 2H), 7.53 (d, *J* = 8.2 Hz, 2H), 6.89 (s, 1H), 6.40 (d, *J* = 15.8 Hz, 1H), 5.81 (m, 1H), 5.54 (ddd, *J* = 15.0, 8.2, 6.4 Hz, 1H), 5.41 (dd, *J* = 15.8, 8.6 Hz, 1H), 5.29 (d, *J* = 12.8 Hz, 1H), 5.25 (d, *J* = 12.8 Hz, 1H), 4.76 (m, 1H), 4.66 (d, *J* = 3.8 Hz, 1H), 4.60 (d, *J* = 3.4 Hz, 1H), 4.47 (d, *J* = 6.4 Hz, 1H), 3.87 (s, 3H), 3.83 (s, 3H), 3.75 (td, *J* = 8.8, 3.7 Hz, 1H), 2.25–2.10 (overlapped, 2H), 2.05 (m, 1H), 1.13 (d, *J* = 6.1 Hz, 3H); ^13^C NMR (125 MHz, DMSO-*d*_6_): *δ* 166.4, 166.1, 156.0, 154.6, 142.3, 136.4, 135.7, 134.4, 129.7, 129.6, 129.5, 127.7, 125.8, 117.9, 112.4, 97.9, 79.0, 74.8, 74.6, 71.3, 69.9, 57.0, 52.6, 37.7, 36.4, 20.8; HRESIMS *m*/*z* 547.1746 [M + H]^+^ (calcd for C_28_H_32_O_9_Cl, 547.1729); 88% yield.

**Compound 20**: White, amorphous powder; ^1^H NMR (500 MHz, CDCl_3_): *δ* ppm 7.60 (d, *J* = 8.0 Hz, 2H), 7.51 (d, *J* = 8.0 Hz, 2H), 6.59 (d, *J* = 15.8 Hz, 1H), 6.45 (d, *J* = 1.8 Hz, 1H), 6.37 (d, *J* = 1.8 Hz, 1H), 6.04 (ddd, *J* = 15.8, 8.4, 5.4 Hz, 1H), 5.84 (m, 1H), 5.59 (dd, *J* = 15.8, 7.8 Hz, 1H), 5.20 (m, 1H), 5.14 (s, 2H), 4.11 (t, *J* = 8.3 Hz, 1H), 3.94 (br s, 1H), 3.77 (s, 3H), 3.66 (d, *J* = 7.9 Hz, 1H), 2.56 (dt, *J* = 15.5, 7.7 Hz, 1H), 2.43–2.28 (overlapped, 3H), 1.30 (d, *J* = 6.2 Hz, 3H); ^13^C NMR (125 MHz, CDCl_3_): *δ* 167.4, 161.2, 156.6, 140.5, 138.0, 131.3, 131.2, 130.8, 129.7, 127.0, 125.4, 125.4, 122.9, 116.0, 103.4, 99.0, 76.6, 72.3, 72.3, 72.0, 69.5, 55.4, 38.0, 35.4, 20.6; HRESIMS *m*/*z* 523.1949 [M + H]^+^ (calcd for C_27_H_30_O_7_F_3_, 523.1938); 84% yield.

**Compound 21**: White, amorphous powder; ^1^H NMR (500 MHz, CDCl_3_): *δ* ppm 7.80 (d, *J* = 7.6 Hz, 1H), 7.50 (t, *J* = 7.0 Hz, 1H), 7.44–7.37 (overlapped, 3H), 7.35 (t, *J* = 7.6 Hz, 1H), 7.28 (d, *J* = 8.0 Hz, 2H), 6.56 (d, *J* = 15.8 Hz, 1H), 6.43 (s, 1H), 6.41 (s, 1H), 6.05 (m, 1H), 5.81 (m, 1H), 5.58 (dd, *J* = 15.8, 7.8 Hz, 1H), 5.16 (m, 1H), 5.09 (s, 2H), 4.07 (t, *J* = 8.0 Hz, 1H), 3.91 (br s, 1H), 3.75 (s, 3H), 3.66 (d, *J* = 8.0 Hz, 1H), 3.59 (s, 3H), 2.55 (dt, *J* = 15.0, 7.3 Hz, 1H), 2.39–2.31 (overlapped, 3H), 1.30 (d, *J* = 6.2 Hz, 3H); ^13^C NMR (125 MHz, CDCl_3_): *δ* 169.0, 167.7, 161.2, 156.8, 142.0, 140.8, 137.8, 135.4, 131.2, 130.6, 129.9, 129.7, 129.5, 128.7, 128.7, 128.4, 127.4, 127.2, 126.7, 116.1, 103.3, 99.1, 76.9, 72.6, 72.5, 72.0, 70.2, 55.4, 51.9, 38.1, 35.3, 20.6; HRESIMS *m*/*z* 589.2452 [M + H]^+^ (calcd for C_34_H_37_O_9_, 589.2432); 83% yield.

**Compound 22**: White, amorphous powder; ^1^H NMR (500 MHz, CDCl_3_): *δ* ppm 7.76 (d, *J* = 7.6 Hz, 1H), 7.64 (t, *J* = 7.6 Hz, 1H), 7.58–7.49 (overlapped, 5H), 7.44 (t, *J* = 7.6 Hz, 1H), 6.61 (d, *J* = 16.0 Hz, 1H), 6.46 (s, 1H), 6.44 (s, 1H), 6.05 (ddd, *J* = 15.2, 8.2, 5.6 Hz, 1H), 5.87 (dt, *J* = 15.2, 6.6 Hz, 1H), 5.62 (dd, *J* = 16.0, 7.6 Hz, 1H), 5.23 (m, 1H), 5.13 (s, 2H), 4.12 (m, 1H), 3.97 (br s, 1H), 3.79 (s, 3H), 3.68 (d, *J* = 7.8 Hz, 1H), 2.59 (dt, *J* = 15.6, 7.8 Hz, 1H), 2.44–2.33 (overlapped, 3H), 1.32 (d, *J* = 6.2 Hz, 3H); ^13^C NMR (125 MHz, CDCl_3_): *δ* 167.4, 161.2, 156.8, 145.0, 137.8, 137.6, 137.1, 133.7, 132.8, 131.5, 131.2, 130.4, 130.0, 129.9, 128.8, 127.6, 127.3, 118.6, 116.0, 111.2, 103.4, 99.0, 76.4, 72.1, 71.9, 70.0, 60.4, 55.4, 38.1, 35.5, 20.7; HRESIMS *m*/*z* 556.2346 [M + H]^+^ (calcd for C_33_H_34_O_7_N, 556.2330); 87% yield.

**Compound 23**: White, amorphous powder; ^1^H NMR (500 MHz, CDCl_3_, DMSO-*d*_6_): *δ* ppm 7.75 (d, *J* = 7.6 Hz, 1H), 7.66 (t, *J* = 7.6 Hz, 1H), 7.53–7.49 (overlapped, 5H), 7.46 (t, *J* = 7.6 Hz, 1H), 6.67 (s, 1H), 6.35 (d, *J* = 16.2 Hz, 1H), 5.87 (m, 1H), 5.59 (ddd, *J* = 15.2, 9.5, 5.4 Hz, 1H), 5.41 (m, 1H), 5.18 (s, 2H), 4.82 (m, 1H), 4.46–4.32 (overlapped, 3H), 3.85 (s, 3H), 3.79 (t, *J* = 8.8 Hz, 1H), 3.45–3.43 (overlapped, 2H), 2.53 (m, 1H), 2.25–2.08 (overlapped, 3H), 1.16 (d, *J* = 6.1 Hz, 3H); ^13^C NMR (125 MHz, CDCl_3_, DMSO-*d*_6_): *δ* 170.9, 160.7, 159.4, 149.3, 142.6, 141.8, 140.7, 140.5, 138.5, 138.0, 137.9, 135.2, 134.9, 133.6, 132.8, 132.4, 130.5, 123.2, 122.7, 117.8, 115.6, 102.0, 83.6, 79.3, 79.0, 76.0, 75.1, 61.3, 42.8, 41.4, 25.6; HRESIMS *m*/*z* 588.1801 [M – H]^–^ (calcd for C_33_H_31_O_7_NCl_2_, 588.1784); 89% yield.

**Compound 24**: White, amorphous powder; ^1^H NMR (500 MHz, CDCl_3_): *δ* ppm 7.40 (t, *J* = 8.0 Hz, 1H), 7.29 (d, *J* = 8.0 Hz, 1H), 7.25 (d, *J* = 8.0 Hz, 1H), 6.61 (d, *J* = 15.8 Hz, 1H), 6.46 (s, 1H), 6.41 (s, 1H), 6.04 (m, 1H), 587 (m, 1H), 5.61 (dd, *J* = 15.8, 7.8 Hz, 1H), 5.23 (m, 1H), 5.10 (d, *J* = 12.6 Hz, 1H), 5.06 (d, *J* = 12.6 Hz, 1H), 4.15 (t, *J* = 7.8 Hz, 1H), 3.98 (br s, 1H), 3.80 (s, 3H), 3.67 (d, *J* = 7.8 Hz, 1H), 3.06 (s, 1H), 2.64–2.54 (overlapped, 2H), 2.50 (s, 1H), 2.44–2.36 (overlapped, 3H), 1.31 (d, *J* = 6.2 Hz, 3H); ^13^C NMR (125 MHz, CDCl_3_): *δ* ppm 167.1, 161.3, 156.5, 137.9, 131.5, 131.2, 130.4, 130.4, 130.3, 129.9, 127.6, 127.6, 118.9, 118.7, 115.9, 103.7, 98.8, 76.3, 72.0, 71.9, 63.7, 63.7, 55.4, 38.1, 35.5, 20.6; HRESIMS *m*/*z* 551.1090 [M + H]^+^ (calcd for C_26_H_29_O_7_BrF, 551.1075); 84% yield.

**Compound 25**: White, amorphous powder; ^1^H NMR (500 MHz, CDCl_3_, DMSO-*d*_6_): *δ* ppm 7.46–7.28 (overlapped, 3H), 6.80 (s, 1H), 6.35 (d, *J* = 15.8 Hz, 1H), 5.83 (m, 1H), 5.55 (m, 1H), 5.40 (dd, *J* = 15.8, 8.7 Hz, 1H), 5.14 (s, 2H), 4.78 (m, 1H), 4.52 (d, *J* = 3.1 Hz, 1H), 4.46 (d, *J* = 2.1 Hz, 1H), 4.39 (d, *J* = 6.4 Hz, 1H), 3.89 (s, 3H), 3.75 (m, 1H), 3.42–3.37 (overlapped, 2H), 2.12–2.10 (overlapped, 4H), 1.08 (d, *J* = 6.4 Hz, 3H); ^13^C NMR (125 MHz, CDCl_3_, DMSO-*d*_6_): *δ* 165.9, 156.0, 154.4, 136.2, 135.8, 133.7, 131.8, 130.0, 127.7, 125.7, 123.2, 122.2, 119.1, 118.9, 117.9, 113.0, 97.4, 78.5, 74.6, 74.3, 71.2, 64.4, 56.7, 37.9, 36.6, 20.6; HRESIMS *m*/*z* 585.0694 [M + H]^+^ (calcd for C_26_H_28_O_7_BrClF, 585.0685); 86% yield.

**Compound 26**: White, amorphous powder; ^1^H NMR (500 MHz, CDCl_3_): *δ* ppm 7.35 (d, *J* = 8.4 Hz, 2H), 7.30 (d, *J* = 8.4 Hz, 2H), 6.54 (d, *J* = 15.8 Hz, 1H), 6.41 (d, *J* = 1.8 Hz, 1H), 6.39 (d, *J* = 1.8 Hz, 1H), 6.03 (ddd, *J* = 15.8, 8.2, 5.4 Hz, 1H), 5.80 (m, 1H), 5.57 (dd, *J* = 15.8, 7.8 Hz, 1H), 5.14 (m, 1H), 5.02 (d, *J* = 12.8 Hz, 1H), 5.00 (d, *J* = 12.8 Hz, 1H), 4.06 (t, *J* = 7.8 Hz, 1H), 3.89 (br s, 1H), 3.74 (s, 3H), 3.65 (d, *J* = 7.8 Hz, 1H), 2.54 (dt, *J* = 15.0, 7.4 Hz, 1H), 2.37–2.31 (overlapped, 3H), 1.30 (s, 9H), 1.25 (d, *J* = 6.2 Hz, 3H); ^13^C NMR (125 MHz, CDCl_3_): *δ* ppm 167.8, 161.1, 157.0, 150.8, 137.7, 133.5, 131.4, 131.1, 131.1, 129.5, 126.8, 125.3, 116.1, 103.2, 99.0, 72.6, 72.6, 72.0, 70.3, 60.4, 55.4, 38.0, 35.3, 34.5, 31.3, 20.5; HRESIMS *m*/*z* 511.2703 [M + H]^+^ (calcd for C_30_H_39_O_7_, 511.2690); 83% yield.

**Compound 27**: White, amorphous powder; ^1^H NMR (500 MHz, CDCl_3_): *δ* ppm 7.26 (t, *J* = 7.4 Hz, 2H), 7.19–7.14 (overlapped, 3H), 6.53 (d, *J* = 15.6 Hz, 1H), 6.41 (s, 1H), 6.29 (s, 1H), 6.07 (m, 1H), 5.79 (m, 1H), 5.57 (dd, *J* = 15.6, 7.8 Hz, 1H), 5.11 (m, 1H), 4.61 (s, 1H), 4.22 (s, 1H), 4.16 (s, 1H), 4.03 (t, *J* = 8.0 Hz, 1H), 3.93–3.89 (overlapped, 3H), 3.74 (s, 3H), 3.68 (d, *J* = 8.0 Hz, 1H), 2.75 (t, *J* = 7.5 Hz, 2H), 2.56 (m, 1H), 2.38–2.31 (overlapped, 3H), 2.09–1.99 (overlapped, 2H), 1.36 (d, *J* = 6.4 Hz, 3H); ^13^C NMR (125 MHz, CDCl_3_): *δ* ppm 168.0, 161.1, 157.1, 141.3, 137.6, 131.5, 131.4, 131.2, 129.2, 128.5, 128.4, 125.9, 115.9, 102.8, 98.2, 73.0, 73.0, 72.9, 71.9, 67.3, 55.3, 38.0, 35.2, 31.9, 30.6, 20.7; HRESIMS *m*/*z* 483.2383 [M + H]^+^ (calcd for C_28_H_35_O_7_, 483.2377); 87% yield.

**Compound 28**: Colorless oil; ^1^H NMR (500 MHz, CDCl_3_): *δ* ppm 11.50 (s, 1H), 7.16 (dd, *J* = 15.2, 1.9 Hz, 1H), 6.47 (d, *J* = 2.5 Hz, 1H), 6.40 (d, *J* = 2.5 Hz, 1H), 6.00 (m, 1H), 5.73 (ddd, *J* = 15.2, 10.6, 3.0 Hz, 1H), 5.53 (dd, *J* = 15.2, 8.8 Hz, 1H), 5.46 (m, 1H), 4.57 (t, *J* = 8.0 Hz, 1H), 4.21 (m, 1H), 3.90 (dd, *J* = 8.0, 2.1 Hz, 1H), 3.82 (s, 3H), 2.76 (m, 1H), 2.50 (dd, *J* = 8.1, 3.4 Hz, 1H), 2.44 (dt, *J* = 10.5, 4.5 Hz, 1H), 2.29 (m, 1H), 1.45 (d, *J* = 6.8 Hz, 3H), 1.44 (s, 3H), 1.37 (s, 3H); ^13^C NMR (125 MHz, CDCl_3_): *δ* 170.7, 164.7, 163.9, 142.0, 134.0, 132.6, 129.5, 126.3, 108.4, 107.1, 104.3, 100.0, 81.3, 75.2, 70.5, 68.7, 55.4, 37.8, 35.9, 26.9, 26.9, 19.1; HRESIMS *m*/*z* 403.1765 [M – H]^–^ (calcd for C_22_H_27_O_7_, 403.1751); 95% yield.

**Compound 29**: Colorless oil; ^1^H NMR (500 MHz, CDCl_3_): *δ* ppm 11.36 (s, 1H), 6.63 (dd, *J* = 16.0, 1.2 Hz, 1H), 6.46 (s, 1H), 5.98 (ddd, *J* = 15.4, 6.9, 5.0 Hz, 1H), 5.51 (ddd, *J* = 16.0, 9.0, 3.8 Hz, 1H), 5.44 (dd, *J* = 15.4, 8.8 Hz, 1H), 5.39 (m, 1H), 4.54 (t, *J* = 8.2 Hz, 1H), 4.19 (ddd, *J* = 12.2, 4.8, 2.2 Hz, 1H), 3.90 (s, 3H), 3.80 (dd, *J* = 8.2, 2.2 Hz, 1H), 2.86 (m, 1H), 2.55 (m, 1H), 2.42 (m, 1H), 2.35 (m, 1H), 1.43 (s, 3H), 1.38 (d, *J* = 6.6 Hz, 3H), 1.38 (s, 3H); ^13^C NMR (125 MHz, CDCl_3_): *δ* 170.4, 162.2, 159.8, 139.7, 130.8, 130.6, 130.3, 128.8, 113.9, 108.5, 106.2, 99.3, 80.9, 75.4, 71.2, 68.2, 56.4, 37.0, 34.7, 27.0, 26.9, 19.3; HRESIMS *m*/*z* 437.1376 [M – H]^–^ (calcd for C_22_H_26_O_7_ Cl, 437.1362); 94% yield.

**Compound 30**: Colorless oil; EIMS *m/z*: 461.38 [M + H]^+^; ^1^H NMR (500 MHz, CDCl_3_): *δ* ppm 7.03 (dd, *J* = 15.6, 1.4 Hz, 1H), 6.76 (d, *J* = 2.4 Hz, 1H), 6.53 (d, *J* = 2.4 Hz, 1H), 5.98 (dt, *J* = 15.6, 5.1 Hz, 1H), 5.80 (m, 1H), 5.48 (dd, *J* = 15.6, 8.9 Hz, 1H), 5.41 (m, 1H), 4.54 (t, *J* = 8.0 Hz, 1H), 4.14 (m, 1H), 3.86 (dd, *J* = 8.0, 2.1 Hz, 1H), 3.80 (s, 3H), 2.65 (m, 1H), 2.58 (q, *J* = 7.6 Hz, 2H), 2.50–2.46 (overlapped, 2H), 2.27 (dd, *J* = 25.2, 12.0 Hz, 1H), 1.42 (s, 3H), 1.36 (d, *J* = 6.4 Hz, 3H), 1.35 (s, 3H), 1.24 (t, *J* = 7.6 Hz, 3H); ^13^C NMR (125 MHz, CDCl_3_): *δ* 172.7, 165.0, 161.5, 151.2, 139.7, 132.7, 131.2, 130.3, 128.0, 116.9, 110.1, 108.4, 107.7, 81.0, 75.7, 69.3, 68.4, 55.5, 37.0, 36.0, 27.5, 26.9, 26.9, 19.9, 8.8; HRESIMS *m*/*z* 461.2172 [M + H]^+^ (calcd for C_25_H_33_O_8_, 461.2170); 46% yield.

**Compound 31**: Colorless oil; EIMS *m/z*: 495.87 [M + H]^+^; ^1^H NMR (500 MHz, CDCl_3_): *δ* ppm 6.60 (s, 1H), 6.49 (dd, *J* = 16.0, 1.6 Hz, 1H), 5.94 (dt, *J* = 16.0, 4.5 Hz, 1H), 5.77 (ddd, *J* = 16.0, 10.1, 3.7 Hz, 1H), 5.61–5.51 (overlapped, 2H), 4.50 (t, *J* = 8.4 Hz, 1H), 4.12 (m, 1H), 3.88 (s, 3H), 3.78 (d, *J* = 8.4 Hz, 1H), 2.76 (m, 1H), 2.57 (q, *J* = 7.4 Hz, 2H), 2.44–2.39 (overlapped, 2H), 2.29 (m, 1H), 1.41 (s, 3H), 1.37 (s, 3H), 1.29 (d, *J* = 6.4 Hz, 3H), 1.24 (t, *J* = 7.4 Hz, 3H). ^13^C NMR (125 MHz, CDCl_3_): *δ* 172.4, 165.0, 156.9, 147.7, 136.7, 133.2, 131.4, 128.8, 127.1, 119.7, 119.2, 108.5, 105.4, 81.1, 75.4, 69.0, 67.9, 56.5, 37.0, 35.6, 27.4, 26.9, 26.9, 20.7, 8.7; 48% yield.

**Compound 32**: Colorless oil; ^1^H NMR (500 MHz, CDCl_3_): *δ* ppm 7.03 (d, *J* = 15.6 Hz, 1H), 6.74 (d, *J* = 2.2 Hz, 1H), 6.49 (d, *J* = 2.2 Hz, 1H), 5.98 (dt, *J* = 15.6, 4.8 Hz, 1H), 5.79 (m, 1H), 5.54–5.41 (overlapped, 2H), 4.53 (t, *J* = 8.2 Hz, 1H), 4.12 (m, 1H), 3.84 (dd, *J* = 8.2, 1.9 Hz, 1H), 3.80 (s, 3H), 2.62 (m, 1H), 2.53–2.48 (overlapped, 2H), 2.28 (dd, *J* = 24.9, 12.1 Hz, 1H), 1.41 (s, 3H), 1.37–1.33 (overlapped, 15H); ^13^C NMR (125 MHz, CDCl_3_): *δ* 176.4, 165.0, 161.4, 151.1, 139.4, 132.7, 131.5, 129.7, 128.2, 117.4, 110.0, 108.3, 107.4, 81.0, 75.8, 68.9, 68.3, 55.5, 38.9, 37.0, 35.9, 27.0, 26.9, 26.9, 20.3; HRESIMS *m*/*z* 489.2485 [M + H]^+^ (calcd for C_27_H_37_O_8_, 489.2485); 42% yield.

**Compound 33**: Colorless oil; ^1^H NMR (500 MHz, CDCl_3_): *δ* ppm 6.55 (s, 1H), 6.47 (dd, *J* = 16.0, 1.9 Hz, 1H), 5.95 (dt, *J* = 16.0, 4.3 Hz, 1H), 5.76 (ddd, *J* = 16.0, 10.3, 3.6 Hz, 1H), 5.63 (m, 1H), 5.55 (dd, *J* = 16.0, 8.6 Hz, 1H), 4.51 (t, *J* = 8.4 Hz, 1H), 4.11 (m, 1H), 3.89 (s, 3H), 3.77 (dd, *J* = 8.4, 2.1 Hz, 1H), 2.77 (m, 1H), 2.50–2.41 (overlapped, 2H), 2.29 (dd, *J* = 25.3, 11.9 Hz, 1H), 1.41 (s, 3H), 1.37 (s, 3H), 1.34 (s, 9H), 1.29 (d, *J* = 6.4 Hz, 3H); ^13^C NMR (125 MHz, CDCl_3_): *δ* 176.2, 164.7, 156.8, 147.8, 136.7, 133.4, 131.4, 128.6, 127.0, 119.9, 119.0, 108.5, 105.2, 81.1, 75.5, 68.6, 67.9, 56.5, 39.0, 37.1, 35.7, 27.0, 27.0, 26.9, 20.9; 45% yield;

**Compound 34**: Colorless oil; ^1^H NMR (500 MHz, CDCl_3_): *δ* ppm 7.05 (d, *J* = 15.6 Hz, 1H), 6.80 (d, *J* = 2.3 Hz, 1H), 6.57 (d, *J* = 2.3 Hz, 1H), 5.98 (dt, *J* = 15.6, 5.3 Hz, 1H), 5.81 (m, 1H), 5.48 (dd, *J* = 15.6, 8.9 Hz, 1H), 5.39 (m, 1H), 4.55 (t, *J* = 8.5 Hz, 1H), 4.30 (d, *J* = 16.8 Hz, 1H), 4.26 (d, *J* = 16.8 Hz, 1H), 4.16 (m, 1H), 3.87 (dd, *J* = 8.0, 1.9 Hz, 1H), 3.82 (s, 3H), 3.54 (s, 3H), 2.68 (dd, *J* = 14.0, 1.9 Hz, 1H), 2.58–2.43 (overlapped, 2H), 2.28 (dd, *J* = 25.2, 12.0 Hz, 1H), 1.43 (s, 3H), 1.38 (d, *J* = 6.3 Hz, 3H), 1.36 (s, 3H); ^13^C NMR (125 MHz, CDCl_3_): *δ* 168.9, 164.8, 161.6, 150.8, 140.1, 132.7, 131.0, 130.6, 128.1, 116.4, 110.4, 108.4, 107.8, 81.0, 75.7, 69.7, 69.6, 68.4, 59.6, 55.6, 36.9, 36.0, 26.9, 26.9, 19.8; HRESIMS *m*/*z* 477.2125 [M + H]^+^ (calcd for C_25_H_33_O_9_, 477.2119); 46% yield.

**Compound 35**: Colorless oil; ^1^H NMR (500 MHz, CDCl_3_): *δ* ppm 6.63 (s, 1H), 6.50 (dd, *J* = 16.0, 1.9 Hz, 1H), 5.95 (dt, *J* = 16.0, 4.6 Hz, 1H), 5.76 (ddd, *J* = 16.0, 10.1, 3.7 Hz, 1H), 5.60–5.47 (overlapped, 2H), 4.51 (t, *J* = 8.4 Hz, 1H), 4.29 (d, *J* = 16.8 Hz, 1H), 4.24 (d, *J* = 16.8 Hz, 1H), 4.15 (ddd, *J* = 12.0, 4.2, 2.3 Hz, 1H), 4.06 (s, 2H), 3.89 (s, 3H), 3.79 (dd, *J* = 8.4, 2.2 Hz, 1H), 3.53 (s, 3H), 3.46 (s, 3H), 2.77 (ddd, *J* = 14.6, 6.5, 3.8 Hz, 1H), 2.51–2.39 (overlapped, 2H), 2.31 (m, 1H), 1.41 (s, 3H), 1.37 (s, 3H), 1.30 (d, *J* = 6.4 Hz, 3H); ^13^C NMR (125 MHz, CDCl_3_): *δ* 168.6, 164.8, 157.1, 147.3, 137.1, 133.3, 131.4, 128.7, 127.1, 119.7, 119.3, 108.6, 105.3, 81.1, 75.5, 69.5, 69.3, 69.2, 67.9, 59.6, 59.3, 56.6, 36.9, 35.5, 29.6, 26.9, 26.8, 20.7; HRESIMS *m*/*z* 581.0936 [M – H]^–^ (calcd for C_28_H_34_O_11_Cl, 581.1784); 47% yield.

**Compound 36**: Colorless oil; ^1^H NMR (500 MHz, CDCl_3_): *δ* ppm 11.49 (s, 1H), 7.39–7.27 (overlapped, 5H), 7.16 (dd, *J* = 15.4, 1.8 Hz, 1H), 6.47 (d, *J* = 2.5 Hz, 1H), 6.40 (d, *J* = 2.5 Hz, 1H), 6.00 (m, 1H), 5.73 (ddd, *J* = 15.2, 10.4, 3.0 Hz, 1H), 5.52 (dd, *J* = 15.2, 8.8 Hz, 1H), 5.45 (m, 1H), 4.63 (s, 2H), 4.57 (t, *J* = 8.0 Hz, 1H), 4.22 (ddd, *J* = 12.2, 4.7, 2.0 Hz, 1H), 4.11 (s, 2H), 3.90 (dd, *J* = 8.0, 2.0 Hz, 1H), 3.81 (s, 3H), 2.76 (m, 1H), 2.51 (ddd, *J* = 15.2, 8.1, 3.1 Hz, 1H), 2.43 (m, 1H), 2.30 (dt, *J* = 14.6, 11.7 Hz, 1H), 1.46–1.42 (overlapped, 6H), 1.37 (s, 3H); ^13^C NMR (125 MHz, CDCl_3_): *δ* 173.5, 170.7, 164.7, 163.9, 142.0, 136.5, 134.0, 132.6, 129.5, 128.5, 128.2, 128.0, 126.3, 108.5, 107.1, 104.3, 100.0, 81.3, 75.2, 73.4, 70.5, 68.7, 66.8, 55.4, 37.8, 35.9, 26.9, 26.9, 19.1; HRESIMS *m*/*z* 553.2416 [M + H]^+^ (calcd for C_31_H_37_O_9_, 553.2432); 43% yield.

**Compound 37**: Colorless oil; ^1^H NMR (500 MHz, CDCl_3_): *δ* ppm 7.30 (d, *J* = 8.0 Hz, 2H), 7.16 (d, *J* = 8.0 Hz, 2H), 6.83 (d, *J* = 15.8 Hz, 1H), 6.47 (d, *J* = 2.0 Hz, 1H), 6.42 (d, *J* = 2.0 Hz, 1H), 5.99 (dt, *J* = 15.8, 5.0 Hz, 1H), 5.88 (m, 1H), 5.65 (dd, *J* = 15.8, 8.6 Hz, 1H), 5.59 (m, 1H), 5.06 (d, *J* = 12.0 Hz, 1H), 5.03 (d, *J* = 12.0 Hz, 1H), 4.55 (t, *J* = 8.0 Hz, 1H), 4.12 (m, 1H), 3.95 (dd, *J* = 8.0, 2.0 Hz, 1H), 3.78 (s, 3H), 2.62 (m, 1H), 2.54–2.48 (overlapped, 2H), 2.33 (s, 3H), 2.28 (dd, *J* = 24.2, 12.3 Hz, 1H), 1.43 (s, 3H), 1.38 (s, 3H), 1.36 (d, *J* = 6.4 Hz, 3H); ^13^C NMR (125 MHz, CDCl_3_): *δ* 166.4, 161.4, 157.6, 138.1, 137.4, 133.6, 132.6, 131.4, 130.0, 129.1, 127.4, 126.9, 115.6, 108.3, 102.8, 99.5, 81.2, 75.6, 70.4, 68.9, 68.4, 55.4, 37.6, 35.7, 26.9, 26.8, 21.1, 20.6; HRESIMS *m*/*z* 509.2527 [M + H]^+^ (calcd for C_30_H_37_O_7_, 509.2534); 86% yield.

**Compound 38**: Colorless oil; ^1^H NMR (500 MHz, CDCl_3_): *δ* ppm 7.75 (d, *J* = 7.6 Hz, 1H), 7.63 (m, 1H), 7.59–7.53 (overlapped, 4H), 7.50 (d, *J* = 7.8 Hz, 1H), 7.43 (t, *J* = 7.6 Hz, 1H), 6.86 (d, *J* = 15.8 Hz, 1H), 6.50 (d, *J* = 1.8 Hz, 1H), 6.45 (d, *J* = 1.8 Hz, 1H), 6.00 (dt, *J* = 15.8, 5.0 Hz, 1H), 5.90 (m, 1H), 5.71–5.59 (overlapped, 2H), 5.17 (d, *J* = 12.5 Hz, 1H), 5.14 (d, *J* = 12.5 Hz, 1H), 4.56 (t, *J* = 8.0 Hz, 1H), 4.13 (m, 1H), 3.96 (dd, *J* = 8.0, 1.9 Hz, 1H), 3.80 (s, 3H), 2.62 (m, 1H), 2.57–2.47 (overlapped, 2H), 2.29 (dd, *J* = 24.3, 12.2 Hz, 1H), 1.43 (s, 3H), 1.39 (d, *J* = 6.7 Hz, 3H), 1.38 (s, 3H); ^13^C NMR (125 MHz, CDCl_3_): *δ* 166.3, 161.5, 157.5, 145.0, 138.3, 137.5, 137.3, 133.7, 132.8, 132.5, 131.4, 130.0, 130.0, 128.9, 127.6, 127.5, 127.0, 118.6, 115.6, 111.1, 108.3, 103.0, 99.4, 81.2, 75.6, 70.1, 69.0, 68.4, 55.4, 37.5, 35.8, 26.9, 26.8, 20.7; HRESIMS *m*/*z* 596.2665 [M + H]^+^ (calcd for C_36_H_38_O_7_N, 596.2643); 82% yield.

**Compound 39**: Colorless oil; ^1^H NMR (500 MHz, CDCl_3_): *δ* ppm 7.81 (dd, *J* = 7.6, 0.8 Hz, 1H), 7.52 (td, *J* = 7.6, 1.2 Hz, 1H), 7.45 (d, *J* = 8.0 Hz, 2H), 7.40 (td, *J* = 7.6, 1.2 Hz, 1H), 7.36 (d, *J* = 7.6 Hz, 1H), 7.31 (d, *J* = 8.0 Hz, 2H), 6.85 (d, *J* = 16.2 Hz, 1H), 6.49 (d, *J* = 2.0 Hz, 1H), 6.45 (d, *J* = 2.0 Hz, 1H), 6.00 (dt, *J* = 15.8, 5.0 Hz, 1H), 5.89 (m, 1H), 5.65 (dd, *J* = 15.8, 8.6 Hz, 1H), 5.61 (m, 1H), 5.16 (d, *J* = 12.2 Hz, 1H), 5.13 (d, *J* = 12.2 Hz, 1H), 4.56 (t, *J* = 8.0 Hz, 1H), 4.11 (d, *J* = 8.0 Hz, 1H), 3.95 (dd, *J* = 8.0, 2.2 Hz, 1H), 3.79 (s, 3H), 3.60 (s, 3H), 2.67–2.47 (overlapped, 4H), 1.43 (s, 3H), 1.38 (s, 3H), 1.38 (d, *J* = 6.1 Hz, 3H); ^13^C NMR (125 MHz, CDCl_3_): *δ* 169.0, 166.4, 161.5, 157.6, 142.0, 140.8, 138.2, 135.6, 132.5, 131.4, 131.2, 130.8, 130.6, 130.0, 129.7, 128.4, 127.5, 127.2, 126.5, 115.7, 108.3, 102.8, 99.6, 81.2, 75.6, 70.3, 68.9, 68.4, 55.4, 51.8, 37.5, 35.7, 26.9, 26.8, 20.7; HRESIMS *m*/*z* 629.2759 [M + H]^+^ (calcd for C_37_H_41_O_9_, 629.2745); 87% yield.

**Compound 40**: Colorless oil; ^1^H NMR (500 MHz, CDCl_3_): *δ* ppm 7.32 (m, 1H), 7.18 (d, *J* = 7.8 Hz, 1H), 7.15 (d, *J* = 9.5 Hz, 1H), 6.98 (td, *J* = 8.5, 2.0 Hz, 1H), 6.85 (d, *J* = 16.0 Hz, 1H), 6.49 (d, *J* = 2.0 Hz, 1H), 6.39 (d, *J* = 2.0 Hz, 1H), 6.00 (dt, *J* = 16.0, 5.0 Hz, 1H), 5.89 (m, 1H), 5.70–5.55 (overlapped, 2H), 5.10 (d, *J* = 12.6 Hz, 1H), 5.07 (d, *J* = 12.6 Hz, 1H), 4.56 (t, *J* = 8.0 Hz, 1H), 4.12 (m, 1H), 3.95 (dd, *J* = 8.0, 2.1 Hz, 1H), 3.79 (s, 3H), 2.63 (m, 1H), 2.55–2.51 (overlapped, 2H), 2.29 (dd, *J* = 24.2, 12.2 Hz, 1H), 1.44 (s, 3H), 1.39 (d, *J* = 6.4 Hz, 3H), 1.38 (s, 3H); ^13^C NMR (125 MHz, CDCl_3_): *δ* 166.3, 163.9, 161.5, 157.3, 139.2, 138.3, 132.5, 131.4, 130.0, 130.0, 127.6, 122.1, 115.6, 114.7, 113.7, 108.3, 103.0, 99.5, 81.1, 75.6, 69.7, 69.0, 68.4, 55.4, 37.5, 35.7, 26.9, 26.8, 20.6; HRESIMS *m*/*z* 513.2288 [M + H]^+^ (calcd for C_29_H_34_O_7_F, 513.2283); 85% yield.

**Compound 41**: Colorless oil; ^1^H NMR (500 MHz, CDCl_3_): *δ* ppm 7.46 (t, *J* = 8.0 Hz, 1H), 7.31 (d, *J* = 8.3 Hz, 1H), 7.24 (dd, *J* = 9.5, 1.5 Hz, 1H), 6.85 (d, *J* = 15.8 Hz, 1H), 6.50 (d, *J* = 1.9 Hz, 1H), 6.43 (d, *J* = 1.9 Hz, 1H), 6.00 (dt, *J* = 15.8, 5.0 Hz, 1H), 5.88 (m, 1H), 5.66–5.55 (overlapped, 2H), 5.09 (s, 2H), 4.55 (t, *J* = 8.2 Hz, 1H), 4.12 (m, 1H), 3.93 (dd, *J* = 8.0, 2.0 Hz, 1H), 3.80 (s, 3H), 2.63 (m, 1H), 2.54–2.46 (overlapped, 2H), 2.28 (dd, *J* = 24.2, 12.3 Hz, 1H), 1.43 (s, 3H), 1.38 (s, 3H), 1.37 (d, *J* = 6.6 Hz, 3H); ^13^C NMR (125 MHz, CDCl_3_): *δ* 166.2, 161.6, 160.6, 158.6, 157.1, 138.5, 132.5, 131.3, 130.2, 130.0, 127.7, 123.2, 121.8, 118.6, 115.4, 108.3, 103.3, 99.2, 81.1, 75.6, 69.0, 68.4, 63.7, 55.4, 37.5, 35.7, 26.9, 26.8, 20.6; HRESIMS *m*/*z* 591.1400 [M + H]^+^ (calcd for C_29_H_33_O_7_BrF, 591.1388); 82% yield.

**Compound 42**: Colorless oil; ^1^H NMR (500 MHz, CDCl_3_): *δ* ppm 7.35 (d, *J* = 8.4 Hz, 2H), 7.32 (d, *J* = 8.4 Hz, 2H), 6.84 (d, *J* = 15.8 Hz, 1H), 6.48 (d, *J* = 1.6 Hz, 1H), 6.39 (d, *J* = 1.6 Hz, 1H), 5.99 (dt, *J* = 15.8, 4.9 Hz, 1H), 5.88 (m, 1H), 5.63 (dd, *J* = 16.0, 8.6 Hz, 1H), 5.59 (m, 1H), 5.06 (d, *J* = 12.6 Hz, 1H), 5.03 (d, *J* = 12.6 Hz, 1H), 4.55 (t, *J* = 8.0 Hz, 1H), 4.12 (m, 1H), 3.94 (dd, *J* = 8.0, 1.5 Hz, 1H), 3.78 (s, 3H), 2.62 (m, 1H), 2.54–2.48 (overlapped, 2H), 2.28 (dd, *J* = 24.3, 12.2 Hz, 1H), 1.43 (s, 3H), 1.38 (s, 3H), 1.36 (d, *J* = 6.4 Hz, 3H); ^13^C NMR (125 MHz, CDCl_3_): *δ* 166.3, 161.5, 157.4, 138.3, 135.1, 133.5, 132.5, 131.3, 130.0, 128.6, 128.1, 127.6, 115.5, 108.3, 102.9, 99.5, 81.1, 75.6, 69.7, 69.0, 68.4, 55.4, 37.5, 35.7, 26.9, 26.8, 20.6; HRESIMS *m*/*z* 529.1998 [M + H]^+^ (calcd for C_29_H_34_O_7_Cl, 529.1988); 84% yield.

**Compound 43**: Colorless oil; ^1^H NMR (500 MHz, CDCl_3_): *δ* ppm 7.57 (s, 1H), 7.42 (d, *J* = 7.8 Hz, 1H), 7.35 (d, *J* = 7.8 Hz, 1H), 7.23 (t, *J* = 7.8 Hz, 1H), 6.84 (d, *J* = 15.8 Hz, 1H), 6.49 (d, *J* = 2.0 Hz, 1H), 6.39 (d, *J* = 2.0 Hz, 1H), 6.00 (dt, *J* = 15.8, 5.0 Hz, 1H), 5.89 (ddd, *J* = 15.3, 10.0, 4.4 Hz, 1H), 5.64–5.60 (overlapped, 2H), 5.06 (d, *J* = 13.0 Hz, 1H), 5.03 (d, *J* = 13.0 Hz, 1H), 4.55 (t, *J* = 8.0 Hz, 1H), 4.12 (dd, *J* = 8.0, 3.3 Hz, 1H), 3.94 (dd, *J* = 8.0, 2.1 Hz, 1H), 3.79 (s, 3H), 2.62 (m, 1H), 2.57–2.47 (overlapped, 2H), 2.29 (dd, *J* = 24.2, 12.3 Hz, 1H), 1.43 (s, 3H), 1.40 (d, *J* = 6.4 Hz, 3H), 1.38 (s, 3H); ^13^C NMR (125 MHz, CDCl_3_): *δ* 166.3, 161.5, 157.3, 138.9, 138.3, 132.5, 131.4, 130.8, 130.0, 129.9, 129.7, 127.7, 125.2, 122.5, 115.7, 108.3, 103.0, 99.5, 81.2, 75.5, 69.6, 69.0, 68.4, 55.4, 37.5, 35.7, 26.9, 26.8, 20.7; HRESIMS *m*/*z* 571.1344 [M – H]^–^ (calcd for C_29_H_32_O_7_Br, 571.1326); 86% yield.

**Compound 44**: Colorless oil; ^1^H NMR (500 MHz, CDCl_3_): *δ* ppm 6.96 (d, *J* = 5.9 Hz, 2H), 6.86 (dd, *J* = 15.8, 1.0 Hz, 1H), 6.72 (m, 1H), 6.51 (d, *J* = 2.1 Hz, 1H), 6.36 (d, *J* = 2.1 Hz, 1H), 6.00 (dt, *J* = 15.8, 5.0 Hz, 1H), 5.90 (ddd, *J* = 15.4, 10.5, 4.4 Hz, 1H), 5.69–5.58 (overlapped, 2H), 5.07 (d, *J* = 14.2 Hz, 1H), 5.04 (d, *J* = 14.2 Hz, 1H), 4.56 (t, *J* = 8.0 Hz, 1H), 4.11 (m, 1H), 3.94 (dd, *J* = 8.0, 2.2 Hz, 1H), 3.80 (s, 3H), 2.63 (m, 1H), 2.57–2.51 (overlapped, 2H), 2.30 (dd, *J* = 24.2, 13.2 Hz, 1H), 1.44 (s, 3H), 1.40 (d, *J* = 6.4 Hz, 3H), 1.38 (s, 3H); ^13^C NMR (125 MHz, CDCl_3_): *δ* 166.2, 164.0, 162.1, 161.5, 157.1, 140.7, 138.5, 132.4, 131.4, 130.0, 127.8, 115.6, 109.4, 108.4, 103.1, 99.5, 81.1, 75.6, 69.2, 69.1, 68.4, 55.4, 37.5, 35.7, 26.9, 26.8, 20.6; HRESIMS *m*/*z* 531.2197 [M + H]^+^ (calcd for C_29_H_33_O_7_F_2_, 531.2189); 83% yield.

**Compound 45**: Colorless oil; ^1^H NMR (500 MHz, CDCl_3_): *δ* ppm 7.62 (d, *J* = 8.2 Hz, 2H), 7.54 (d, *J* = 8.2 Hz, 2H), 6.86 (d, *J* = 16.4 Hz, 1H), 6.50 (d, *J* = 2.0 Hz, 1H), 6.39 (d, *J* = 2.0 Hz, 1H), 6.00 (dt, *J* = 15.8, 5.0 Hz, 1H), 5.89 (ddd, *J* = 15.8, 10.5, 4.4 Hz, 1H), 5.70–5.54 (overlapped, 2H), 5.15 (d, *J* = 13.6 Hz, 1H), 5.12 (d, *J* = 13.6 Hz, 1H), 4.55 (t, *J* = 8.0 Hz, 1H), 4.11 (m, 1H), 3.94 (dd, *J* = 8.0, 2.1 Hz, 1H), 3.79 (s, 3H), 2.63 (m, 1H), 2.53–2.49 (overlapped, 2H), 2.29 (dd, *J* = 24.3, 12.3 Hz, 1H), 1.43 (s, 3H), 1.38 (s, 3H), 1.37 (d, *J* = 6.4 Hz, 3H); ^13^C NMR (125 MHz, CDCl_3_): *δ* 166.2, 161.5, 157.3, 140.6, 138.5, 132.5, 131.3, 130.0, 127.7, 126.8, 126.8, 125.4, 125.4, 115.5, 108.3, 103.0, 99.4, 81.1, 75.6, 69.6, 69.0, 68.4, 55.4, 37.5, 35.8, 26.9, 26.8, 20.6; HRESIMS *m*/*z* 563.2261 [M + H]^+^ (calcd for C_30_H_34_O_7_F_3_, 563.2251); 85% yield.

**Compound 46**: Colorless oil; ^1^H NMR (500 MHz, CDCl_3_): *δ* ppm 7.53 (dd, *J* = 15.0, 8.4 Hz, 1H), 6.89 (td, *J* = 8.4, 1.4 Hz, 1H), 6.84 (d, *J* = 15.2 Hz, 1H), 6.81 (m, 1H), 6.50 (d, *J* = 2.0 Hz, 1H), 6.45 (d, *J* = 2.0 Hz, 1H), 5.99 (dt, *J* = 15.8, 5.0 Hz, 1H), 5.88 (m, 1H), 5.68–5.53 (overlapped, 2H), 5.09 (s, 2H), 4.55 (t, *J* = 8.0 Hz, 1H), 4.11 (m, 1H), 3.93 (dd, *J* = 8.0, 2.1 Hz, 1H), 3.80 (s, 3H), 2.62 (m, 1H), 2.54–2.46 (overlapped, 2H), 2.28 (dd, *J* = 24.2, 12.3 Hz, 1H), 1.43 (s, 3H), 1.37 (s, 3H), 1.36 (d, *J* = 6.8 Hz, 3H); ^13^C NMR (125 MHz, CDCl_3_): *δ* 166.2, 161.6, 157.2, 138.4, 132.5, 131.3, 130.2, 130.0, 127.6, 119.7, 115.5, 111.6, 108.3, 103.7, 103.5, 103.3, 103.3, 99.2, 81.1, 75.6, 68.9, 68.4, 63.8, 55.4, 37.5, 35.7, 26.9, 26.8, 20.6; HRESIMS *m*/*z* 531.2197 [M + H]^+^ (calcd for C_29_H_33_O_7_F_2_, 531.2189); 80% yield.

**Compound 47**: Colorless oil; ^1^H NMR (500 MHz, CDCl_3_): *δ* ppm 7.25–7.21 (overlapped, 3H), 7.10 (d, *J* = 7.2 Hz, 1H), 6.84 (d, *J* = 15.8 Hz, 1H), 6.48 (d, *J* = 2.1 Hz, 1H), 6.43 (d, *J* = 2.1 Hz, 1H), 6.00 (dt, *J* = 15.8, 5.0 Hz, 1H), 5.89 (ddd, *J* = 15.8, 10.4, 4.5 Hz, 1H), 5.66 (dd, *J* = 15.8, 8.4 Hz, 1H), 5.61 (m, 1H), 5.07 (d, *J* = 12.0 Hz, 1H), 5.04 (d, *J* = 12.0 Hz, 1H), 4.55 (t, *J* = 8.0 Hz, 1H), 4.12 (m, 1H), 3.95 (dd, *J* = 8.0, 2.1 Hz, 1H), 3.79 (s, 3H), 2.630 (m, 1H), 2.54–2.48 (overlapped, 2H), 2.35 (s, 3H), 2.29 (dd, *J* = 24.2, 12.2 Hz, 1H), 1.44 (s, 3H), 1.39 (s, 3H), 1.38 (d, *J* = 6.4 Hz, 3H); ^13^C NMR (125 MHz, CDCl_3_): *δ* 166.4, 161.5, 157.6, 138.0, 138.0, 136.5, 132.6, 131.4, 129.9, 128.5, 128.3, 127.5, 127.4, 123.9, 115.7, 108.3, 102.8, 99.5, 81.2, 75.5, 70.6, 68.9, 68.4, 55.4, 37.6, 35.7, 26.9, 26.8, 21.4, 20.6; HRESIMS *m*/*z* 509.2542 [M + H]^+^ (calcd for C_30_H_37_O_7_, 509.2534); 82% yield.

**Compound 48**: Colorless oil; ^1^H NMR (500 MHz, CDCl_3_): *δ* ppm 7.62 (d, *J* = 7.6 Hz, 1H), 7.53 (d, *J* = 7.6 Hz, 1H), 7.33 (t, *J* = 7.6 Hz, 1H), 7.15 (t, *J* = 7.6 Hz, 1H), 6.87 (d, *J* = 15.6 Hz, 1H), 6.50 (d, *J* = 1.6 Hz, 1H), 6.44 (d, *J* = 1.6 Hz, 1H), 6.00 (dt, *J* = 15.6, 4.8 Hz, 1H), 5.89 (m, 1H), 5.67–5.59 (overlapped, 2H), 5.13 (s, 2H), 4.55 (t, *J* = 8.2 Hz, 1H), 4.12 (d, *J* = 8.2 Hz, 1H), 3.95 (m, 1H), 3.80 (s, 3H), 2.65–2.57 (overlapped, 2H), 2.53 (m, 1H), 2.29 (dd, *J* = 24.2, 12.3 Hz, 1H), 1.43 (s, 3H), 1.39 (d, *J* = 6.4 Hz, 3H), 1.38 (s, 3H); ^13^C NMR (125 MHz, CDCl_3_): *δ* 166.3, 161.6, 157.3, 138.4, 135.8, 132.6, 132.3, 131.4, 130.0, 129.0, 128.4, 127.6, 127.6, 121.4, 115.3, 108.3, 103.1, 99.3, 81.1, 75.6, 69.7, 68.9, 68.4, 55.4, 37.5, 35.8, 26.9, 26.8, 20.6; HRESIMS *m*/*z* 573.1503 [M + H]^+^ (calcd for C_29_H_34_O_7_Br, 573.1482); 89% yield.

**Compound 49**: Colorless oil; ^1^H NMR (500 MHz, CDCl_3_): *δ* ppm 6.86 (dd, *J* = 15.8, 2.0 Hz, 1H), 6.61 (d, *J* = 2.2 Hz, 2H), 6.48 (d, *J* = 2.2 Hz, 1H), 6.41 (d, *J* = 2.2 Hz, 1H), 6.38 (t, *J* = 2.0 Hz, 1H), 6.00 (dt, *J* = 15.8, 5.0 Hz, 1H), 5.87 (m, 1H), 5.68–5.55 (overlapped, 2H), 5.05 (d, *J* = 12.4 Hz, 1H), 5.02 (d, *J* = 12.4 Hz, 1H), 4.55 (t, *J* = 8.0 Hz, 1H), 4.12 (m, 1H), 3.94 (dd, *J* = 8.0, 2.1 Hz, 1H), 3.79 (s, 6H), 3.78 (s, 3H), 2.63 (m, 1H), 2.55–2.49 (overlapped, 2H), 2.29 (dd, *J* = 24.2, 12.3 Hz, 1H), 1.44 (s, 3H), 1.38 (s, 3H), 1.38 (d, *J* = 6.2 Hz, 3H); ^13^C NMR (125 MHz, CDCl_3_): *δ* 166.3, 161.5, 160.9, 157.6, 139.1, 138.3, 132.6, 131.4, 129.9, 127.5, 115.5, 108.3, 104.4, 102.9, 99.8, 99.5, 81.1, 75.6, 70.3, 68.8, 68.4, 55.4, 55.3, 37.5, 35.7, 26.9, 26.8, 20.7; HRESIMS *m*/*z* 555.2598 [M + H]^+^ (calcd for C_31_H_39_O_9_, 555.2589); 86% yield.

**Compound 50**: Colorless oil; ^1^H NMR (500 MHz, CDCl_3_): *δ* ppm 6.86 (dd, *J* = 15.8, 1.0 Hz, 1H), 6.61 (d, *J* = 2.2 Hz, 2H), 6.48 (d, *J* = 2.1 Hz, 1H), 6.41 (d, *J* = 2.1 Hz, 1H), 6.38 (t, *J* = 2.2 Hz, 1H), 6.00 (dt, *J* = 15.8, 5.0 Hz, 1H), 5.87 (m, 1H), 5.61 (m, 2H), 5.05 (d, *J* = 12.5 Hz, 1H), 5.02 (d, *J* = 12.4 Hz, 1H), 4.55 (t, *J* = 8.2 Hz, 1H), 4.12 (dd, *J* = 9.7, 4.5 Hz, 1H), 3.94 (dd, *J* = 8.0, 2.1 Hz, 1H), 3.79 (s, 6H), 3.78 (s, 3H), 2.63 (m, 1H), 2.55–2.49 (overlapped, 2H), 2.29 (dd, *J* = 24.2, 12.3 Hz, 1H), 1.44 (s, 3H), 1.38 (s, 3H), 1.38 (d, *J* = 6.2 Hz, 3H); ^13^C NMR (125 MHz, CDCl_3_): *δ* 166.3, 161.5, 160.9, 157.6, 139.1, 138.3, 132.6, 131.4, 129.9, 127.5, 115.5, 108.3, 104.4, 102.9, 99.8, 99.5, 81.1, 75.6, 70.3, 68.8, 68.4, 55.4, 55.3, 37.5, 35.7, 26.9, 26.8, 20.7; HRESIMS *m*/*z* 589.2208 [M + H]^+^ (calcd for C_31_H_38_O_9_Cl, 589.2199); 85% yield.

**Compound 51**: Colorless oil; ^1^H NMR (500 MHz, CDCl_3_): *δ* ppm 8.03 (d, *J* = 8.2 Hz, 2H), 7.50 (d, *J* = 8.2 Hz, 2H), 6.86 (d, *J* = 15.6 Hz, 1H), 6.49 (d, *J* = 1.8 Hz, 1H), 6.39 (d, *J* = 1.8 Hz, 1H), 6.00 (dt, *J* = 15.6, 4.9 Hz, 1H), 5.89 (m, 1H), 5.68–5.59 (overlapped, 2H), 5.16 (d, *J* = 13.2 Hz, 1H), 5.13 (d, *J* = 13.2 Hz, 1H), 4.55 (t, *J* = 8.2 Hz, 1H), 4.11 (m, 1H), 3.94 (dd, *J* = 8.2, 1.9 Hz, 1H), 3.91 (s, 3H), 3.78 (s, 3H), 2.63 (m, 1H), 2.56–2.48 (overlapped, 2H), 2.29 (dd, *J* = 24.2, 12.2 Hz, 1H), 1.44 (s, 3H), 1.38 (s, 3H), 1.38 (d, *J* = 6.8 Hz, 3H); ^13^C NMR (125 MHz, CDCl_3_): *δ* 166.8, 166.3, 161.5, 157.3, 141.8, 138.4, 132.5, 131.4, 130.0, 129.8, 129.5, 127.7, 126.4, 115.5, 108.3, 103.0, 99.4, 81.1, 75.6, 69.8, 69.0, 68.4, 55.4, 52.1, 37.5, 35.7, 26.9, 26.8, 20.7; HRESIMS *m*/*z* 553.2441 [M + H]^+^ (calcd for C_31_H_37_O_9_, 553.2432); 84% yield.

**Compound 52**: White, amorphous powder; ^1^H NMR (500 MHz, CDCl_3_): *δ* ppm 8.05 (d, *J* = 8.2 Hz, 2H), 7.47 (d, *J* = 8.2 Hz, 2H), 6.45 (d, *J* = 15.8 Hz, 1H), 6.43 (s, 1H), 5.96–5.92 (overlapped, 2H), 5.75 (dd, *J* = 15.8, 8.1 Hz, 1H), 5.65 (m, 1H), 5.18 (d, *J* = 12.8 Hz, 1H), 5.15 (d, *J* = 12.8 Hz, 1H), 4.51 (t, *J* = 8.2 Hz, 1H), 4.16 (m, 1H), 3.92 (s, 3H), 3.90 (dd, *J* = 8.2, 2.3 Hz, 1H), 3.84 (s, 3H), 2.75 (m, 1H), 2.47–2.38 (overlapped, 2H), 2.29 (m, 1H), 1.44 (s, 3H), 1.43 (s, 3H), 1.25 (d, *J* = 6.4 Hz, 3H); ^13^C NMR (125 MHz, CDCl_3_): *δ* 166.7, 166.3, 156.5, 154.4, 141.2, 135.5, 132.8, 131.3, 129.9, 129.1, 127.1, 126.7, 117.6, 114.0, 109.9, 108.5, 96.8, 81.5, 75.1, 70.4, 69.4, 67.9, 56.4, 52.1, 37.9, 35.3, 26.9, 26.8, 21.1; HRESIMS *m*/*z* 587.2051 [M + H]^+^ (calcd for C_31_H_36_O_9_Cl, 587.2042); 82% yield.

**Compound 53**: Colorless oil; ^1^H NMR (500 MHz, CDCl_3_): *δ* ppm 7.90–7.80 (overlapped, 4H), 7.53 (d, *J* = 8.5 Hz, 1H), 7.48 (m, 2H), 6.86 (d, *J* = 15.8 Hz, 1H), 6.49 (d, *J* = 1.8 Hz, 1H), 6.48 (d, *J* = 1.8 Hz,, 1H), 6.01 (dt, *J* = 15.8, 5.0 Hz, 1H), 5.90 (ddd, *J* = 15.3, 10.5, 4.4 Hz, 1H), 5.71–5.57 (overlapped, 2H), 5.28 (d, *J* = 12.7 Hz, 1H), 5.25 (d, *J* = 12.7 Hz, 1H), 4.56 (t, *J* = 8.0 Hz, 1H), 4.13 (m, 1H), 3.96 (dd, *J* = 8.0, 2.0 Hz, 1H), 3.78 (s, 3H), 2.63 (m, 1H), 2.56–2.48 (overlapped, 2H), 2.30 (dd, *J* = 24.1, 12.3 Hz, 1H), 1.44 (s, 3H), 1.40 (s, 3H), 1.39 (d, *J* = 6.4 Hz, 3H); ^13^C NMR (125 MHz, CDCl_3_): *δ* 166.4, 161.5, 157.6, 138.2, 134.1, 133.2, 133.0, 132.6, 131.4, 129.9, 128.3, 127.9, 127.7, 127.5, 126.1, 125.9, 125.7, 124.7, 115.7, 108.3, 102.9, 99.6, 81.2, 75.6, 70.7, 69.0, 68.4, 55.4, 37.6, 35.7, 26.9, 26.8, 20.7; HRESIMS *m*/*z* 545.2543 [M + H]^+^ (calcd for C_33_H_37_O_7_, 545.2534); 87% yield.

**Compound 54**: Colorless oil; ^1^H NMR (500 MHz, CDCl_3_): *δ* ppm 11.48 (s, 1H), 7.13 (dd, *J* = 15.2, 1.9 Hz, 1H), 6.44 (d, *J* = 2.5 Hz, 1H), 6.36 (d, *J* = 2.5 Hz, 1H), 5.97 (ddd, *J* = 15.2, 8.1, 5.2 Hz, 1H), 5.71 (ddd, *J* = 15.2, 10.5, 3.0 Hz, 1H), 5.50 (dd, *J* = 15.2, 8.8 Hz, 1H), 5.43 (m, 1H), 4.55 (t, *J* = 8.0 Hz, 1H), 4.19 (m, 1H), 3.87 (dd, *J* = 8.0, 2.1 Hz, 1H), 3.78 (s, 3H), 2.73 (m, 1H), 2.49 (ddd, *J* = 15.2, 8.1, 3.3 Hz, 1H), 2.41 (dtd, *J* = 15.2, 5.2, 1.3 Hz, 1H), 2.27 (m, 1H), 1.42 (d, *J* = 6.4 Hz, 3H); ^13^C NMR (125 MHz, CDCl_3_): *δ* 170.7, 164.6, 163.8, 142.0, 133.9, 132.6, 129.4, 126.4, 108.2, 107.1, 104.3, 100.0, 81.3, 75.1, 70.5, 68.7, 55.3, 37.8, 36.0, 19.1; HRESIMS *m*/*z* 409.2146 [M – H]^–^ (calcd for C_22_H_21_D_6_O_7_, 409.2139); 93% yield.

**Compound 55**: Colorless oil; ^1^H NMR (500 MHz, CDCl_3_): *δ* ppm 11.35 (s, 1H), 6.62 (dd, *J* = 16.0, 1.2 Hz, 1H), 6.44 (s, 1H), 5.97 (ddd, *J* = 15.2, 6.9, 5.1 Hz, 1H), 5.70–5.29 (overlapped, 3H), 4.53 (t, *J* = 8.4 Hz, 1H), 4.18 (ddd, *J* = 12.3, 4.9, 2.2 Hz, 1H), 3.89 (s, 3H), 3.79 (dd, *J* = 8.4, 2.2 Hz, 1H), 2.85 (m, 1H), 2.54 (dd, *J* = 15.6, 7.0 Hz, 1H), 2.41 (m, 1H), 2.34 (m, 1H), 1.37 (d, *J* = 6.3 Hz, 3H); ^13^C NMR (125 MHz, CDCl_3_): *δ* 170.4, 162.2, 159.7, 139.7, 130.8, 130.6, 130.2, 128.8, 113.9, 108.3, 106.2, 99.3, 80.9, 75.3, 71.2, 68.2, 56.3, 37.0, 34.7, 19.3; HRESIMS *m*/*z* 409.2146 [M – H]^–^ (calcd for C_22_H_21_D_6_ClO_7_, 409.2139); 92% yield.

**Compound 56**: Colorless oil; ^1^H NMR (500 MHz, CDCl_3_): *δ* ppm 7.04 (dd, *J* = 15.6, 1.7 Hz, 1H), 6.77 (d, *J* = 2.5 Hz, 1H), 6.53 (d, *J* = 2.5 Hz, 1H), 5.98 (dt, *J* = 15.6, 5.1 Hz, 1H), 5.80 (m, 1H), 5.48 (dt, *J* = 15.6, 8.0 Hz, 1H), 5.42 (m, 1H), 4.54 (t, *J* = 8.0 Hz, 1H), 4.15 (ddd, *J* = 12.2, 4.2, 2.2 Hz, 1H), 3.86 (dd, *J* = 8.0, 2.2 Hz, 1H), 3.81 (s, 3H), 2.66 (ddd, *J* = 13.8, 6.1, 3.9 Hz, 1H), 2.58 (q, *J* = 7.5 Hz, 2H), 2.52–2.47 (overlapped, 2H), 2.28 (dd, *J* = 25.2, 11.9 Hz, 1H), 1.36 (d, *J* = 6.3 Hz, 3H), 1.24 (t, *J* = 7.6 Hz, 3H); ^13^C NMR (125 MHz, CDCl_3_): *δ* 172.8, 165.1, 161.5, 151.2, 139.7, 132.7, 131.2, 130.2, 128.0, 116.9, 110.1, 108.3, 107.7, 81.0, 75.7, 69.4, 68.4, 55.5, 36.9, 36.0, 27.5, 19.9, 8.8; HRESIMS *m*/*z* 489.2370 [M + Na]^+^ (calcd for C_25_H_26_D_6_O_8_Na, 489.2366); 17% yield.

**Compound 57**: Colorless oil; ^1^H NMR (500 MHz, CDCl_3_): *δ* ppm 7.06 (dd, *J* = 15.6, 1.6 Hz, 1H), 6.77 (d, *J* = 2.4 Hz, 1H), 6.55 (d, *J* = 2.4 Hz, 1H), 6.01 (dt, *J* = 15.6, 5.1 Hz, 1H), 5.85 (m, 1H), 5.49 (dt, *J* = 16.3, 8.2 Hz, 1H), 5.45–5.32 (overlapped, 2H), 4.59 (t, *J* = 8.2 Hz, 1H), 3.96 (dd, *J* = 7.9, 2.3 Hz, 1H), 3.82 (s, 3H), 2.65–2.55 (overlapped, 3H), 2.53–2.49 (overlapped, 2H), 2.40–2.36 (overlapped, 3H), 1.37 (d, *J* = 6.3 Hz, 3H), 1.25 (t, *J* = 7.5 Hz, 3H), 1.18 (t, *J* = 6.9 Hz, 3H); ^13^C NMR (125 MHz, CDCl_3_): *δ* 173.5, 172.7, 165.0, 161.6, 151.2, 139.4, 133.1, 131.0, 130.3, 127.3, 116.8, 110.0, 108.6, 108.0, 79.4, 76.5, 69.7, 69.4, 55.5, 37.0, 35.0, 27.7, 27.5, 20.0, 9.1, 8.8; 26% yield.

**Compound 58**: Colorless oil; ^1^H NMR (500 MHz, CDCl_3_): *δ* ppm 7.62 (d, *J* = 7.6 Hz, 1H), 7.54 (d, *J* = 7.6 Hz, 1H), 7.34 (t, *J* = 7.6 Hz, 1H), 7.16 (t, *J* = 7.6 Hz, 1H), 6.87 (d, *J* = 16.2 Hz, 1H), 6.50 (d, *J* = 2.0 Hz, 1H), 6.44 (d, *J* = 2.0 Hz, 1H), 6.01 (dt, *J* = 15.8, 5.0 Hz, 1H), 5.89 (m, 1H), 5.71–5.58 (overlapped, 2H), 5.13 (s, 2H), 4.56 (t, *J* = 8.0 Hz, 1H), 4.12 (m, 1H), 3.95 (dd, *J* = 8.0, 2.0 Hz, 1H), 3.80 (s, 3H), 2.63 (m, 1H), 2.57–2.48 (overlapped, 2H), 2.29 (dd, *J* = 24.2, 12.4 Hz, 1H), 1.39 (d, *J* = 6.4 Hz, 3H); ^13^C NMR (125 MHz, CDCl_3_): *δ* 166.3, 161.6, 157.3, 138.4, 135.9, 132.6, 132.3, 131.4, 130.0, 129.0, 128.4, 127.6, 127.6, 121.4, 115.3, 108.2, 103.0, 99.3, 81.1, 75.6, 69.7, 68.9, 68.4, 55.4, 37.5, 35.7, 20.6; HRESIMS *m*/*z* 579.1871 [M + H]^+^ (calcd for C_29_H_28_D_6_O_7_Br, 579.1859); 83% yield.

**Compound 59**: Colorless oil; ^1^H NMR (500 MHz, CDCl_3_): *δ* ppm 7.57 (s, 1H), 7.42 (d, *J* = 7.8 Hz, 1H), 7.35 (d, *J* = 7.8 Hz, 1H), 7.23 (t, *J* = 7.8 Hz, 1H), 6.85 (d, *J* = 16.0 Hz, 1H), 6.50 (d, *J* = 2.0 Hz, 1H), 6.39 (d, *J* = 2.0 Hz, 1H), 6.00 (dt, *J* = 16.0, 5.0 Hz, 1H), 5.90 (ddd, *J* = 15.4, 10.6, 4.4 Hz, 1H), 5.70–5.57 (overlapped, 2H), 5.07 (d, *J* = 13.2 Hz, 1H), 5.04 (d, *J* = 13.2 Hz, 1H), 4.55 (t, *J* = 8.0 Hz, 1H), 4.12 (m, 1H), 3.94 (dd, *J* = 8.0, 2.1 Hz, 1H), 3.80 (s, 3H), 2.63 (m, 1H), 2.58–2.50 (overlapped, 2H), 2.29 (dd, *J* = 24.2, 12.2 Hz, 1H), 1.40 (d, *J* = 6.4 Hz, 3H); ^13^C NMR (125 MHz, CDCl_3_): *δ* 166.3, 161.5, 157.3, 138.9, 138.3, 132.5, 131.4, 130.8, 130.1, 129.9, 129.7, 127.6, 125.2, 122.5, 115.7, 108.2, 103.0, 99.5, 81.1, 75.5, 69.6, 69.0, 68.4, 55.4, 37.5, 35.7, 20.7; HRESIMS *m*/*z* 579.1871 [M + H]^+^ (calcd for C_29_H_28_D_6_O_7_Br, 579.1859); 84% yield.

**Compound 60**: Colorless oil; ^1^H NMR (500 MHz, CDCl_3_): *δ* ppm 7.32 (m, 1H), 7.18 (d, *J* = 7.8 Hz, 1H), 7.15 (d, *J* = 9.6 Hz, 1H), 6.98 (td, *J* = 8.5, 2.2 Hz, 1H), 6.85 (d, *J* = 15.6 Hz, 1H), 6.49 (d, *J* = 2.0 Hz, 1H), 6.39 (d, *J* = 2.0 Hz, 1H), 6.00 (dt, *J* = 15.6, 5.0 Hz, 1H), 5.89 (ddd, *J* = 15.6, 10.5, 4.4 Hz, 1H), 5.69–5.58 (overlapped, 2H), 5.10 (d, *J* = 12.6 Hz, 1H), 5.07 (d, *J* = 12.6 Hz, 1H), 4.55 (t, *J* = 8.0 Hz, 1H), 4.12 (m, 1H), 3.95 (dd, *J* = 8.0, 2.1 Hz, 1H), 3.79 (s, 3H), 2.63 (m, 1H), 2.55–2.50 (overlapped, 2H), 2.29 (dd, *J* = 24.3, 12.2 Hz, 1H), 1.39 (d, *J* = 6.4 Hz, 3H); ^13^C NMR (125 MHz, CDCl_3_): *δ* 166.3, 163.9, 161.9, 161.5, 157.3, 139.2, 138.3, 132.5, 131.4, 130.0, 127.6, 122.1, 115.6, 114.7, 113.6, 108.2, 103.0, 99.5, 81.1, 75.5, 69.7, 69.0, 68.4, 55.4, 37.5, 35.7, 20.6; HRESIMS *m*/*z* 517.2519 [M – H]^–^ (calcd for C_29_H_26_D_6_O_7_F, 517.2503); 86% yield.

**Compound 61**: Colorless oil; ^1^H NMR (500 MHz, CDCl_3_): *δ* ppm 7.30 (d, *J* = 7.9 Hz, 2H), 7.16 (d, *J* = 7.9 Hz, 2H), 6.84 (d, *J* = 15.8 Hz, 1H), 6.47 (d, *J* = 2.0 Hz, 1H), 6.42 (d, *J* = 2.0 Hz, 1H), 6.00 (dt, *J* = 15.8, 5.0 Hz, 1H), 5.88 (ddd, *J* = 15.8, 10.5, 4.4 Hz, 1H), 5.65 (dd, *J* = 15.8, 8.5 Hz, 1H), 5.60 (m, 1H), 5.07 (d, *J* = 12.0 Hz, 1H), 5.04 (d, *J* = 12.0 Hz, 1H), 4.55 (t, *J* = 8.0 Hz, 1H), 4.12 (m, 1H), 3.95 (dd, *J* = 8.0, 1.9 Hz, 1H), 3.78 (s, 3H), 2.62 (dd, *J* = 13.7, 1.4 Hz, 1H), 2.54–2.50 (overlapped, 2H), 2.34 (s, 3H), 2.29 (dd, *J* = 23.6, 12.9 Hz, 1H), 1.37 (d, *J* = 6.4 Hz, 3H); ^13^C NMR (125 MHz, CDCl_3_): *δ* 166.4, 161.5, 157.6, 138.1, 137.4, 133.6, 132.6, 132.6, 131.4, 130.0, 129.1, 127.4, 126.9, 108.2, 102.7, 99.5, 81.1, 75.5, 70.4, 68.9, 68.4, 55.4, 37.6, 35.7, 21.1, 20.6; HRESIMS *m*/*z* 515.2920 [M + H]^+^ (calcd for C_30_H_31_D_6_O_7_, 515.2910); 85% yield.

**Compound 62**: Colorless oil; ^1^H NMR (500 MHz, CDCl_3_): *δ* ppm 7.28–7.20 (overlapped, 3H), 7.10 (d, *J* = 7.2 Hz, 1H), 6.84 (d, *J* = 15.8 Hz, 1H), 6.48 (d, *J* = 1.6 Hz, 1H), 6.43 (d, *J* = 1.6 Hz, 1H), 6.00 (dt, *J* = 15.8, 4.9 Hz, 1H), 5.89 (ddd, *J* = 15.8, 10.6, 4.4 Hz, 1H), 5.66 (dd, *J* = 15.8, 8.6 Hz, 1H), 5.62 (m, 1H), 5.07 (d, *J* = 12.2 Hz, 1H), 5.04 (d, *J* = 12.2 Hz, 1H), 4.55 (t, *J* = 8.0 Hz, 1H), 4.12 (m, 1H), 3.95 (dd, *J* = 8.0, 1.8 Hz, 1H), 3.79 (s, 3H), 2.62 (m, 1H), 2.56–2.49 (overlapped, 2H), 2.35 (s, 3H), 2.29 (dd, *J* = 24.2, 12.2 Hz, 1H), 1.38 (d, *J* = 6.3 Hz, 3H); ^13^C NMR (125 MHz, CDCl_3_): *δ* ppm 166.4, 161.5, 157.6, 138.0, 136.5, 132.6, 131.4, 129.9, 128.6, 128.5, 128.3, 127.5, 127.4, 123.9, 115.7, 108.1, 102.8, 99.5, 81.1, 75.5, 70.6, 68.9, 68.4, 55.4, 37.6, 35.7, 21.4, 20.6; HRESIMS *m*/*z* 515.2919 [M + H]^+^ (calcd for C_30_H_31_D_6_O_7_, 515.2910); 80% yield.

**Compound 63**: Colorless oil; ^1^H NMR (500 MHz, CDCl_3_): *δ* ppm 7.44 (d, *J* = 8.0 Hz, 1H), 7.24–7.17 (overlapped, 3H), 6.83 (d, *J* = 15.8 Hz, 1H), 6.47 (d, *J* = 7.6 Hz, 2H), 5.99 (dt, *J* = 15.8, 5.0 Hz, 1H), 5.89 (m, 1H), 5.65 (dd, *J* = 15.8, 8.4 Hz, 1H), 5.58 (m, 1H), 5.07 (d, *J* = 12.0 Hz, 1H), 5.03 (d, *J* = 12.0 Hz, 1H), 4.55 (t, *J* = 8.0 Hz, 1H), 4.12 (m, 1H), 3.95 (dd, *J* = 8.0, 1.8 Hz, 1H), 3.80 (s, 3H), 2.63 (m, 1H), 2.53–2.46 (overlapped, 2H), 2.35 (s, 3H), 2.29 (dd, *J* = 24.1, 12.3 Hz, 1H), 1.33 (d, *J* = 6.4 Hz, 3H); ^13^C NMR (125 MHz, CDCl_3_): *δ* 166.4, 161.5, 157.6, 138.1, 136.0, 134.4, 132.6, 131.4, 130.1, 129.9, 127.9, 127.8, 127.5, 125.9, 115.6, 108.2, 102.6, 99.3, 81.1, 75.5, 69.1, 68.9, 68.4, 55.4, 37.6, 35.7, 20.6, 18.8; HRESIMS *m*/*z* 515.2920 [M + H]^+^ (calcd for C_30_H_31_D_6_O_7_, 515.2910); 83% yield.

**Compound 64**: Colorless oil; ^1^H NMR (500 MHz, CDCl_3_): *δ* ppm 7.87 (d, *J* = 10.6 Hz, 1H), 7.85–7.79 (overlapped, 3H), 7.54 (dd, *J* = 8.6, 1.2 Hz, 1H), 7.50–7.43 (overlapped, 2H), 6.86 (d, *J* = 16.0 Hz, 1H), 6.49 (d, *J* = 2.0 Hz, 1H), 6.48 (d, *J* = 2.0 Hz, 1H), 6.01 (dt, *J* = 16.0, 5.0 Hz, 1H), 5.90 (m, 1H), 5.72–5.57 (overlapped, 2H), 5.27 (d, *J* = 12.6 Hz, 1H), 5.24 (d, *J* = 12.6 Hz, 1H), 4.56 (t, *J* = 8.0 Hz, 1H), 4.12 (m, 1H), 3.96 (dd, *J* = 8.0, 2.2 Hz, 1H), 3.78 (s, 3H), 2.63 (m, 1H), 2.60–2.45 (overlapped, 2H), 2.30 (dd, *J* = 24.2, 12.2 Hz, 1H), 1.39 (d, *J* = 6.4 Hz, 3H); ^13^C NMR (125 MHz, CDCl_3_): *δ* 166.4, 161.5, 157.6, 138.2, 134.1, 133.2, 133.0, 132.6, 131.4, 130.0, 128.3, 127.9, 127.7, 127.5, 126.1, 125.9, 125.7, 124.7, 115.7, 108.2, 102.8, 99.6, 81.2, 75.5, 70.7, 69.0, 68.4, 55.4, 37.6, 35.7, 20.7; HRESIMS *m*/*z* 515.2920 [M + H]^+^ (calcd for C_30_H_31_D_6_O_7_, 515.2910); 87% yield.

**Compound 65**: Colorless oil; ^1^H NMR (500 MHz, CDCl_3_): *δ* ppm 7.76 (dd, *J* = 7.8, 0.8 Hz, 1H), 7.64 (td, *J* = 7.8, 1.2 Hz, 1H), 7.59–7.53 (overlapped, 4H), 7.51 (dd, *J* = 7.8, 0.5 Hz, 1H), 7.44 (td, *J* = 7.8, 1.1 Hz, 1H), 6.86 (d, *J* = 16.6 Hz, 1H), 6.50 (d, *J* = 2.2 Hz, 1H), 6.45 (d, *J* = 2.2 Hz, 1H), 6.01 (dt, *J* = 15.8, 5.0 Hz, 1H), 5.90 (m, 1H), 5.75–5.53 (overlapped, 2H), 5.18 (d, *J* = 12.4 Hz, 1H), 5.15 (d, *J* = 12.4 Hz, 1H), 4.56 (t, *J* = 8.0 Hz, 1H), 4.13 (m, 1H), 3.96 (dd, *J* = 8.0, 2.2 Hz, 1H), 3.80 (s, 3H), 2.64 (m, 1H), 2.54–2.50 (overlapped, 2H), 2.30 (dd, *J* = 24.3, 12.3 Hz, 1H), 1.39 (d, *J* = 6.4 Hz, 3H); ^13^C NMR (125 MHz, CDCl_3_): *δ* 166.3, 161.5, 157.5, 145.0, 138.3, 137.5, 137.3, 133.7, 132.8, 132.5, 131.4, 130.0, 130.0, 128.9, 127.5, 127.5, 127.0, 118.6, 115.6, 111.2, 109.9, 103.0, 99.5, 81.1, 75.5, 70.1, 69.0, 68.4, 55.4, 37.5, 35.7, 20.6; HRESIMS *m*/*z* 602.3026 [M + H]^+^ (calcd for C_36_H_32_D_6_O_7_N, 602.3019); 82% yield.

**Compound 66**: Colorless oil; ^1^H NMR (500 MHz, CDCl_3_): *δ* ppm 7.44 (dd, *J* = 16.9, 9.1 Hz, 1H), 6.93 (td, *J* = 9.6, 6.5 Hz, 1H), 6.87 (d, *J* = 15.8 Hz, 1H), 6.52 (d, *J* = 1.8 Hz, 1H), 6.42 (d, *J* = 1.8 Hz, 1H), 6.00 (dt, *J* = 15.8, 5.0 Hz, 1H), 5.89 (m, 1H), 5.73–5.45 (overlapped, 2H), 5.08 (s, 2H), 4.55 (t, *J* = 8.0 Hz, 1H), 4.12 (m, 1H), 3.93 (dd, *J* = 8.0, 1.9 Hz, 1H), 3.81 (s, 3H), 2.63 (m, 1H), 2.53–2.49 (overlapped, 2H), 2.29 (dd, *J* = 24.4, 12.2 Hz, 1H), 1.39 (d, *J* = 6.4 Hz, 3H); ^13^C NMR (125 MHz, CDCl_3_): *δ* ppm 166.1, 161.6, 157.0, 138.6, 132.4, 131.3, 130.0, 127.8, 116.9, 116.8, 115.4, 108.2, 105.4, 105.3, 105.2, 105.0, 103.5, 99.3, 81.1, 75.6, 69.1, 68.4, 63.3, 55.4, 37.4, 35.8, 20.5; HRESIMS *m*/*z* 553.2340 [M – H]^–^ (calcd for C_29_H_24_D_6_O_7_F_3_, 553.2315); 86% yield.

**Compound 67**: Colorless oil; ^1^H NMR (500 MHz, CDCl_3_): *δ* ppm 7.81 (dd, *J* = 7.6, 0.6 Hz, 1H), 7.52 (td, *J* = 7.6, 1.0 Hz, 1H), 7.45 (d, *J* = 8.0 Hz, 2H), 7.40 (td, *J* = 7.6, 0.8 Hz, 1H), 7.36 (d, *J* = 7.6 Hz, 1H), 7.30 (d, *J* = 8.0 Hz, 2H), 6.85 (d, *J* = 15.8 Hz, 1H), 6.49 (d, *J* = 1.8 Hz, 1H), 6.45 (d, *J* = 1.8 Hz, 1H), 6.00 (dt, *J* = 15.8, 5.0 Hz, 1H), 5.89 (m, 1H), 5.67–5.57 (overlapped, 2H), 5.16 (d, *J* = 12.4 Hz, 1H), 5.13 (d, *J* = 12.4 Hz, 1H), 4.55 (t, *J* = 8.0 Hz, 1H), 4.12 (m, 1H), 3.95 (dd, *J* = 8.0, 2.0 Hz, 1H), 3.79 (s, 3H), 3.60 (s, 3H), 2.62 (m, 1H), 2.55–2.49 (overlapped, 2H), 2.29 (dd, *J* = 24.2, 12.3 Hz, 1H), 1.38 (d, *J* = 6.4 Hz, 3H); ^13^C NMR (125 MHz, CDCl_3_): *δ* 169.1, 166.4, 161.4, 157.6, 142.0, 140.8, 138.1, 135.6, 132.5, 131.4, 131.2, 130.8, 130.6, 130.0, 129.7, 128.4, 127.5, 127.2, 126.5, 115.6, 108.2, 102.8, 99.6, 81.1, 75.5, 70.3, 68.9, 68.4, 55.4, 51.8, 37.5, 35.8, 20.6; HRESIMS *m*/*z* 633.2980 [M – H]^–^ (calcd for C_37_H_33_D_6_O_9_, 633.2965); 84% yield.

**Compound 68**: Colorless oil; ^1^H NMR (500 MHz, CDCl_3_): *δ* ppm 7.46 (t, *J* = 8.0 Hz, 1H), 7.31 (dd, *J* = 8.0, 1.2 Hz, 1H), 7.24 (dd, *J* = 9.5, 1.7 Hz, 1H), 6.85 (dd, *J* =15.8, 0.9, 1H), 6.50 (d, *J* = 2.1 Hz, 1H), 6.43 (d, *J* = 2.1 Hz, 1H), 6.00 (dt, *J* = 15.8, 5.0 Hz, 1H), 5.88 (m, 1H), 5.69–5.54 (overlapped, 2H), 5.10 (d, *J* = 13.2 Hz, 1H), 5.08 (d, *J* = 13.2 Hz, 1H), 4.55 (t, *J* = 8.0 Hz, 1H), 4.12 (ddd, *J* = 5.9, 3.7, 1.7 Hz, 1H), 3.93 (dd, *J* = 8.0, 2.2 Hz, 1H), 3.80 (s, 3H), 2.63 (m, 1H), 2.57–2.49 (overlapped, 2H), 2.29 (dd, *J* = 24.2, 12.2 Hz, 1H), 1.37 (d, *J* = 6.4 Hz, 3H); ^13^C NMR (125 MHz, CDCl_3_): *δ* 166.2, 161.6, 160.6, 158.6, 157.1, 138.5, 132.5, 131.3, 130.2, 130.0, 127.7, 127.7, 121.8, 118.8, 115.4, 108.2, 103.3, 99.2, 81.1, 75.5, 68.9, 68.4, 63.7, 55.4, 37.5, 35.7, 20.6; HRESIMS *m*/*z* 595.1630 [M – H]^–^ (calcd for C_29_H_25_D_6_O_7_BrF, 595.1608); 83% yield.

**Compound 69**: Colorless oil; ^1^H NMR (500 MHz, CDCl_3_): *δ* ppm 6.84 (d, *J* = 15.2 Hz, 1H), 6.72 (d, *J* = 2.0 Hz, 1H), 6.55 (d, *J* = 2.0 Hz, 1H), 6.03 (m, 1H), 5.94 (m, 1H), 5.63 (dd, *J* = 15.2, 7.8 Hz, 1H), 5.47 (m, 1H), 5.31 (br s, 1H), 5.15 (br s, 1H), 5.10 (br s, 1H), 3.80 (s, 3H), 2.64–2.54 (overlapped, 3H), 2.51–2.37 (overlapped, 3H), 2.39–2.17 (overlapped, 6H), 1.38 (d, *J* = 6.2 Hz, 3H), 1.23 (t, *J* = 7.5 Hz, 3H), 1.13–1.07 (overlapped, 9H); ^13^C NMR (125 MHz, CDCl_3_): *δ* 173.7, 173.3, 173.2, 172.5, 165.4, 161.1, 150.6, 138.9, 133.8, 131.2, 126.7, 117.5, 115.3, 110.4, 107.6, 77.2, 73.4, 72.7, 72.1, 55.5, 37.7, 29.6, 27.7, 27.5, 27.5, 27.4, 20.0, 9.2, 9.0, 8.8, 8.8; 32% yield.

**Compound 70**: Colorless oil; ^1^H NMR (500 MHz, CDCl_3_): *δ* ppm 6.65 (s, 1H), 6.64 (d, *J* = 15.8 Hz, 1H), 5.97 (m, 1H), 5.77 (m, 1H), 5.55 (dd, *J* = 15.8, 8.0 Hz, 1H), 5.43–5.37 (overlapped, 2H), 4.99–4.88 (overlapped, 2H), 3.88 (s, 3H), 2.74 (dd, *J* = 15.7, 10.8 Hz, 1H), 2.59–2.45 (overlapped, 3H), 2.41–2.16 (overlapped, 8H), 1.31 (d, *J* = 6.2 Hz, 3H), 1.21 (t, *J* = 7.6 Hz, 3H), 1.13 (t, *J* = 7.6 Hz, 3H), 1.09 (t, *J* = 7.6 Hz, 3H), 1.08 (t, *J* = 7.6 Hz, 3H);^13^C NMR (125 MHz, CDCl_3_): *δ* 173.8, 173.4, 173.4, 172.1, 165.3, 155.9, 147.0, 136.1, 135.1, 133.3, 127.6, 127.5, 120.7, 119.0, 105.5, 74.8, 73.9, 73.5, 72.3, 56.5, 37.9, 32.9, 27.6, 27.6, 27.3, 26.9, 20.6, 9.3, 9.0, 8.7, 8.7; HRESIMS *m*/*z* 621.2106 [M – H]^–^ (calcd for C_31_H_38_O_11_Cl, 621.2097); 28% yield.

**Compound 71**: ^1^H NMR (500 MHz, CDCl_3_): *δ* ppm 6.63 (dd, *J* = 15.8, 1.5 Hz, 1H), 6.59 (s, 1H), 5.95 (m, 1H), 5.80 (m, 1H), 5.48 (dd, *J* = 15.8, 9.0 Hz, 1H), 5.23 (t, *J* = 9.2 Hz, 1H), 4.89 (m, 1H), 4.84 (d, *J* = 6.9 Hz, 1H), 3.97 (dd, *J* = 9.2, 1.2 Hz, 1H), 3.89 (s, 3H), 2.71 (dd, *J* = 15.0, 11.1 Hz, 1H), 2.55 (m, 1H), 2.38 (m, 1H), 2.20 (m, 1H), 1.32 (s, 9H), 1.22 (s, 9H), 1.20 (overlapped, 12H). ^13^C NMR (125 MHz, CDCl_3_): *δ* 184.6, 178.1, 176.1, 165.0, 155.8, 147.2, 136.1, 134.7, 133.7, 127.5, 127.4, 120.5, 118.9, 105.2, 76.8, 74.9, 74.3, 74.0, 56.5, 39.1, 38.9, 38.7, 38.3, 32.9, 27.0, 27.0, 26.9, 21.0. HRESIMS *m*/*z* 649.2799 [M – H]^-^ (calcd for C_34_H_46_O_10_Cl, 649.2774); 26% yield.


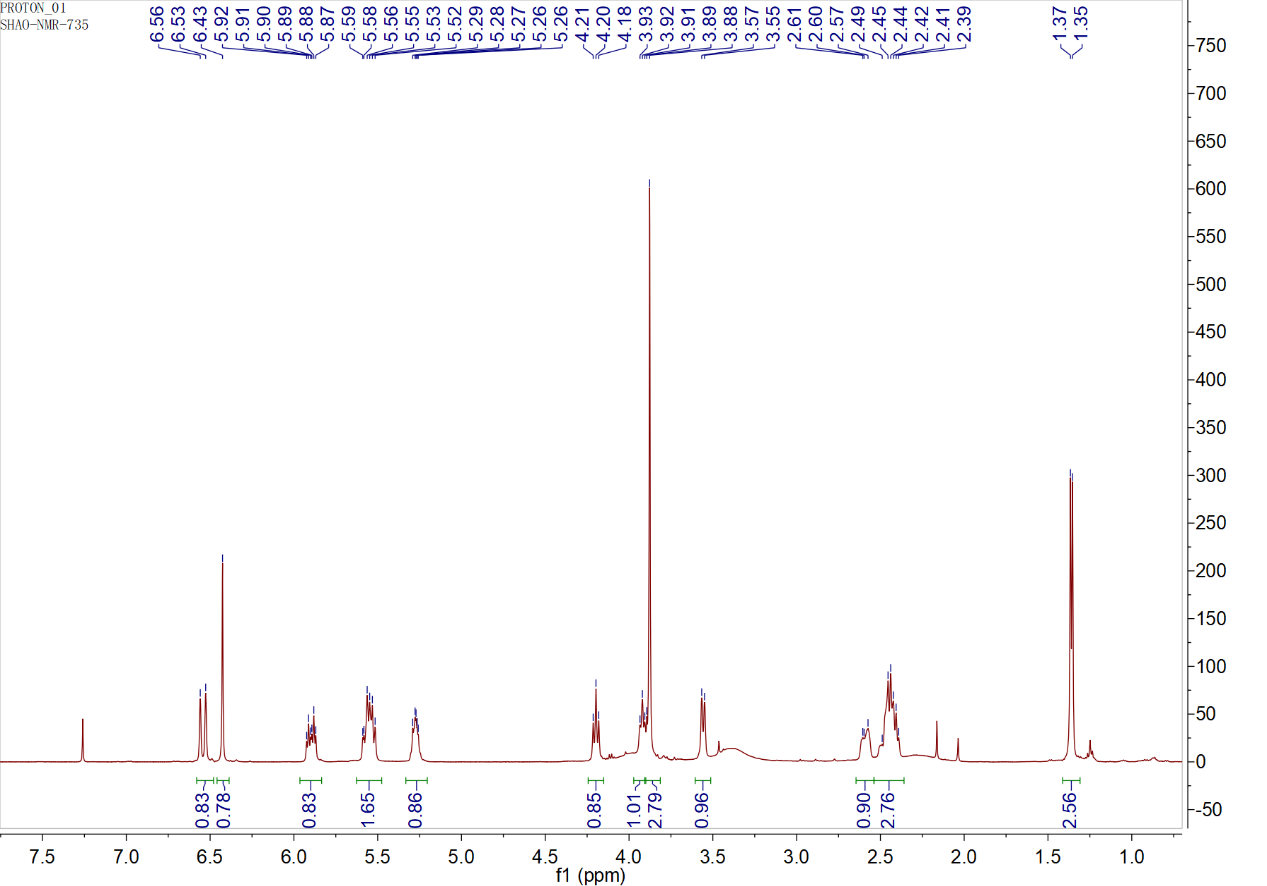


**Figure S1.** ^1^H NMR (500 MHz, CDCl_3_) spectrum of compound **2**


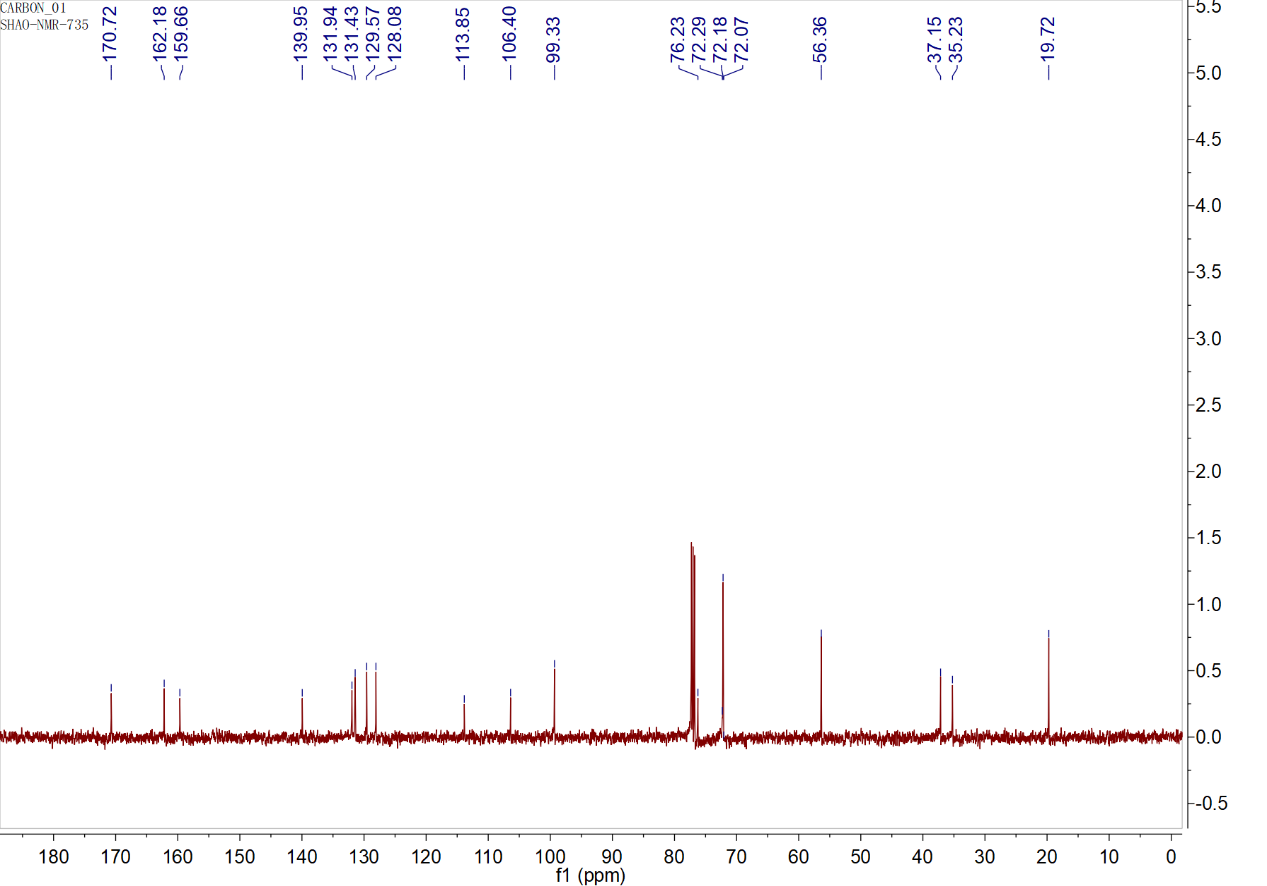


**Figure S2.** ^13^C NMR (125 MHz, CDCl_3_) spectrum of compound **2**

**Figure S3.** HRESIMS spectrum of compound **2**


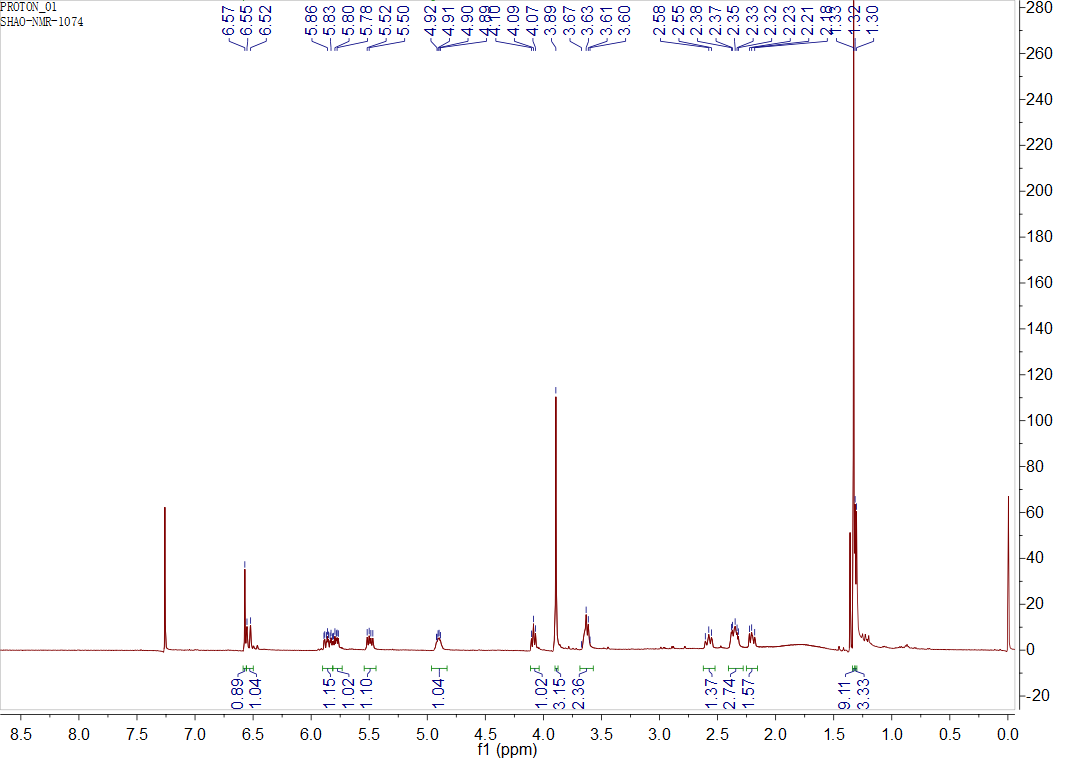


**Figure S4.** ^1^H NMR (500 MHz, CDCl_3_) spectrum of compound **3**


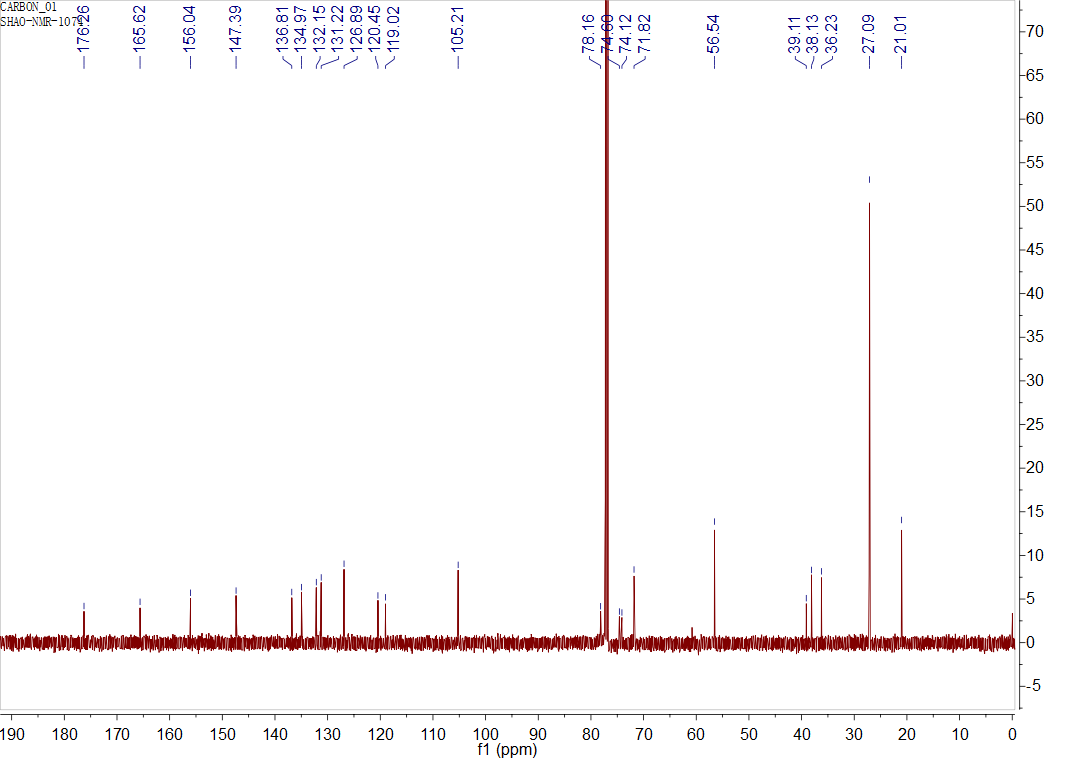


**Figure S5.** ^13^C NMR (125 MHz, CDCl_3_) spectrum of compound **3**

**Figure S6.** HRESIMS spectrum of compound **3**


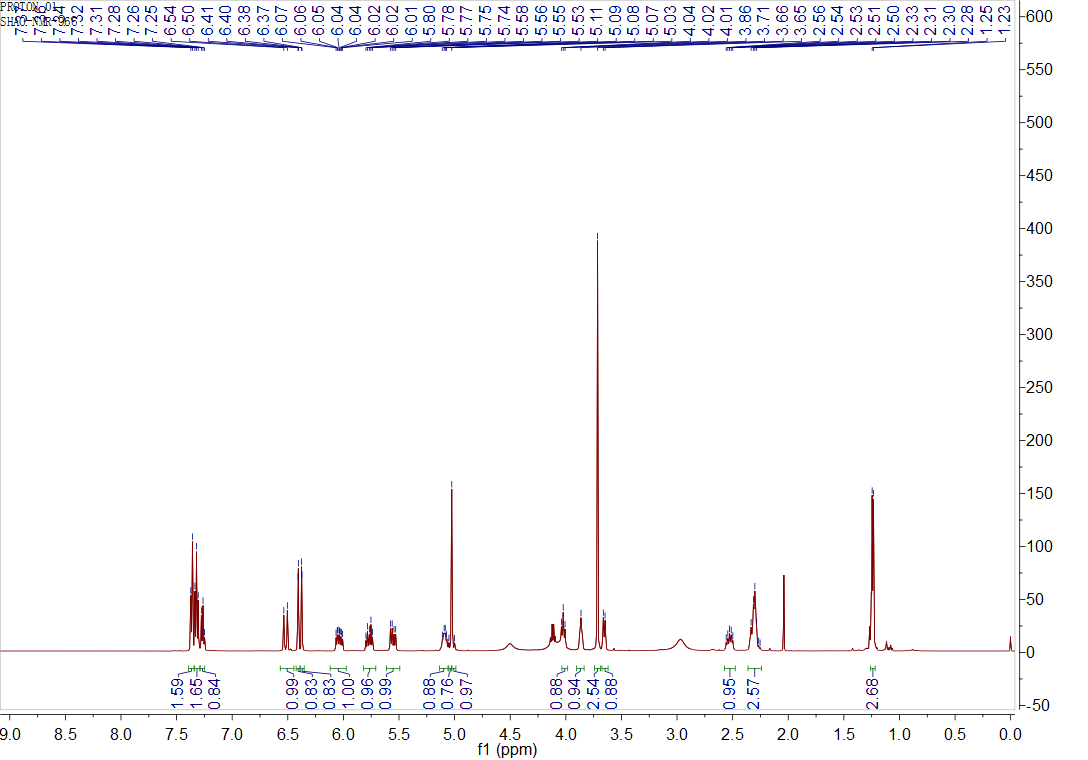


**Figure S7.** ^1^H NMR (500 MHz, CDCl_3_) spectrum of compound **4**


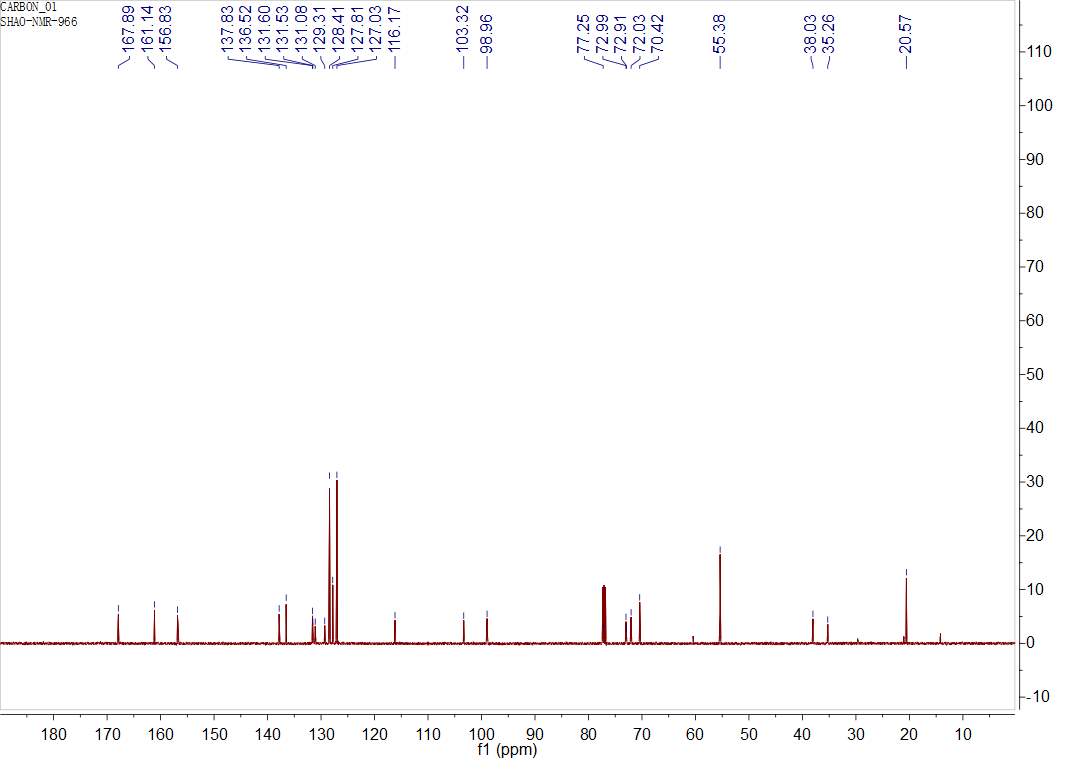


**Figure S8.** ^13^C NMR (125 MHz, CDCl_3_) spectrum of compound **4**

**Figure S9.** HRESIMS spectrum of compound **4**


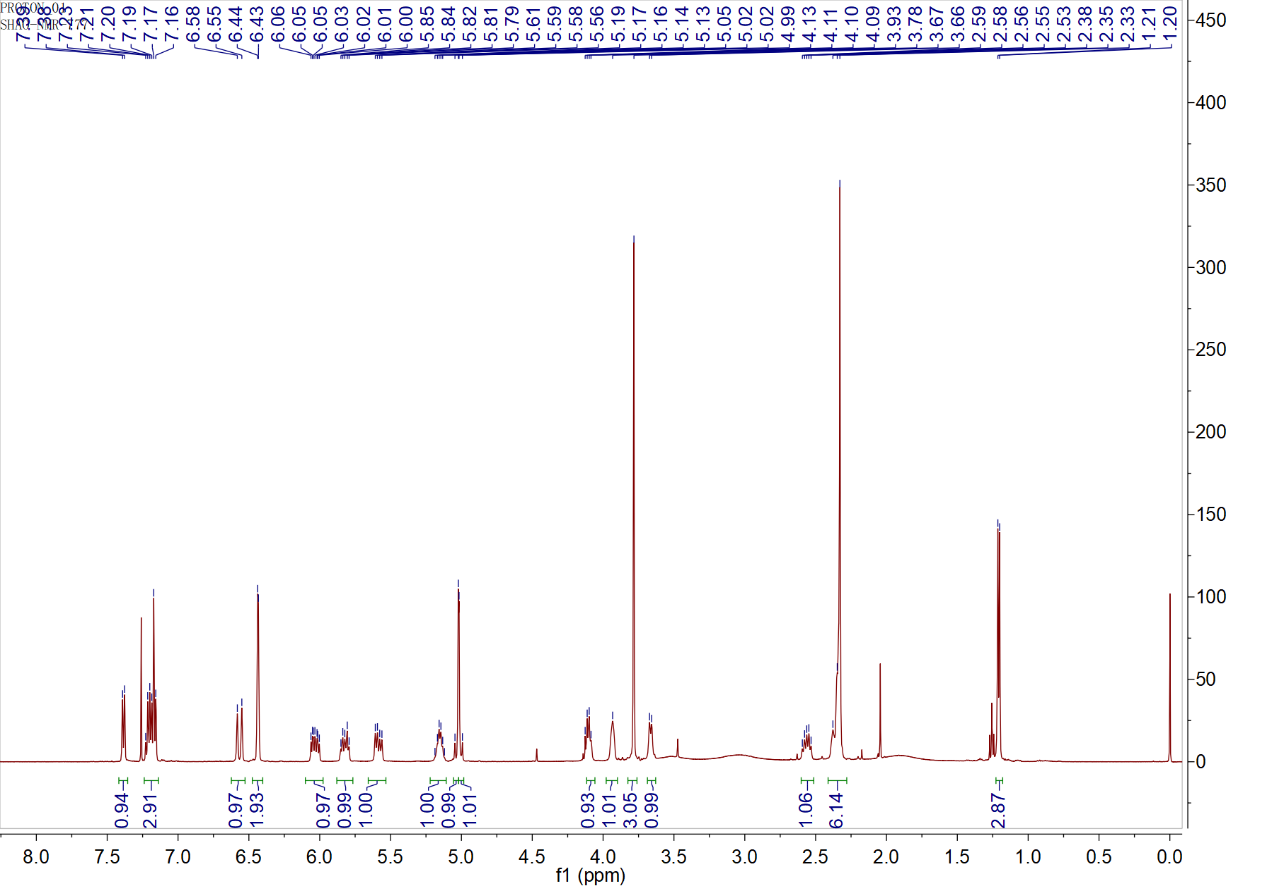


**Figure S10.** ^1^H NMR (500 MHz, CDCl_3_) spectrum of compound **5**


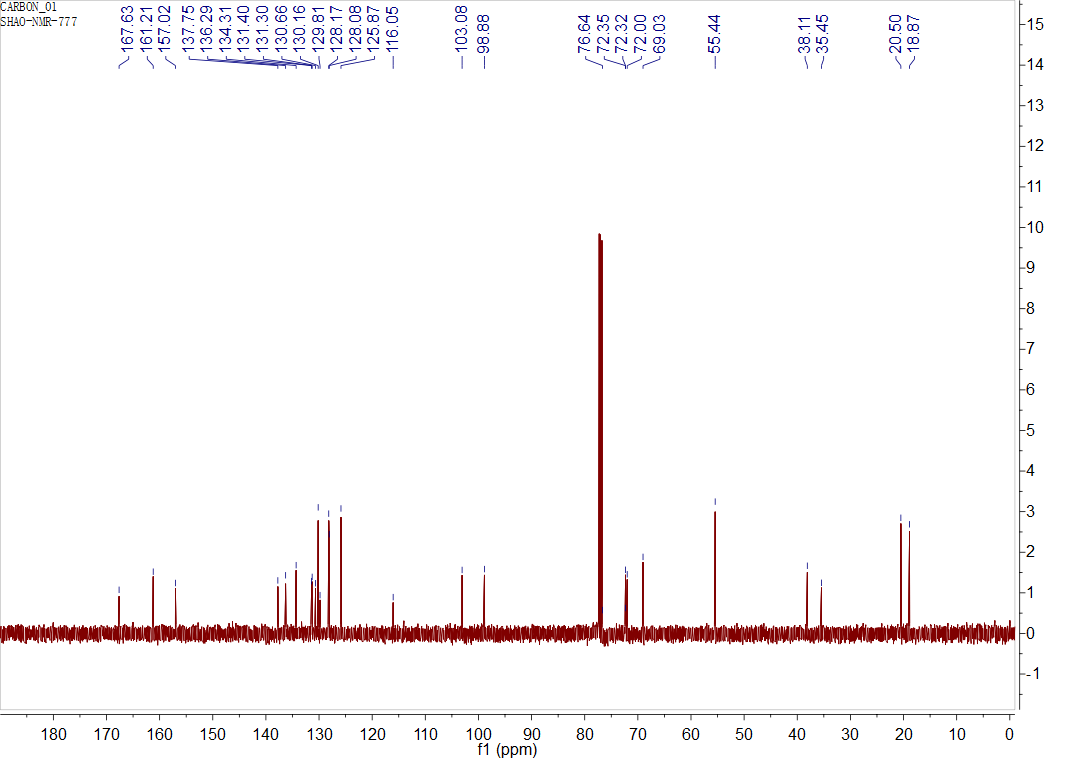


**Figure S11.** ^13^C NMR (125 MHz, CDCl_3_) spectrum of compound **5**

**Figure S12.** HRESIMS spectrum of compound **5**


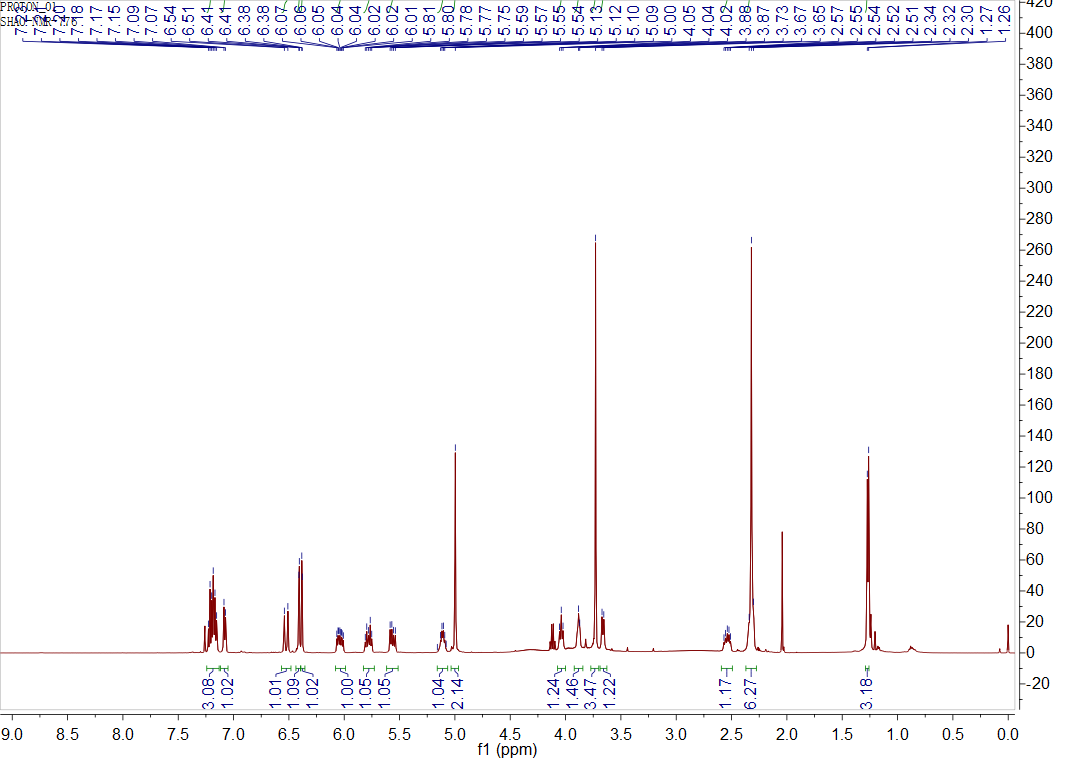


**Figure S13.** ^1^H NMR (500 MHz, CDCl_3_) spectrum of compound **6**


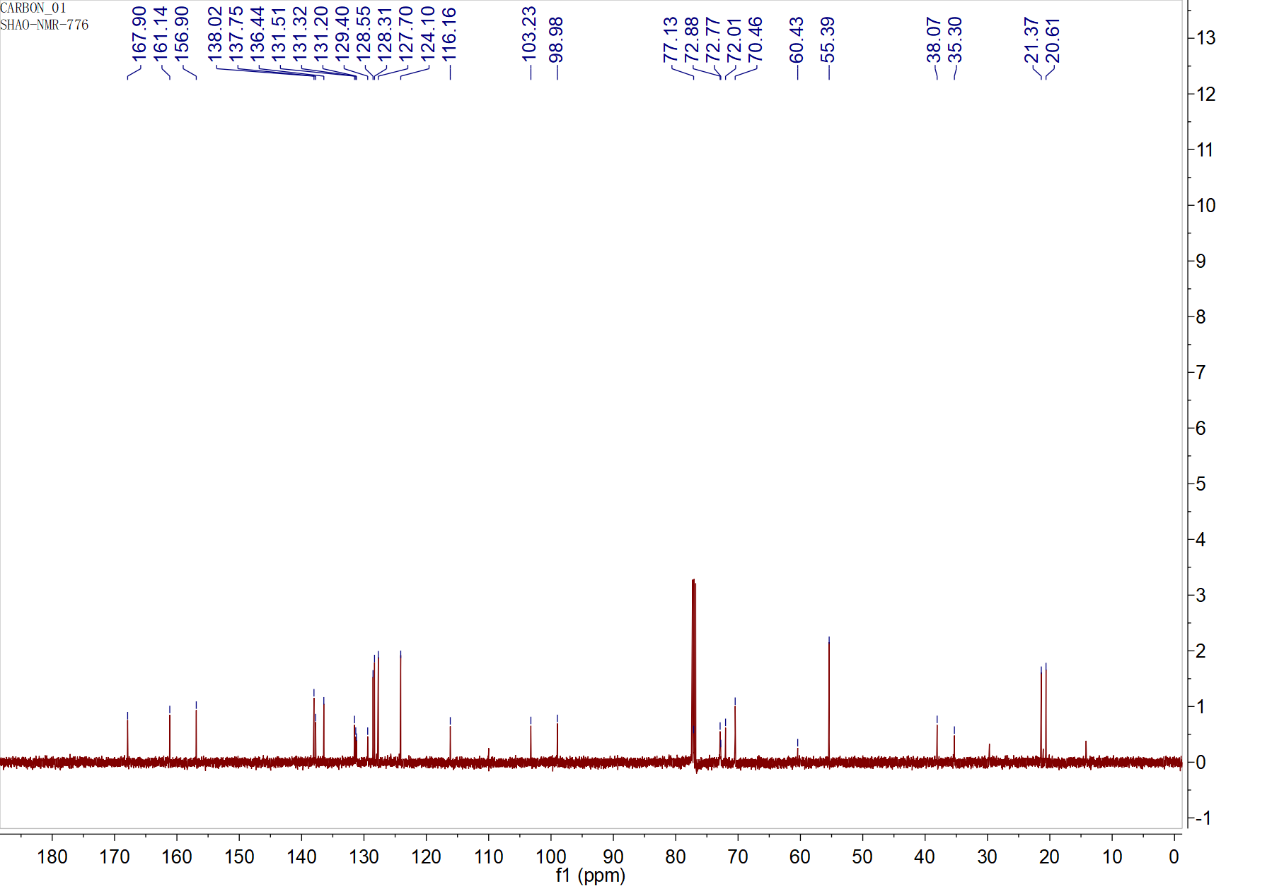


**Figure S14.** ^13^C NMR (125 MHz, CDCl_3_) spectrum of compound **6**

**Figure S15.** HRESIMS spectrum of compound **6**


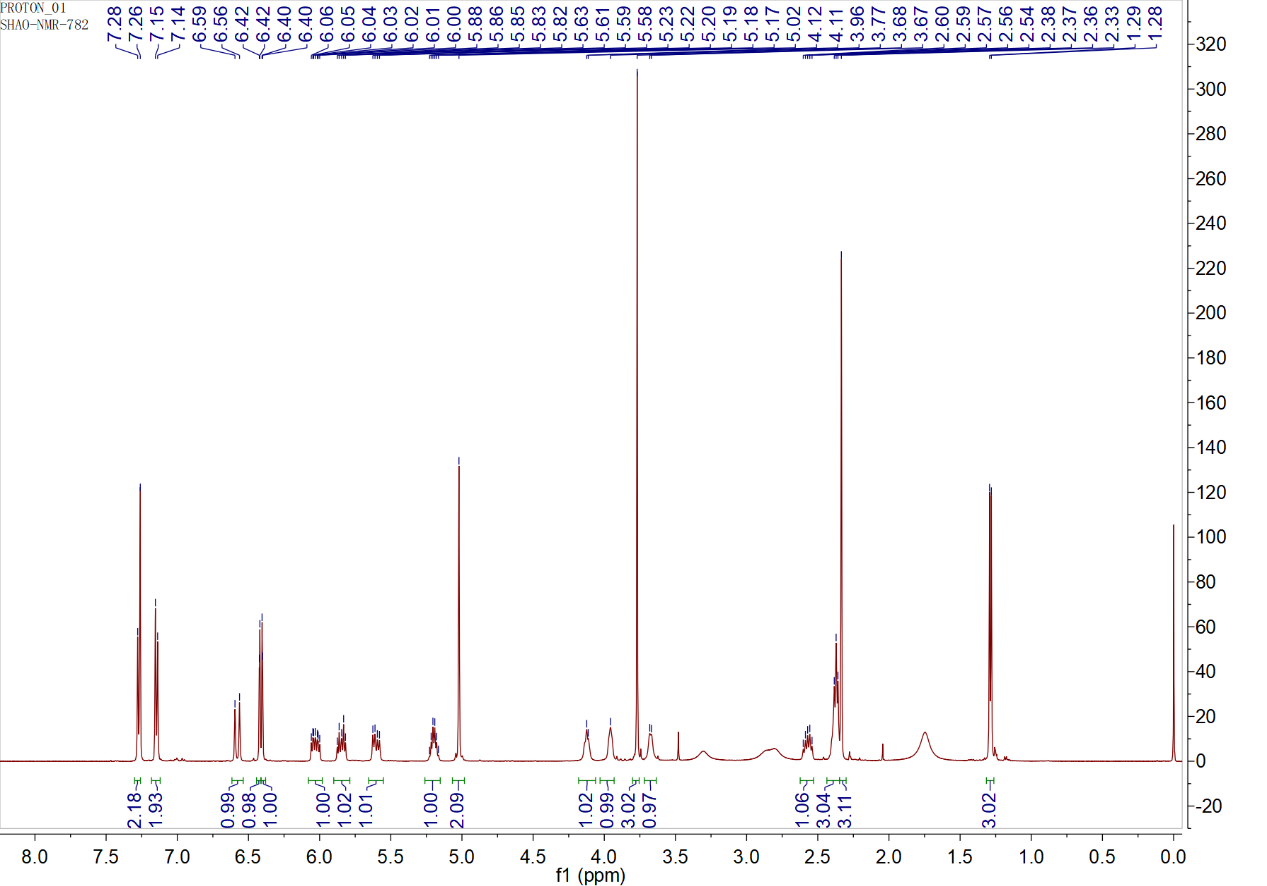


**Figure S16.** ^1^H NMR (500 MHz, CDCl_3_) spectrum of compound **7**


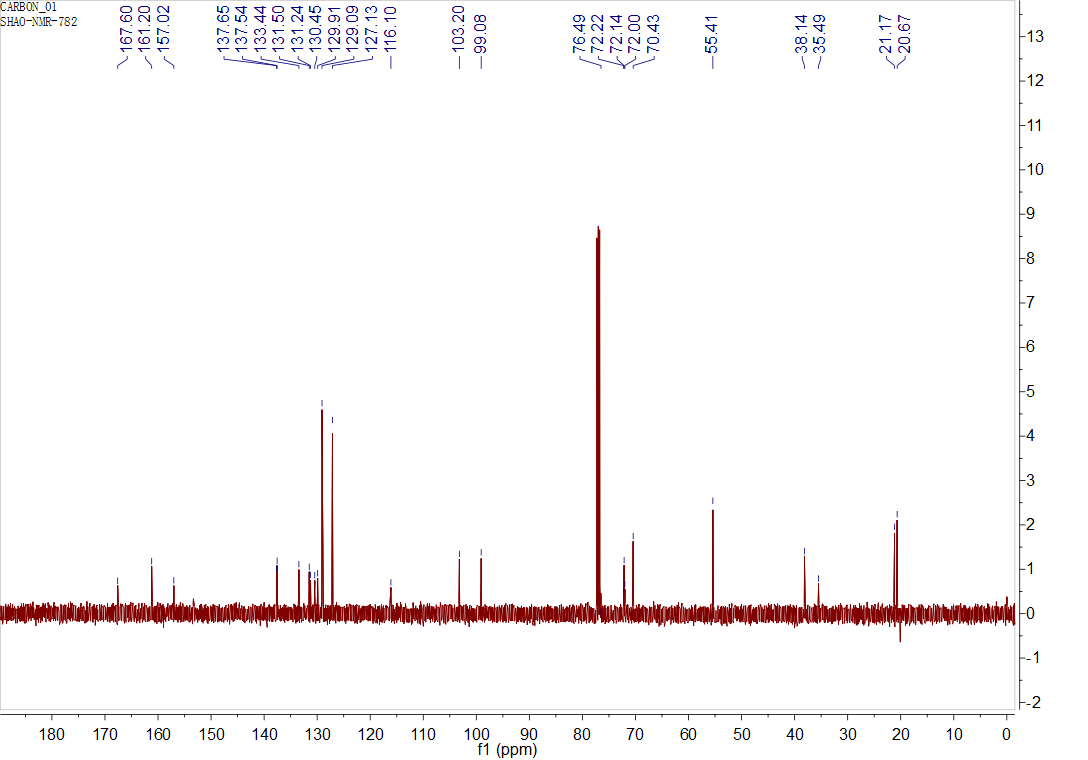


**Figure S17.** ^13^C NMR (125 MHz, CDCl_3_) spectrum of compound **7**

**Figure S18.** HRESIMS spectrum of compound **7**


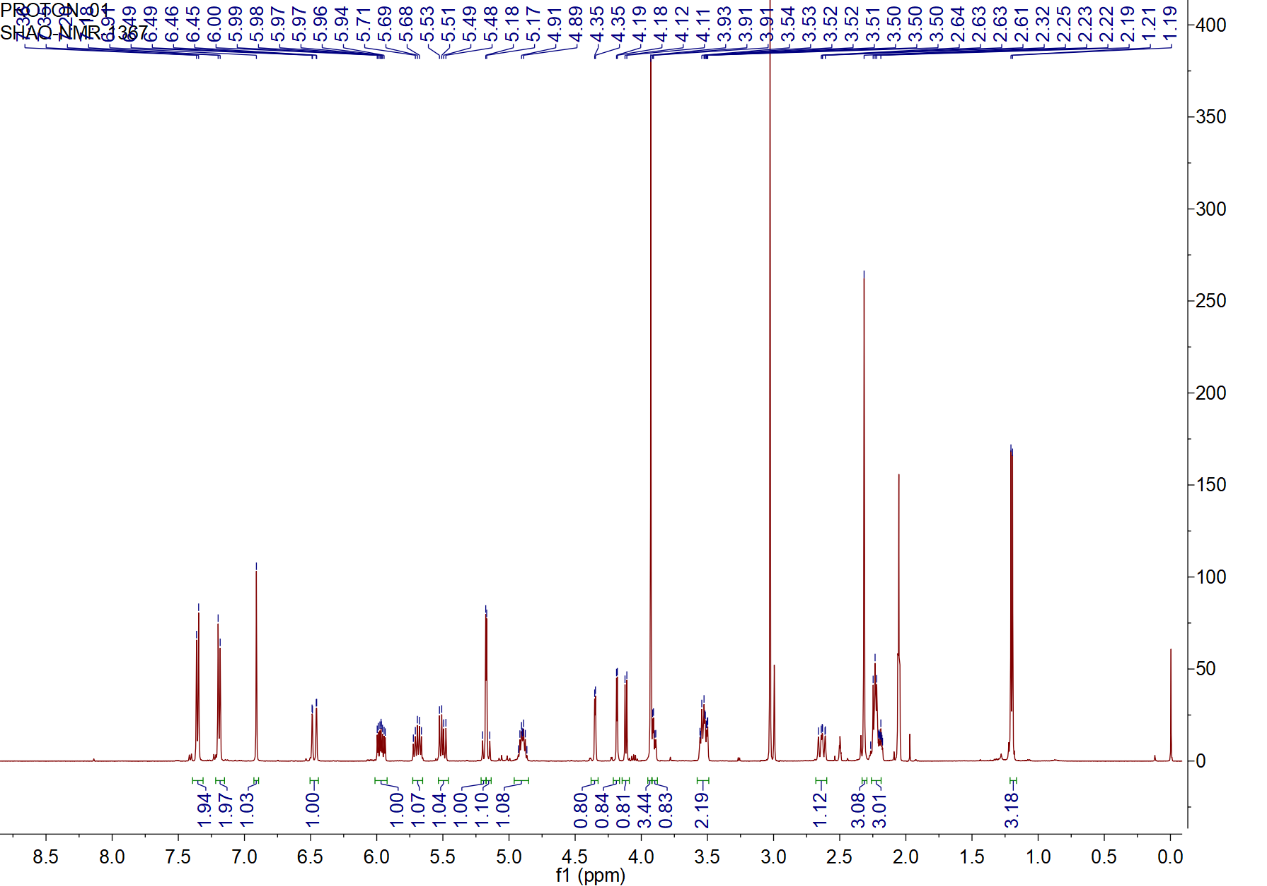


**Figure S19.** ^1^H NMR (500 MHz, CDCl_3_) spectrum of compound **8**


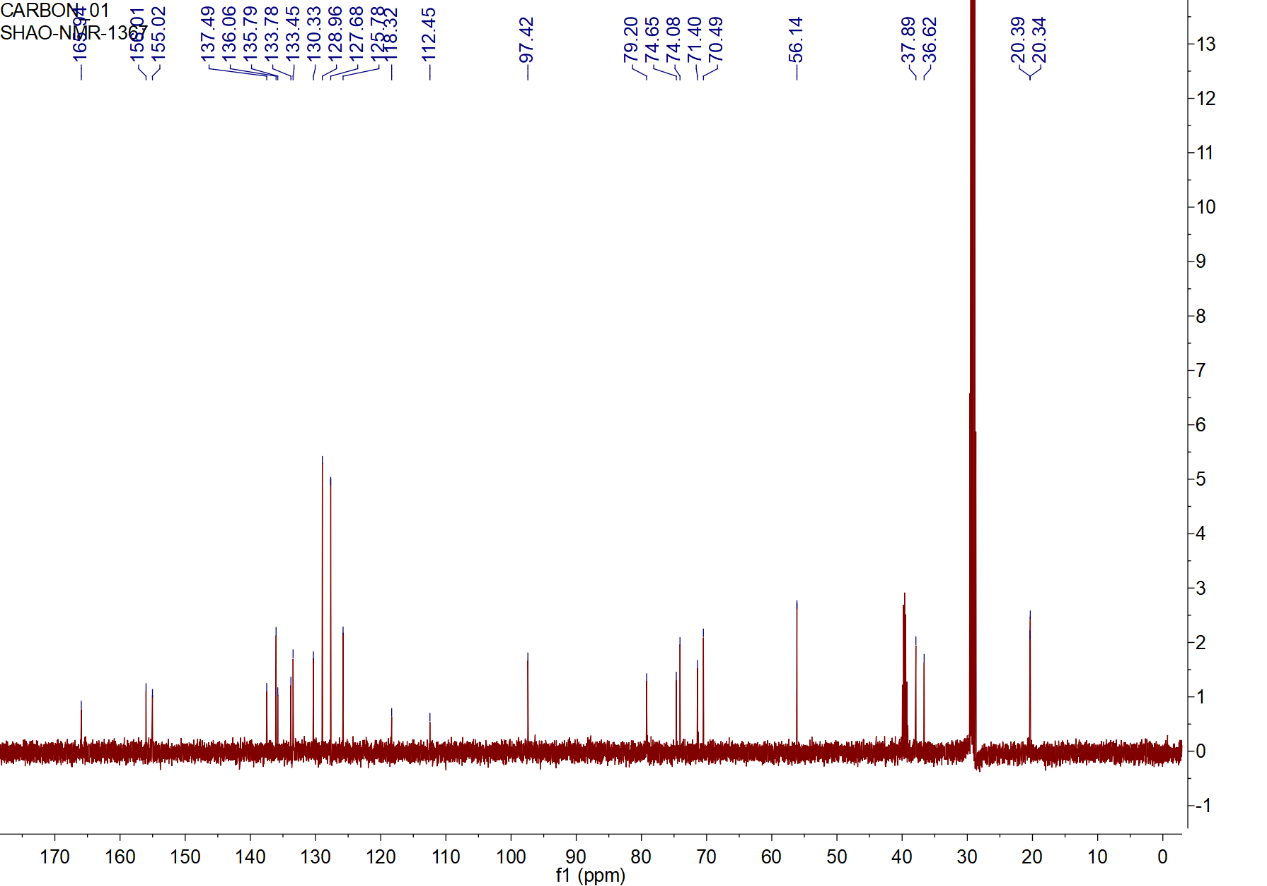


**Figure S20.** ^13^C NMR (125 MHz, CDCl_3_) spectrum of compound **8**

**Figure S21.** HRESIMS spectrum of compound **8**


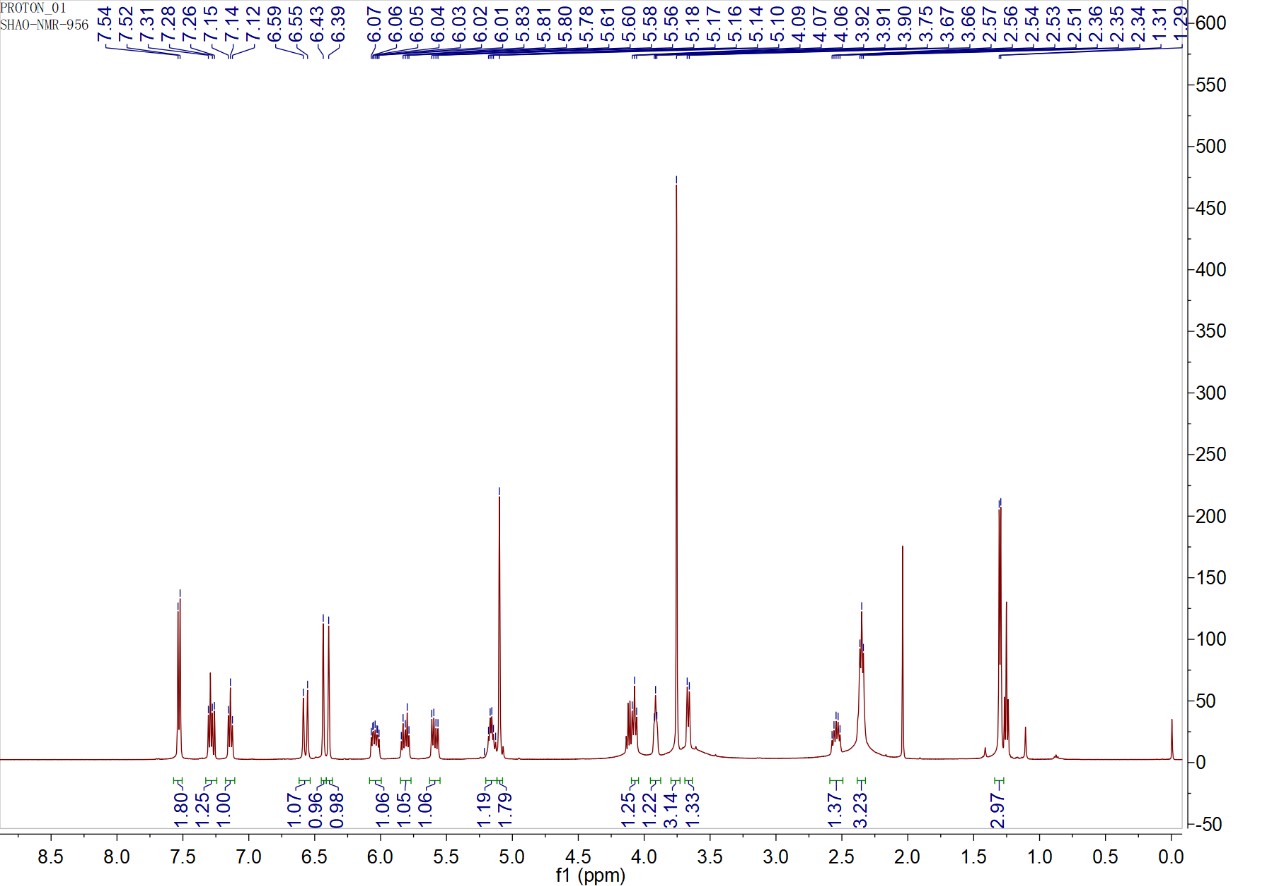


**Figure S22.** ^1^H NMR (500 MHz, CDCl_3_) spectrum of compound **9**


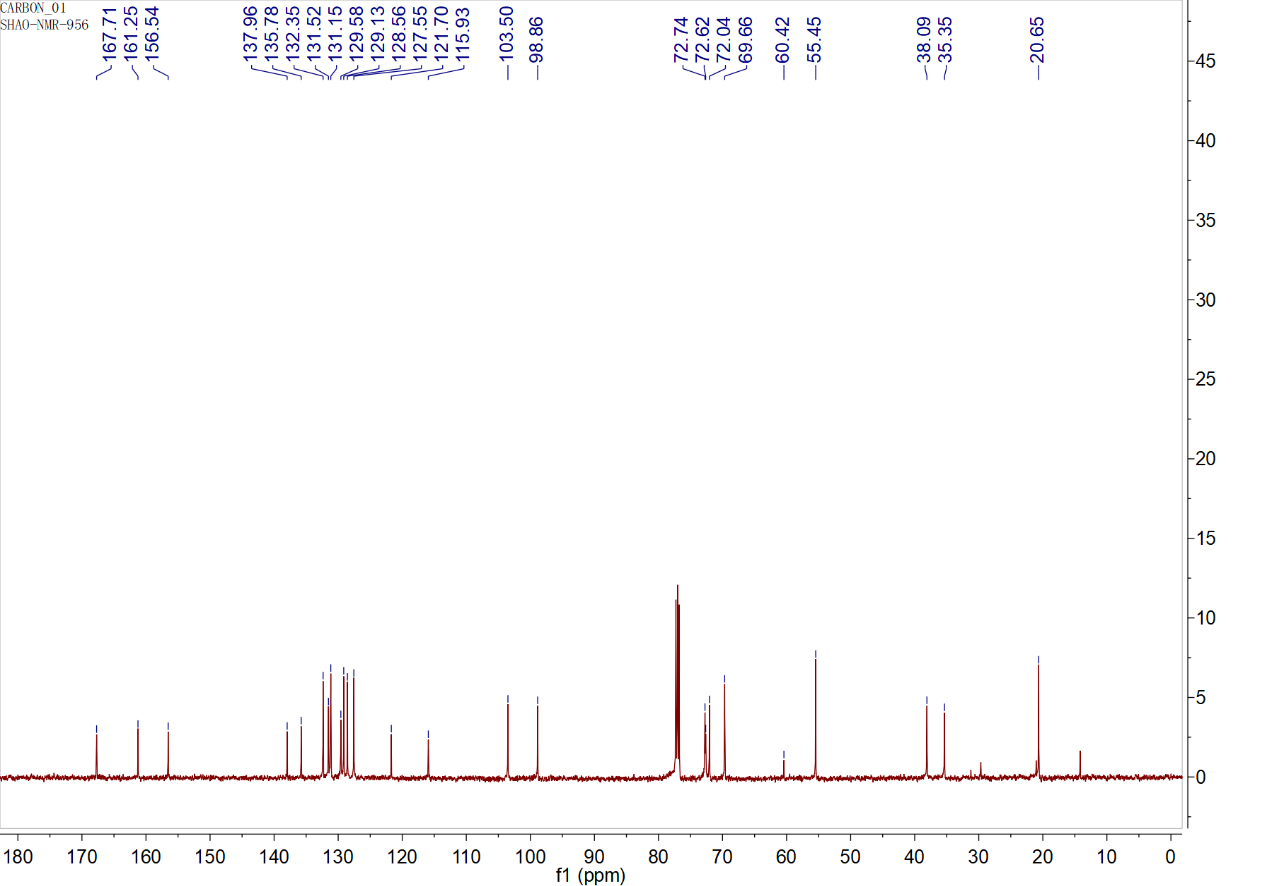


**Figure S23.** ^13^C NMR (125 MHz, CDCl_3_) spectrum of compound **9**

**Figure S24.** HRESIMS spectrum of compound **9**


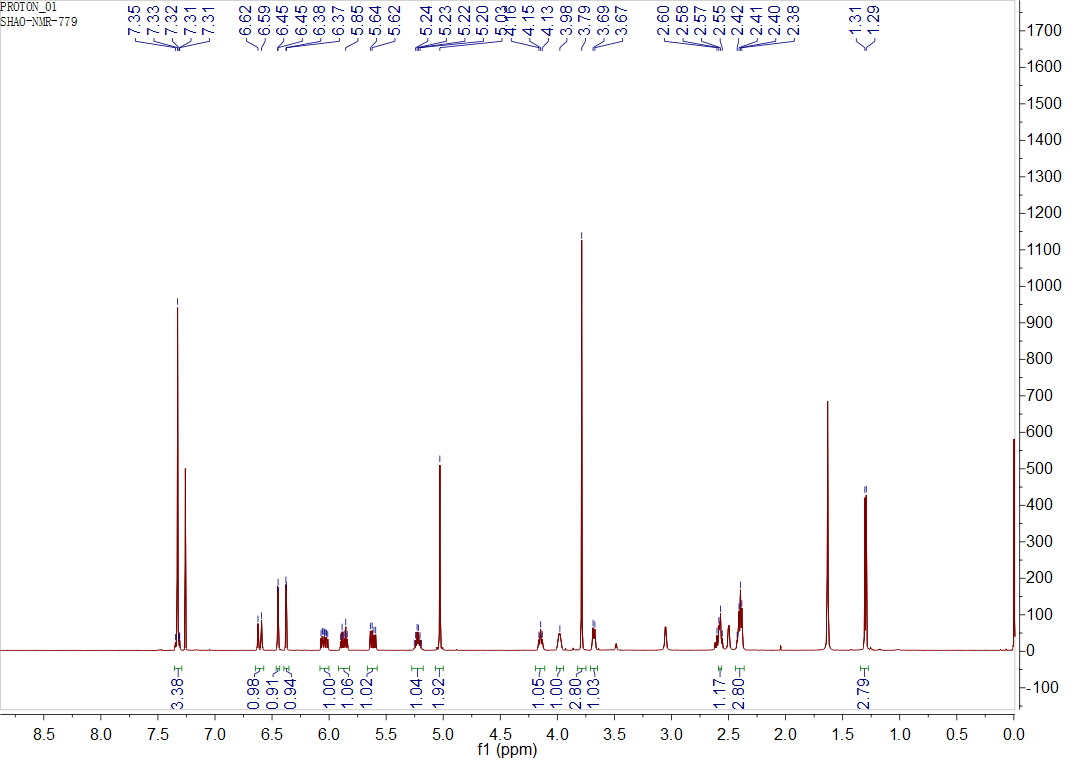


**Figure S25.** ^1^H NMR (500 MHz, CDCl_3_) spectrum of compound **10**


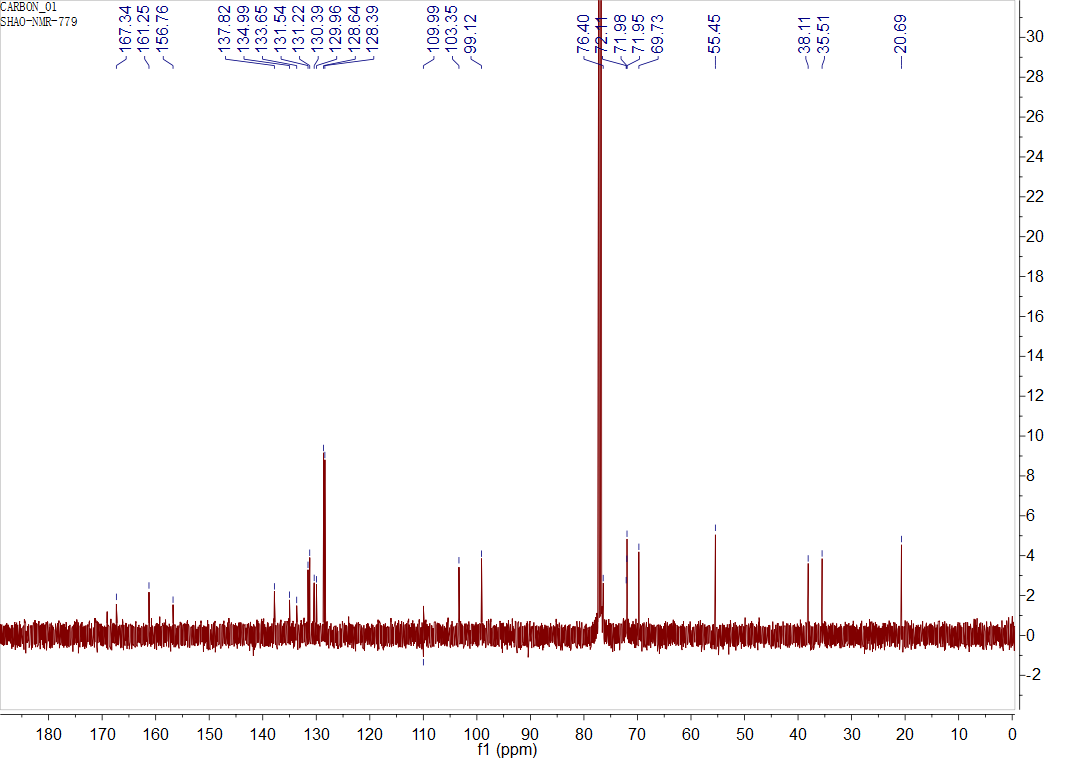


**Figure S26.** ^13^C NMR (125 MHz, CDCl_3_) spectrum of compound **10**.

**Figure S27.** HRESIMS spectrum of compound **10**


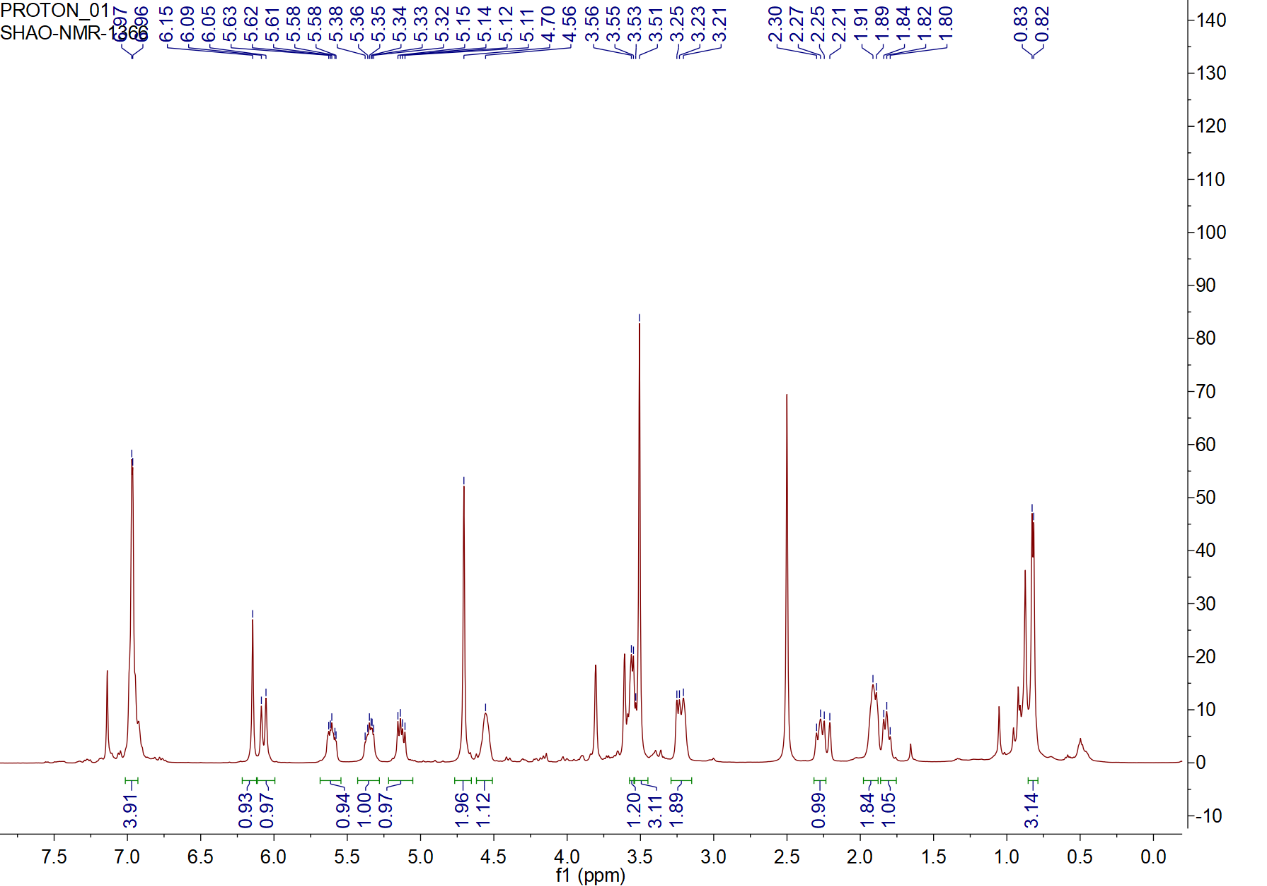


**Figure S28.** ^1^H NMR (500 MHz, CDCl_3_) spectrum of compound **11**


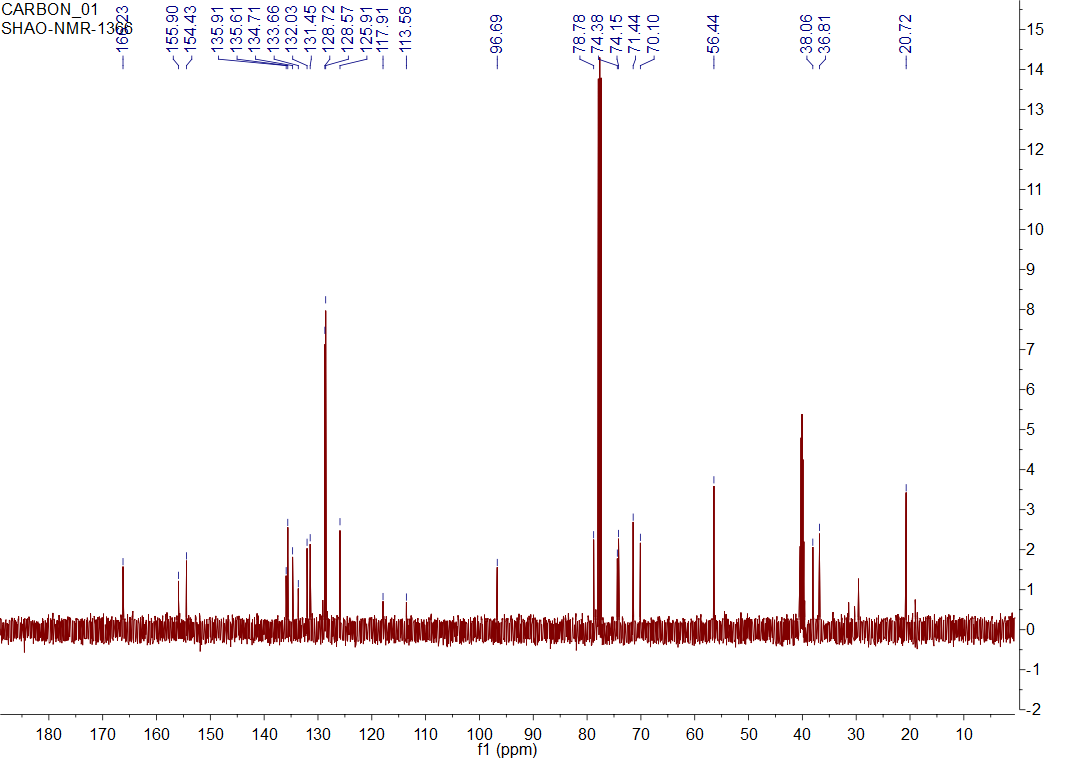


**Figure S29.** ^13^C NMR (125 MHz, CDCl_3_) spectrum of compound **11**

**Figure S30.** HRESIMS spectrum of compound **11**


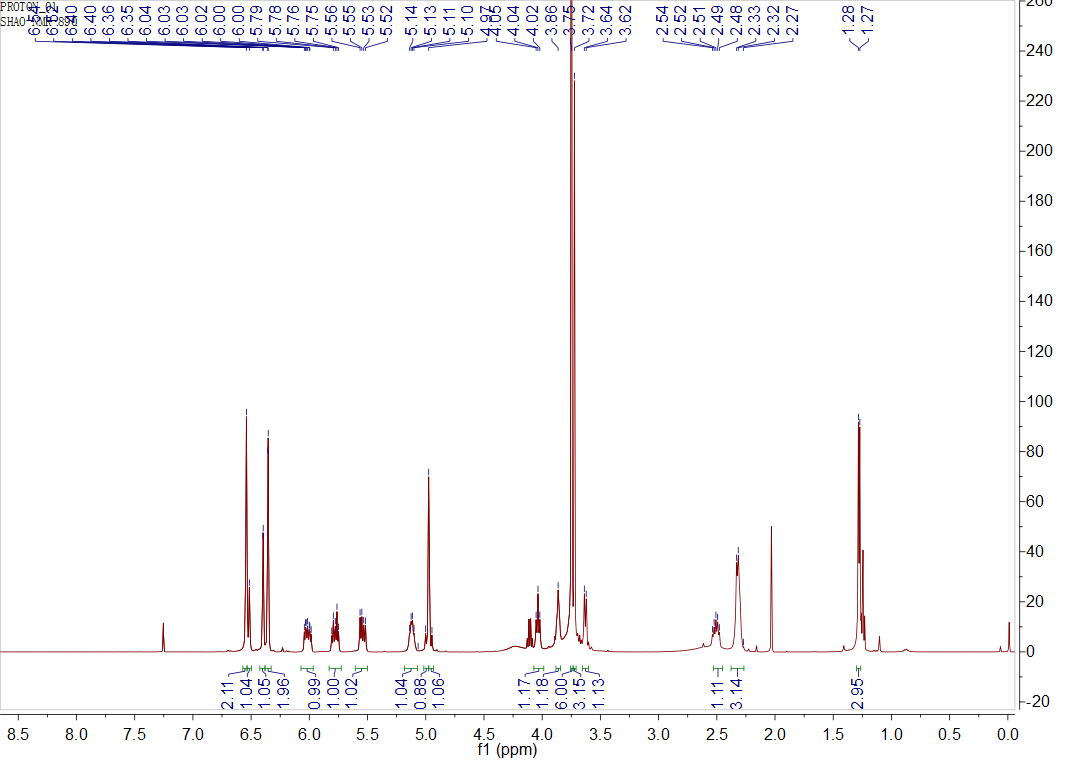


**Figure S31.** ^1^H NMR (500 MHz, CDCl_3_) spectrum of compound **12**


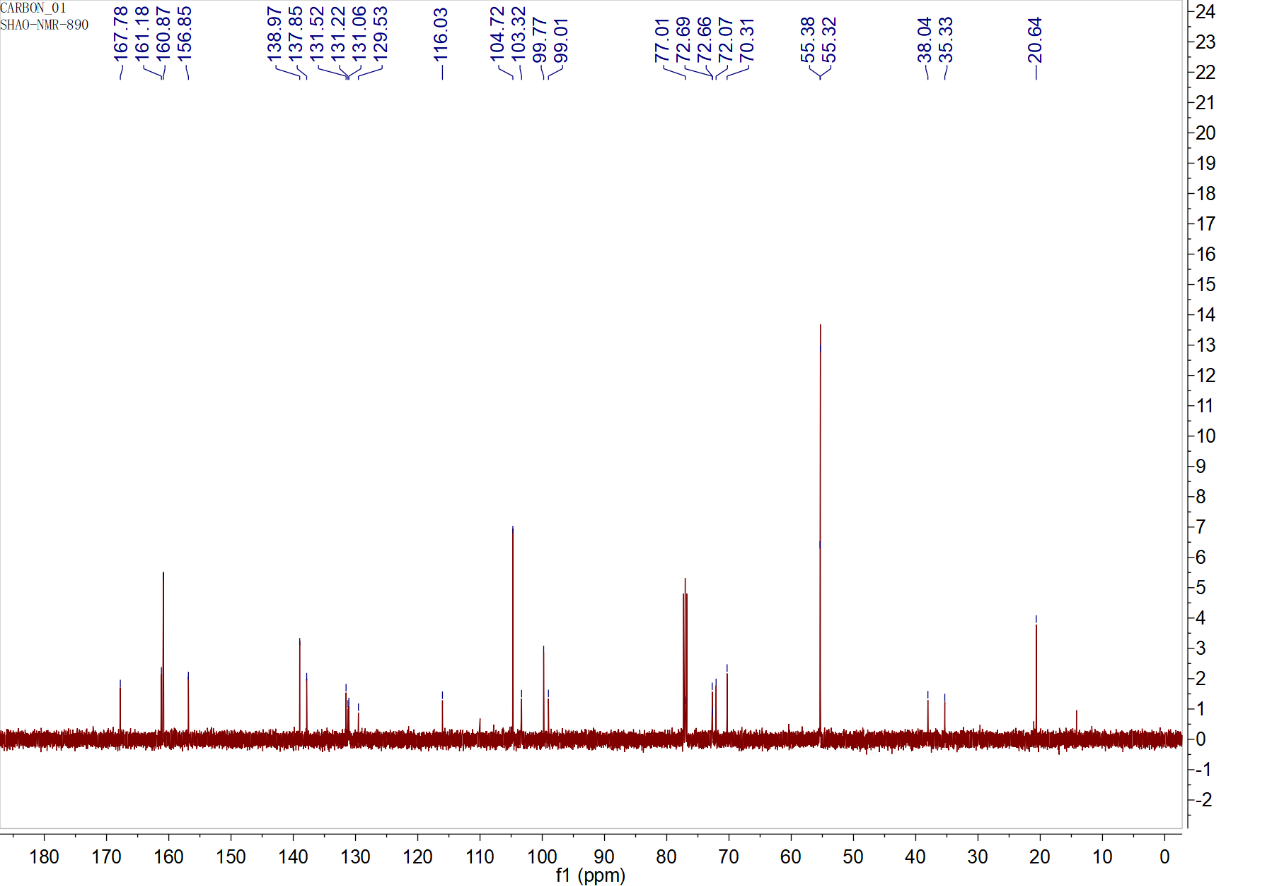


**Figure S32.** ^13^C NMR (125 MHz, CDCl_3_) spectrum of compound **12**

**Figure S33.** HRESIMS spectrum of compound **12**


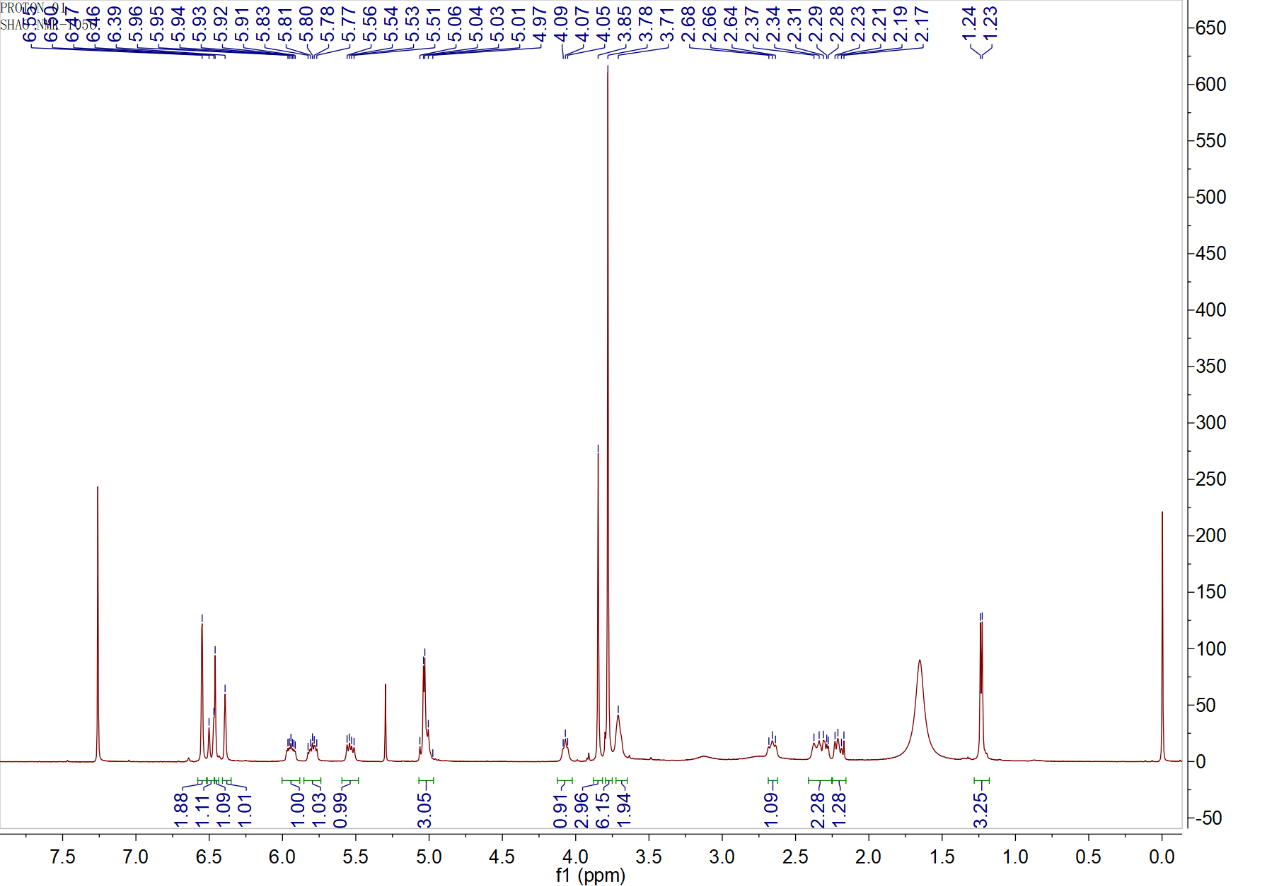


**Figure S34.** ^1^H NMR (500 MHz, CDCl_3_) spectrum of compound **13**


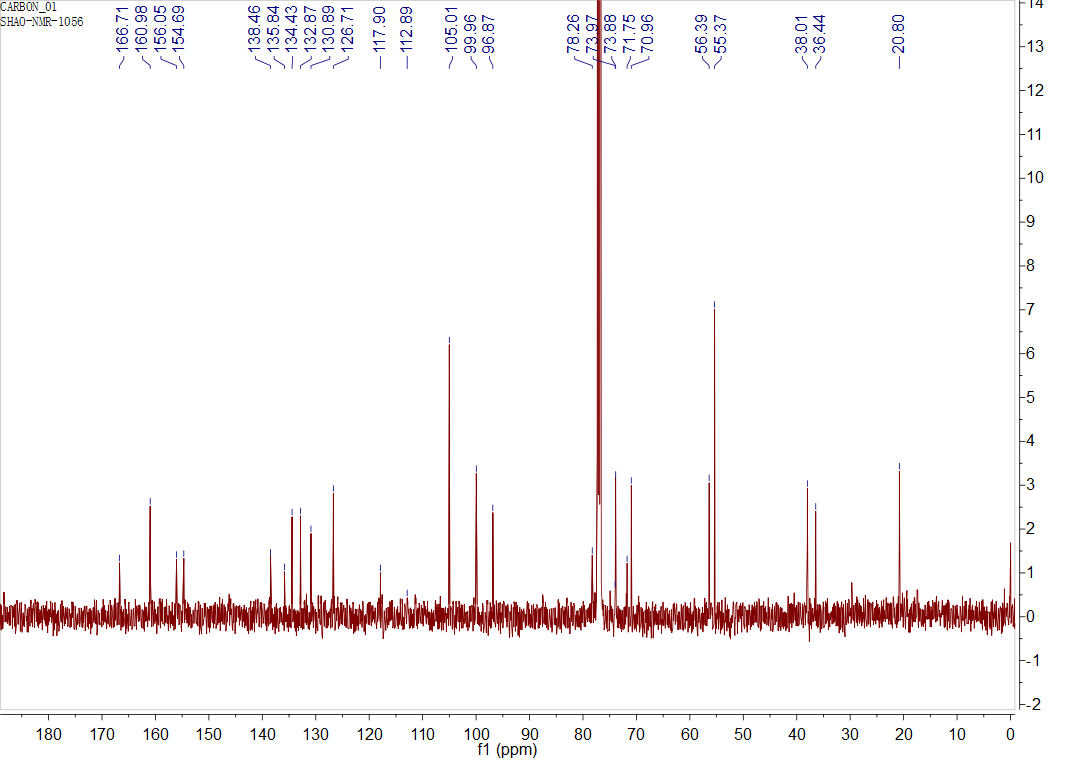


**Figure S35.** ^13^C NMR (125 MHz, CDCl_3_) spectrum of compound **13**

**Figure S36.** HRESIMS spectrum of compound **13**


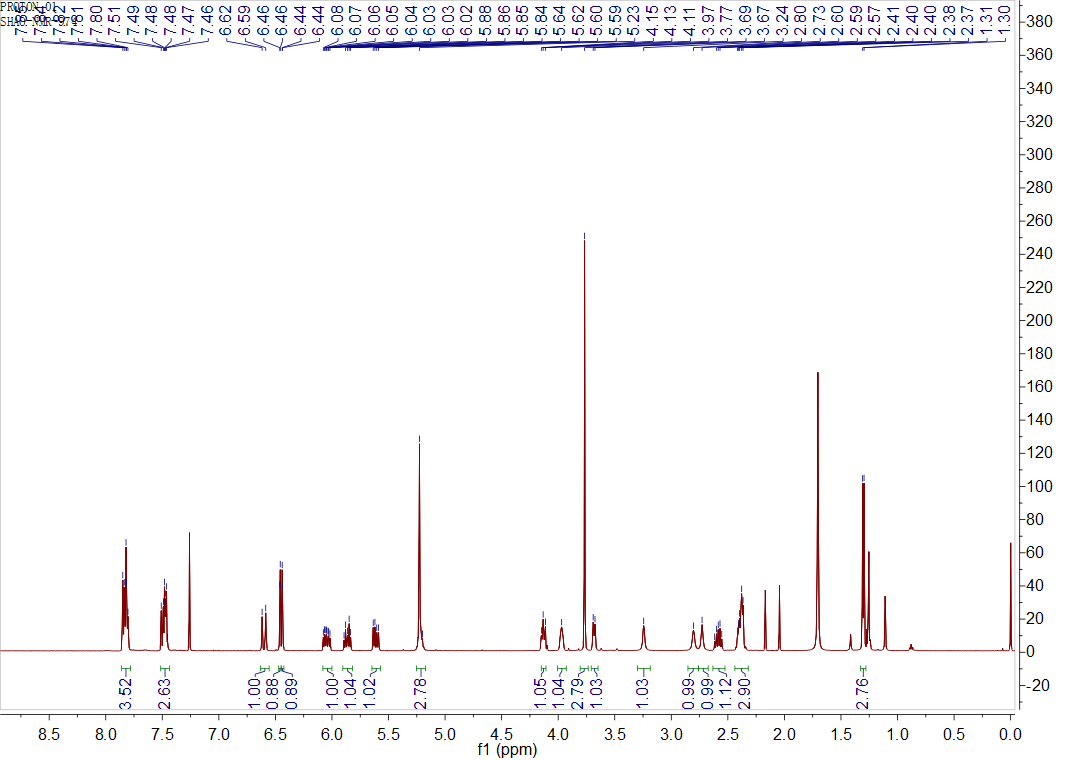


**Figure S37.** ^1^H NMR (500 MHz, CDCl_3_) spectrum of compound **14**


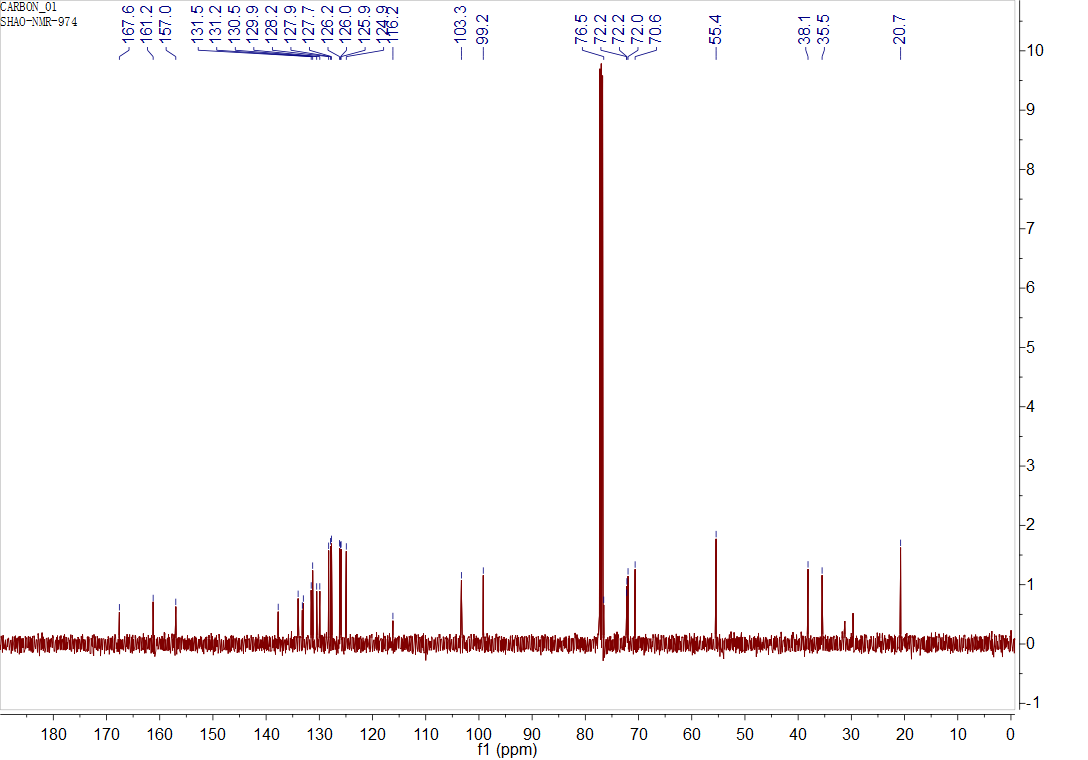


**Figure S38.** ^13^C NMR (125 MHz, CDCl_3_) spectrum of compound **14**

**Figure S39.** HRESIMS spectrum of compound **14**


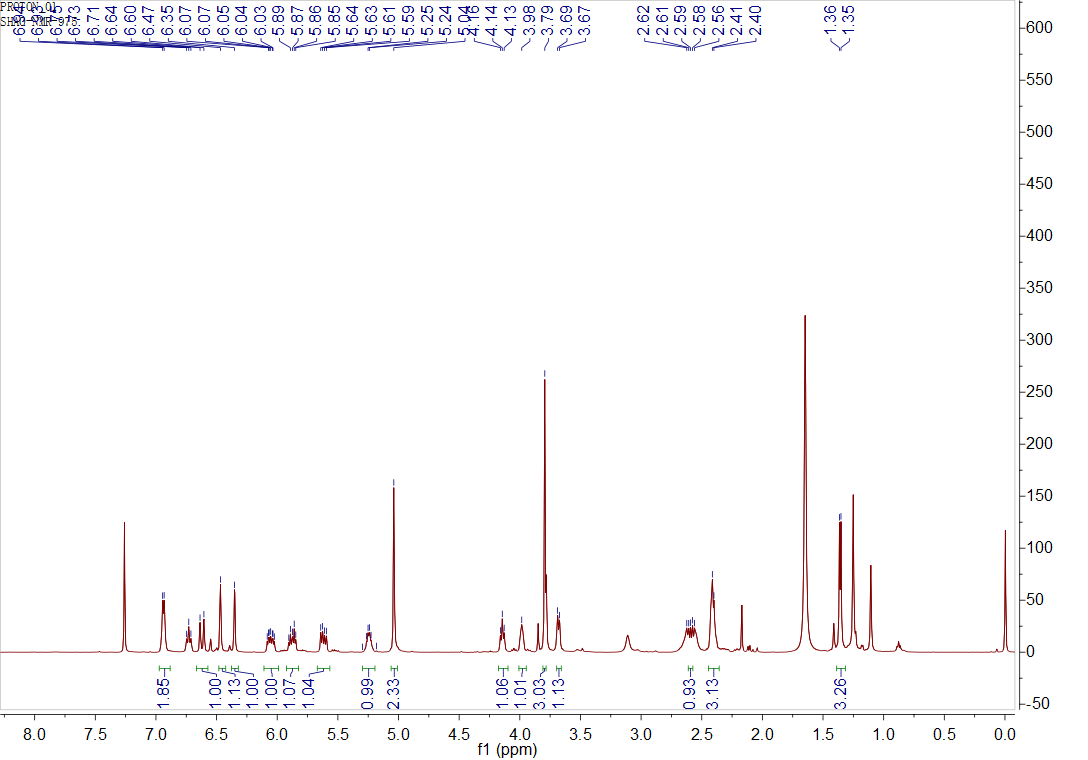


**Figure S40.** ^1^H NMR (500 MHz, CDCl_3_) spectrum of compound **15**


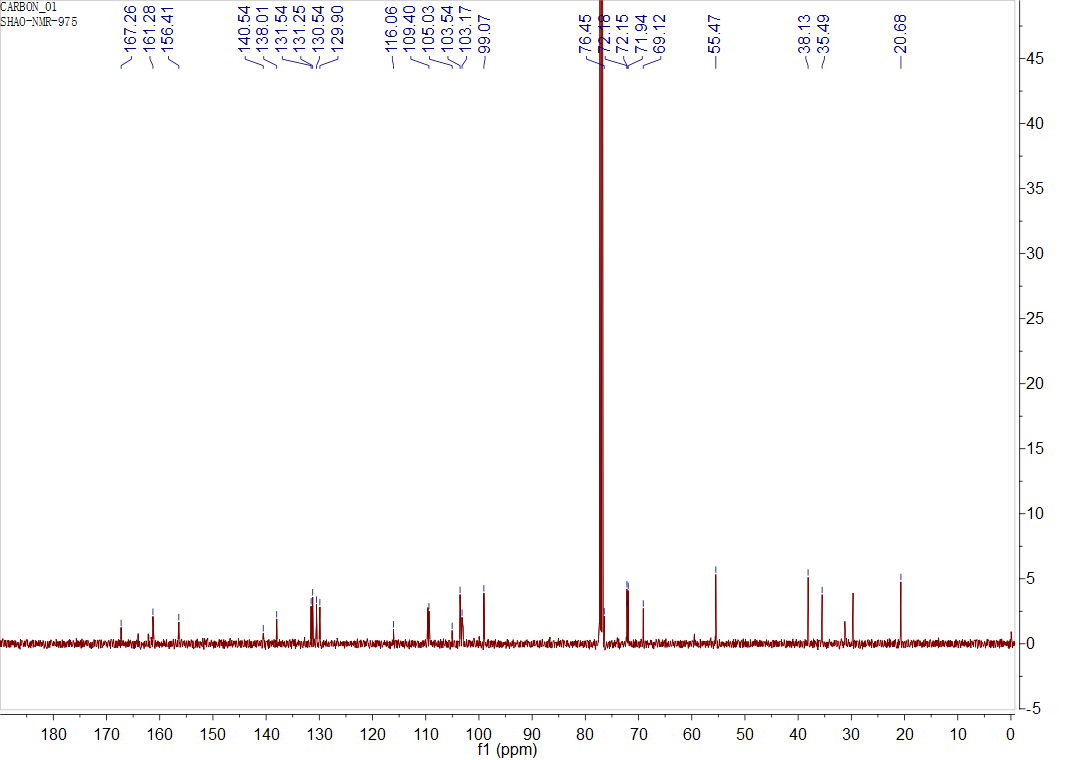


**Figure S41.** ^13^C NMR (125 MHz, CDCl_3_) spectrum of compound **15**

**Figure S42.** HRESIMS spectrum of compound **15**


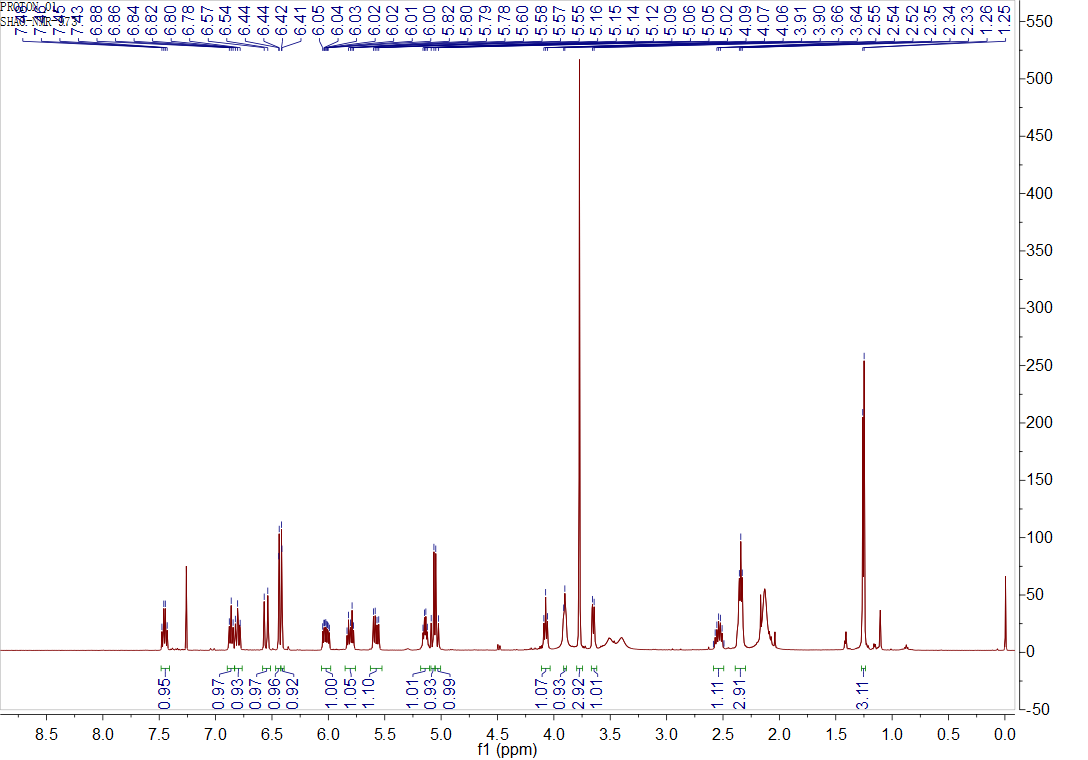


**Figure S43.** ^1^H NMR (500 MHz, CDCl_3_) spectrum of compound **16**


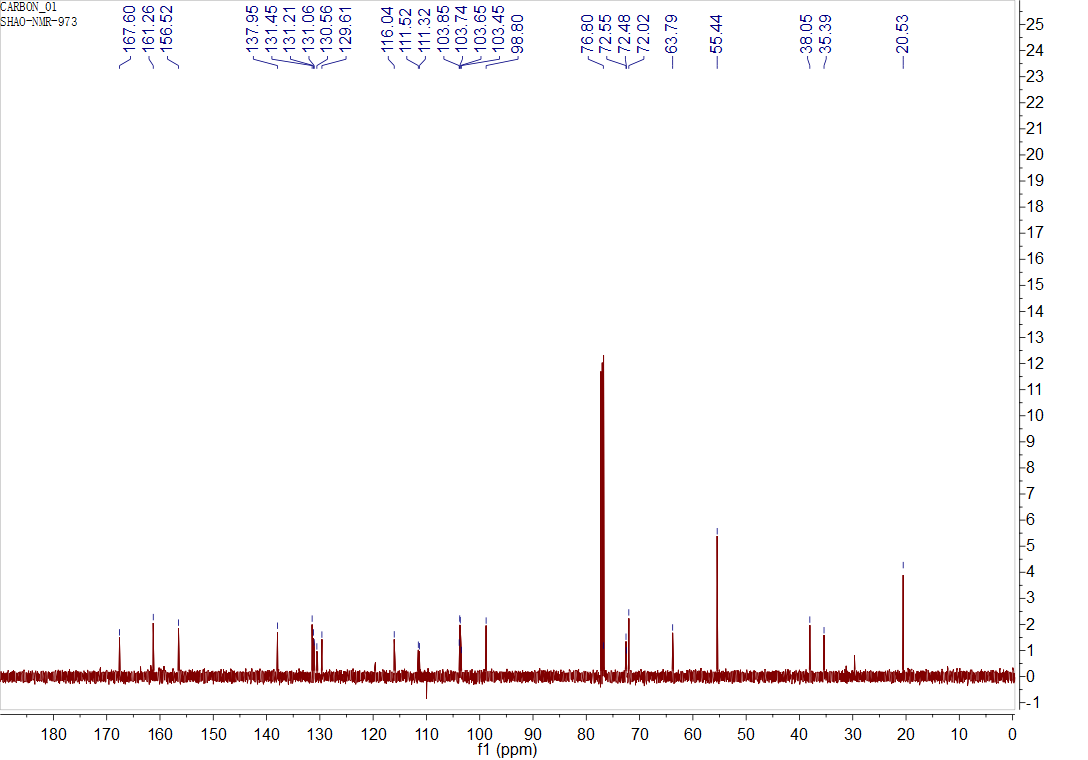


**Figure S44.** ^13^C NMR (125 MHz, CDCl_3_) spectrum of compound **16**

**Figure S45.** HRESIMS spectrum of compound **16**


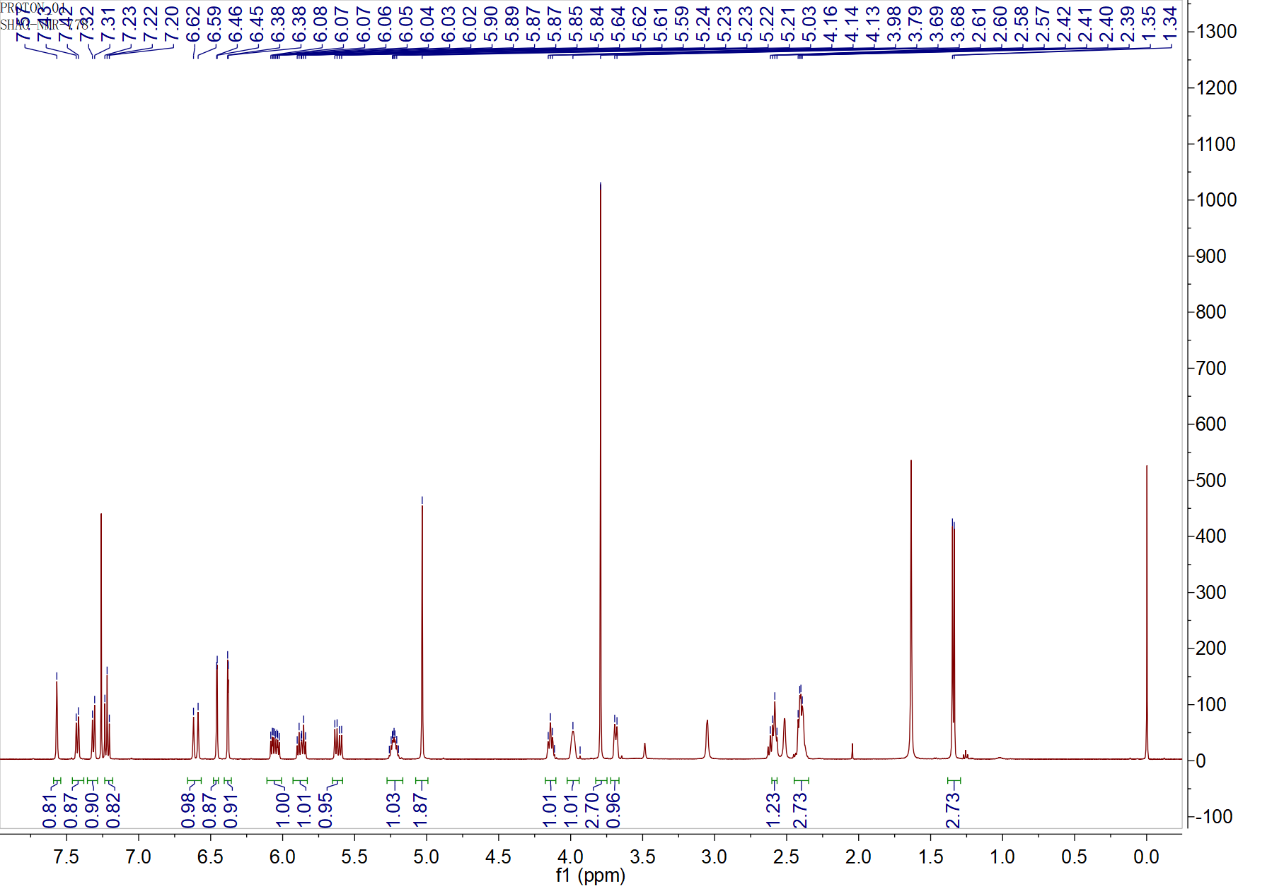


**Figure S46.** ^1^H NMR (500 MHz, CDCl_3_) spectrum of compound **17**


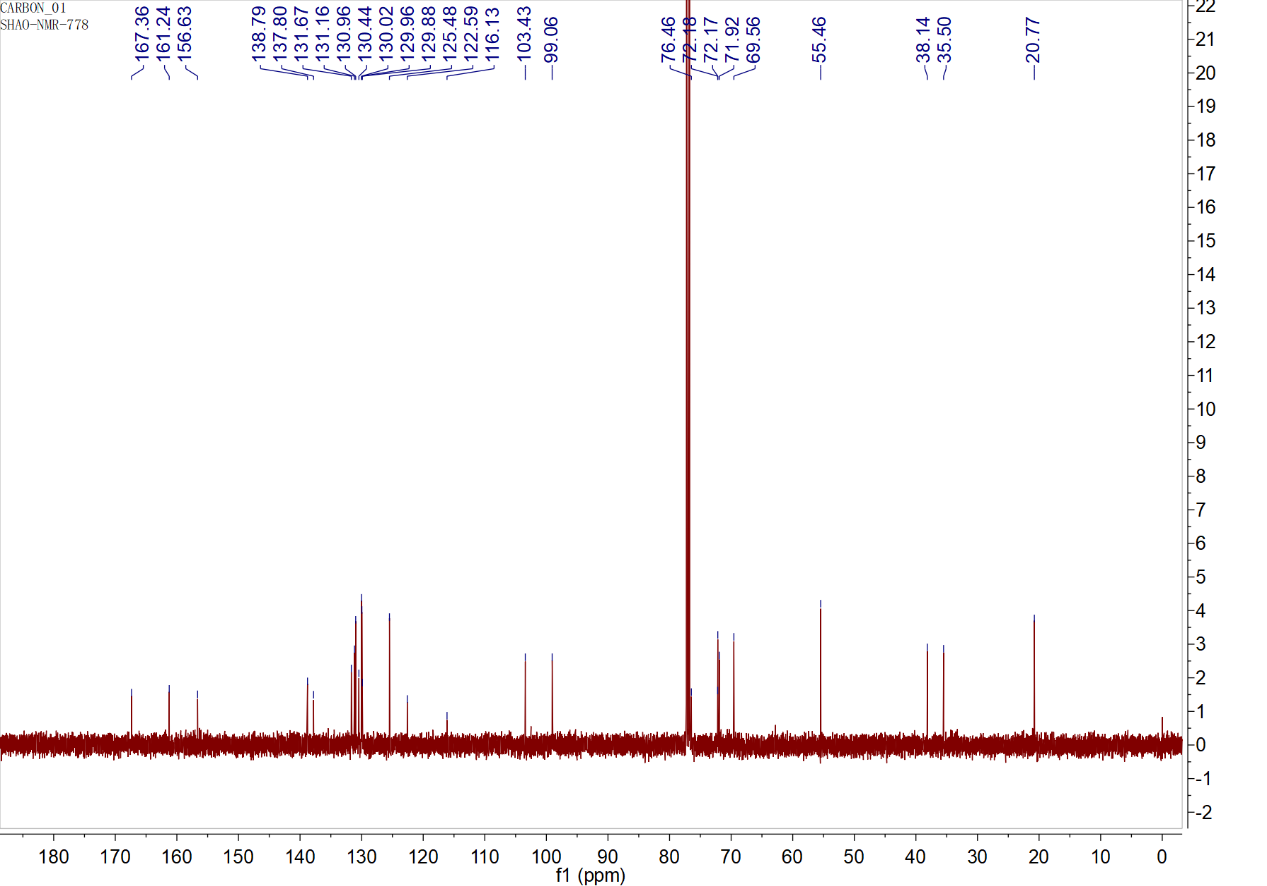


**Figure S47.** ^13^C NMR (125 MHz, CDCl_3_) spectrum of compound **17**

**Figure S48.** HRESIMS spectrum of compound **17**


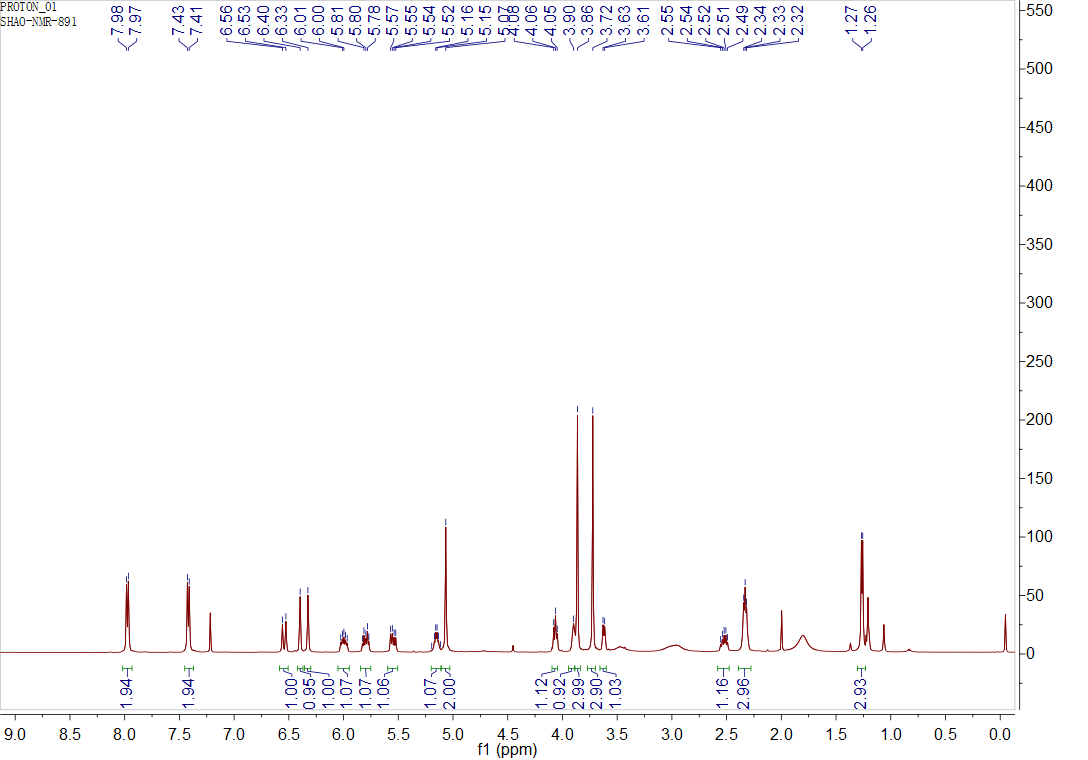


**Figure S49.** ^1^H NMR (500 MHz, CDCl_3_) spectrum of compound **18**


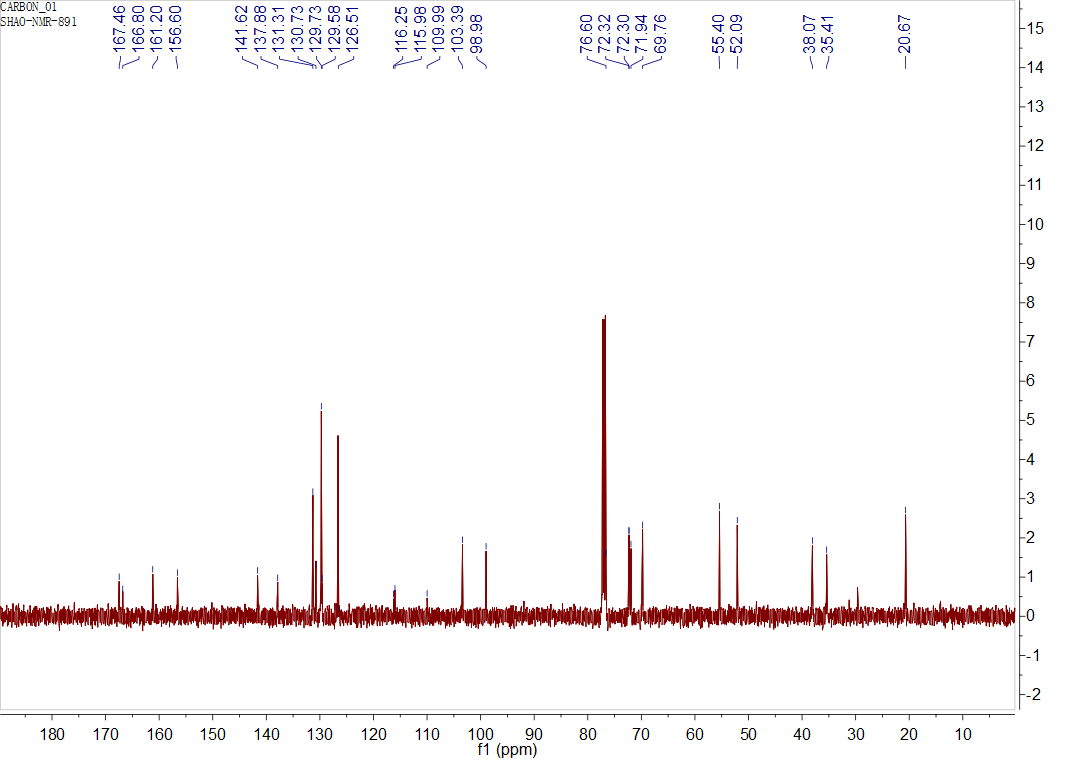


**Figure S50.** ^13^C NMR (125 MHz, CDCl_3_) spectrum of compound **18**

**Figure S51.** HRESIMS spectrum of compound **18**


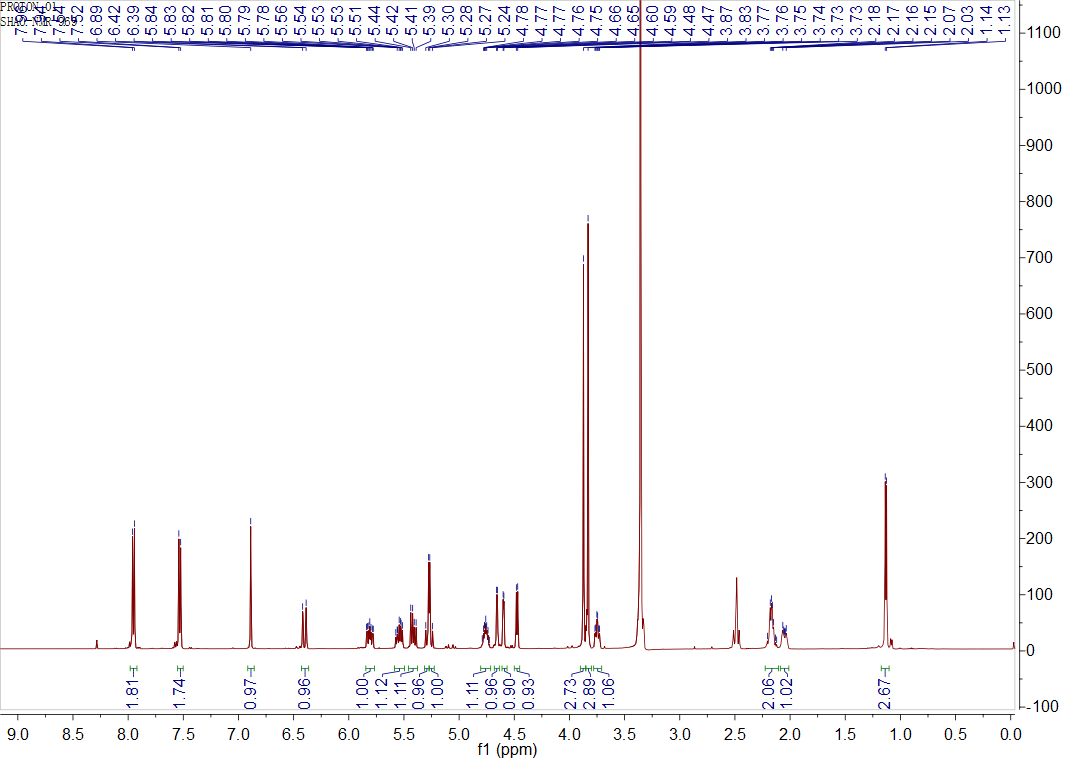


**Figure S52.** ^1^H NMR (500 MHz, CDCl_3_) spectrum of compound **19**


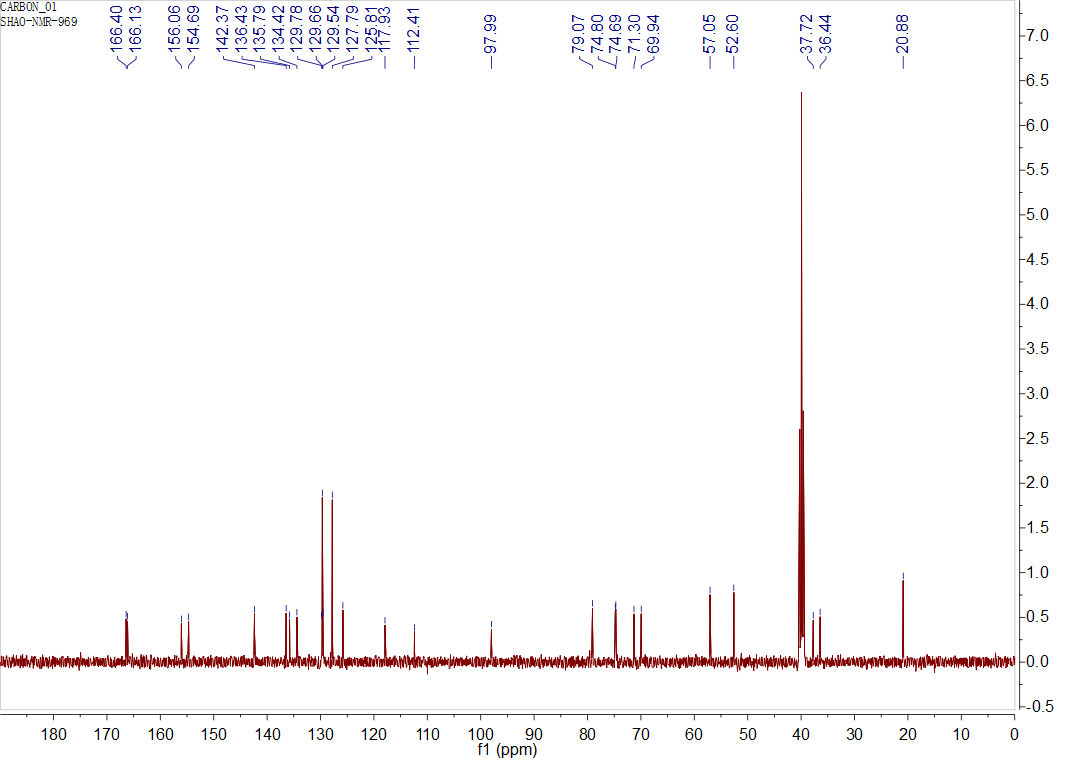


**Figure S53.** ^13^C NMR (125 MHz, CDCl_3_) spectrum of compound **19**

**Figure S54.** HRESIMS spectrum of compound **19**


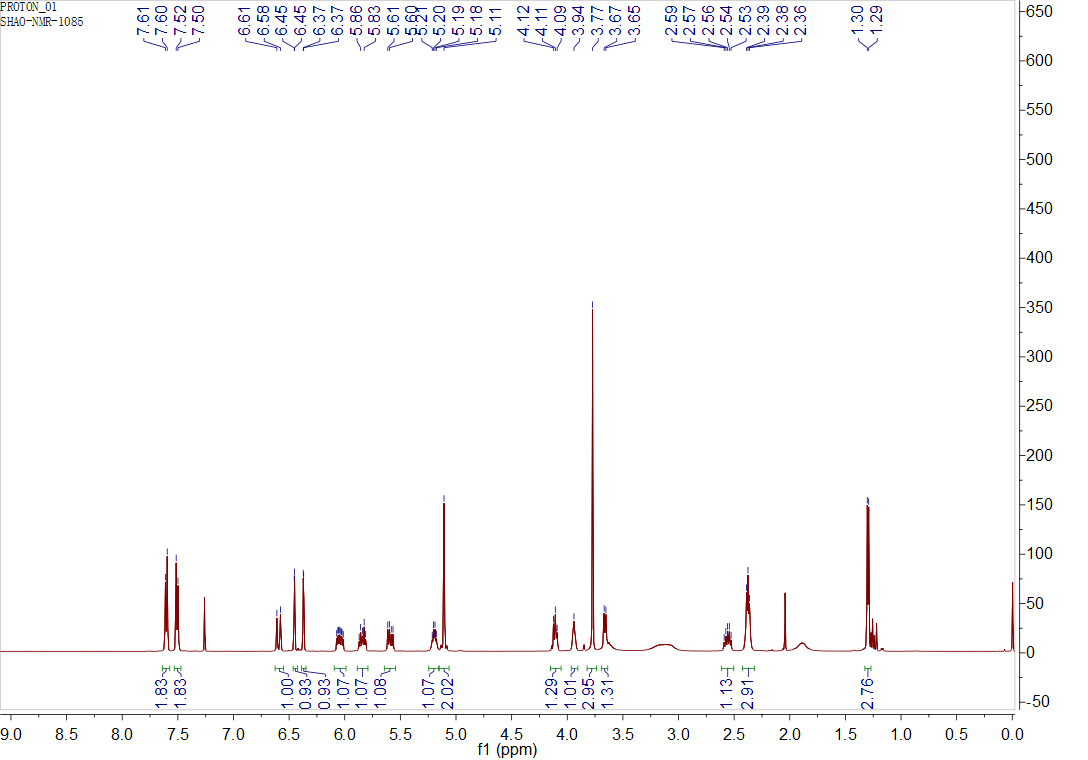


**Figure S55.** ^1^H NMR (500 MHz, CDCl_3_) spectrum of compound **20**


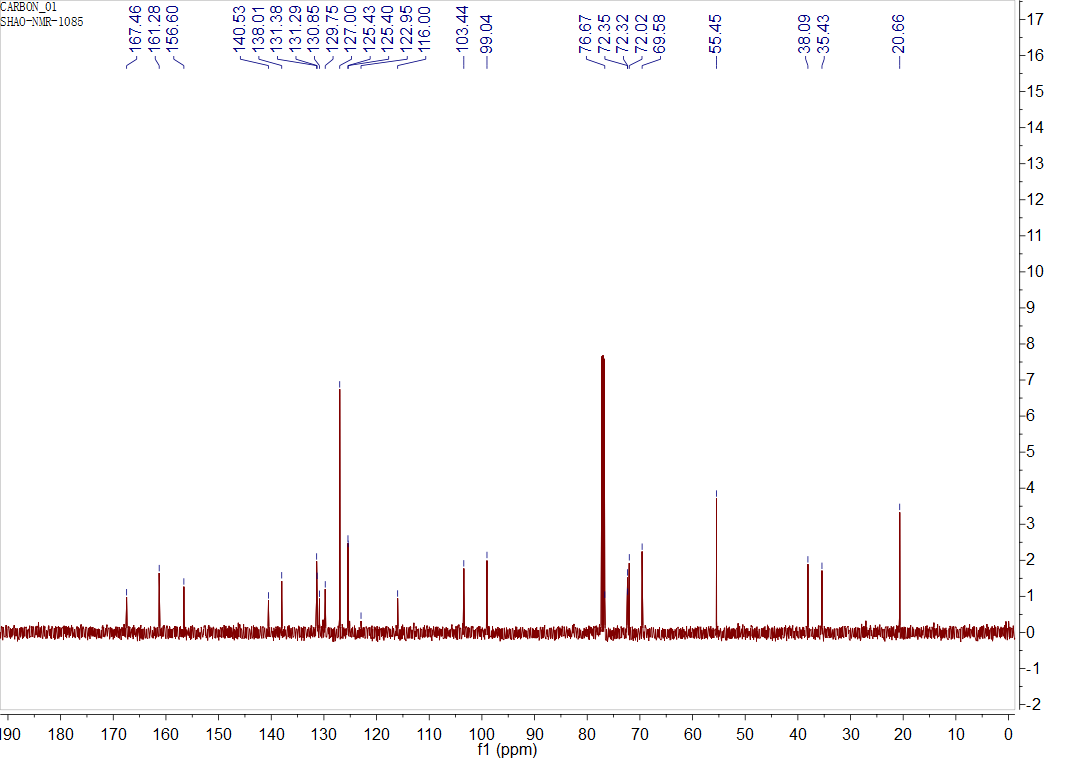


**Figure S56.** ^13^C NMR (125 MHz, CDCl_3_) spectrum of compound **20**

**Figure S57.** HRESIMS spectrum of compound **20**


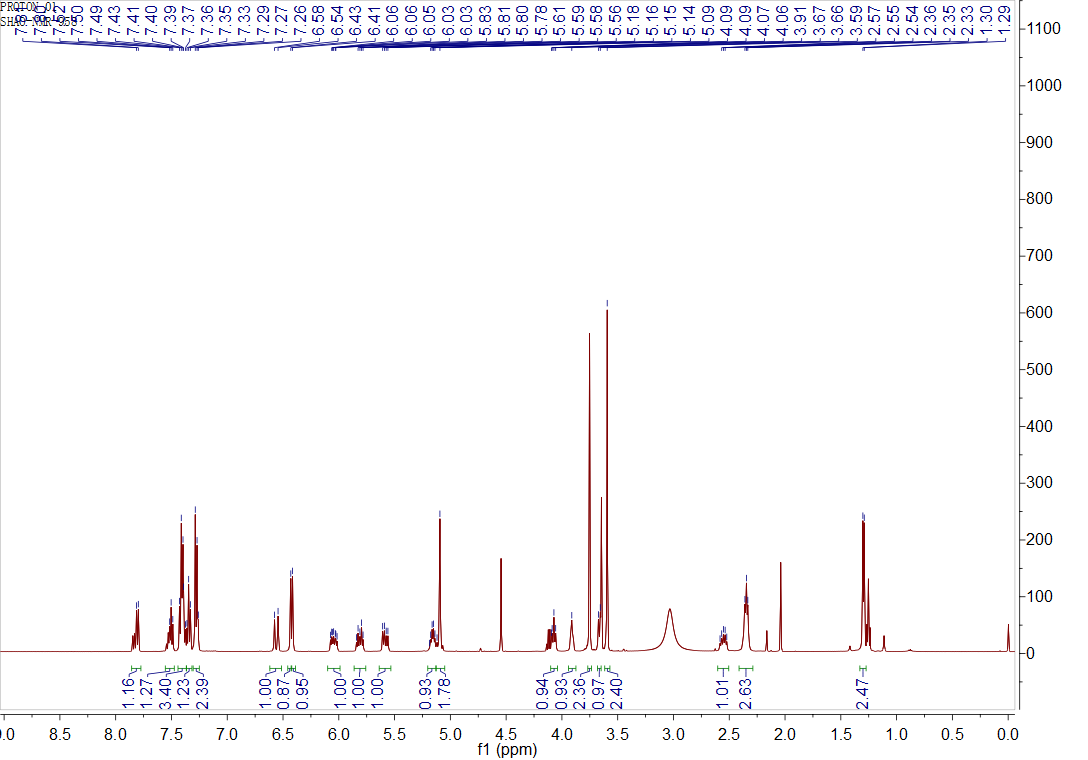


**Figure S58.** ^1^H NMR (500 MHz, CDCl_3_) spectrum of compound **21**


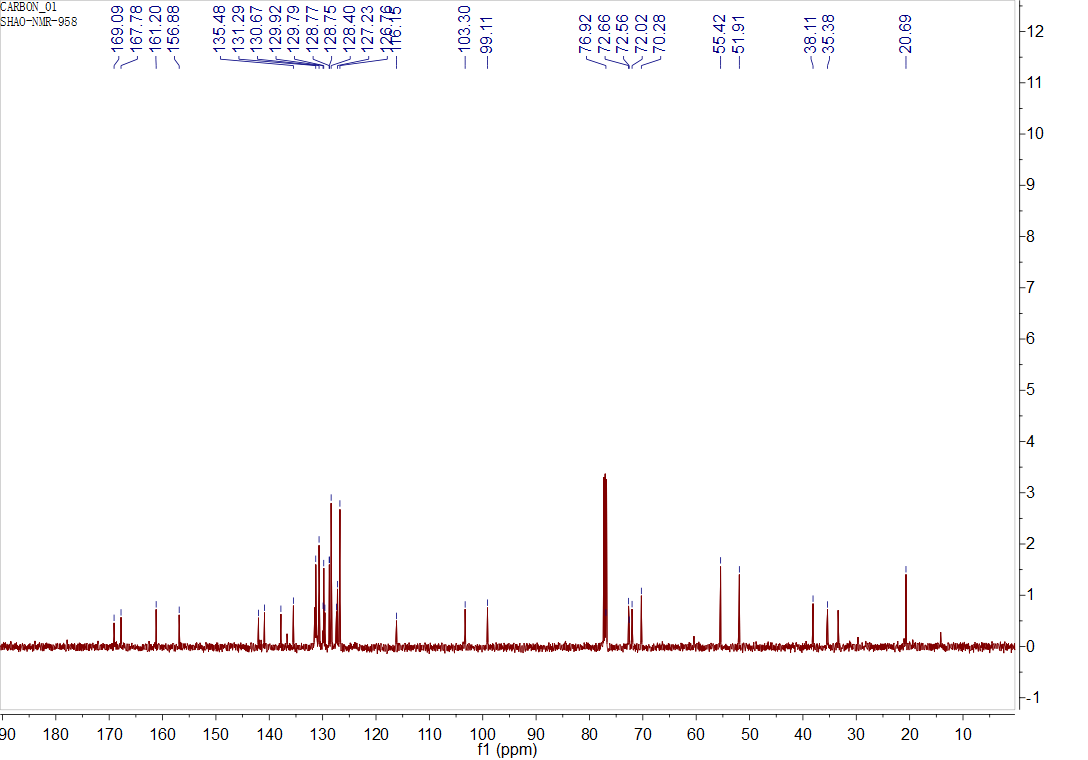


**Figure S59.** ^13^C NMR (125 MHz, CDCl_3_) spectrum of compound **21**

**Figure S60.** HRESIMS spectrum of compound **21**


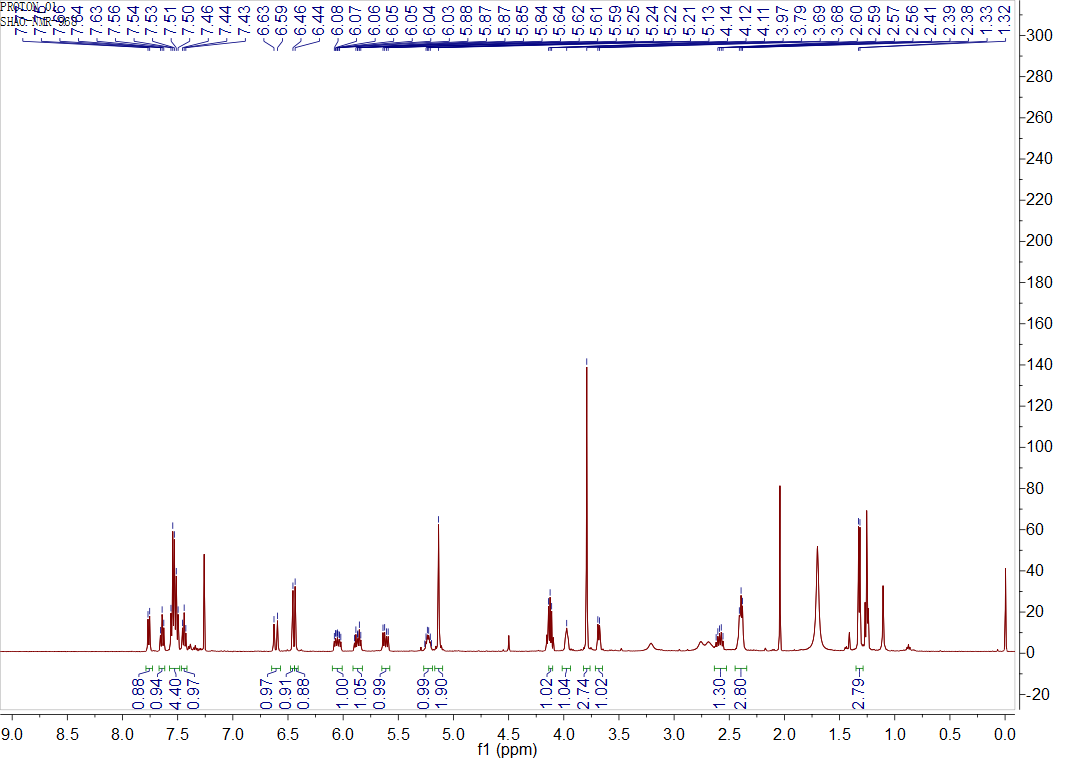


**Figure S61.** ^1^H NMR (500 MHz, CDCl_3_) spectrum of compound **22**


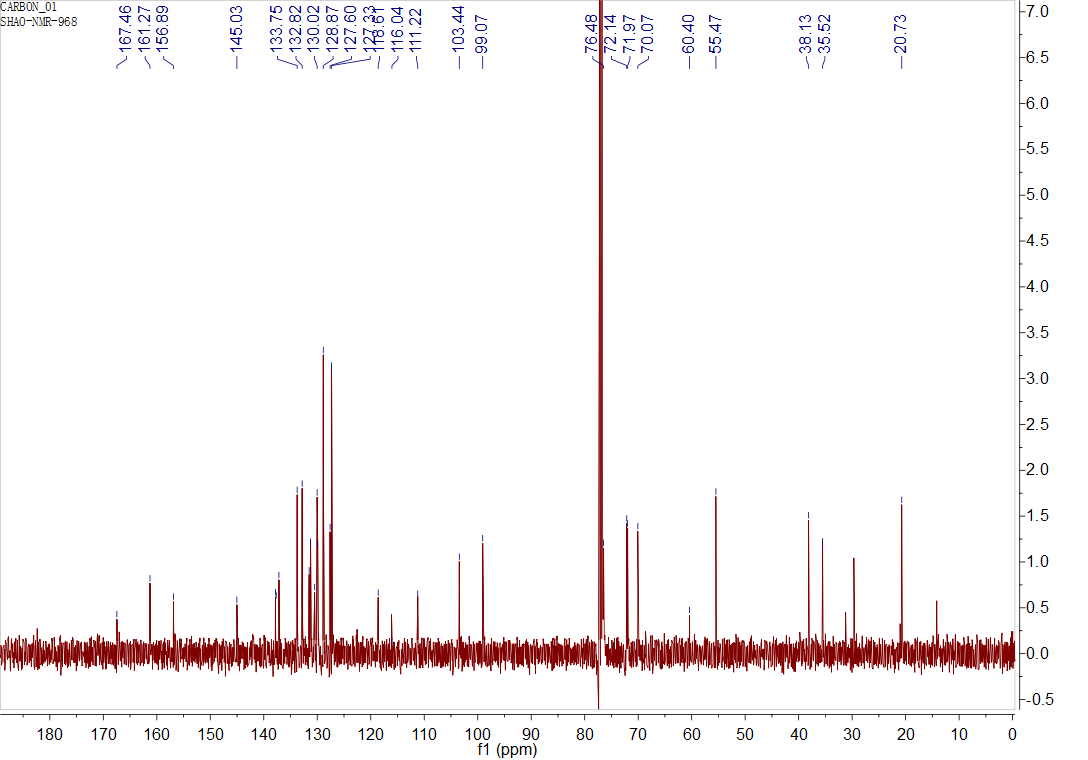


**Figure S62.** ^13^C NMR (125 MHz, CDCl_3_) spectrum of compound **22**

**Figure S63.** HRESIMS spectrum of compound **22**


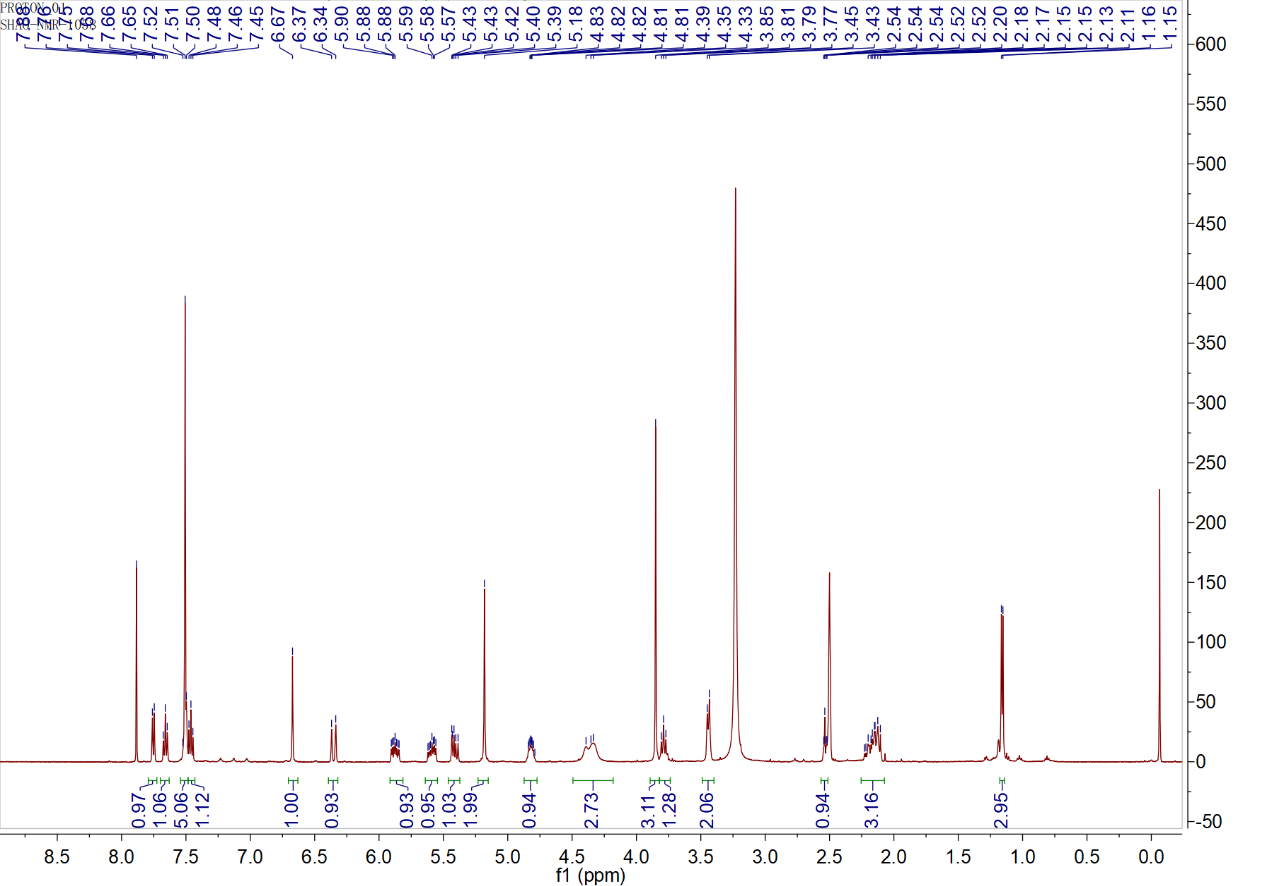


**Figure S64.** ^1^H NMR (500 MHz, CDCl_3_) spectrum of compound **23**


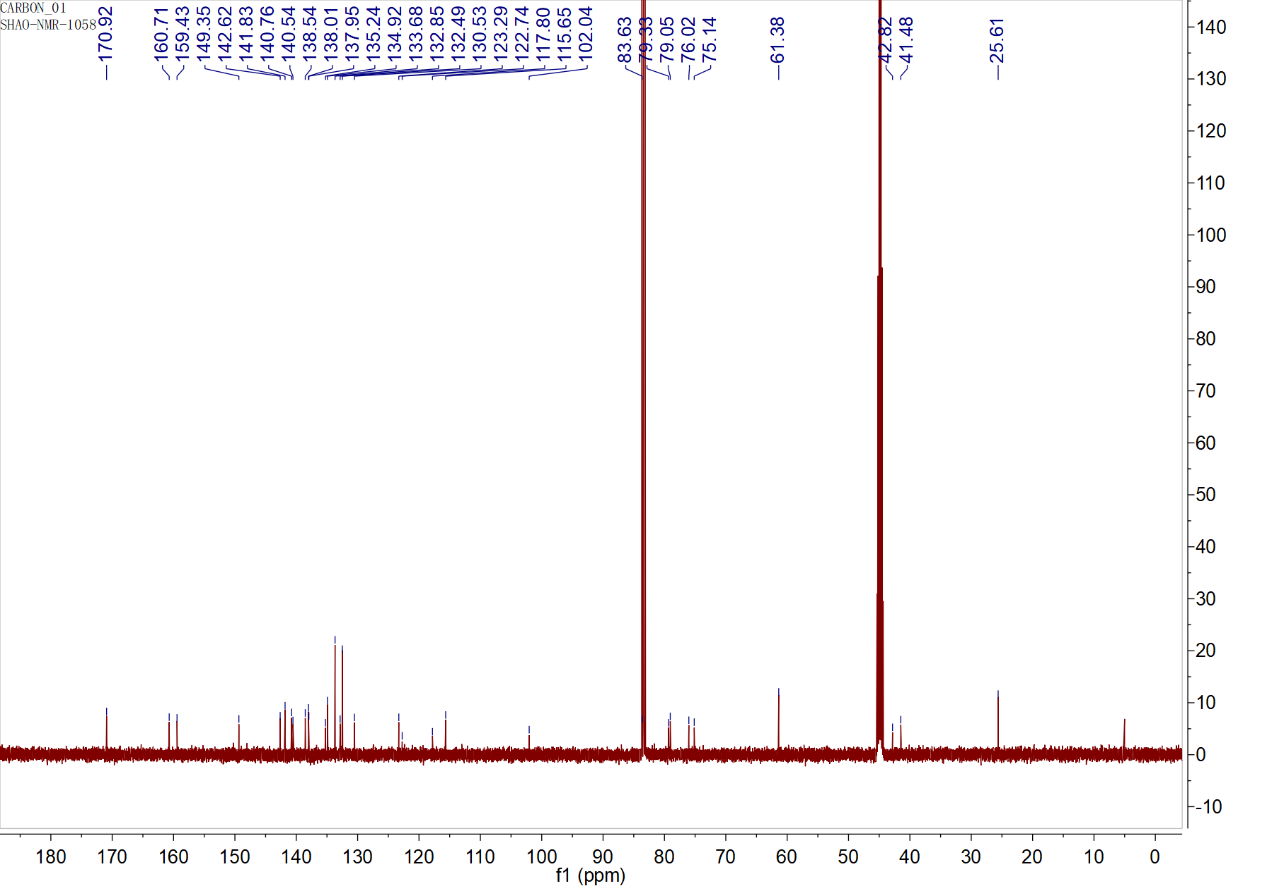


**Figure S65.** ^13^C NMR (125 MHz, CDCl_3_) spectrum of compound **23**

**Figure S66.** HRESIMS spectrum of compound **23**


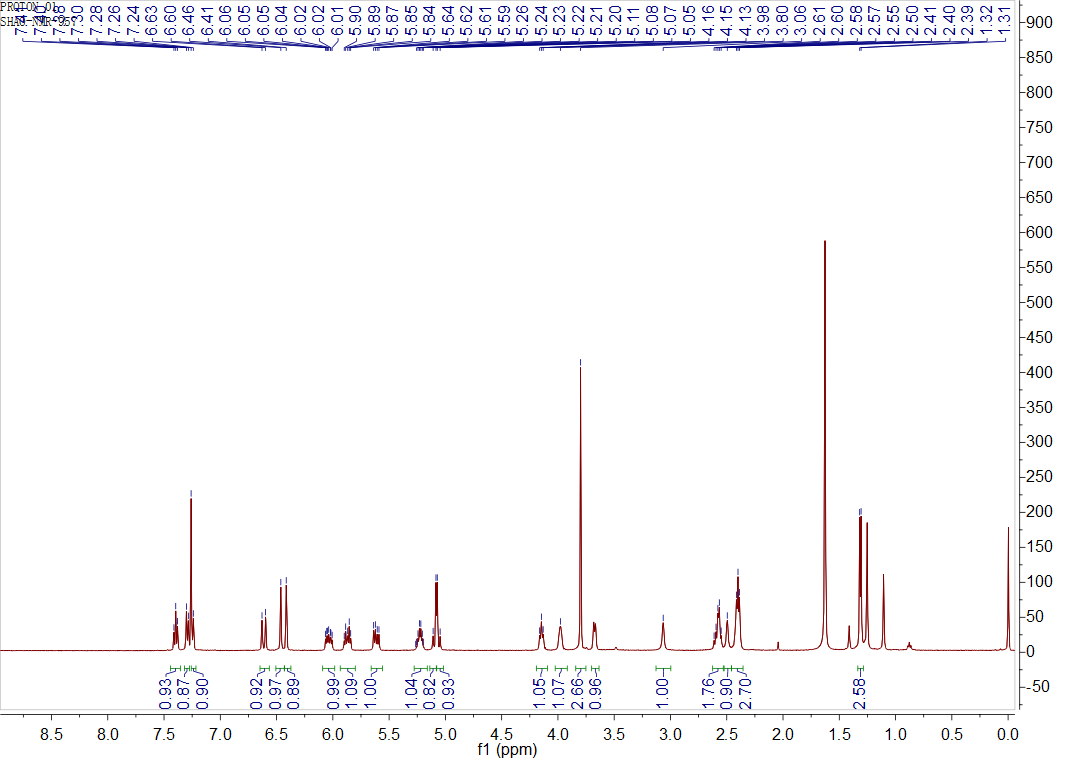


**Figure S67.** ^1^H NMR (500 MHz, CDCl_3_) spectrum of compound **24**


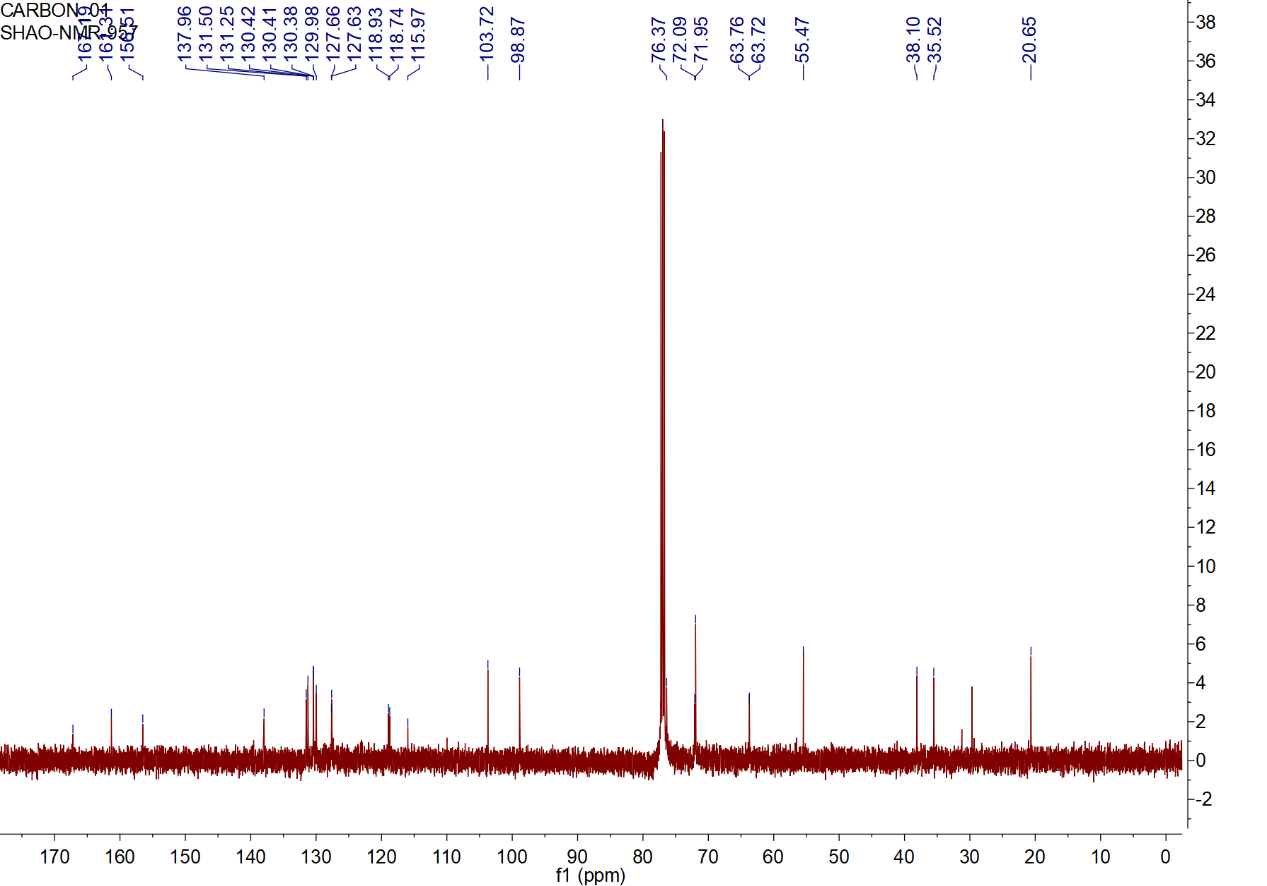


**Figure S68.** ^13^C NMR (125 MHz, CDCl_3_) spectrum of compound **24**

**Figure S69.** HRESIMS spectrum of compound **24**


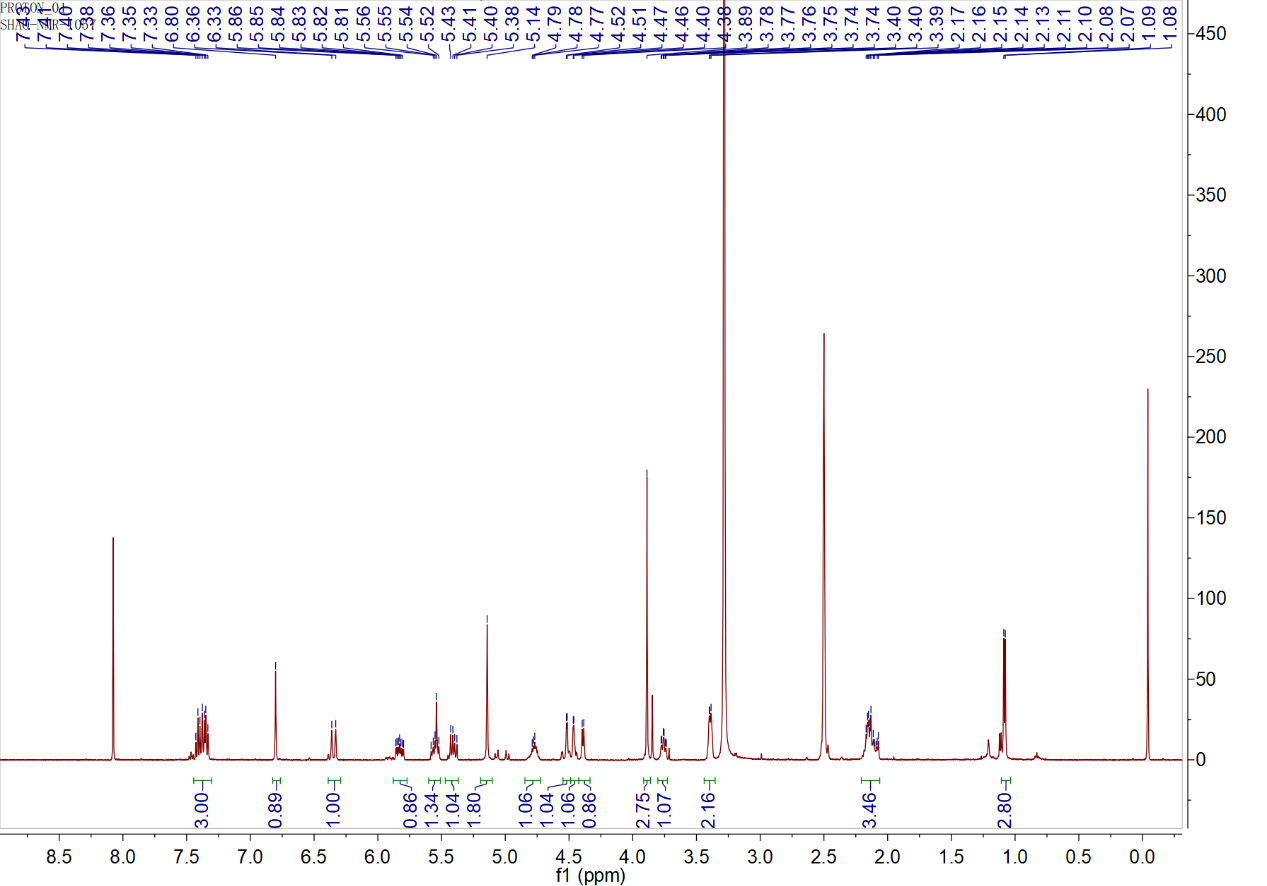


**Figure S70.** ^1^H NMR (500 MHz, CDCl_3_) spectrum of compound **25**


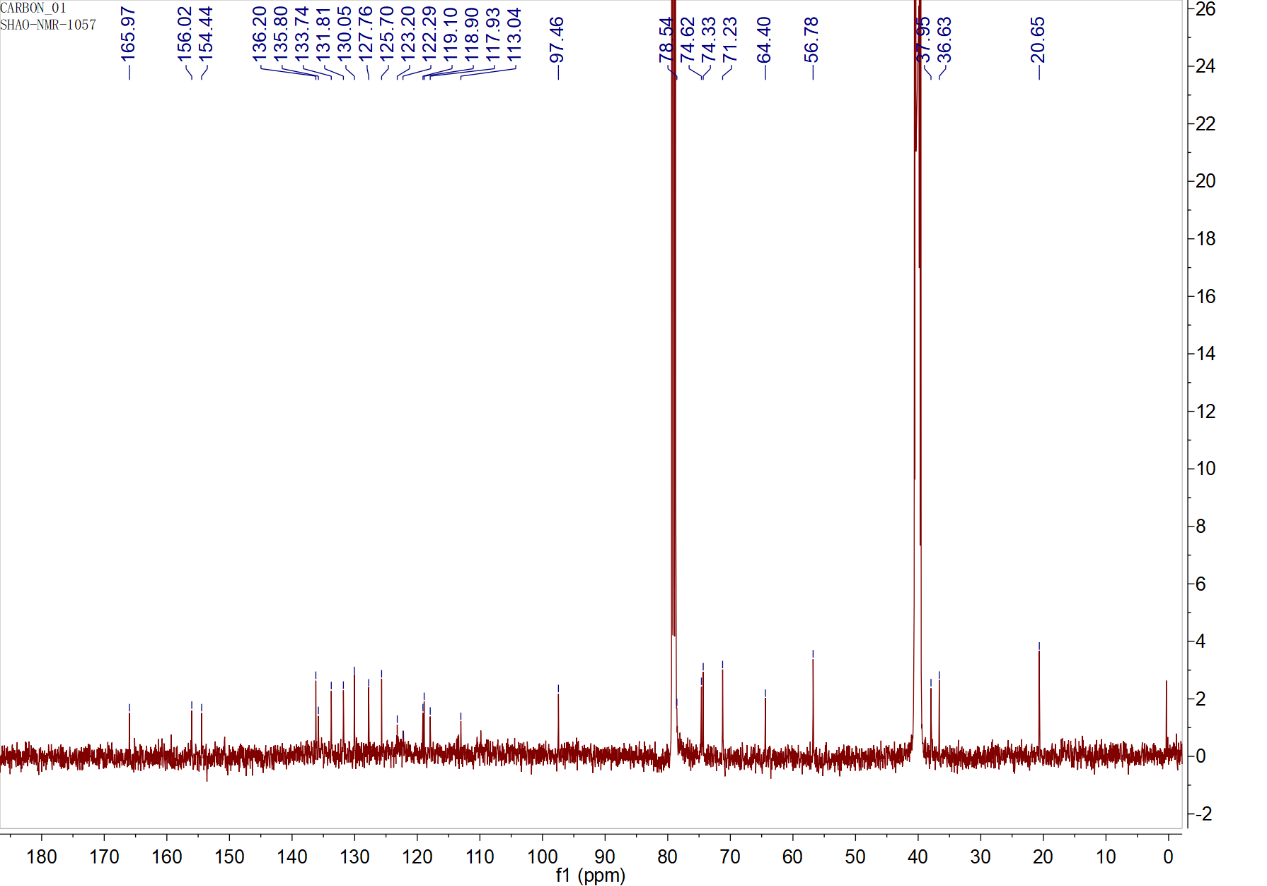


**Figure S71.** ^13^C NMR (125 MHz, CDCl_3_) spectrum of compound **25**

**Figure S72.** HRESIMS spectrum of compound **25**


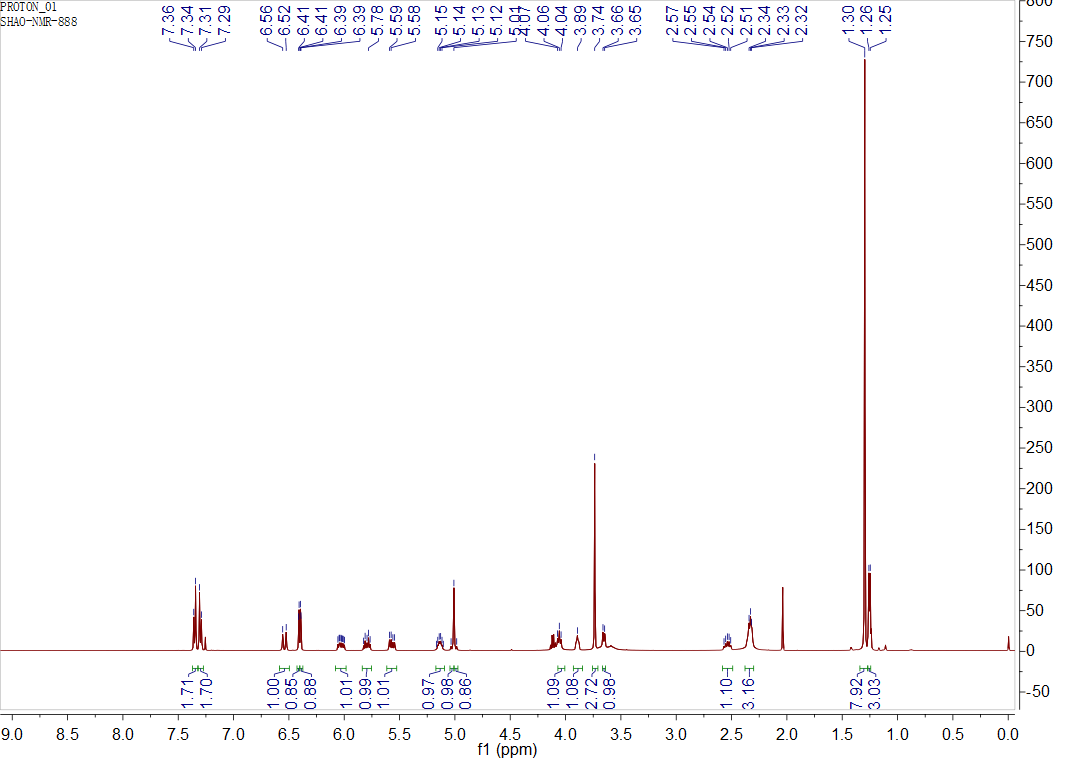


**Figure S73.** ^1^H NMR (500 MHz, CDCl_3_) spectrum of compound **26**


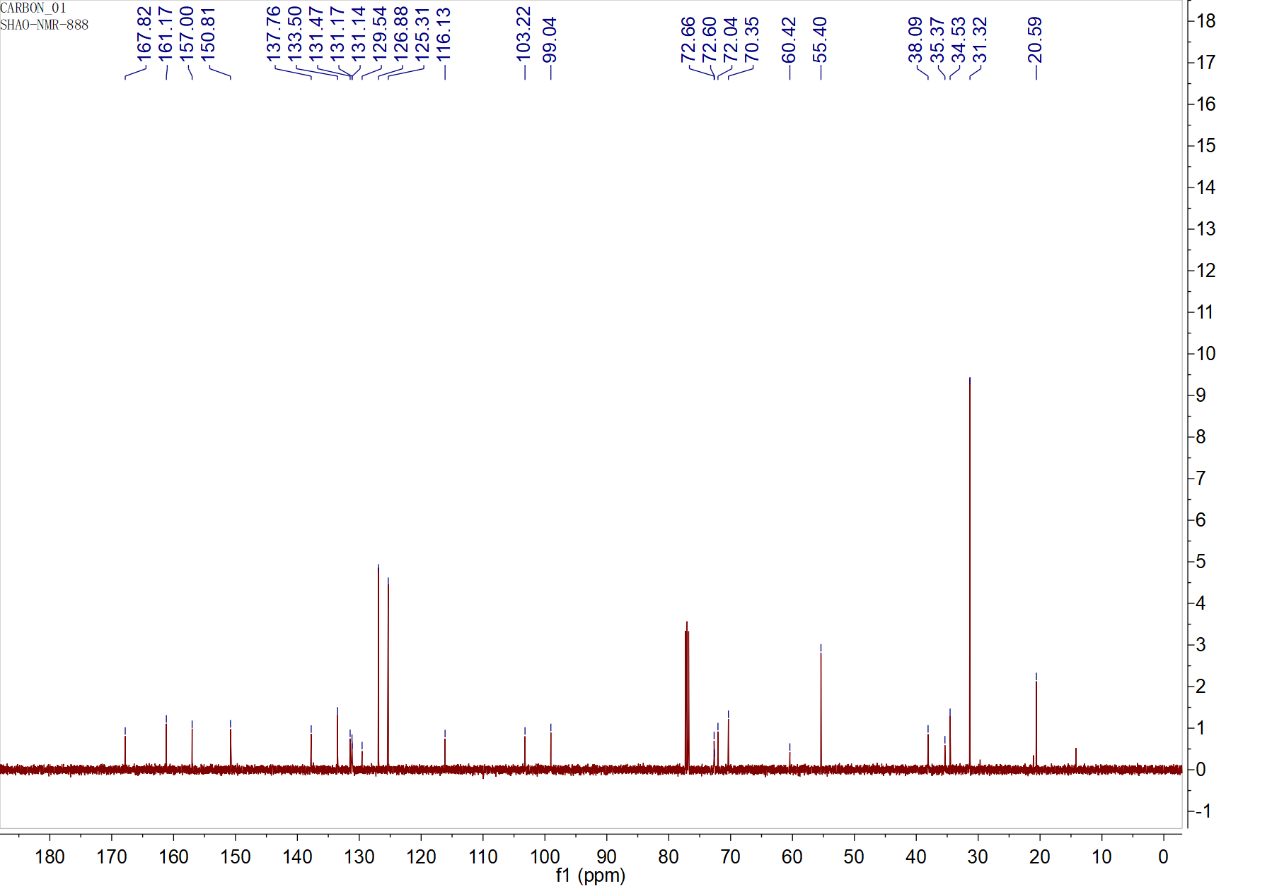


**Figure S74.** ^13^C NMR (125 MHz, CDCl_3_) spectrum of compound **26**

**Figure S75.** HRESIMS spectrum of compound **26**


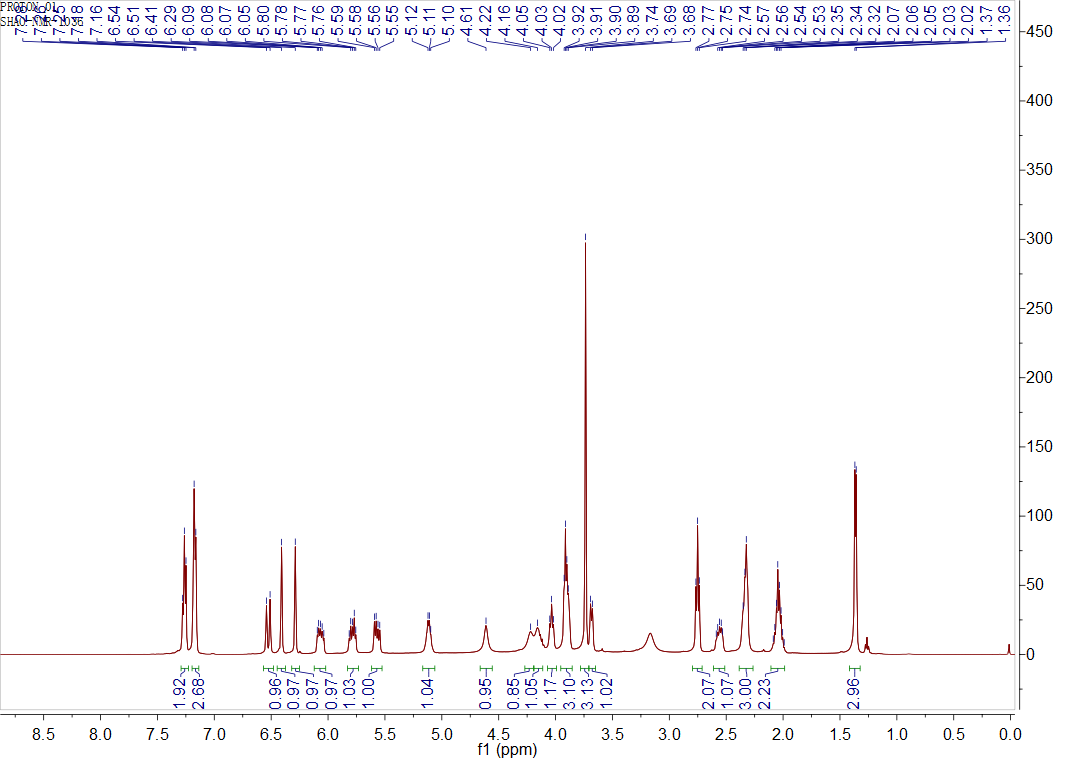


**Figure S76.** ^1^H NMR (500 MHz, CDCl_3_) spectrum of compound **27**


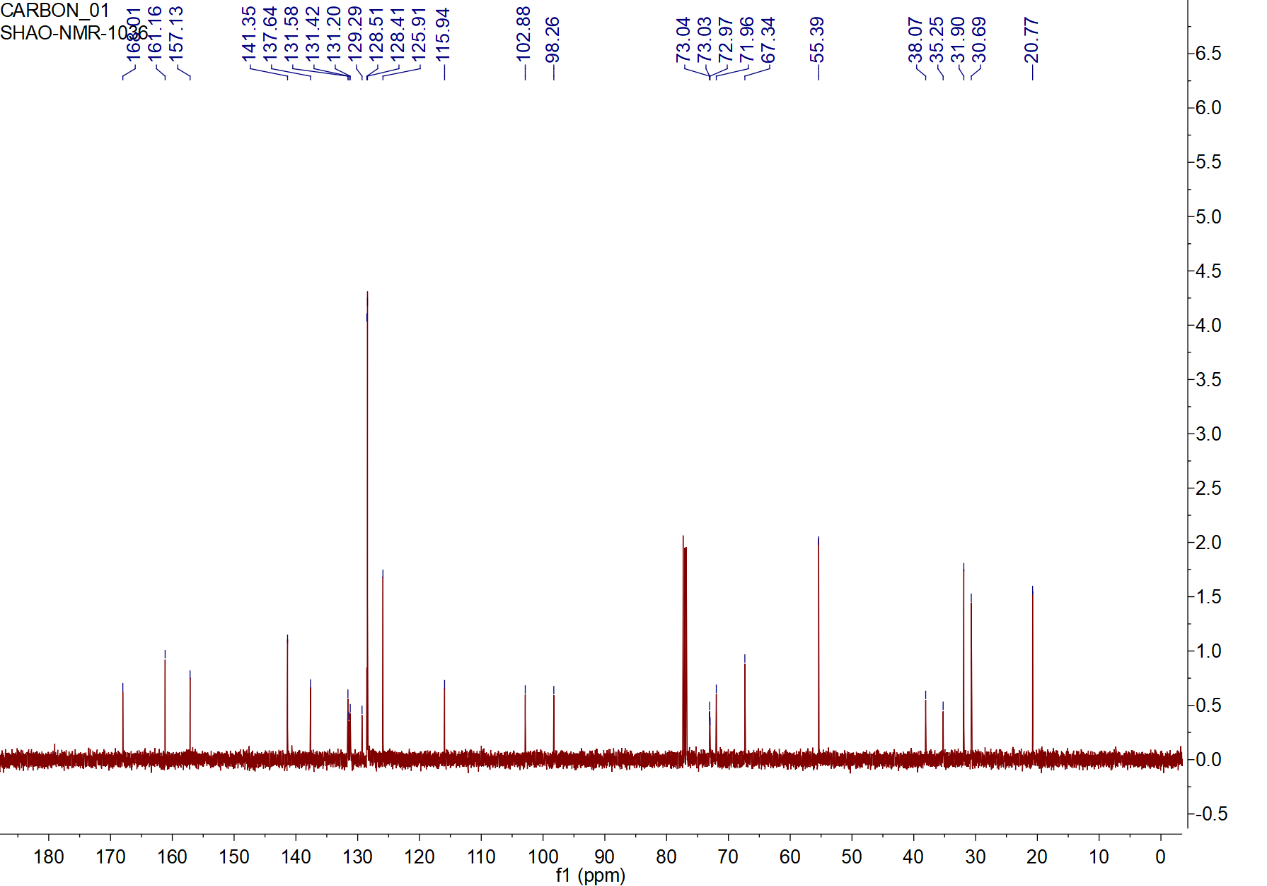


**Figure S77.** ^13^C NMR (125 MHz, CDCl_3_) spectrum of compound **27**

**Figure S78.** HRESIMS spectrum of compound **27**


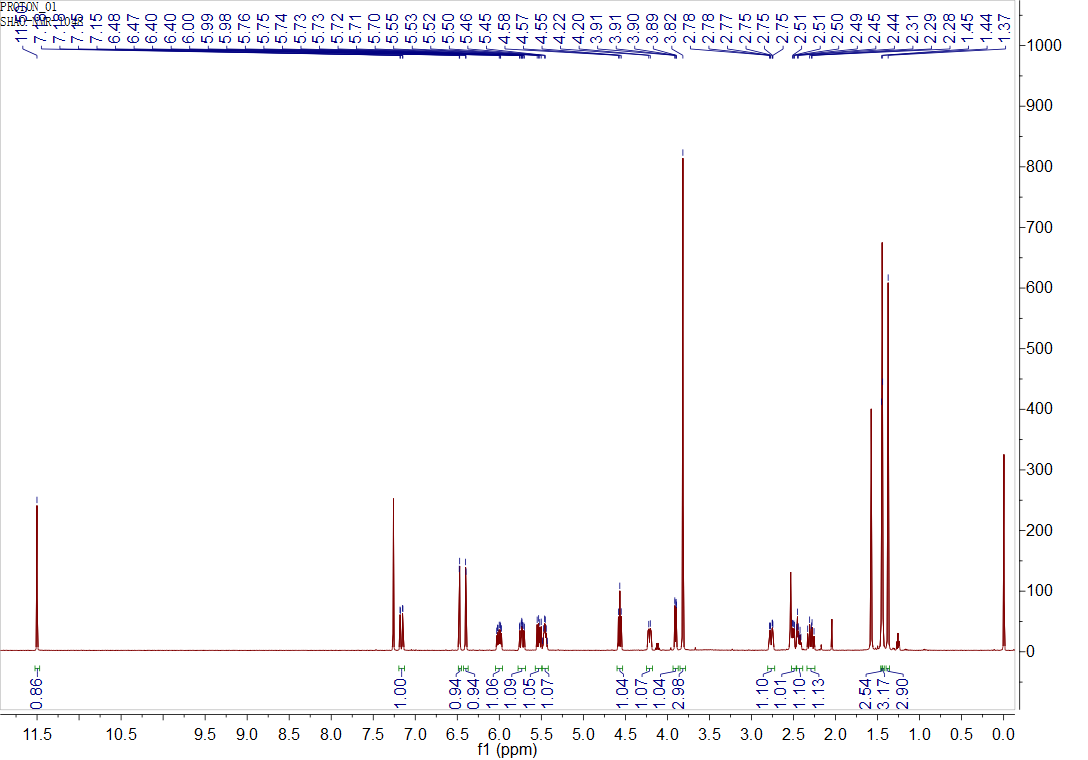


**Figure S79.** ^1^H NMR (500 MHz, CDCl_3_) spectrum of compound **28**


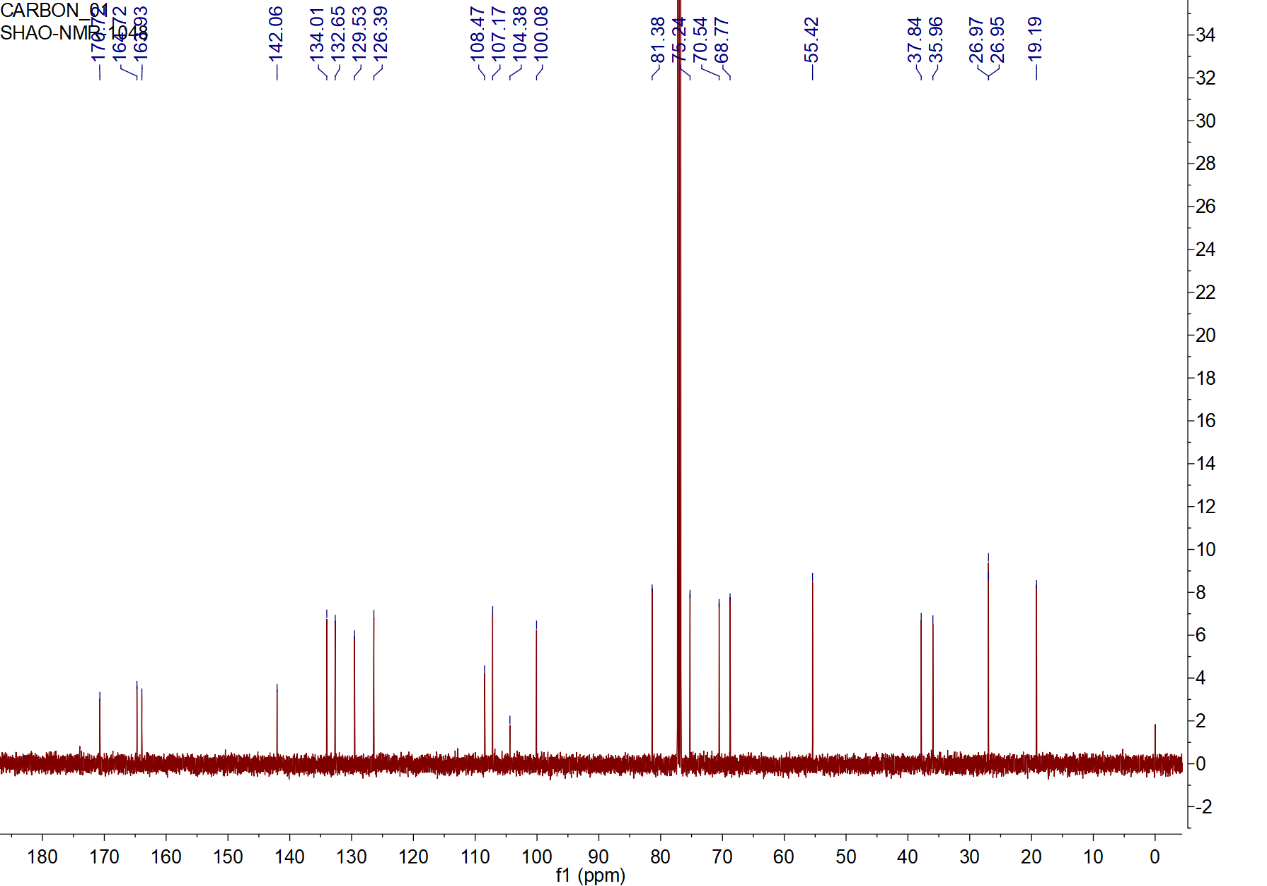


**Figure S80.** ^13^C NMR (125 MHz, CDCl_3_) spectrum of compound **28**

**Figure S81.** HRESIMS spectrum of compound **28**


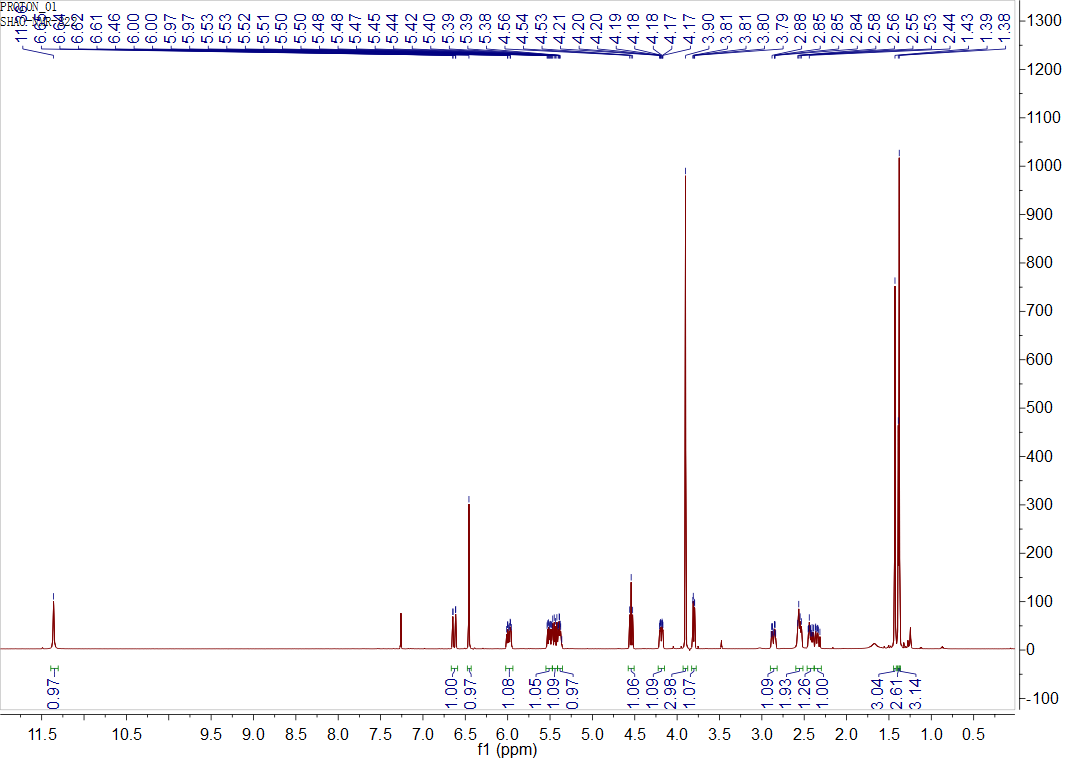


**Figure S82.** ^1^H NMR (500 MHz, CDCl_3_) spectrum of compound **29**


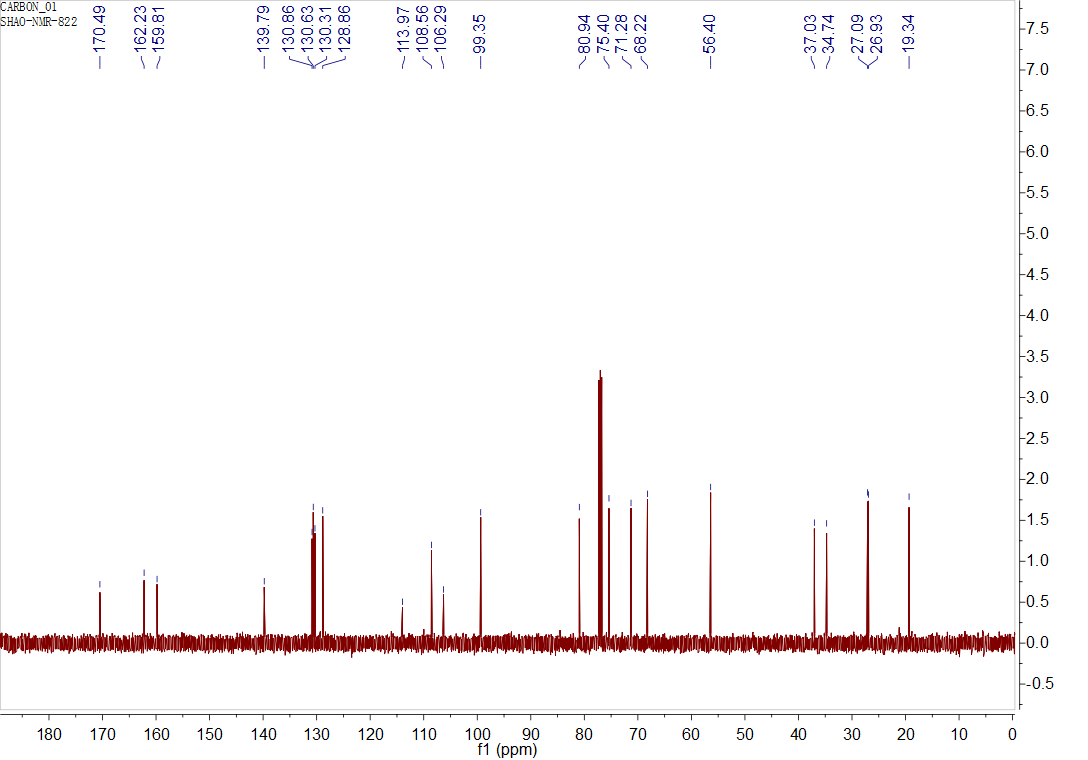


**Figure S83.** ^13^C NMR (125 MHz, CDCl_3_) spectrum of compound **29**

**Figure S84.** HRESIMS spectrum of compound **29**


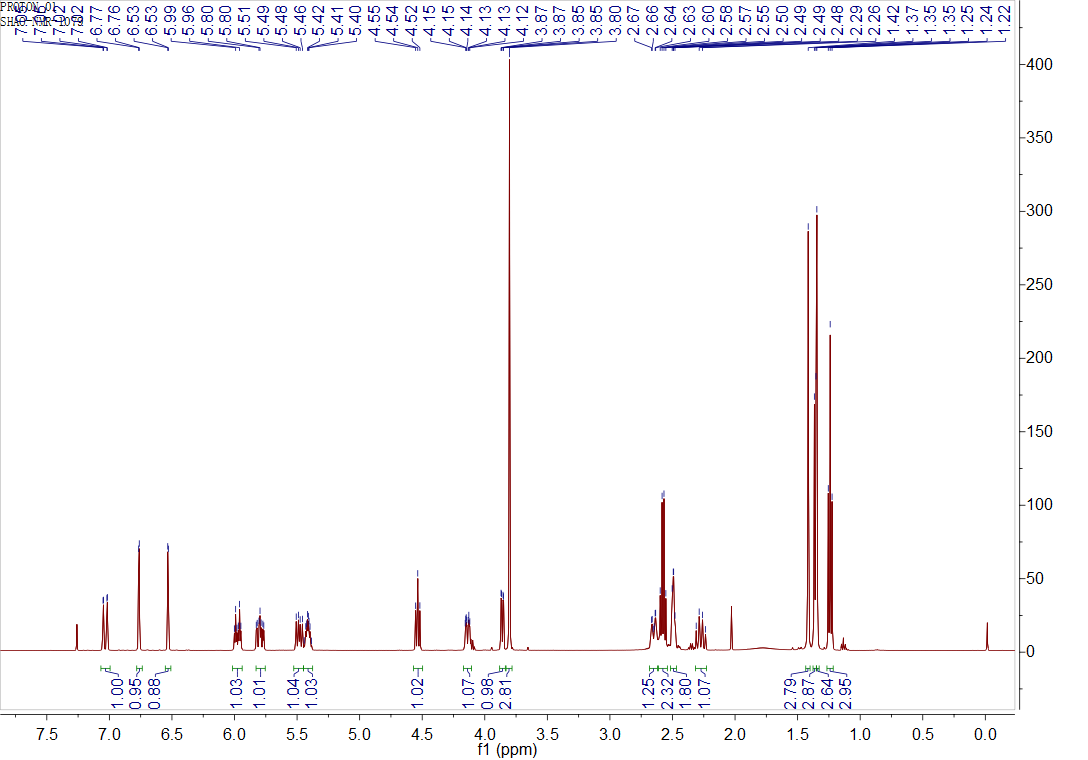


**Figure S85.** ^1^H NMR (500 MHz, CDCl_3_) spectrum of compound **30**


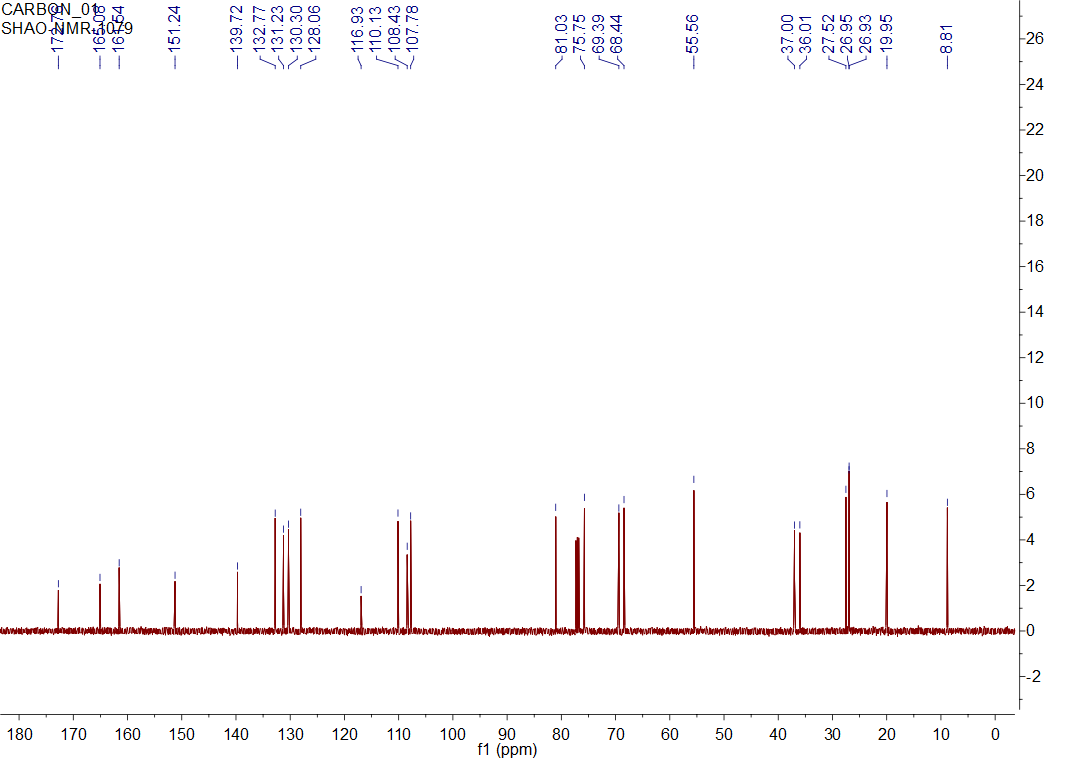


**Figure S86.** ^13^C NMR (125 MHz, CDCl_3_) spectrum of compound **30**

**Figure S87.** HRESIMS spectrum of compound **30**


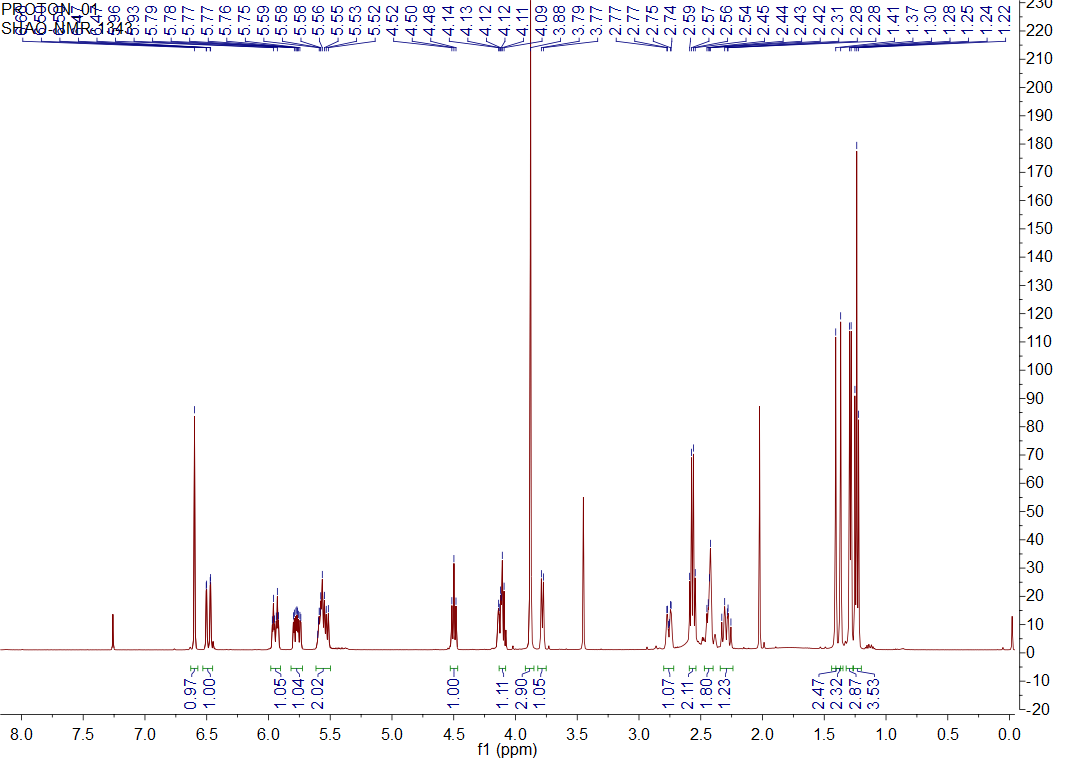


**Figure S88.** ^1^H NMR (500 MHz, CDCl_3_) spectrum of compound **31**


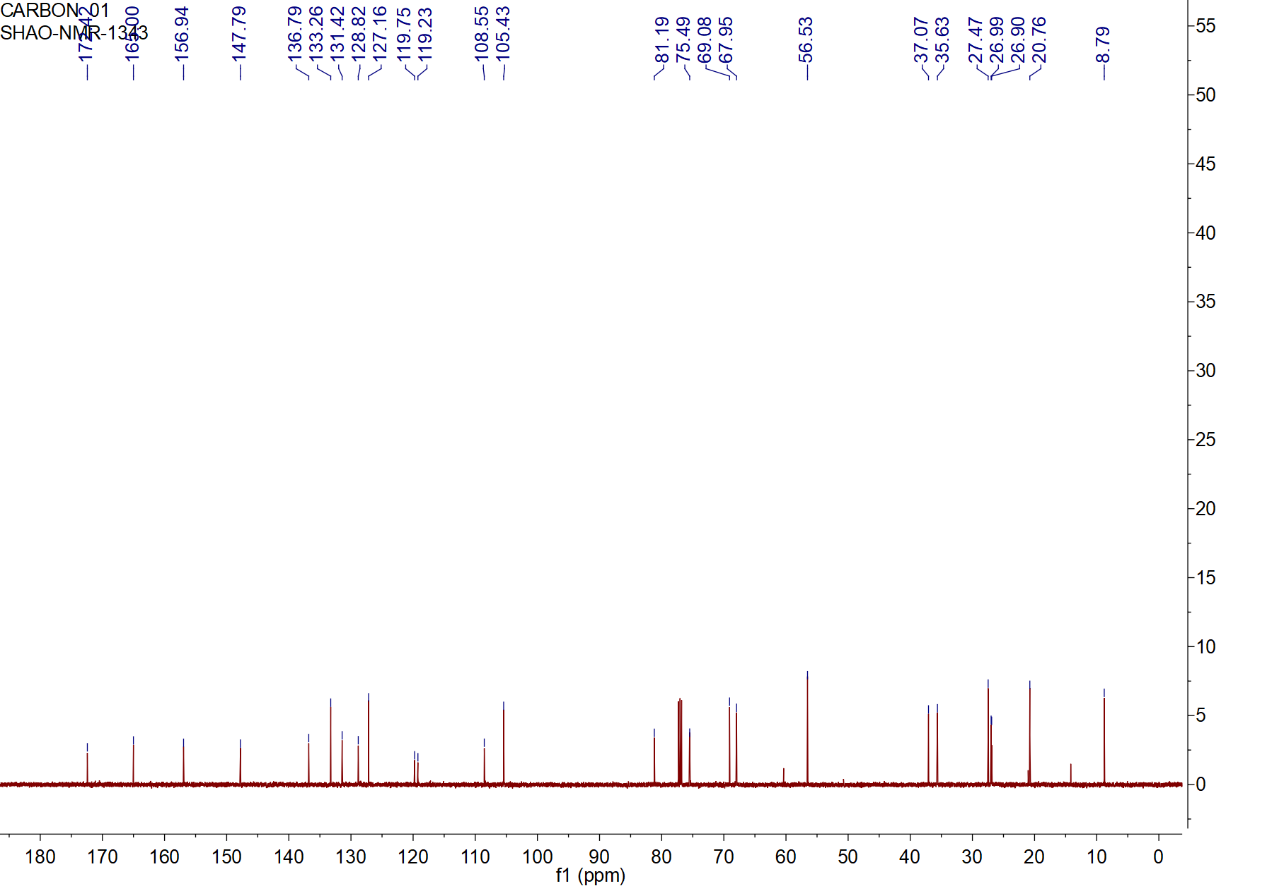


**Figure S89.** ^13^C NMR (125 MHz, CDCl_3_) spectrum of compound **31**


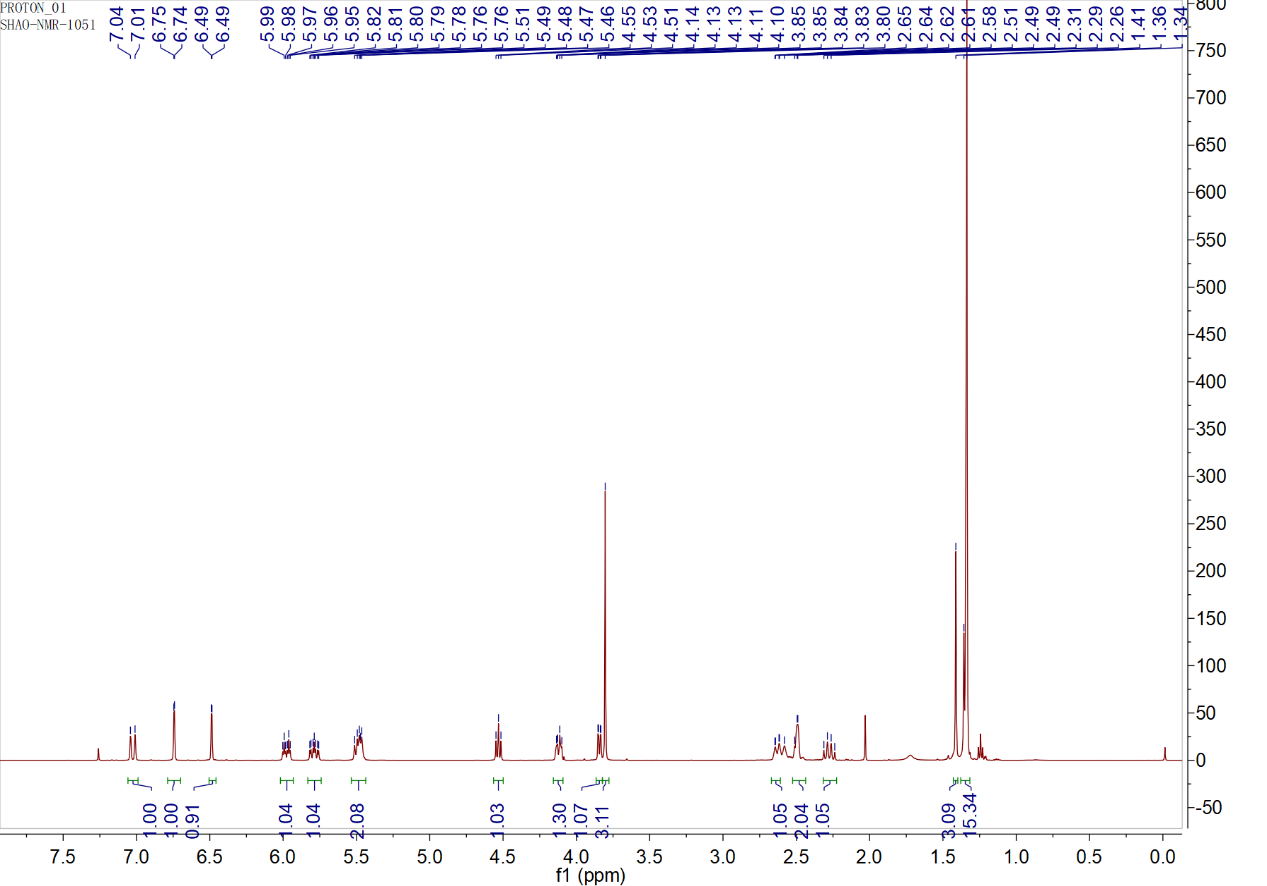


**Figure S90.** ^1^H NMR (500 MHz, CDCl_3_) spectrum of compound **32**


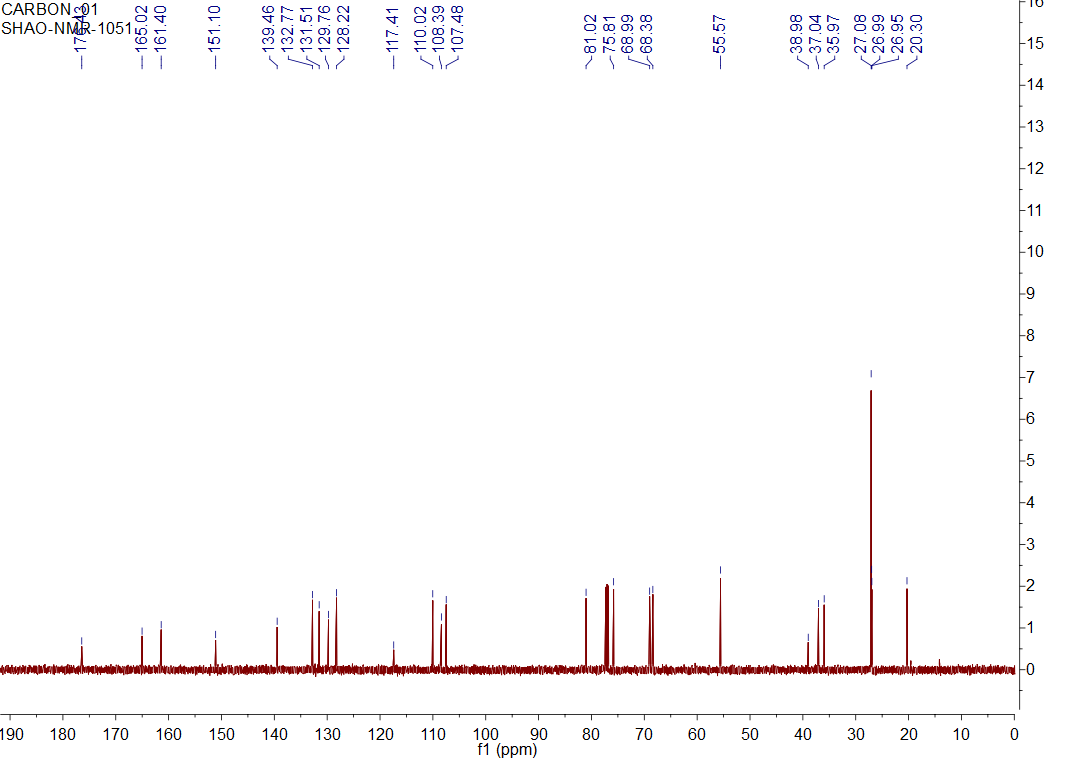


**Figure S91.** ^13^C NMR (125 MHz, CDCl_3_) spectrum of compound **32**

**Figure S92.** HRESIMS spectrum of compound **32**


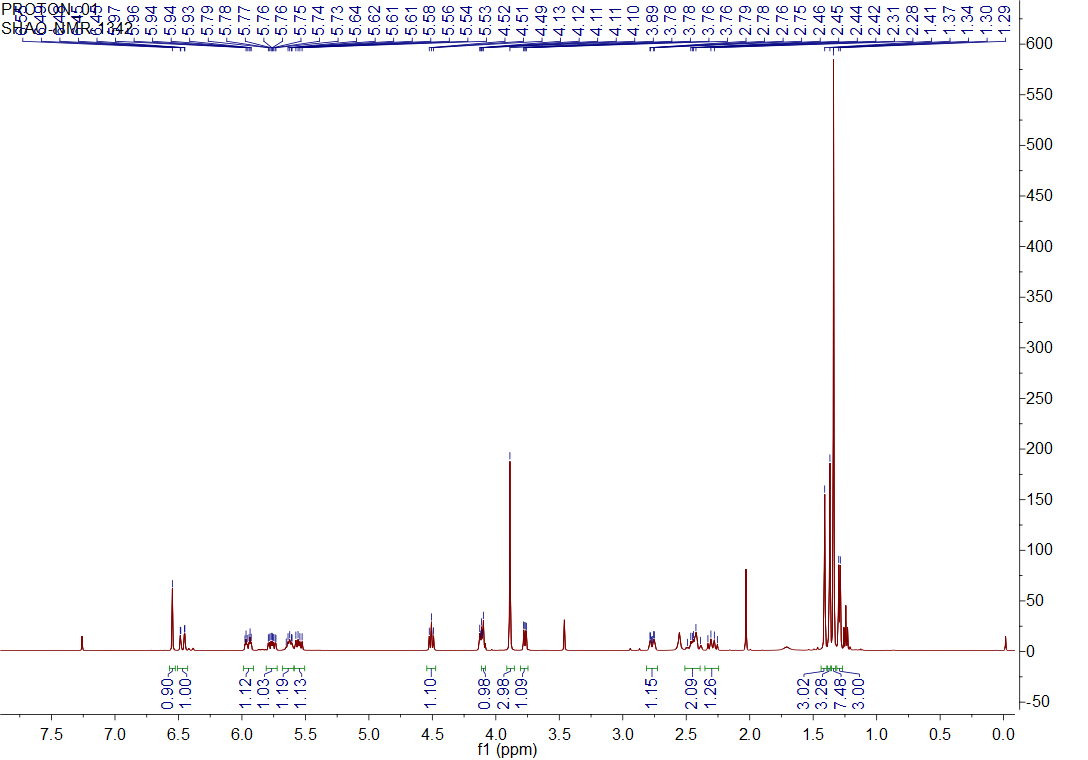


**Figure S93.** ^1^H NMR (500 MHz, CDCl_3_) spectrum of compound **33**


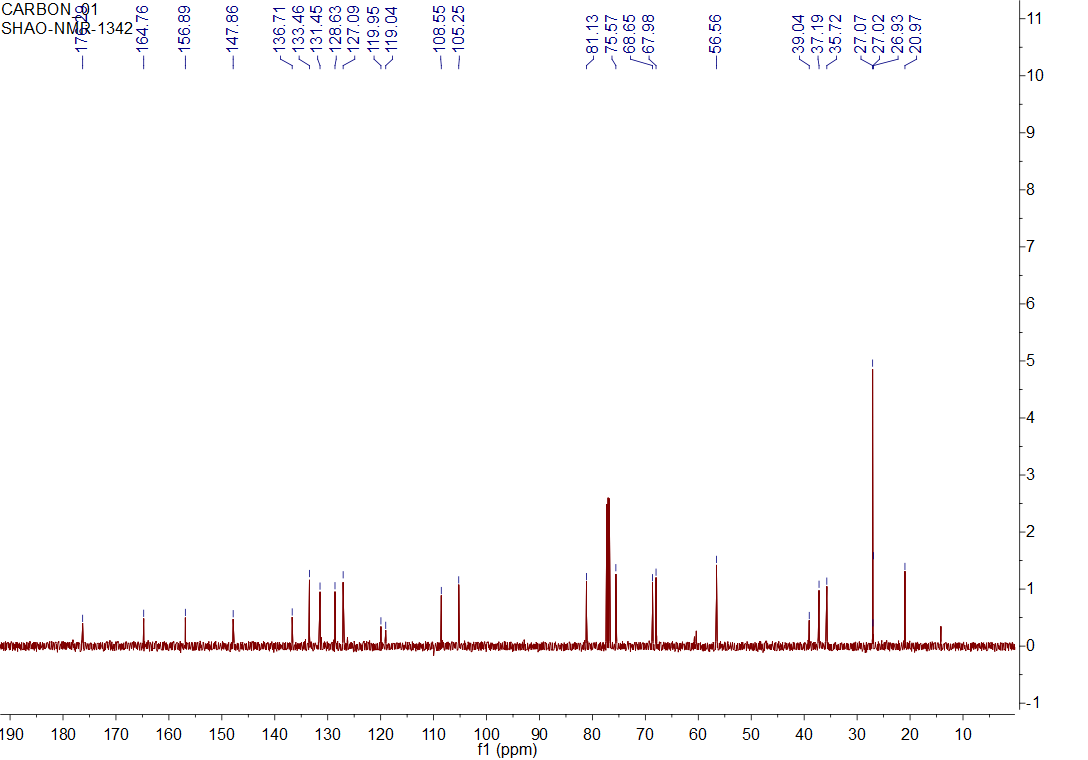


**Figure S94.** ^13^C NMR (125 MHz, CDCl_3_) spectrum of compound **33**


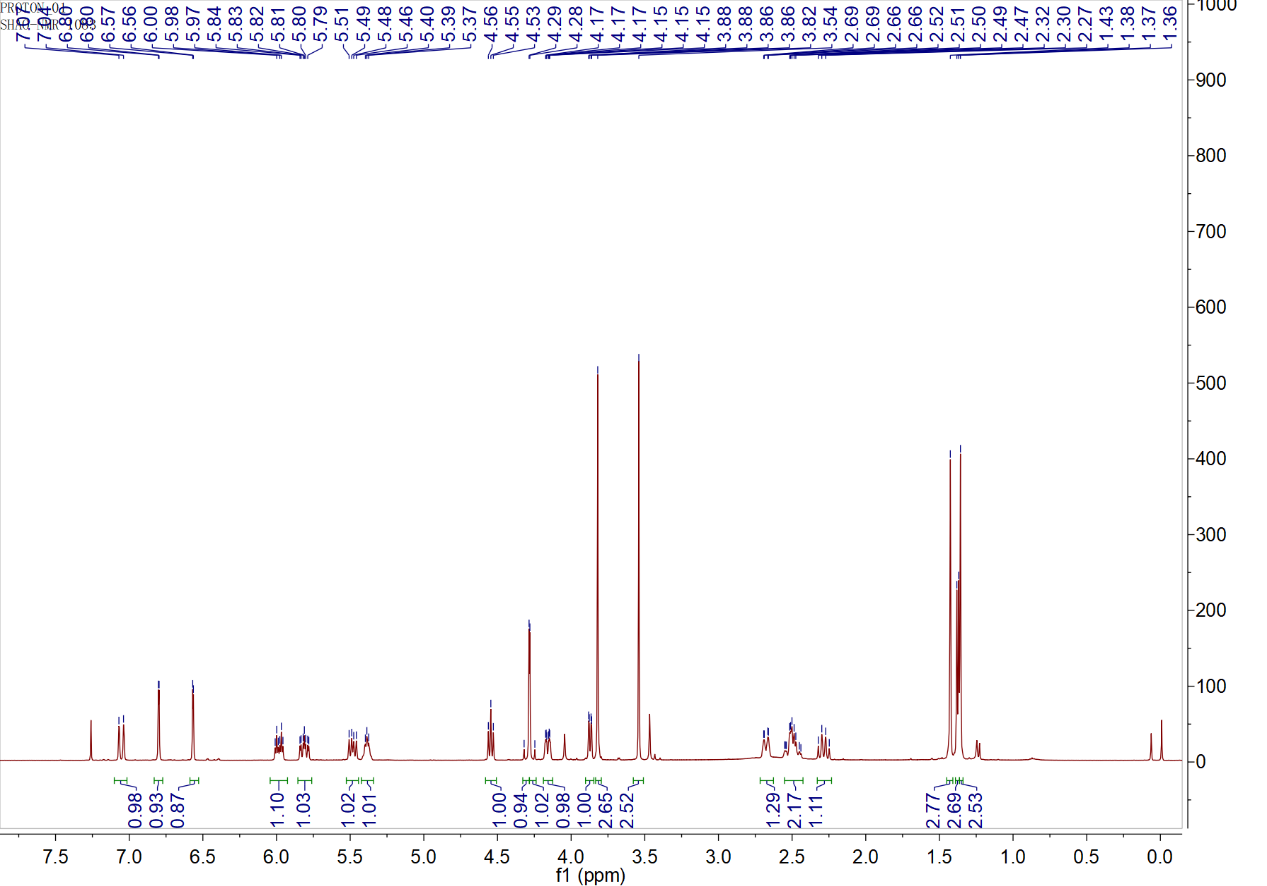


**Figure S95.** ^1^H NMR (500 MHz, CDCl_3_) spectrum of compound **34**


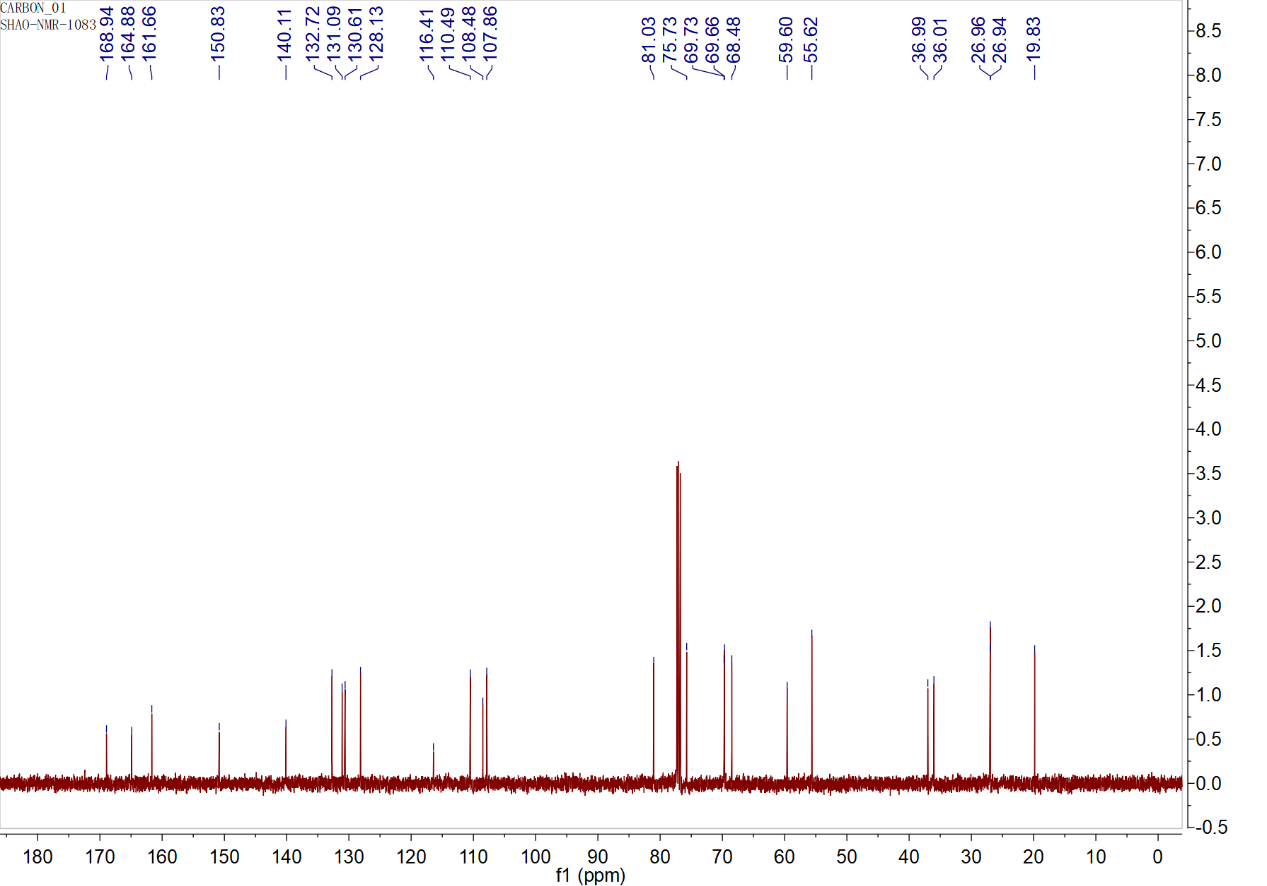


**Figure S96.** ^13^C NMR (125 MHz, CDCl_3_) spectrum of compound **34**

**Figure S97.** HRESIMS spectrum of compound **34**


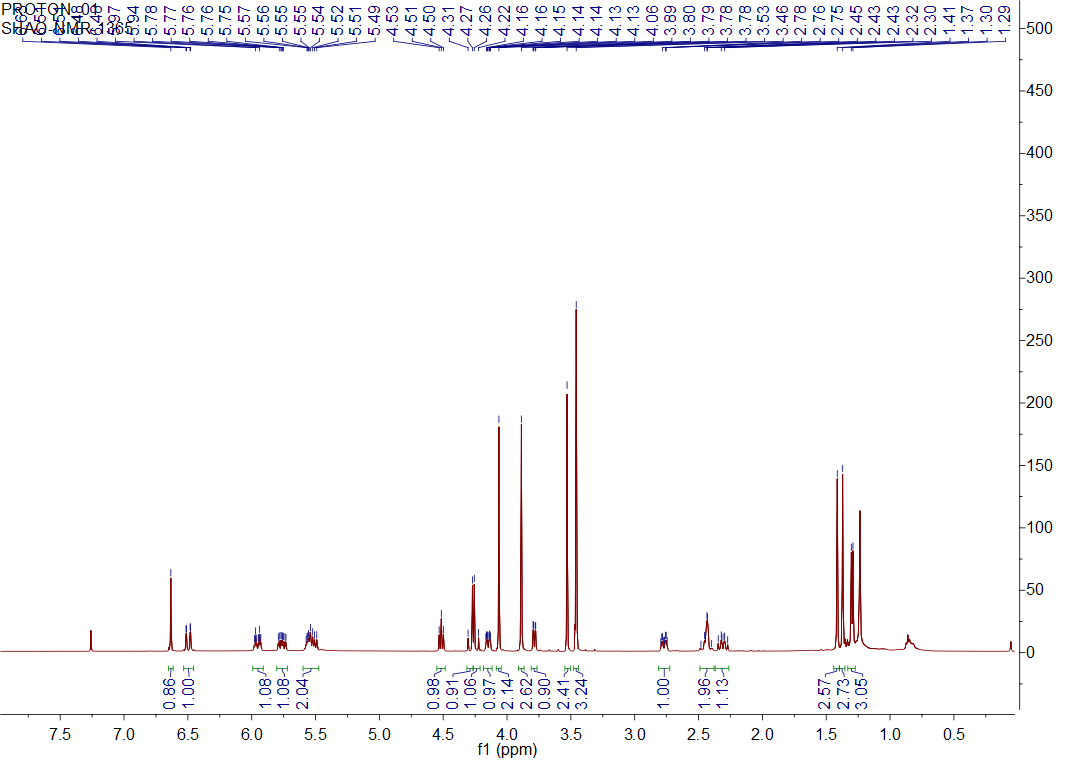


**Figure S98.** ^1^H NMR (500 MHz, CDCl_3_) spectrum of compound **35**


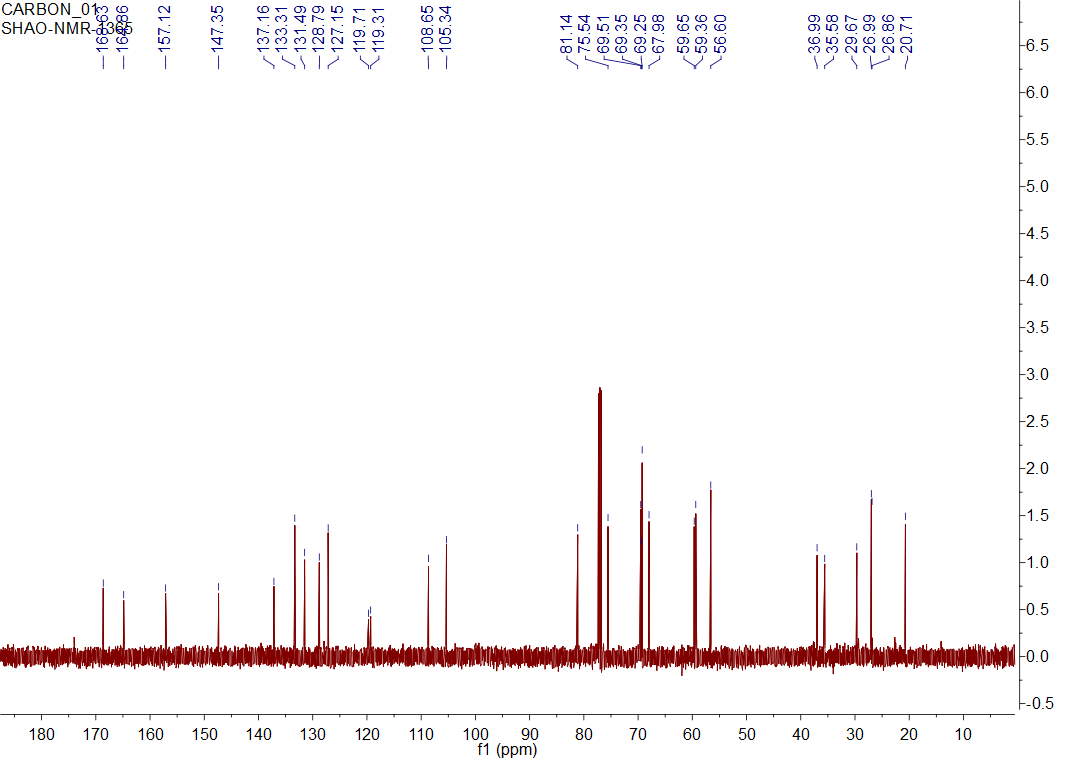


**Figure S99.** ^13^C NMR (125 MHz, CDCl_3_) spectrum of compound **35**

**Figure S100.** HRESIMS spectrum of compound **35**


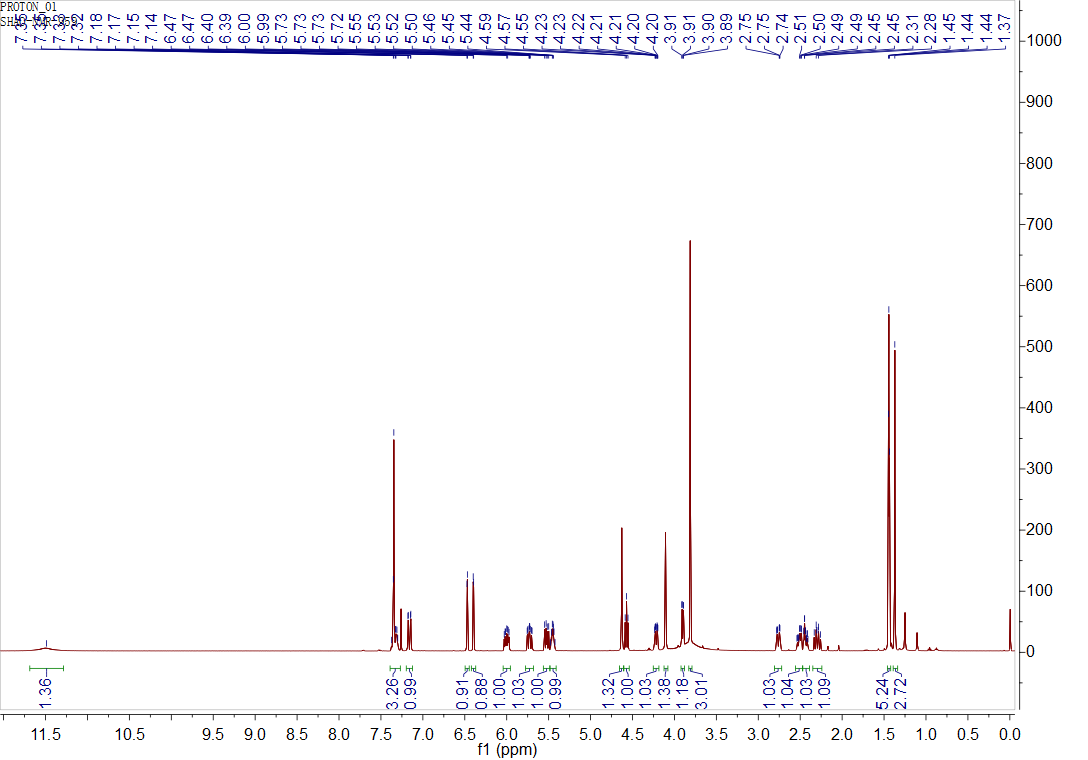


**Figure S101.** ^1^H NMR (500 MHz, CDCl_3_) spectrum of compound **36**


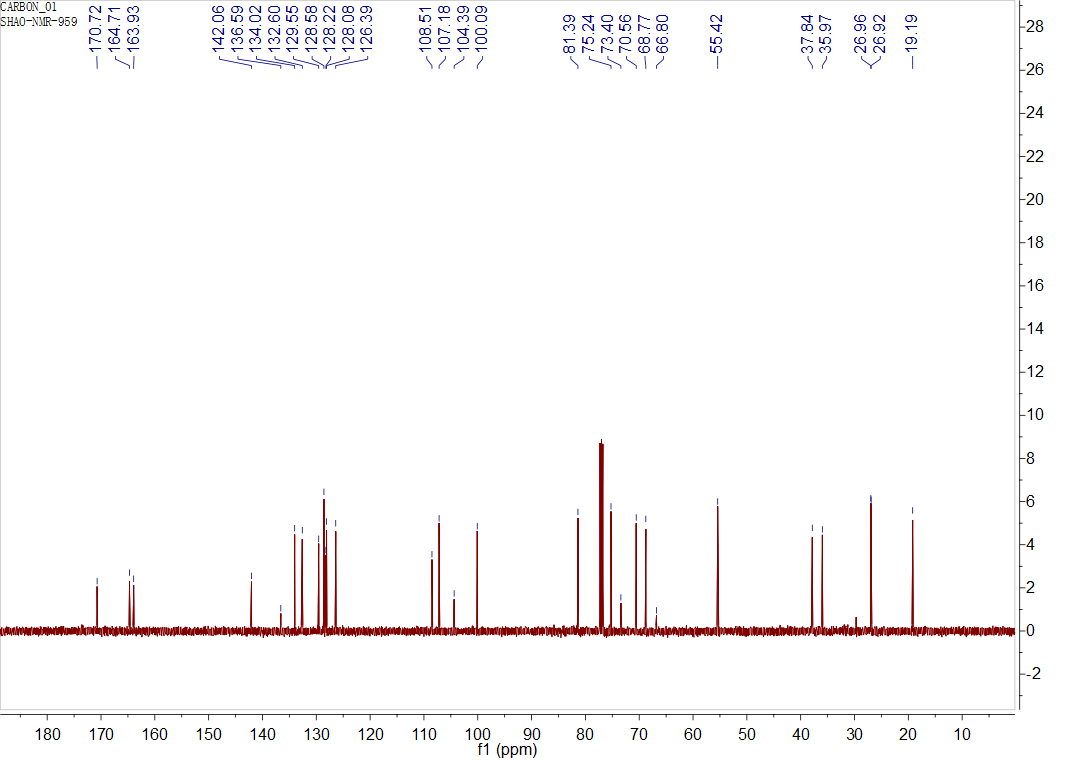


**Figure S102.** ^13^C NMR (125 MHz, CDCl_3_) spectrum of compound **36**

**Figure S103.** HRESIMS spectrum of compound **36**


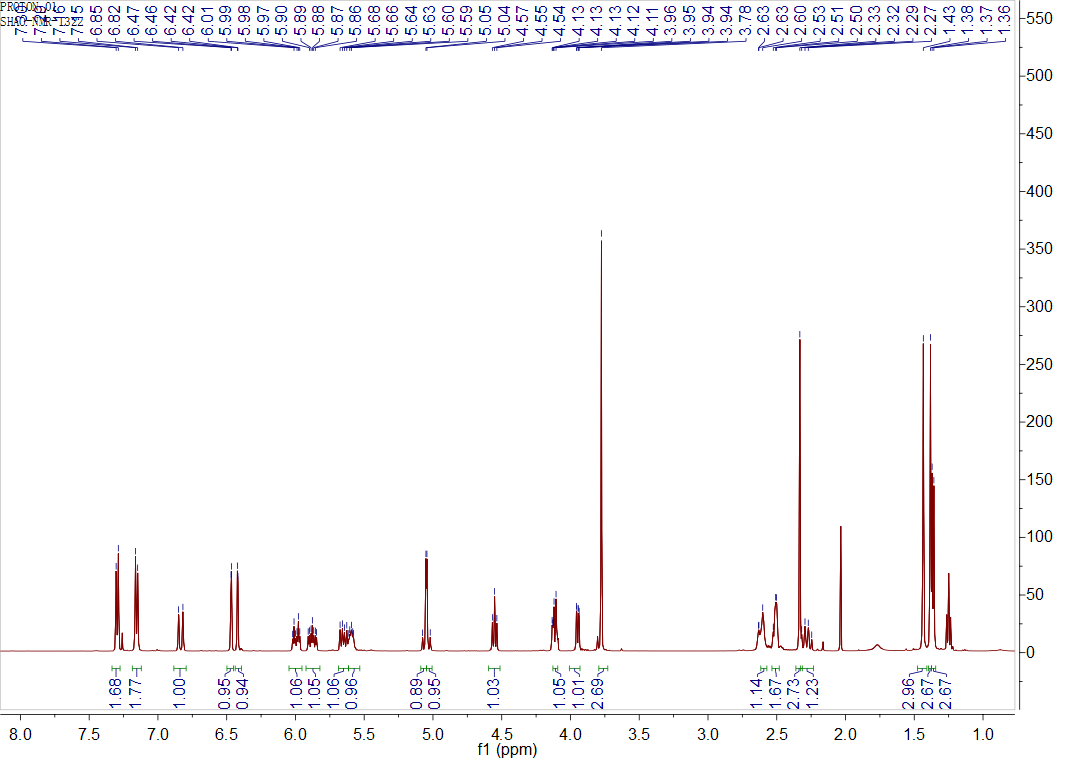


**Figure S104.** ^1^H NMR (500 MHz, CDCl_3_) spectrum of compound **37**


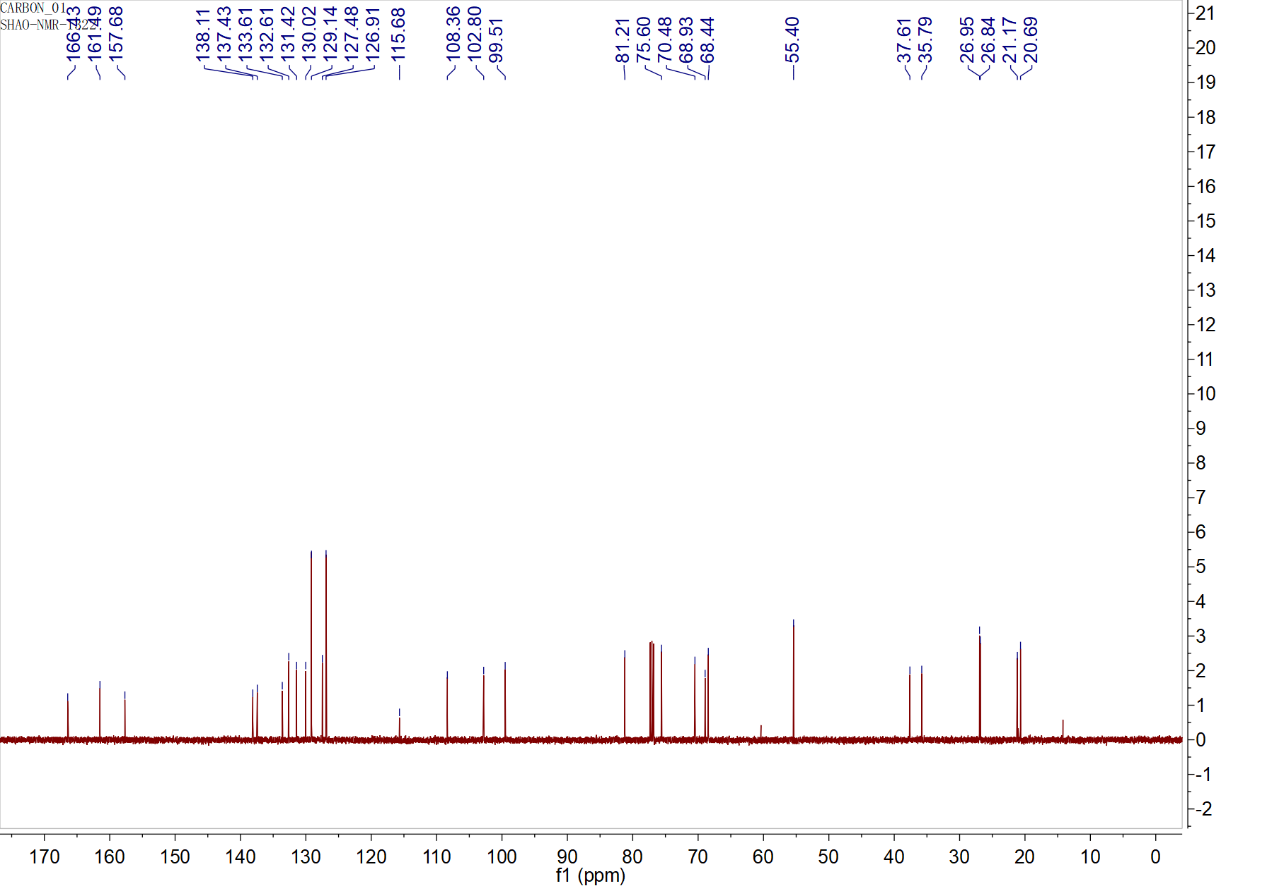


**Figure S105.** ^13^C NMR (125 MHz, CDCl_3_) spectrum of compound **37**

**Figure S106.** HRESIMS spectrum of compound **37**


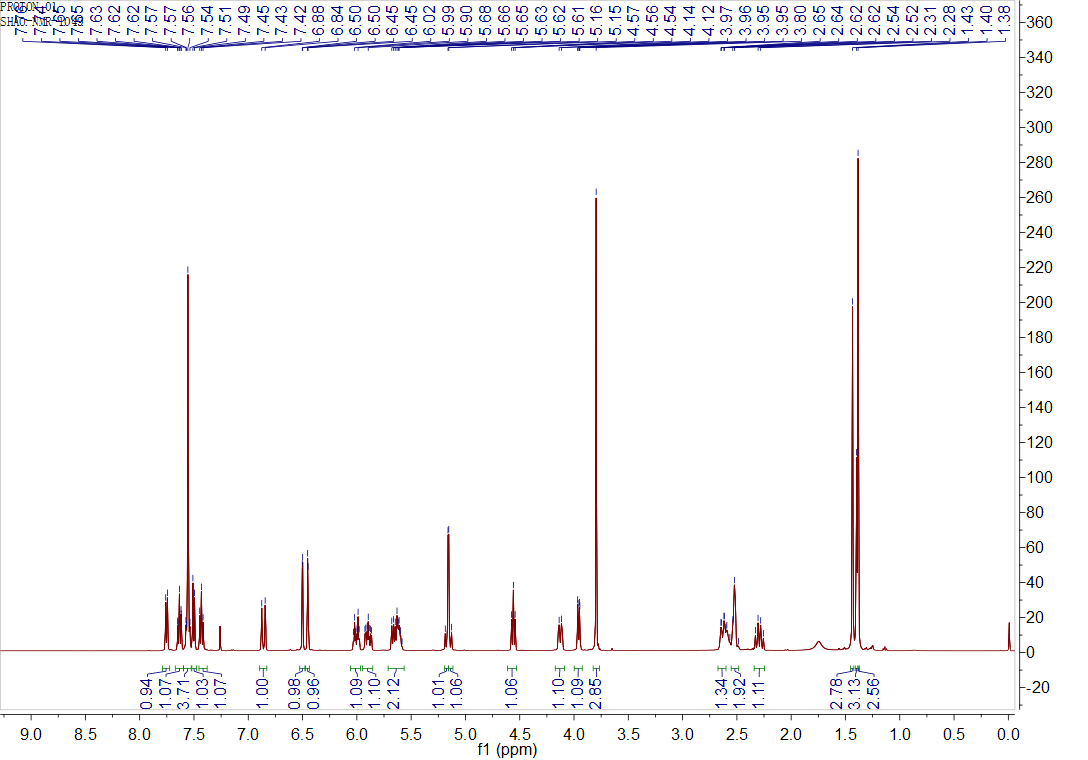


**Figure S107.** ^1^H NMR (500 MHz, CDCl_3_) spectrum of compound **38**


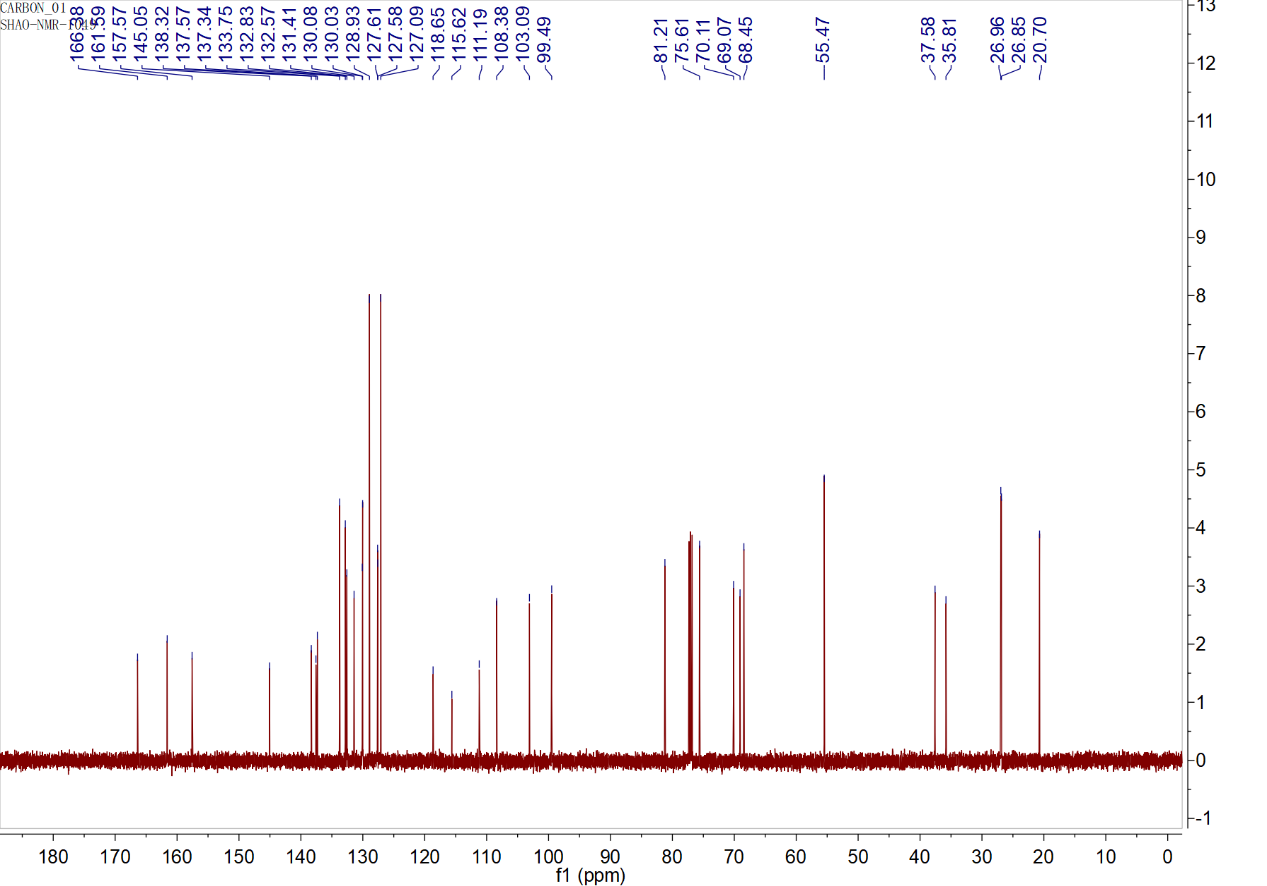


**Figure S108.** ^13^C NMR (125 MHz, CDCl_3_) spectrum of compound **38**

**Figure S109.** HRESIMS spectrum of compound **38**


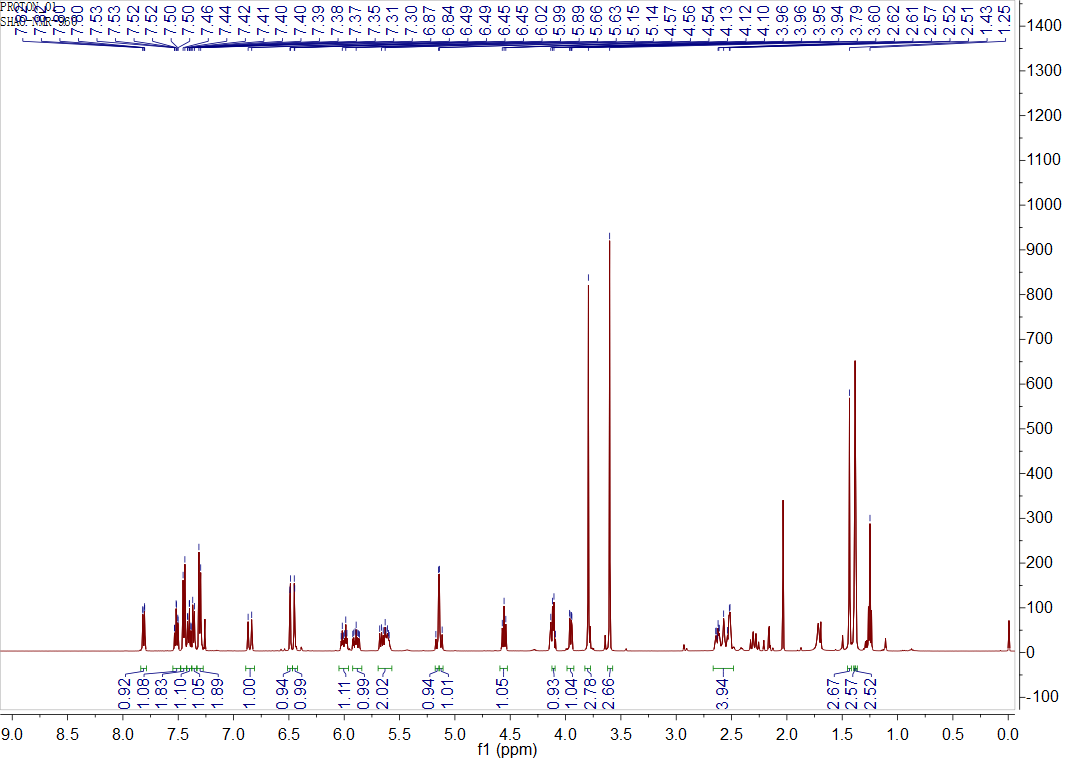


**Figure S110.** ^1^H NMR (500 MHz, CDCl_3_) spectrum of compound **39**


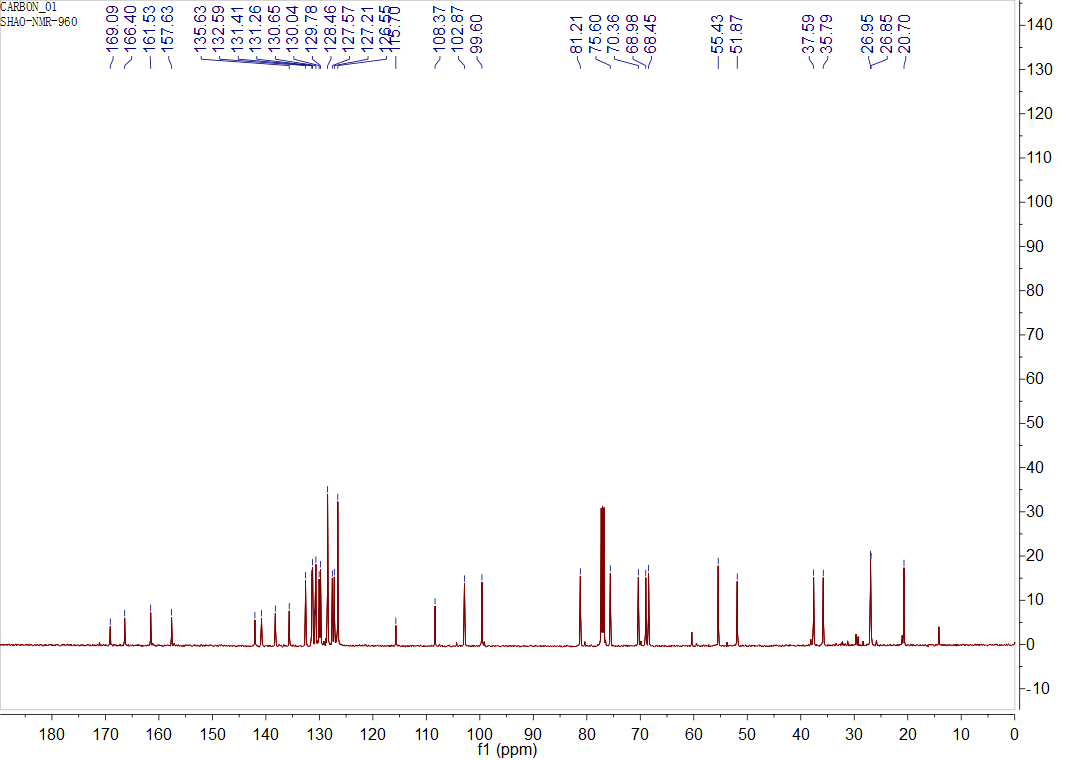


**Figure S111.** ^13^C NMR (125 MHz, CDCl_3_) spectrum of compound **39**

**Figure S112.** HRESIMS spectrum of compound **39**


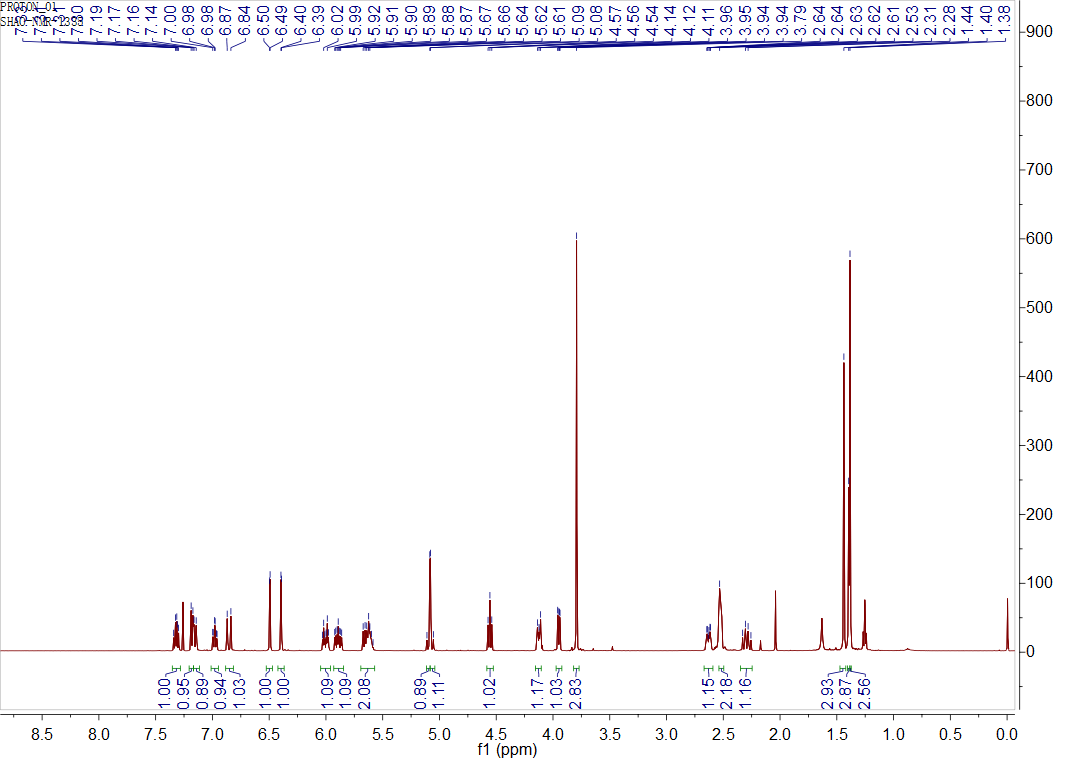


**Figure S113.** ^1^H NMR (500 MHz, CDCl_3_) spectrum of compound **40**


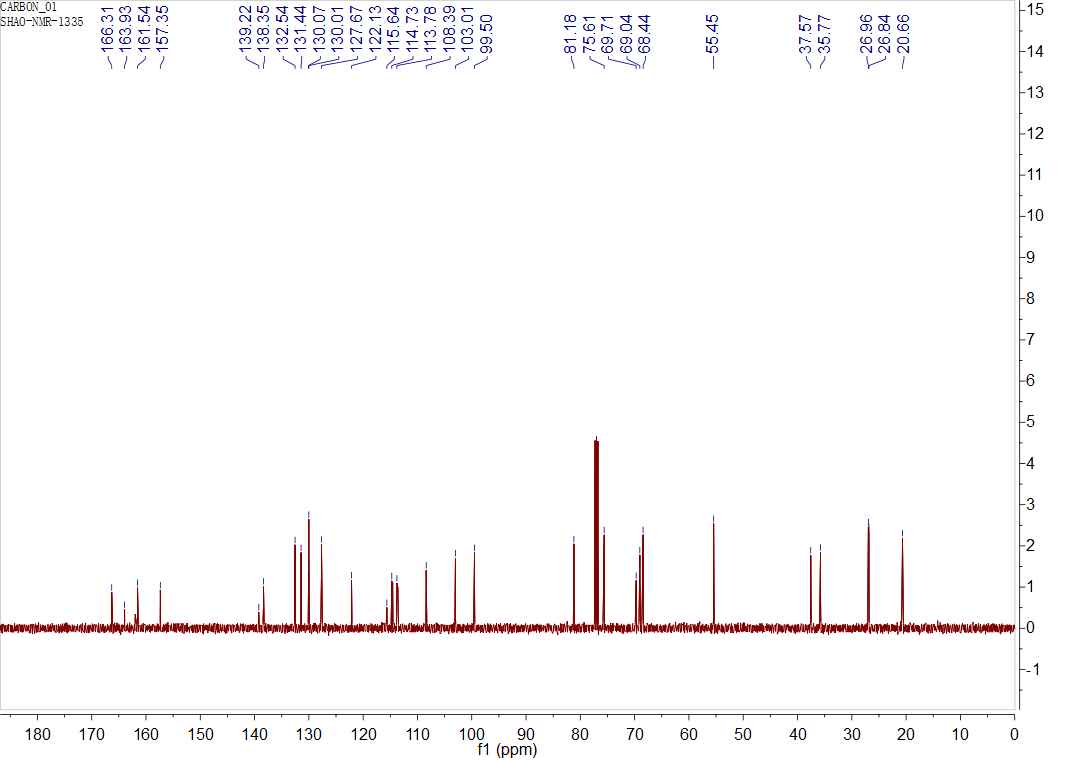


**Figure S114.** ^13^C NMR (125 MHz, CDCl_3_) spectrum of compound **40**

**Figure S115.** HRESIMS spectrum of compound **40**


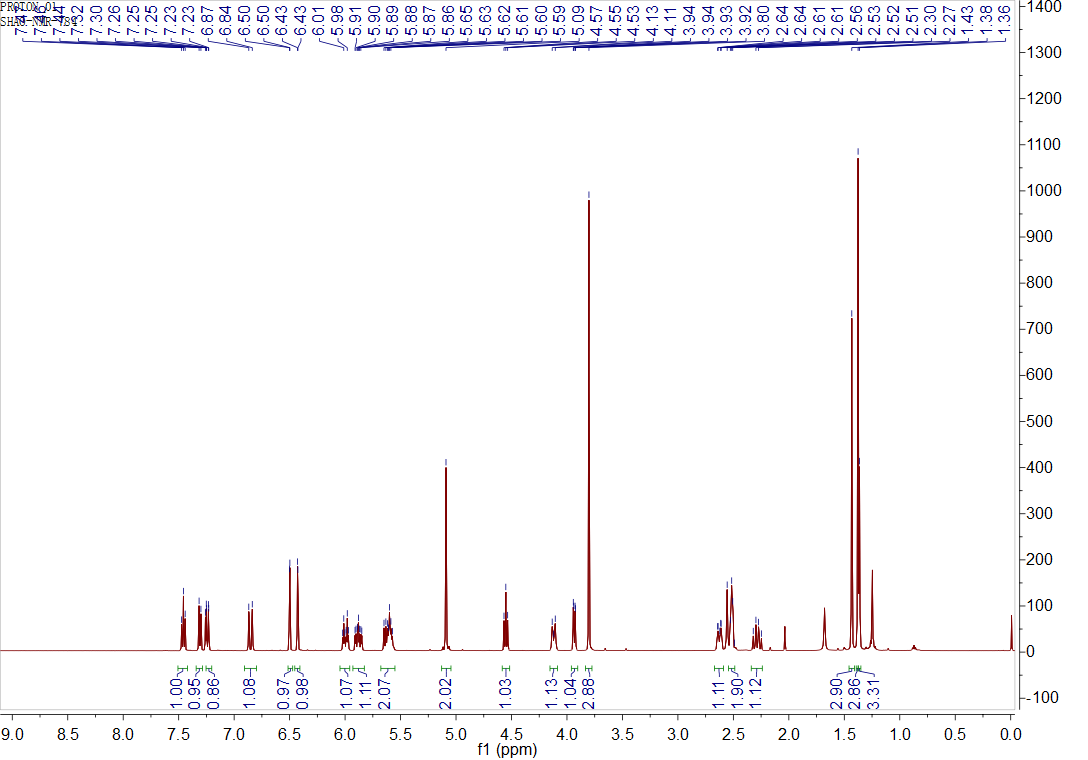


**Figure S116.** ^1^H NMR (500 MHz, CDCl_3_) spectrum of compound **41**


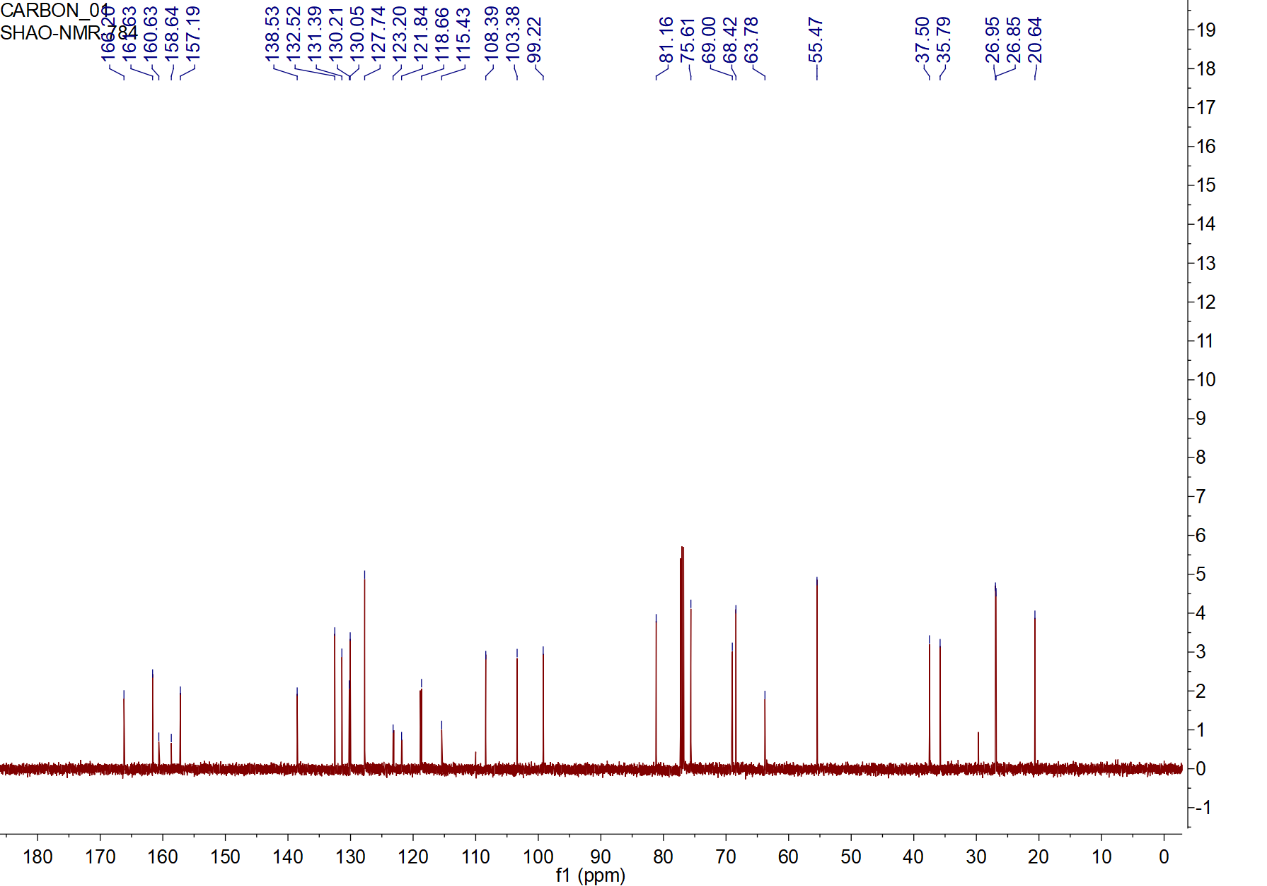


**Figure S117.** ^13^C NMR (125 MHz, CDCl_3_) spectrum of compound **41**

**Figure S118.** HRESIMS spectrum of compound **41**


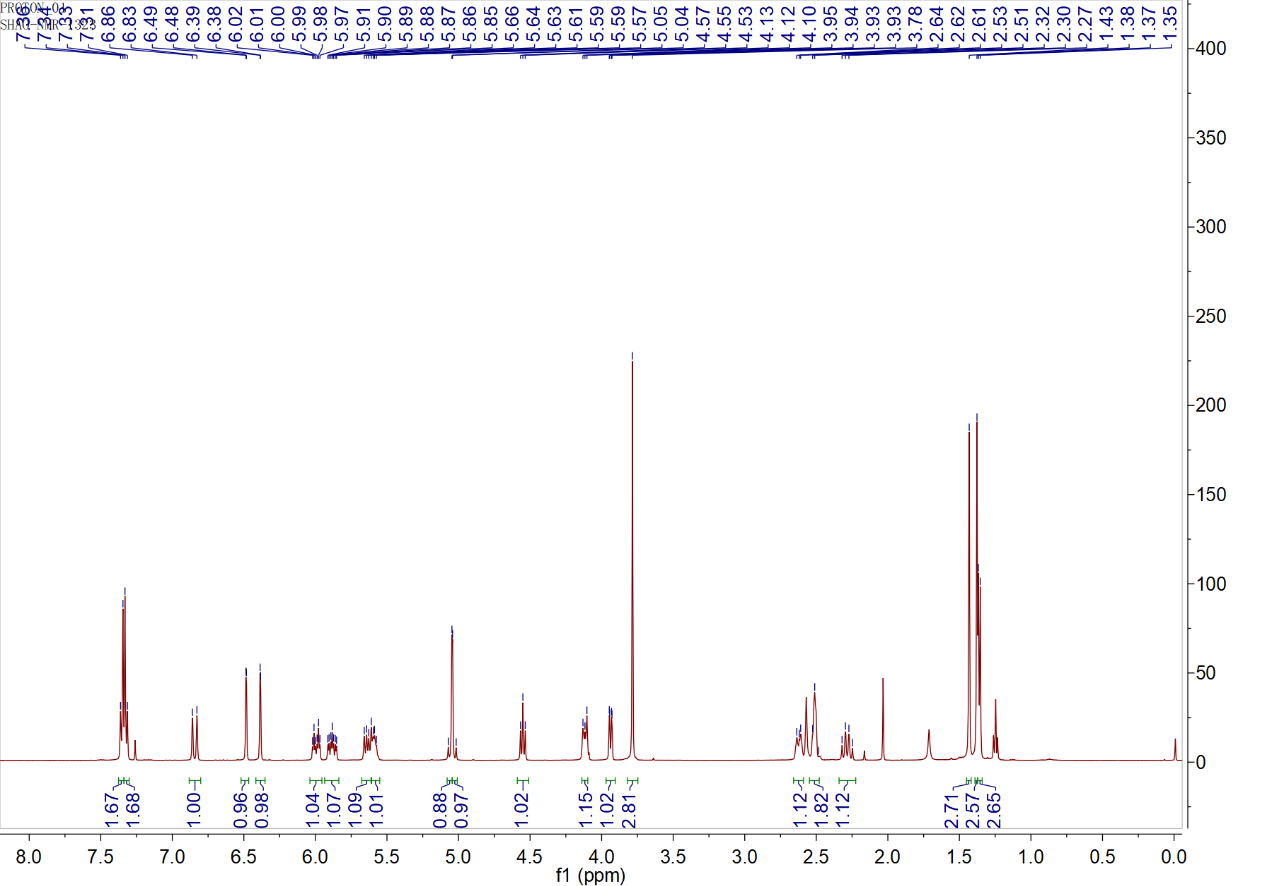


**Figure S119.** ^1^H NMR (500 MHz, CDCl_3_) spectrum of compound **42**


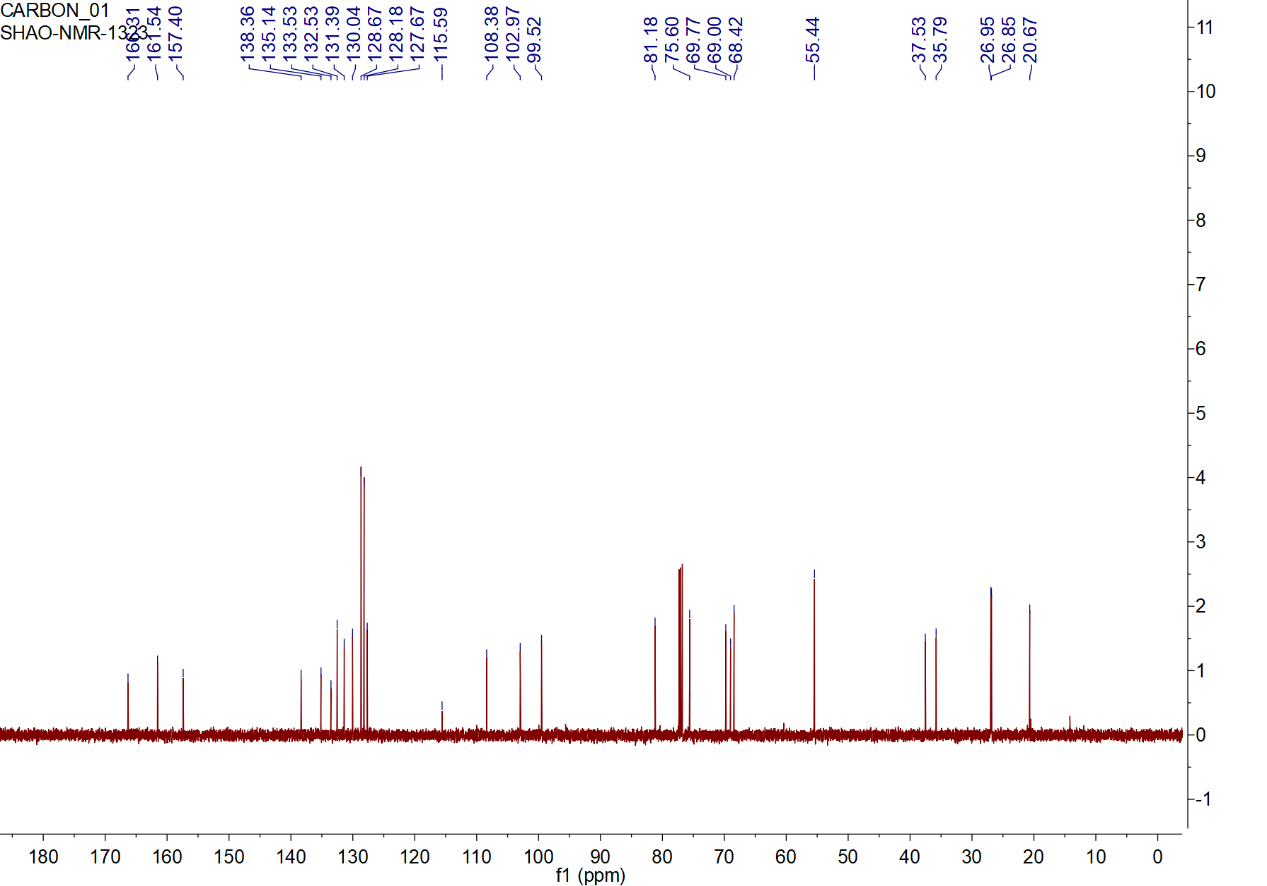


**Figure S120.** ^13^C NMR (125 MHz, CDCl_3_) spectrum of compound **42**

**Figure S121.** HRESIMS spectrum of compound **42**


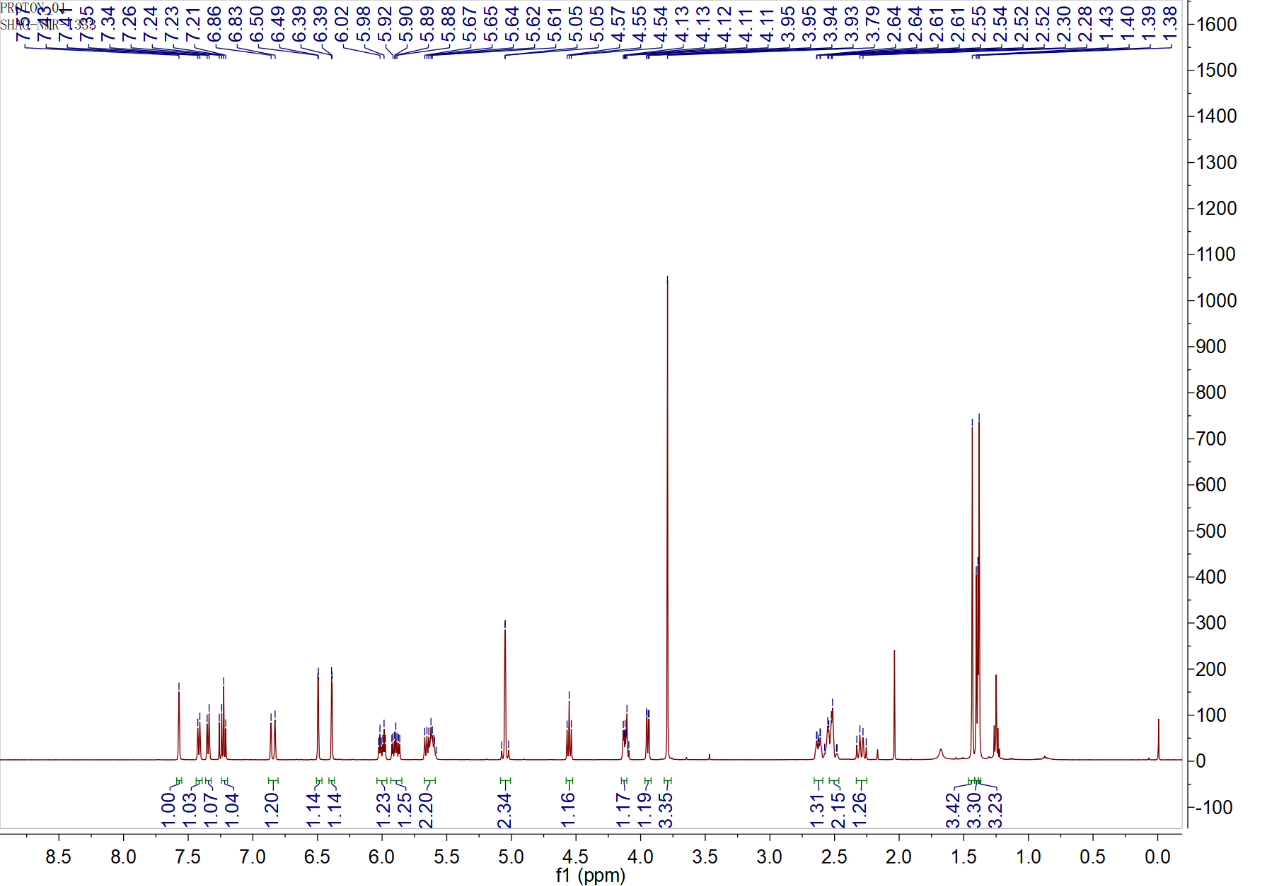


**Figure S122.** ^1^H NMR (500 MHz, CDCl_3_) spectrum of compound **43**


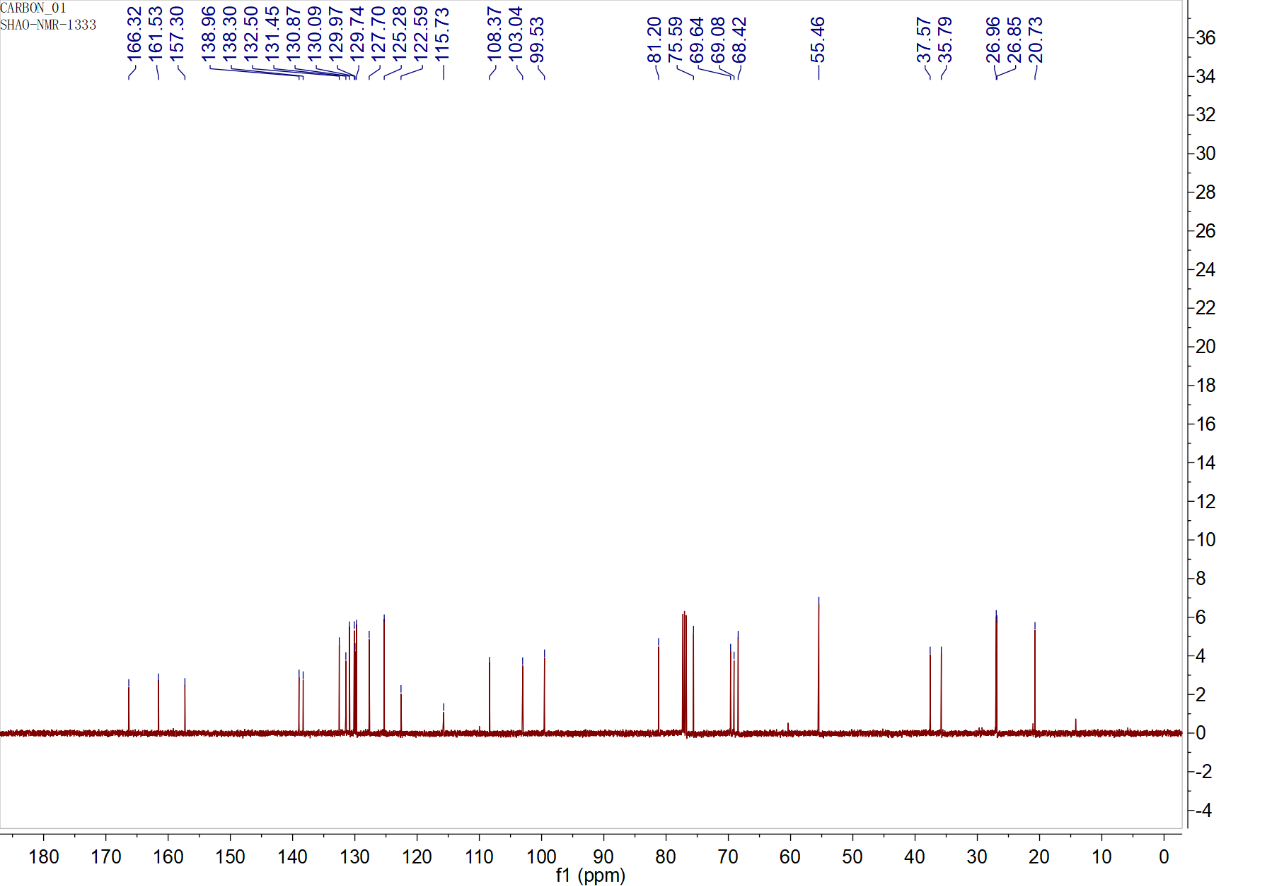


**Figure S123.** ^13^C NMR (125 MHz, CDCl_3_) spectrum of compound **43**

**Figure S124.** HRESIMS spectrum of compound **43**


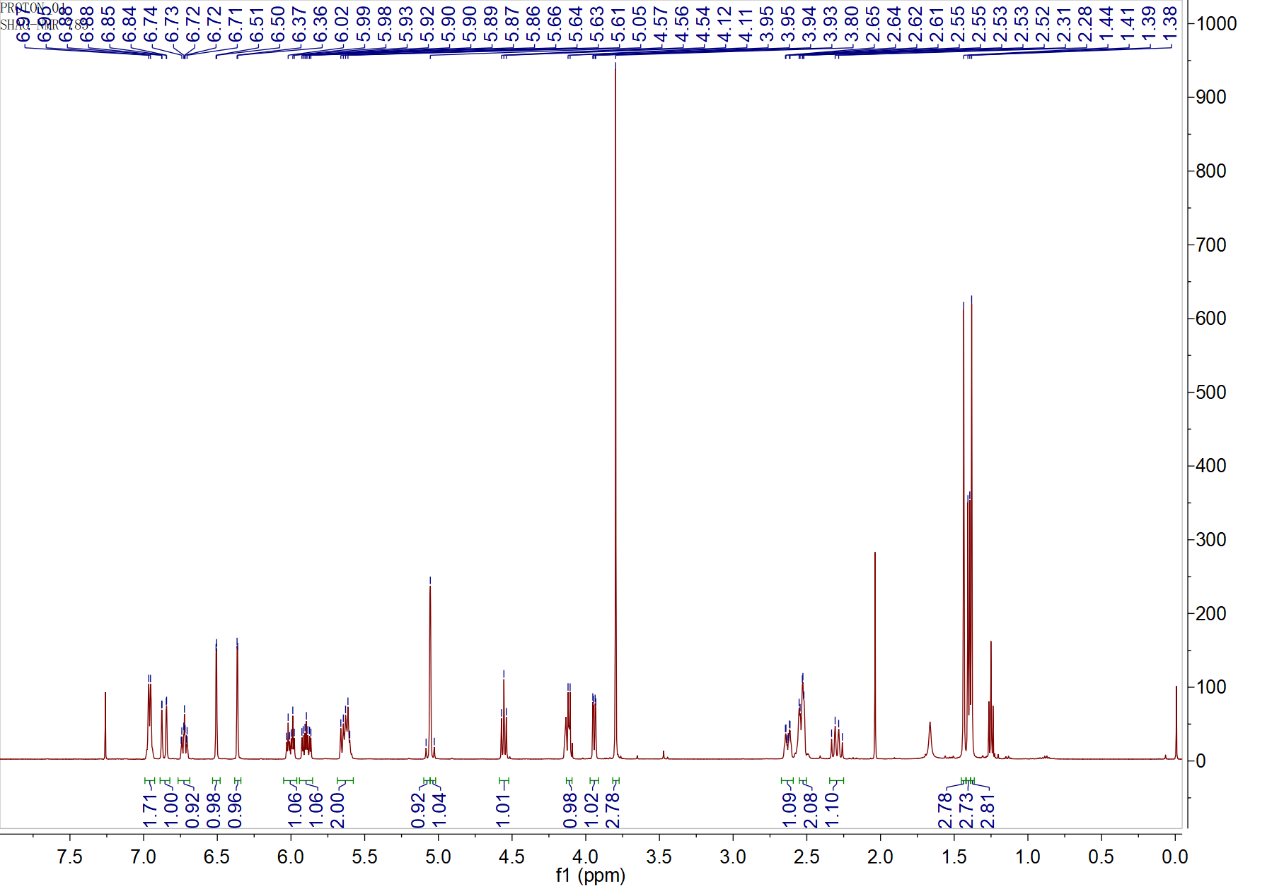


**Figure S125.** ^1^H NMR (500 MHz, CDCl_3_) spectrum of compound **44**


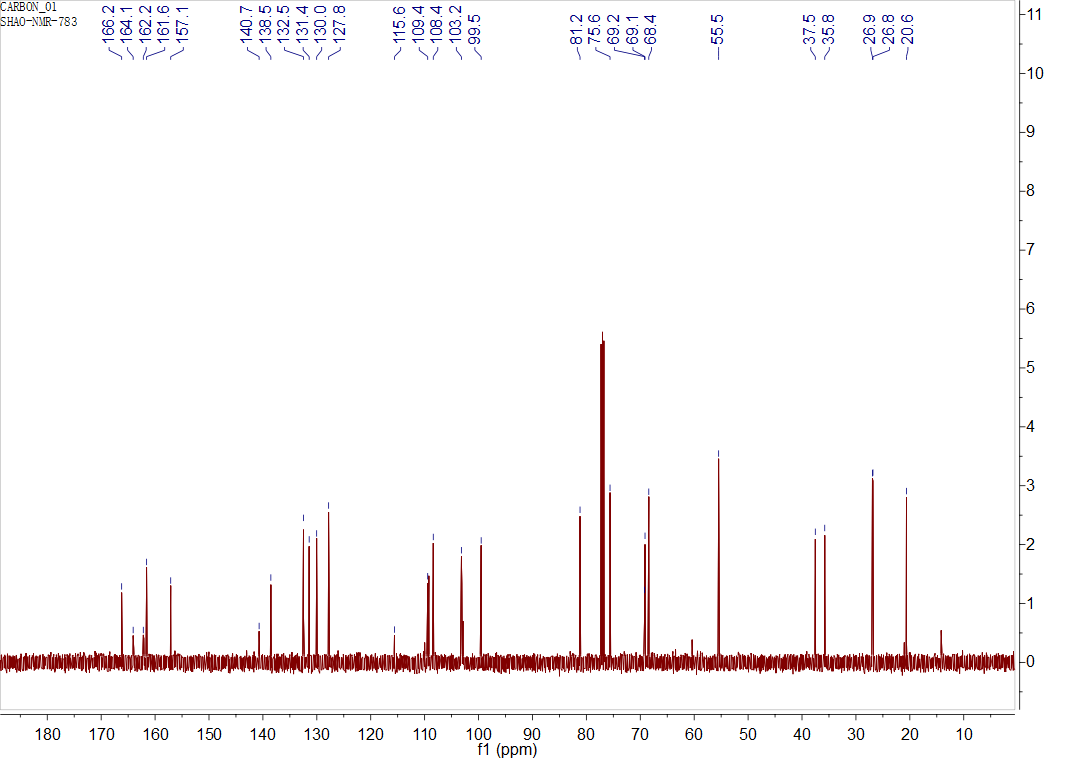


**Figure S126.** ^13^C NMR (125 MHz, CDCl_3_) spectrum of compound **44**

**Figure S127.** HRESIMS spectrum of compound **44**


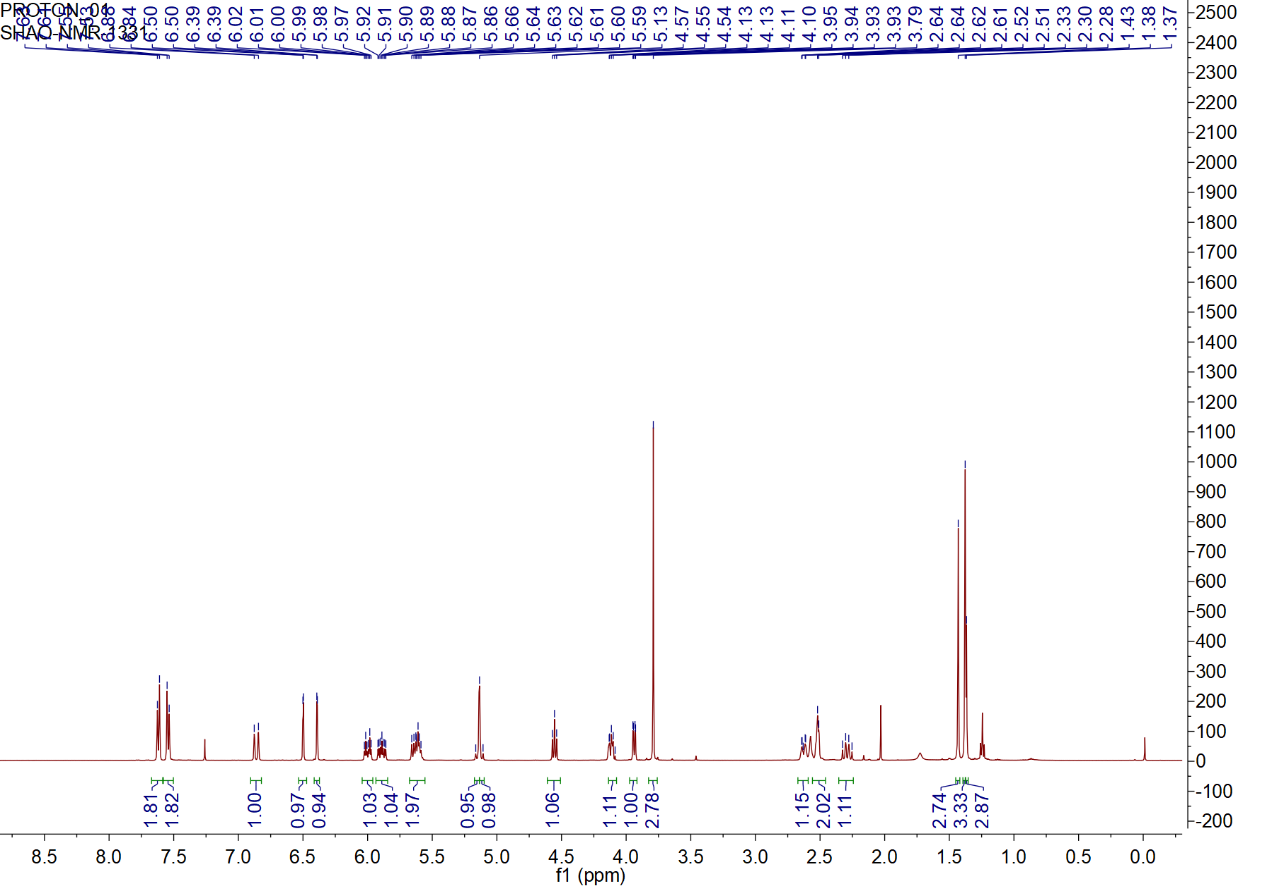


**Figure S128.** ^1^H NMR (500 MHz, CDCl_3_) spectrum of compound **45**


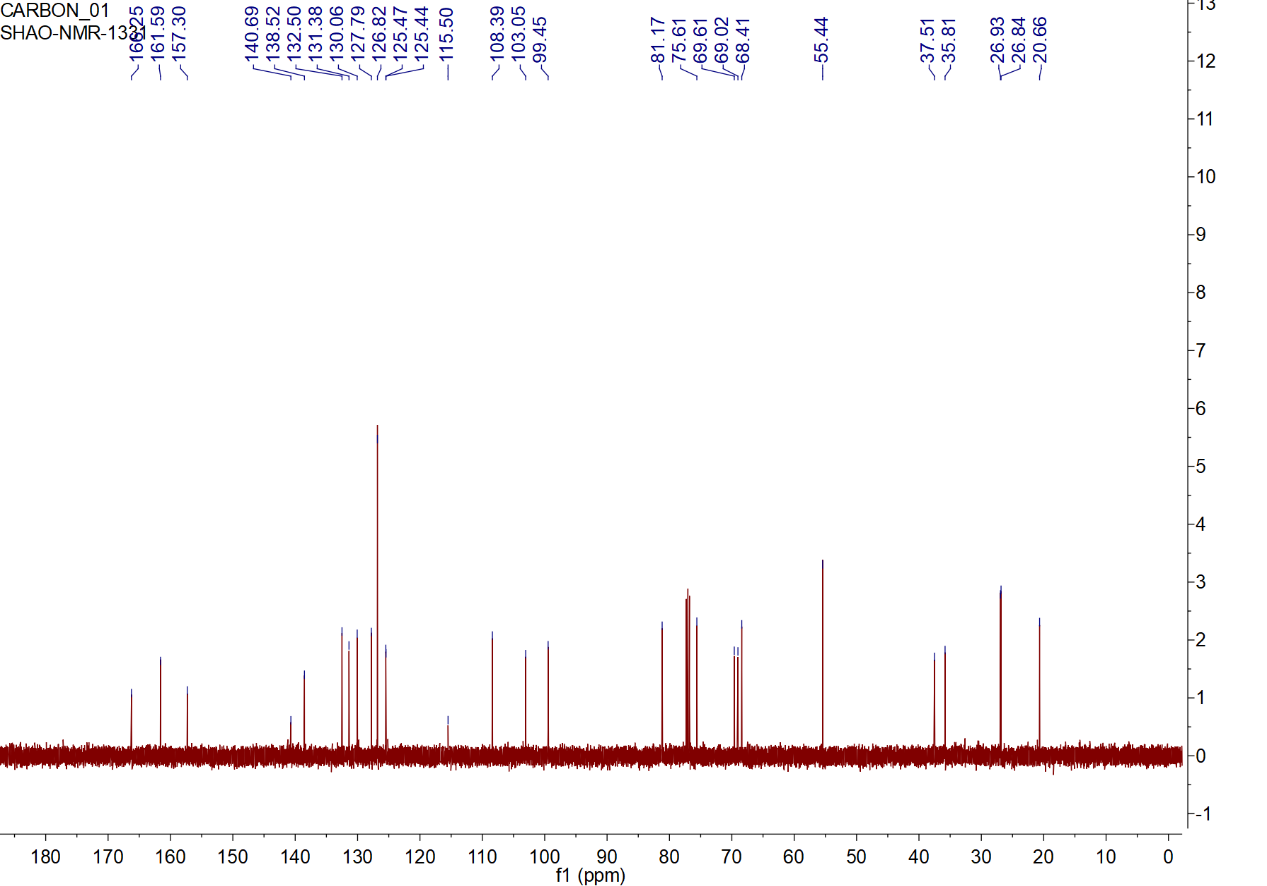


**Figure S129.** ^13^C NMR (125 MHz, CDCl_3_) spectrum of compound **45**

**Figure S130.** HRESIMS spectrum of compound **45**


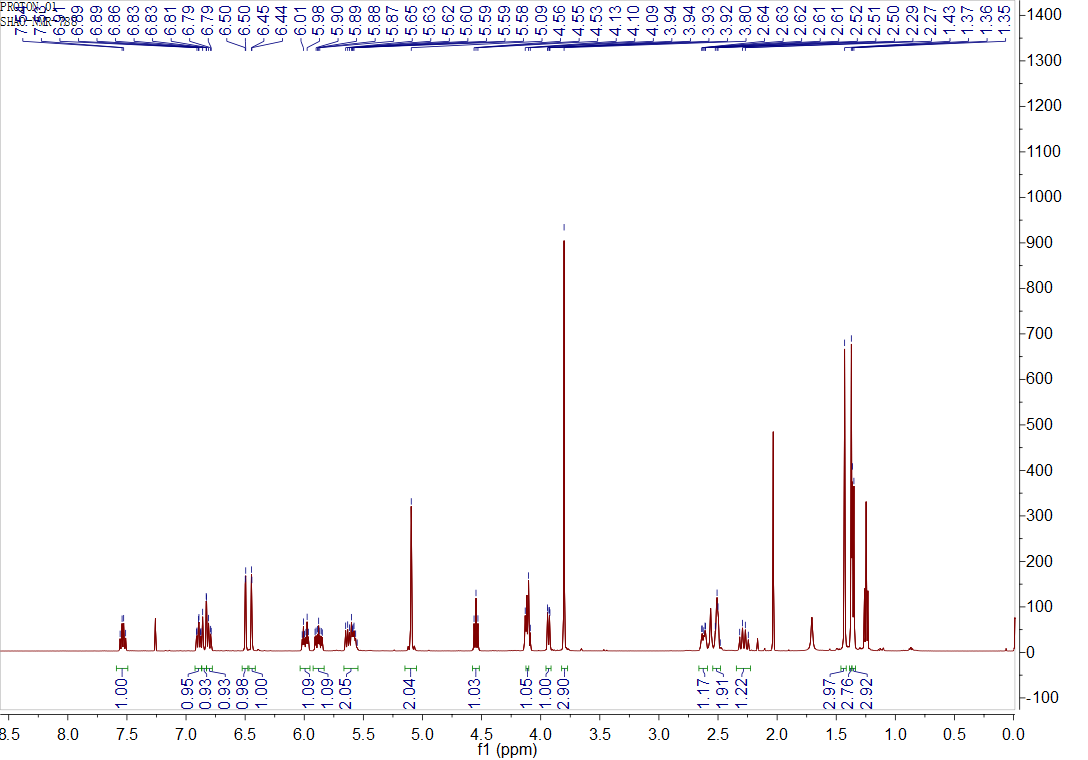


**Figure S131.** ^1^H NMR (500 MHz, CDCl_3_) spectrum of compound **46**


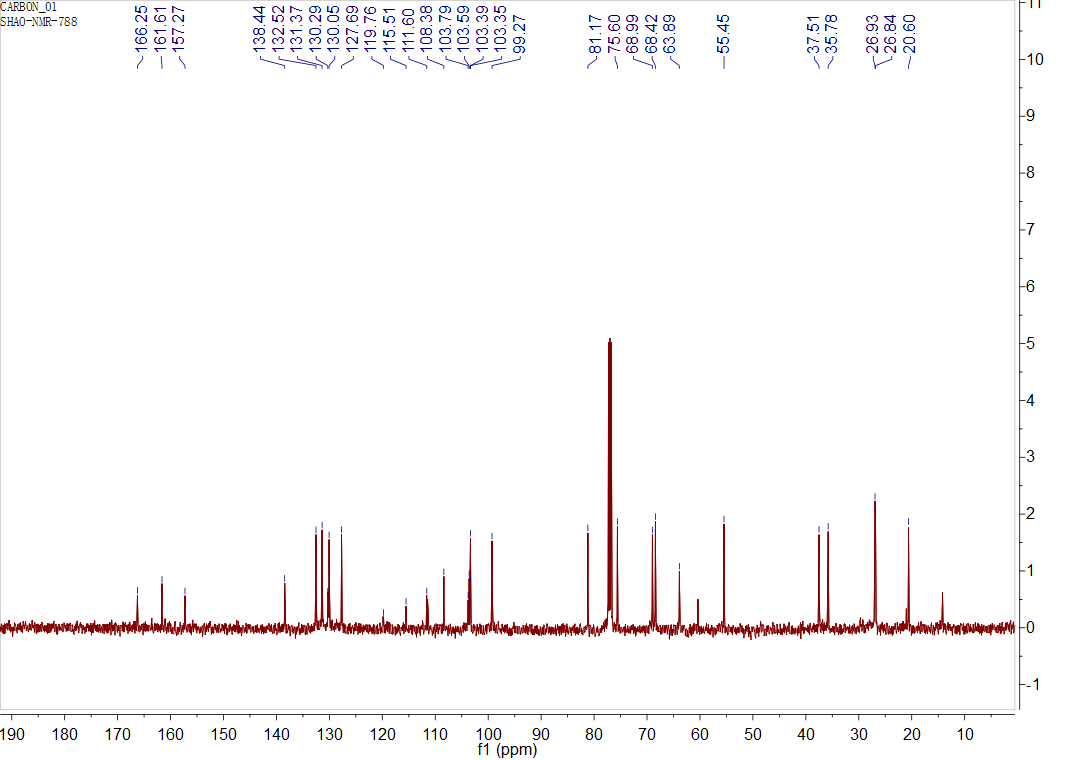


**Figure S132.** ^13^C NMR (125 MHz, CDCl_3_) spectrum of compound **46**

**Figure S133.** HRESIMS spectrum of compound **46**


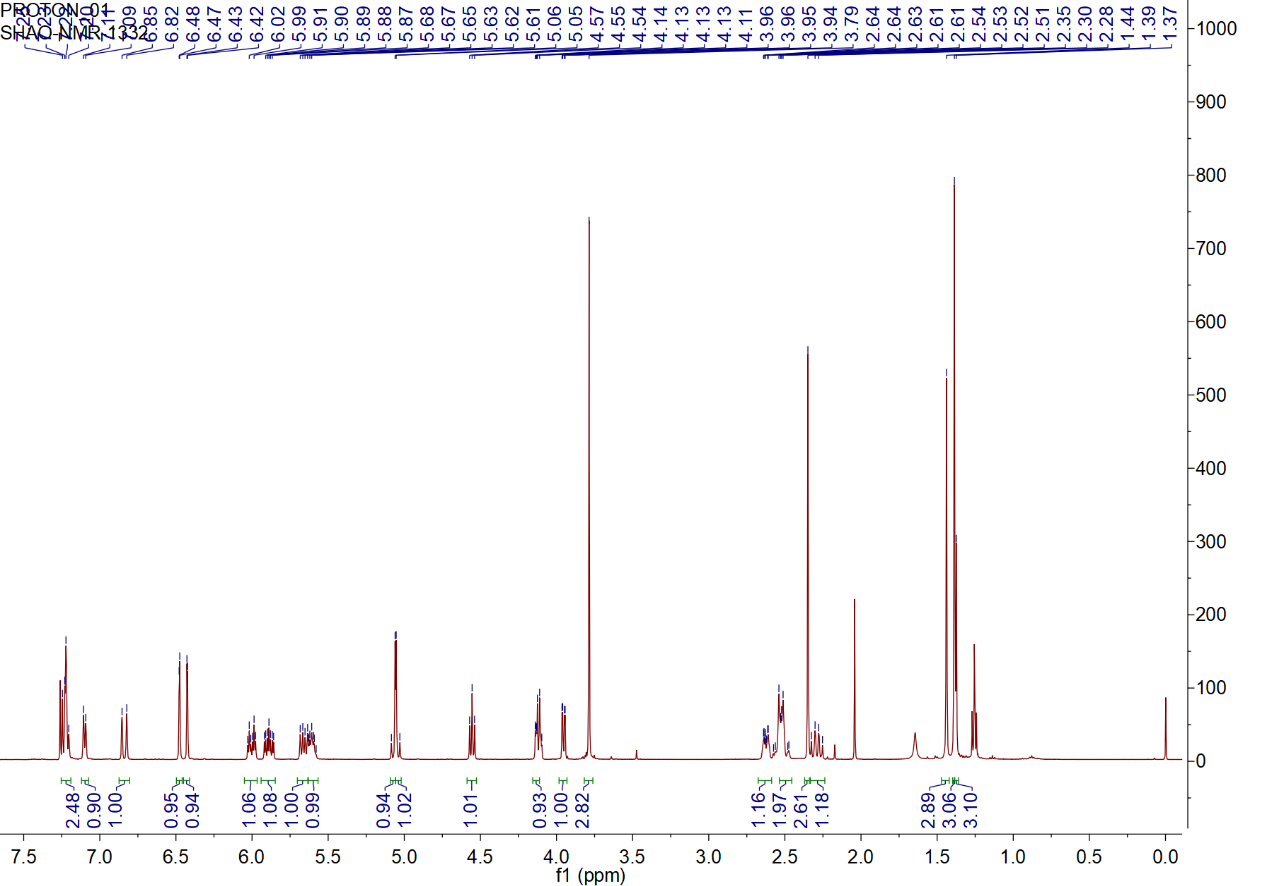


**Figure S134.** ^1^H NMR (500 MHz, CDCl_3_) spectrum of compound **47**


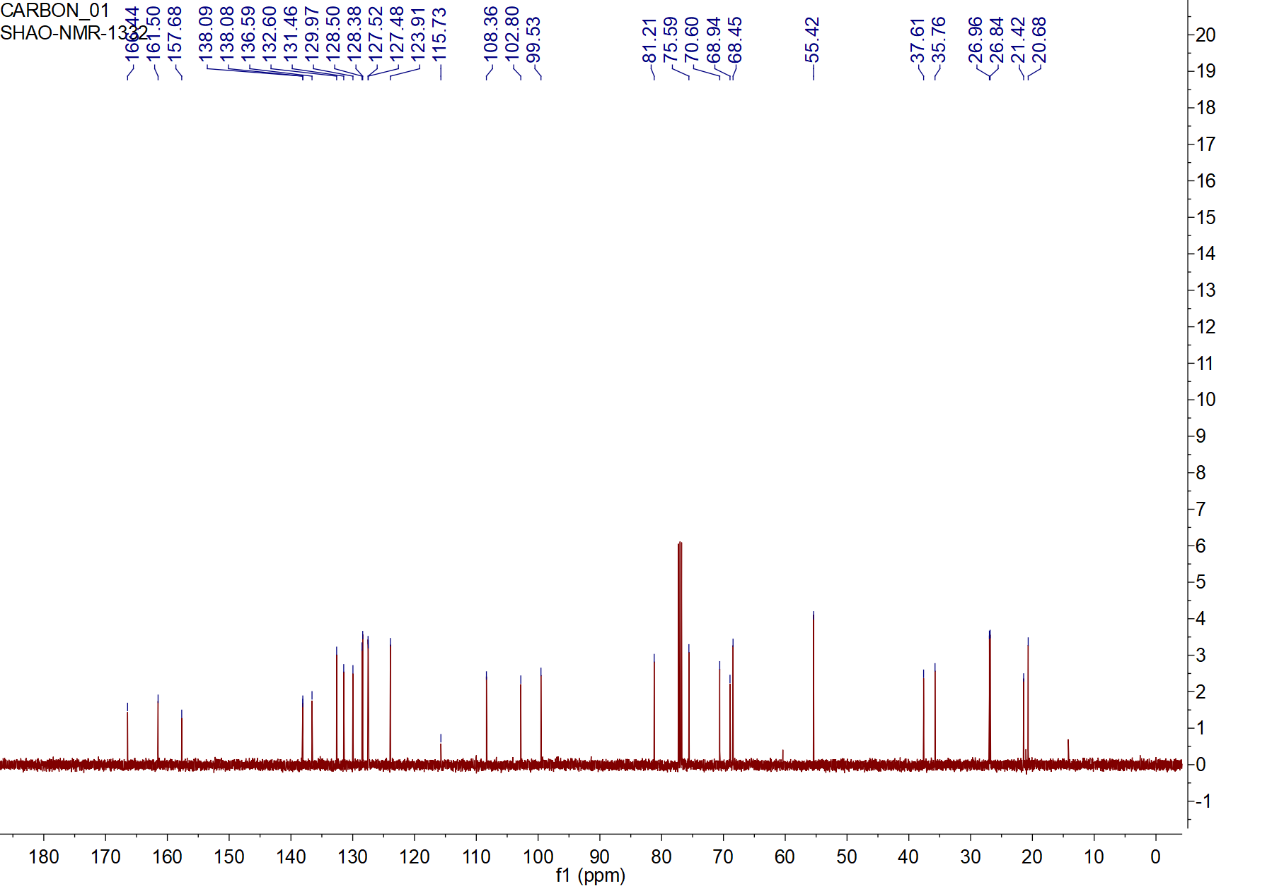


**Figure S135.** ^13^C NMR (125 MHz, CDCl_3_) spectrum of compound **47**

**Figure S136.** HRESIMS spectrum of compound **47**


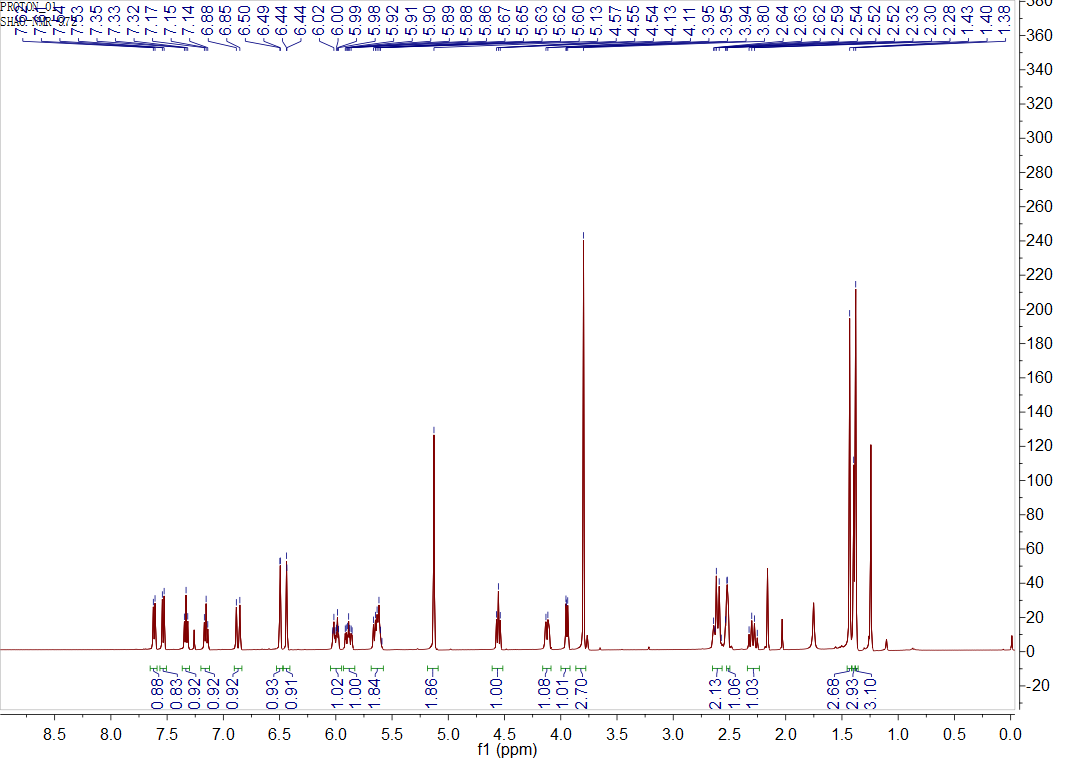


**Figure S137.** ^1^H NMR (500 MHz, CDCl_3_) spectrum of compound **48**


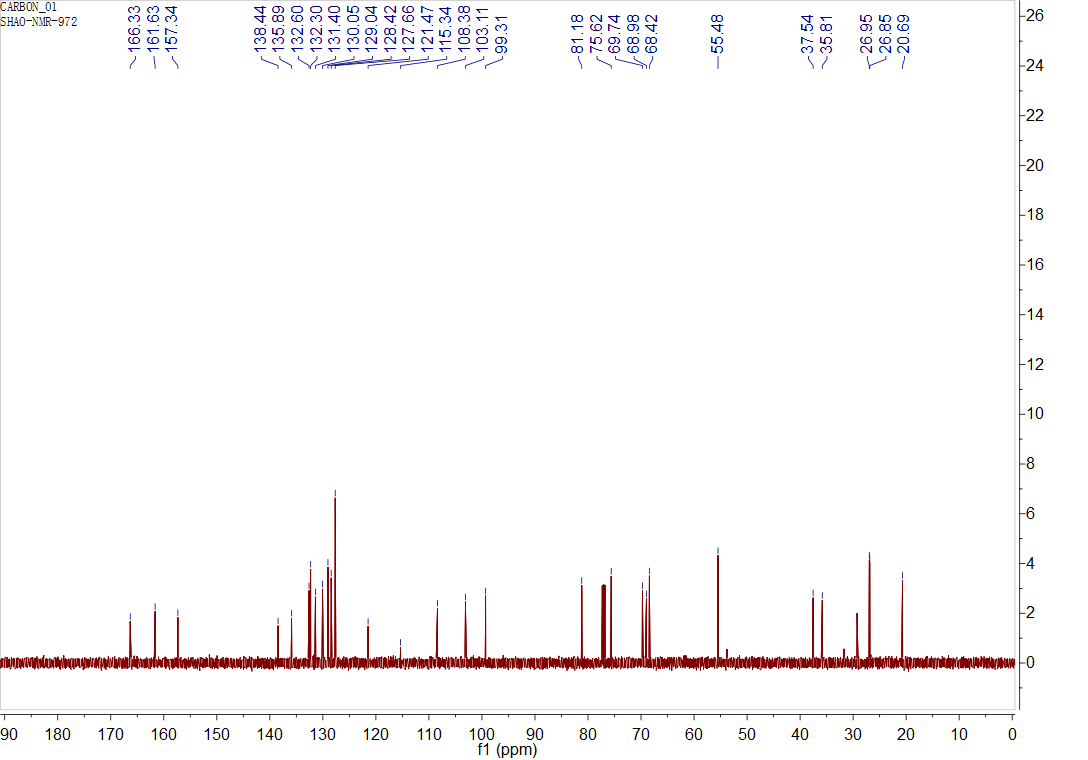


**Figure S138.** ^13^C NMR (125 MHz, CDCl_3_) spectrum of compound **48**

**Figure S139.** HRESIMS spectrum of compound **48**


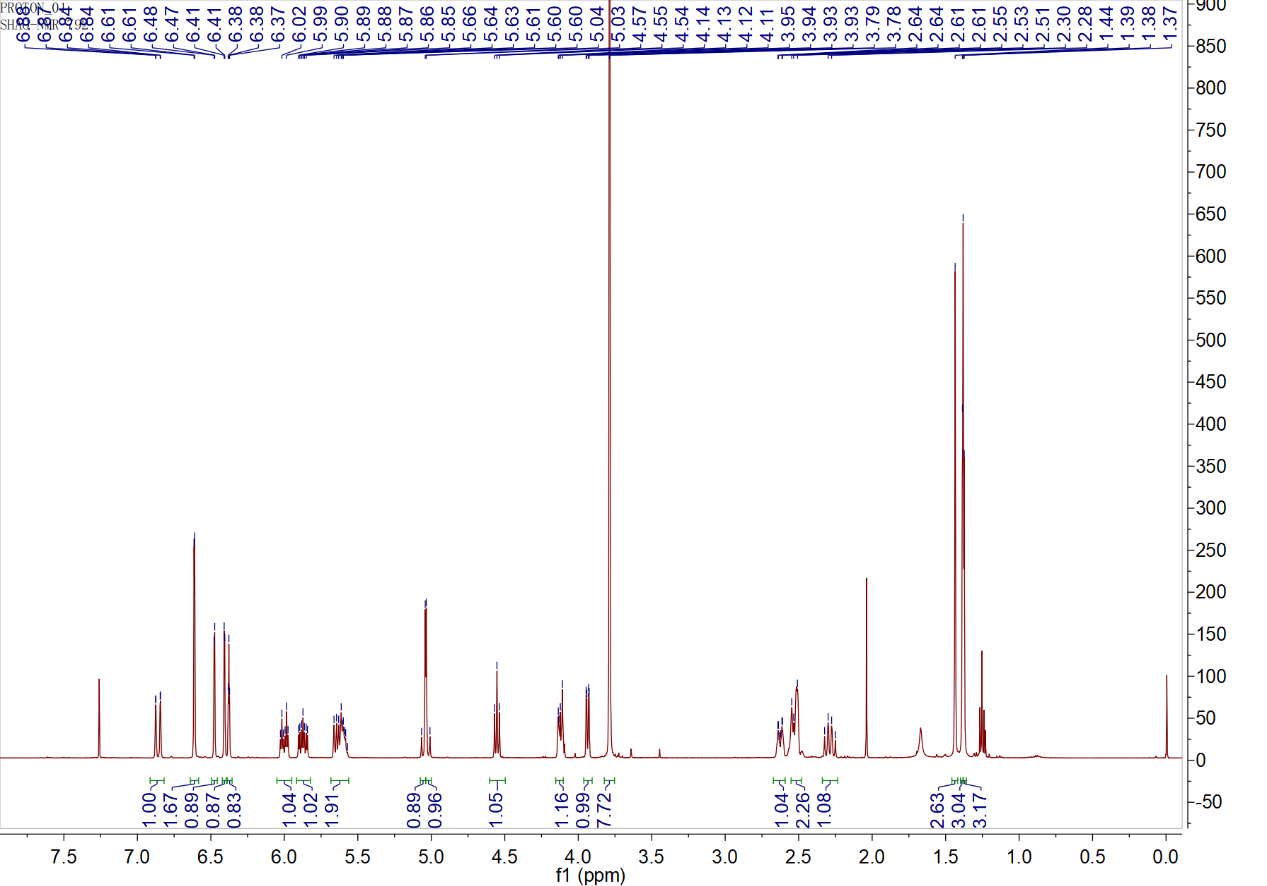


**Figure S140.** ^1^H NMR (500 MHz, CDCl_3_) spectrum of compound **49**


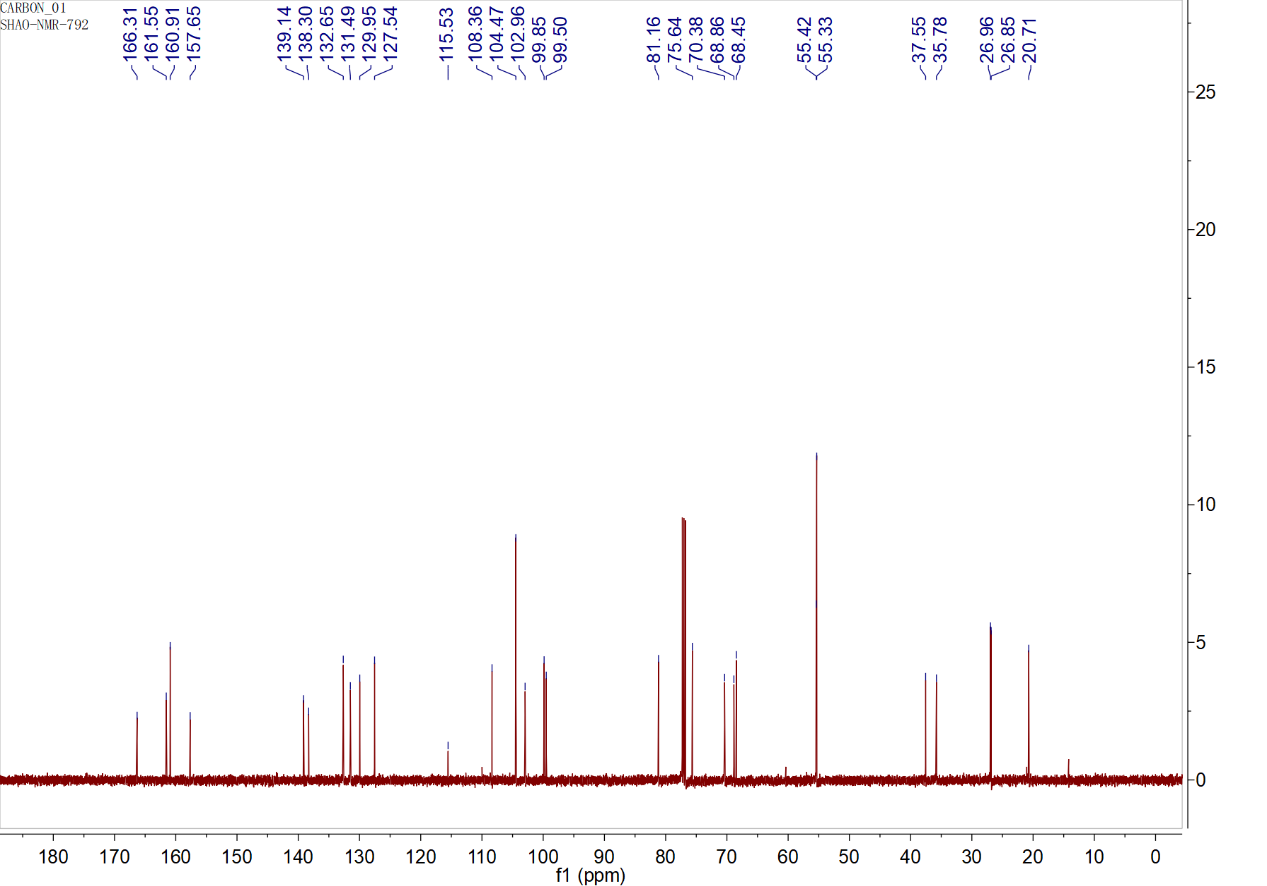


**Figure S141.** ^13^C NMR (125 MHz, CDCl_3_) spectrum of compound **49**

**Figure S142.** HRESIMS spectrum of compound **49**


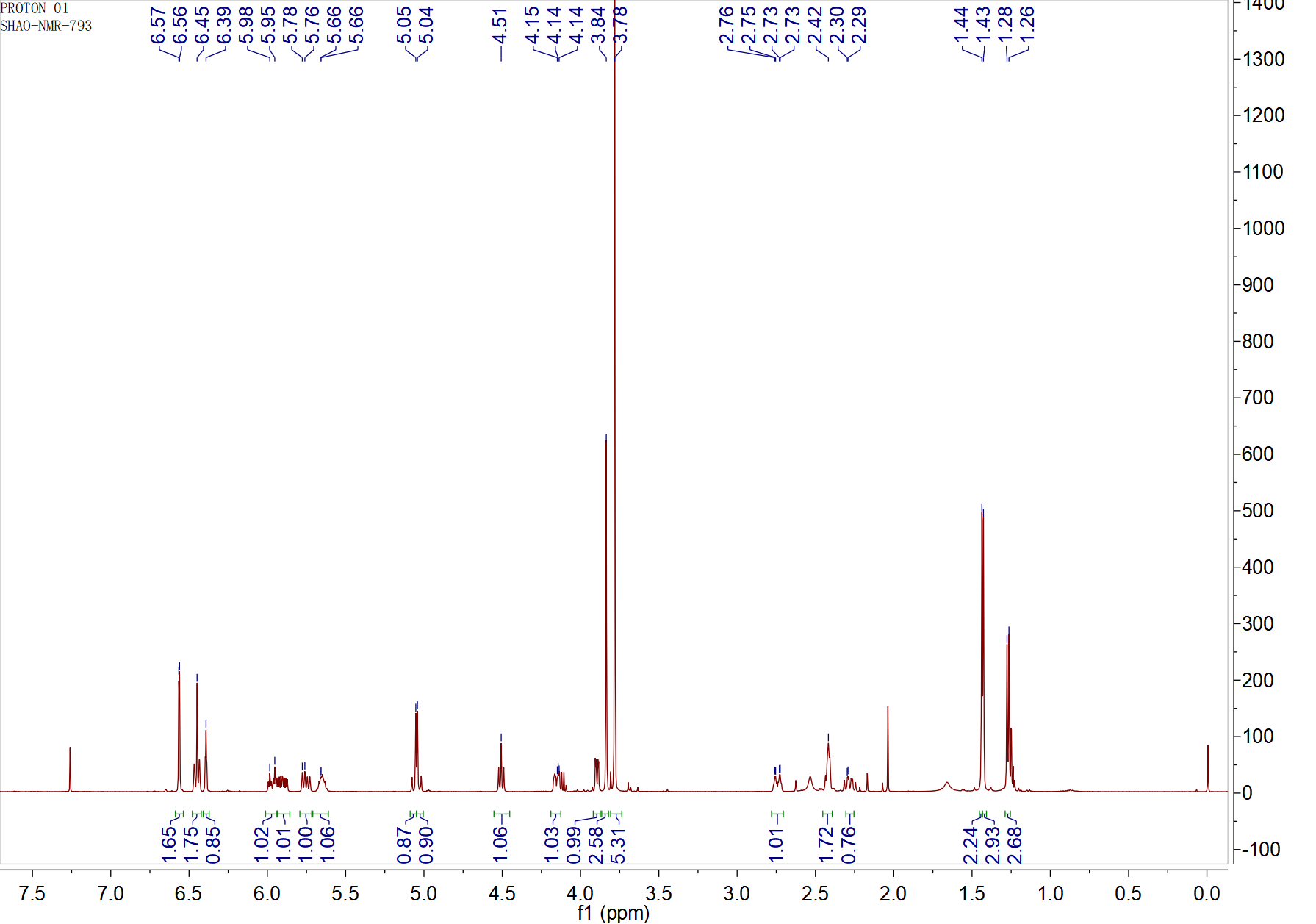


**Figure S143.** ^1^H NMR (500 MHz, CDCl_3_) spectrum of compound **50**


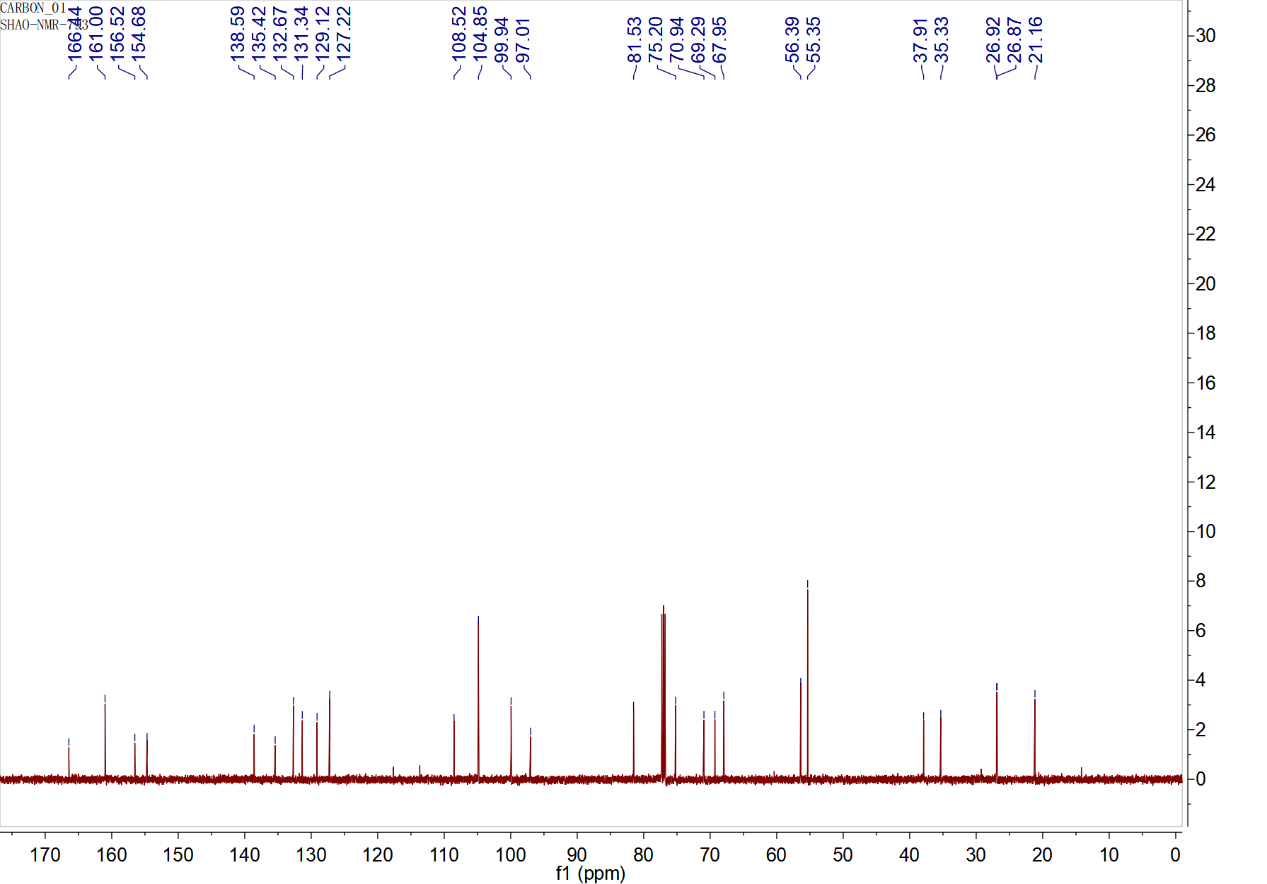


**Figure S144.** ^13^C NMR (125 MHz, CDCl_3_) spectrum of compound **50**

**Figure S145.** HRESIMS spectrum of compound **50**


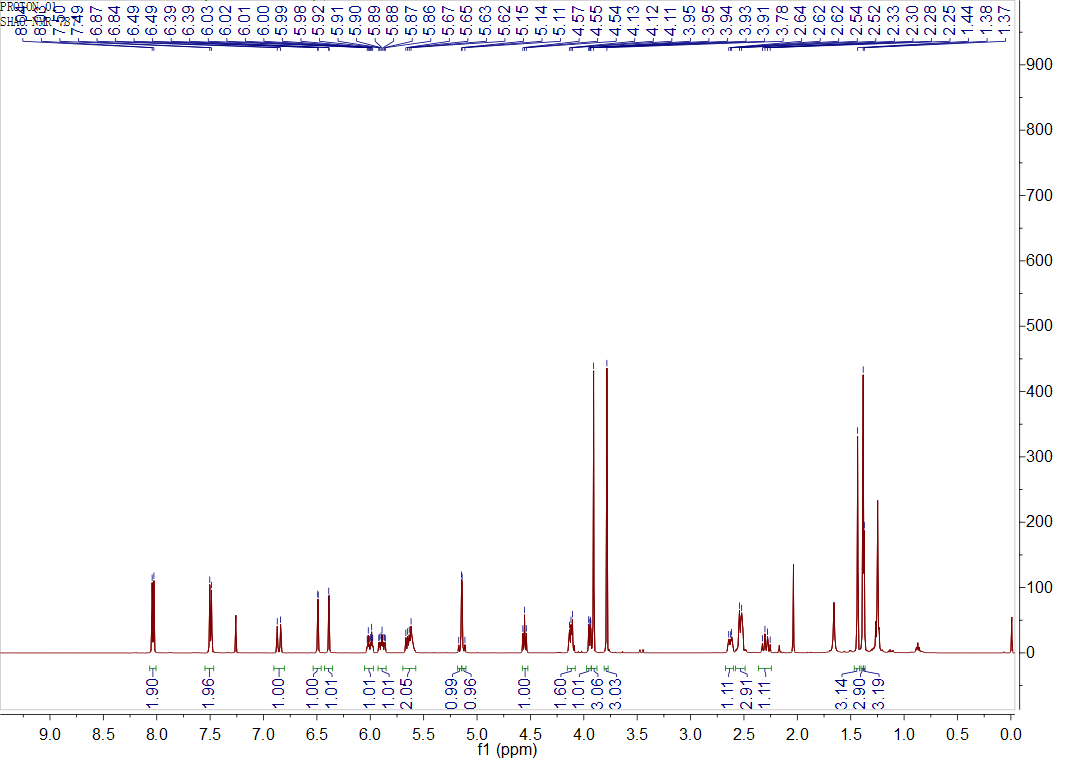


**Figure S146.** ^1^H NMR (500 MHz, CDCl_3_) spectrum of compound **51**


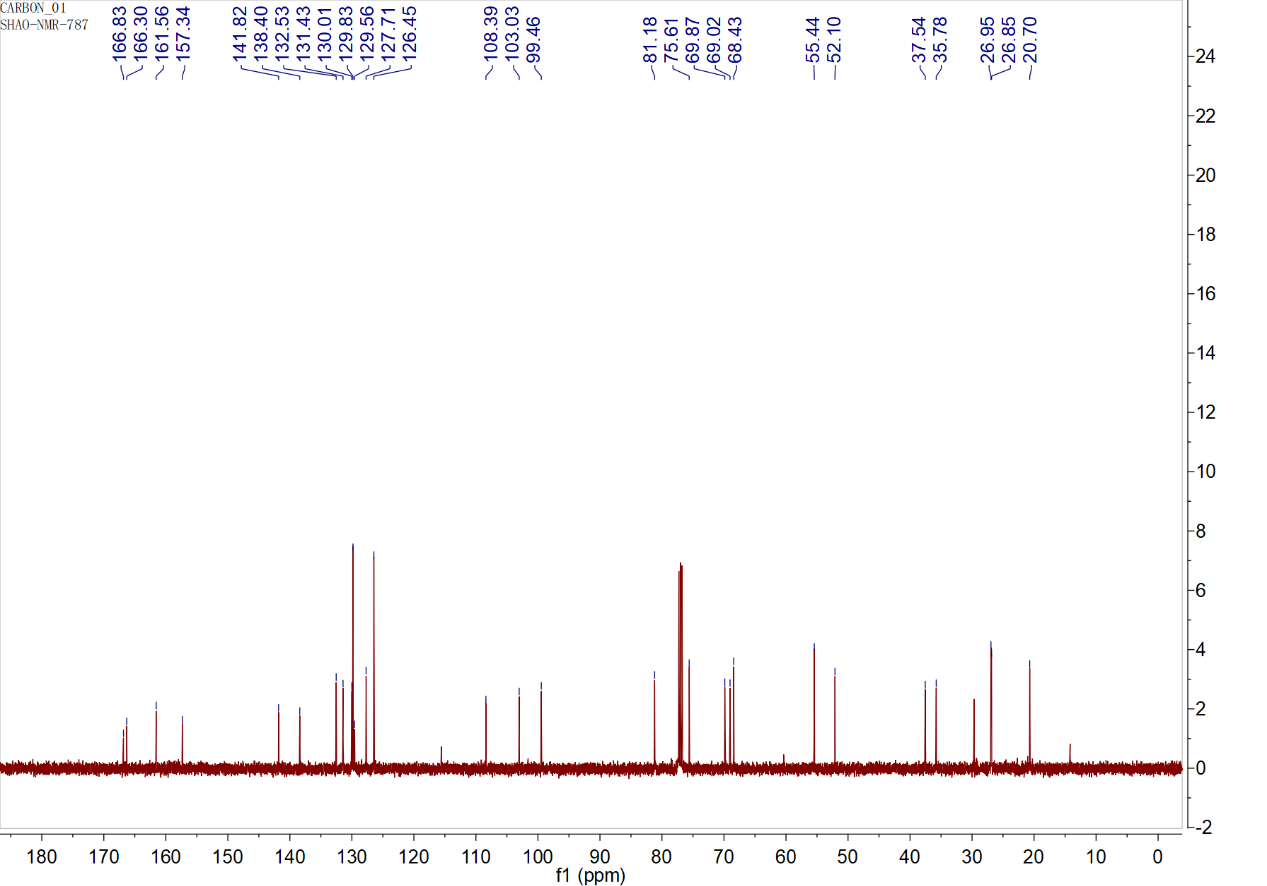


**Figure S147** ^13^C NMR (125 MHz, CDCl_3_) spectrum of compound **51**

**Figure S148.** HRESIMS spectrum of compound **51**

**Figure S149.** ^1^H NMR (500 MHz, CDCl_3_) spectrum of compound **52**

**Figure S150** ^13^C NMR (125 MHz, CDCl_3_) spectrum of compound **52**

**Figure S151.** HRESIMS spectrum of compound **52**

**Figure S152.** ^1^H NMR (500 MHz, CDCl_3_) spectrum of compound **53**

**Figure S153.** ^13^C NMR (125 MHz, CDCl_3_) spectrum of compound **53**

**Figure S154.** HRESIMS spectrum of compound **53**

**Figure S155.** ^1^H NMR (500 MHz, CDCl_3_) spectrum of compound **54**

**Figure S156.** ^13^C NMR (125 MHz, CDCl_3_) spectrum of compound **54**

**Figure S157.** HRESIMS spectrum of compound **54**

**Figure S158.** ^1^H NMR (500 MHz, CDCl_3_) spectrum of compound **55**

**Figure S159.** ^13^C NMR (125 MHz, CDCl_3_) spectrum of compound **55**

**Figure S160.** HRESIMS spectrum of compound **55**

**Figure S161.** ^1^H NMR (500 MHz, CDCl_3_) spectrum of compound **56**

**Figure S162.** ^13^C NMR (125 MHz, CDCl_3_) spectrum of compound **56**

**Figure S163.** HRESIMS spectrum of compound **56**

**Figure S164.** ^1^H NMR (500 MHz, CDCl_3_) spectrum of compound **57**

**Figure S165.** ^13^C NMR (125 MHz, CDCl_3_) spectrum of compound **57**

**Figure S166.** ^1^H NMR (500 MHz, CDCl_3_) spectrum of compound **58**

**Figure S167.** ^13^C NMR (125 MHz, CDCl_3_) spectrum of compound **58**

**Figure S168.** HRESIMS spectrum of compound **58**

**Figure S169.** ^1^H NMR (500 MHz, CDCl_3_) spectrum of compound **59**

**Figure S170.** ^13^C NMR (125 MHz, CDCl_3_) spectrum of compound **59**

**Figure S171.** HRESIMS spectrum of compound **59**

**Figure S172.** ^1^H NMR (500 MHz, CDCl_3_) spectrum of compound **60**

**Figure S173.** ^13^C NMR (125 MHz, CDCl_3_) spectrum of compound **60**

**Figure S174.** HRESIMS spectrum of compound **60**

**Figure S175.** ^1^H NMR (500 MHz, CDCl_3_) spectrum of compound **61**

**Figure S176.** ^13^C NMR (125 MHz, CDCl_3_) spectrum of compound **61**

**Figure S177.** HRESIMS spectrum of compound **61**

**Figure S178.** ^1^H NMR (500 MHz, CDCl_3_) spectrum of compound **62**

**Figure S179.** ^13^C NMR (125 MHz, CDCl_3_) spectrum of compound **62**

**Figure S180.** HRESIMS spectrum of compound **62**

**Figure S181.** ^1^H NMR (500 MHz, CDCl_3_) spectrum of compound **63**

**Figure S182.** ^13^C NMR (125 MHz, CDCl_3_) spectrum of compound **63**

**Figure S183.** HRESIMS spectrum of compound **63**

**Figure S184.** ^1^H NMR (500 MHz, CDCl_3_) spectrum of compound **64**

**Figure S185.** ^13^C NMR (125 MHz, CDCl_3_) spectrum of compound **64**

**Figure S186.** HRESIMS spectrum of compound **64**

**Figure S187.** ^1^H NMR (500 MHz, CDCl_3_) spectrum of compound **65**

**Figure S288.** ^13^C NMR (125 MHz, CDCl_3_) spectrum of compound **65**

**Figure S189.** HRESIMS spectrum of compound **65**

**Figure S190.** ^1^H NMR (500 MHz, CDCl_3_) spectrum of compound **66**

**Figure S191.** ^13^C NMR (125 MHz, CDCl_3_) spectrum of compound **66**

**Figure S192.** HRESIMS spectrum of compound **66**

**Figure S193.** ^1^H NMR (500 MHz, CDCl_3_) spectrum of compound **67**

**Figure S194.** ^13^C NMR (125 MHz, CDCl_3_) spectrum of compound **67**

**Figure S195.** HRESIMS spectrum of compound **67**

**Figure S196.** ^1^H NMR (500 MHz, CDCl_3_) spectrum of compound **68**

**Figure S197.** ^13^C NMR (125 MHz, CDCl_3_) spectrum of compound **68**

**Figure S198.** HRESIMS spectrum of compound **68**

**Figure S199.** ^1^H NMR (500 MHz, CDCl_3_) spectrum of compound **69**

**Figure S200.** ^13^C NMR (125 MHz, CDCl_3_) spectrum of compound **69**

**Figure S201.** ^1^H NMR (500 MHz, CDCl_3_) spectrum of compound **70**

**Figure S202.** ^13^C NMR (125 MHz, CDCl_3_) spectrum of compound **70**

**Figure S203.** HRESIMS spectrum of compound **70**

**Figure S204.** ^1^H NMR (500 MHz, CDCl_3_) spectrum of compound **71**

**Figure S205.** ^13^C NMR (125 MHz, CDCl_3_) spectrum of compound **71**

**Figure S206.** HRESIMS spectrum of compound **71**
